# Overall survival comparison between pediatric and adult Ewing sarcoma of bone and adult nomogram construction: a large population-based analysis

**DOI:** 10.3389/fped.2023.1103565

**Published:** 2023-05-23

**Authors:** Chi-Jen Hsu, Yongguang Ma, Peilun Xiao, Chia-Chien Hsu, Dawei Wang, Mei Na Fok, Rong Peng, Xianghe Xu, Huading Lu

**Affiliations:** ^1^ Department of Orthopedics, The Fifth Affiliated Hospital of Sun Yat-sen University, Zhuhai, China; ^2^ College of Medicine, Chang Gung University, Taoyuan, Taiwan; ^3^ Centro Hospitalar Conde São Januário, Macau SAR, China

**Keywords:** Ewing sarcoma, nomogram, SEER, PSM, overall survival.

## Abstract

**Background:**

Ewing sarcoma (ES) is a common primary bone tumor in children. Our study aimed to compare overall survival (OS) between pediatric and adult bone ES patients, identify independent prognostic factors and develop a nomogram for predicting OS in adult patients with ES of bone.

**Methods:**

We retrospectively analyzed data for the 2004–2015 period from the Surveillance, Epidemiology, and End Results (SEER) database. To guarantee well-balanced characteristics between the comparison groups, propensity score matching (PSM) was used. Kaplan–Meier (KM) curves were used to compare OS between pediatric and adult patients with ES of bone. Univariate and multivariate Cox regression analyses were used to screen independent prognostic factors for ES of bone, and a prognostic nomogram was constructed by using the factors identified. The prediction accuracy and clinical benefit were evaluated using receiver operating characteristic (ROC) curves, areas under the curves (AUCs), calibration curves, and decision curve analysis (DCA).

**Results:**

Our results showed that adult ES patients had lower OS than younger ES patients. Age, surgery, chemotherapy, and TNM stage were independent risk factors for bone ES in adults and were used to develop a nomogram. AUCs for 3-, 5-, and 10-year OS were 76.4 (67.5, 85.3), 77.3 (68.6, 85.9) and 76.6 (68.6, 84.5), respectively. Calibration curves and DCA results indicated excellent performance for our nomogram.

**Conclusion:**

We found that ES pediatric patients have better OS than adult ES patients, and we constructed a practical nomogram to predict the 3-, 5- and 10-year OS of adult patients with ES of bone based on independent prognostic factors (age, surgery, chemotherapy, T stage, N stage and M stage).

## Introduction

In the 2020 WHO classification, ES is grouped with round cell sarcomas with EWSR1-nonerythroblast transformation specific (ETS) fusions, CIC-rearranged sarcomas, and sarcomas with BCOR genetic alterations in a new chapter named “undifferentiated small round cell sarcomas of bone and soft tissue” to represent a more accurate biological landscape (1). Over the last two decades, ES treatment has advanced significantly, with many clinical trials on different treatment modalities and combinations (2). However, these treatments may be accompanied by acute and chronic side effects that may impair patient quality of life.

To date, few articles have presented a comparison of overall survival (OS) between pediatric and adult patients with ES of bone, and there is no nomogram currently available for predicting bone ES in adult patients. Thus, we utilized the Surveillance, Epidemiology, and End Results (SEER) database to identify independent risk factors for adult patients, compare OS between pediatric and adult patients with ES of bone and create a clinical nomogram to predict the OS of adult patients with ES of bone. A nomogram is a mathematical formula or algorithm that can predict a particular end point (3). Under the American legal system, the legal age of majority is 18, and children under the age of 18 cannot make healthcare decisions without their parents' permission (4). Therefore, we constructed a nomogram that predicts OS prognosis of adults with ES of bone and offers both clinicians and patients different perspectives on treatment.

## Materials & methods

### Data source and patient selection criteria

Data for patients diagnosed with ES between 2004 and 2015 were obtained from the SEER 17 registry online database using SEER*Stat 8.4.0.1 software. The site code for histological types was limited to ICD-O-3:9260/3: ES, and the tumor site was set as C40.0-C41.9. The inclusion criteria were as follows: (1) diagnosed between 2004 and 2015 and (2) confirmation by positive histology. The exclusion criteria were as follows: (1) incomplete information and (2) ES not the primary tumor. Only 756 patients met these criteria. To compare the OS of pediatric and adult patients, we divided the patients into two groups (<18 and ≥18 years old).

The variables obtained from the SEER database included age, sex, race, surgery, radiation, chemotherapy, T stage, N stage, M stage and primary site. We calculated the cutoff value for age using X-tile 3.6.1, which can subclassify tumors based on biomarker expression and has a wide variety of clinical applications (5). TNM stage was defined following the 6th TNM staging system. The other clinicopathological features of the patients were labeled as follows: sex (female, male), race (white, black, others), surgery (yes, no), radiation (yes, no/unknown), chemotherapy (yes, no/unknown), and primary site (axial, extremity).

### Statistical analysis

We used univariate and multivariate Cox regression analyses to identify independent prognostic factors and Kaplan–Meier (KM) curves to identify whether the OS of pediatric and adult bone ES patients differed significantly. To reduce the effects of biases and confounding variables, we used the chi-square test and Fisher's test to determine variables that were imbalanced among the baseline characteristics (*P* < 0.05). Propensity score matching (PSM) is a method used to achieve balanced variables between two groups and decrease selection bias in nonrandomized research (6), and we performed PSM analysis to balance the variables. For PSM, the caliper was set to 0.02, and nearest neighbor matching (in a 1:1 ratio) was performed to create matching pairs between the pediatric and adult groups. After PSM, we constructed a KM curve to evaluate differences in the OS of the pediatric and adult patients with ES of bone.

We performed univariate Cox and multivariate Cox regression analyses to identify independent risk factors. The hazard ratios (HRs) and 95% confidence intervals (95% CIs) of the variables were calculated (7). We constructed our nomogram based on the identified independent prognostic factors by *R* with the rms package (8). Using these independent risk factors, we created a nomogram for predicting OS at 3, 5, and 10 years. We evaluated our nomogram's prediction ability using receiver operating characteristic (ROC) curves and areas under the curve (AUC). Then, using a bootstrapping procedure with 1,000 resamples, calibration curves were built to assess the degree of agreement between the actual and predicted probabilities based on our nomogram (9). We performed decision curve analysis (DCA) to compare our nomogram with the TNM nomogram in terms of clinical usefulness and net benefits (10).

All statistical analyses were carried out using SPSS version 26.0 (IBM, Chicago, IL, USA), R software (version 4.2.1; http://www.Rproject.org) for Windows, and X-tile 3.6.1. In all our statistical tests, a *P* value <0.05 was statistically significant.

## Results

### Patient baseline characteristics

**Figure 1:**
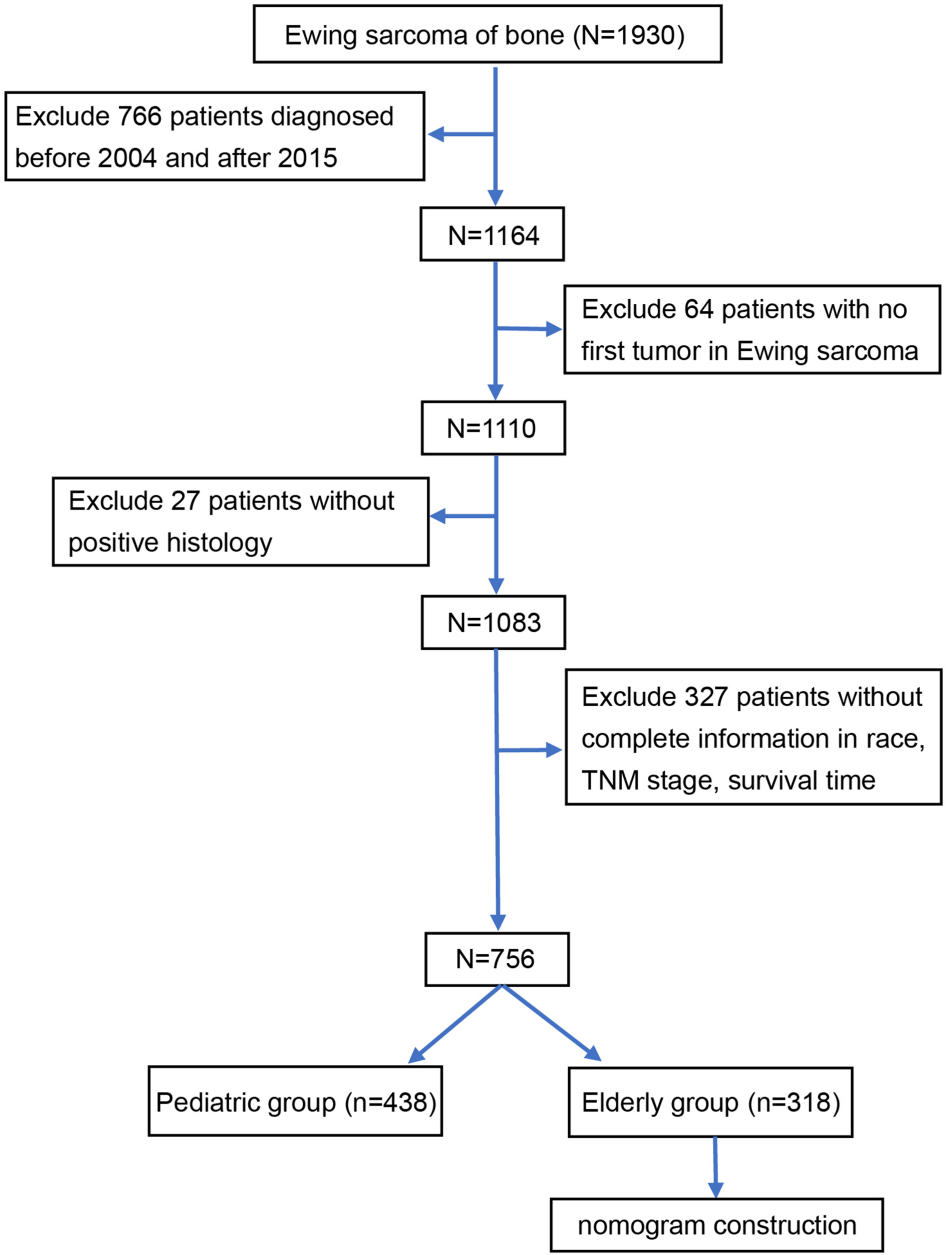
Flow diagram of the selection process for the patient cohort from the SEER database. Finally, 756 patients were included in our study and divided into an adult group (*n* = 428) and a pediatric group (*n* = 318). SEER Surveillance, Epidemiology, and End Results.

**Figure 2:**
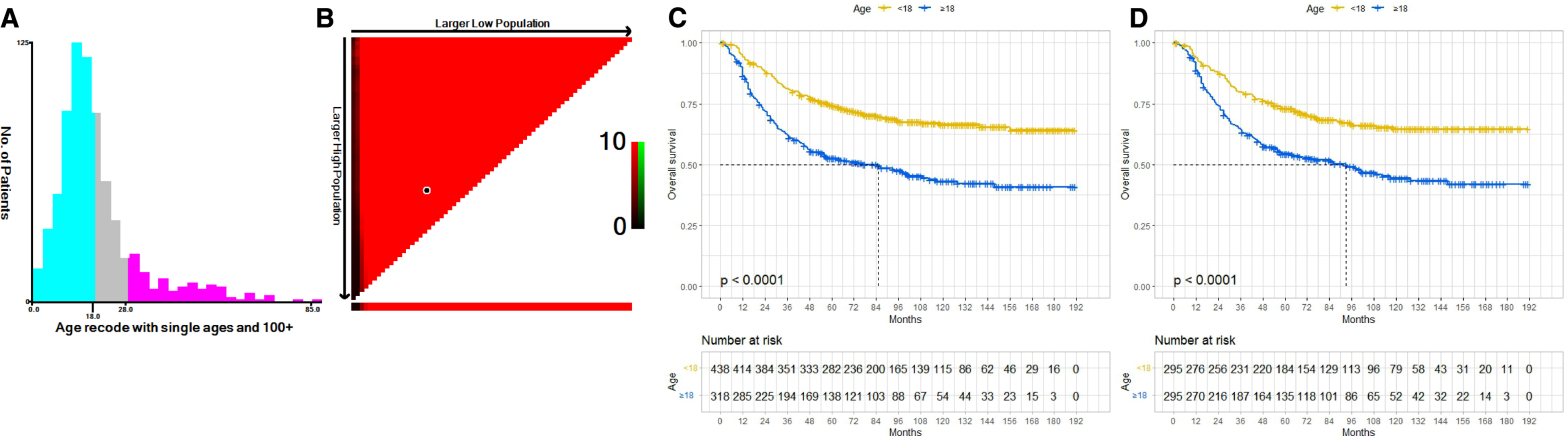
The optimal cutoff value of age was identified by X-tile analysis (**A,B**). The optimal age cutoffs were 18 and 28 years. KM curves of OS for our data before PSM (**C**) and after PSM (**D**). Both before and after PSM, the OS of the pediatric group was better than that of the adult group. OS, overall survival; KM, Kaplan–Meier; PSM, Propensity Score Matching.

Figure 1 illustrates the data selection process used in our investigation; a total of 756 patients were selected for the study. We used X-tile software to investigate the association between patient age and risk of mortality. The X-tile results showed that the optimal cutoff values of age in terms of OS were 18 and 28 years, and survival curves were plotted using the KM method for those age subgroups to assess OS (Figures 2A,B). To study the effect of differences in survival between pediatric and adult ES patients, we divided the patients into two distinct groups: a pediatric group (age <18, *n* = 438) and an adult group (age ≥18, *n* = 318).

### Baseline information before and after PSM

Based on the results of chi-square tests and Fisher tests, obvious differences in race, sex, surgery, and chemotherapy were found between the two groups. This indicated that the baseline characteristics of the two groups were not well balanced. After matching, 295 pediatric patients and 295 adult patients were enrolled in the final analysis, and the baseline characteristics were balanced in the final model (Table 1).

**Table 1:** Comparison of baseline variables between the pediatric and adult groups before and after PSM.

	Before PSM			After PSM		
	Pediatric	Adult	*P* value	Pediatric	Adult	*P* value
Race			0.008			1.000
White	391	280		267	267	
Black	7	17		7	7	
Others	40	21		21	21	
Sex			0.041			1.000
Male	263	214		199	199	
Female	175	104		96	96	
T stage			0.159			0.418
T1	222	147		143	137	
T2	195	146		136	134	
T3	21	25		16	24	
N stage			0.624			0.210
N0	405	297		269	277	
N1	33	21		26	18	
M stage			0.215			0.786
M0	325	223		210	207	
M1	113	95		85	88	
Surgery			0.003			1
Yes	296	181		171	171	
No	142	137		124	124	
Radiation			0.566			0.934
Yes	207	157		151	150	
No/Unknown	231	161		144	145	
Chemotherapy			0.002			1.000
Yes	433	303		293	293	
No/Unknown	5	15		2	2	
Primary site			0.200			0.458
Extremity	219	144		143	134	
Axial	219	174		152	161	

PSM, Propensity Score Matching.

### Effect on OS between pediatric and adult patients before and after PSM

Before PSM, univariate Cox regression analyses showed that adult patients had worse OS (HR: 2.11; 95% CI: 1.68–2.65; *P* < 0.001), and multivariate Cox regression analyses indicated age to be an independent prognostic factor (HR, 1.97; 95% CI: 1.56–2.48; *P* < 0.001) (Table 2A). After PSM, adult patients also had poorer OS than pediatric patients (HR: 1.91; 95% CI: 1.47–2.47; *P* < 0.001), and age remained an independent prognostic factor (HR: 1.99; 95% CI: 1.53–2.58; *P* < 0.001) (Table 2B). We constructed KM curves to compare the OS of pediatric and adult ES patients. Both before and after PSM, the OS of the adult group was worse than that of the pediatric group [Figures 2C,D].

**Table 2A:** Univariate and multivariate Cox analyses of factors related to OS before PSM.

	Univariate Cox			Multivariate Cox		
	HR	95% CI	*P* value	HR	95% CI	*P* value
Age (years)						
<18	Reference			Reference		
≥18	2.11	1.68–2.65	<0.001	1.97	1.56–2.48	<0.001
Race						
White	Reference					
Black	1.74	0.99–3.03	0.053			
Others	1.05	0.70–1.59	0.809			
Sex						
Male	Reference					
Female	0.87	0.68–1.10	0.241			
T stage						
T1	Reference			Reference		
T2	1.56	1.23–1.98	<0.001	1.25	0.96–1.61	0.092
T3	3.72	2.51–5.51	<0.001	2.07	1.35–3.19	0.001
N stage						
N0	Reference			Reference		
N1	1.94	1.34–2.79	<0.001	1.59	1.09–2.32	0.017
M stage						
M0	Reference			Reference		
M1	3.13	2.49–3.94	<0.001	2.22	1.70–2.88	<0.001
Surgery						
Yes	Reference			Reference		
No	2.20	1.75–2.76	<0.001	1.34	1.04–1.72	0.025
Radiation						
Yes	Reference			Reference		
No/Unknown	0.59	0.47–0.74	<0.001	0.8	0.63–1.03	<0.088
Chemotherapy						** **
Yes	Reference			Reference		
No/Unknown	2.77	1.59–4.82	<0.001	3.07	1.72–5.49	<0.001
Primary site						
Extremity	Reference			Reference		
Axial	1.42	1.13–1.79	0.003	1.2	0.95–1.53	0.129

**Table 2B:** Univariate and multivariate Cox analyses of factors related to OS after PSM.

	Univariate Cox			Multivariate Cox		
	HR	95% CI	*P* value	HR	95% CI	*P* value
Age (years)						
<18	Reference			Reference		
≥18	1.91	1.47–2.47	<0.001	1.99	1.53–2.58	<0.001
Race						
White	Reference					
lack	1.30	0.61–2.77	0.490			
Others	1.13	0.71–1.80	0.613			
Sex						
Male	Reference					
Female	0.90	0.69–1.19	0.469			
T stage						
T1	Reference			Reference		
T2	1.63	1.24–2.13	<0.001	1.27	0.96–1.69	0.099
T3	4.13	2.71–6.30	<0.001	2.24	1.41–3.55	<0.001
N stage						
N0	Reference			Reference		
N1	1.87	1.25–2.79	0.002	1.61	1.06–2.44	0.024
M stage						
M0	Reference			Reference		
M1	3.00	2.33–3.87	<0.001	2.18	1.63–2.92	<0.001
Surgery						
Yes	Reference			Reference		
No	1.96	1.52–2.52	<0.001	1.30	0.98–1.73	0.066
Radiation						
Yes	Reference			Reference		
No/Unknown	0.64	0.50–0.83	0.001	0.89	0.67–1.16	0.378
Chemotherapy						
Yes	Reference			Reference		
No/Unknown	0.59	0.08–4.20	0.597	1.21	0.93–1.58	0.165
Primary site						
Extremity	Reference					
Axial	1.41	1.09–1.82	0.008			

OS, overall survival.

### Nomogram development and validation

**Figure 3:**
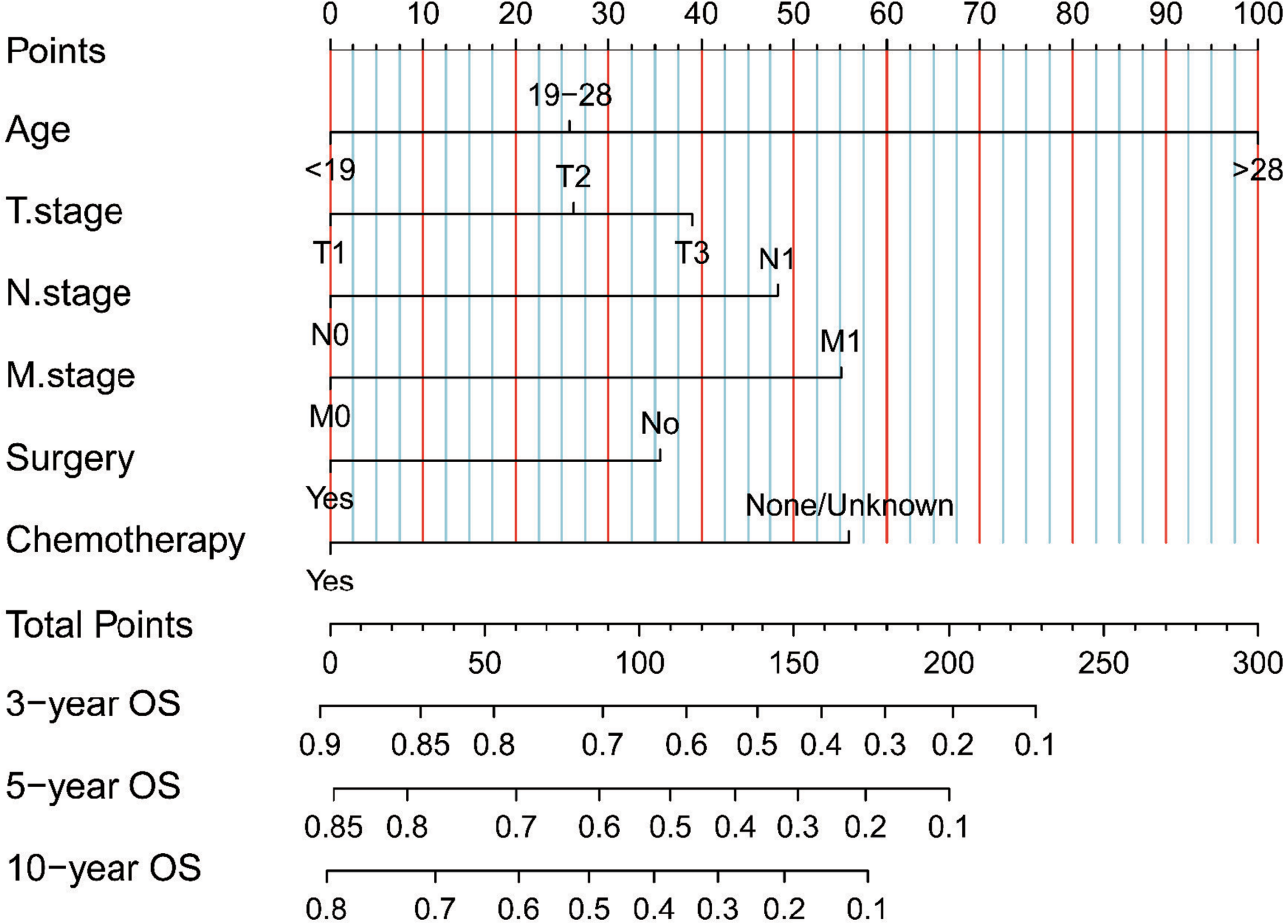
Our nomograms for predicting the 3-, 5-, and 10-year OS of adult patients with ES of bone. It consists of 6 variables (age, T stage, N stage, M stage, surgery, and chemotherapy).

Because the survival rate of adult patients was found to be poorer than that of pediatric patients, we sought to build a nomogram for these adult patients. Univariate and multivariate Cox regression analyses identified that age, T stage, N stage, M stage, surgery, and chemotherapy were independent prognostic factors in adult patients with ES of bone (Table 3). We created our nomogram based on these factors to predict 3-, 5-, and 10-year OS (Figure 3). Calculating the projected chance of survival at each time point was straightforward, as the entire score was added and plotted on the total point scale (11).

**Table 3:** Identification of independent prognostic factors in adult patients based on univariate and multivariate Cox analyses.

	Univariate Cox			Multivariate Cox		
	HR	95% CI	*P* value	HR	95% CI	*P* value
Age (years)						
<19	Reference			Reference		
19–28	1.32	0.72–2.43	0.371	1.41	0.76–2.61	0.273
>28	3.02	1.64–5.54	<0.001	3.79	2.02–7.12	<0.001
Race						
White	Reference					
Black	1.43	0.75–2.71	0.278			
Others	0.98	0.53–1.80	0.936			
Sex						
Male	Reference					
Female	0.87	0.63–1.20	0.395			
T stage						
T1	Reference			Reference		
T2	1.52	1.10–2.11	0.012	1.42	1.01–2.00	0.044
T3	3.59	2.16–5.96	<0.001	1.68	0.97–2.93	0.067
N stage						
N0	Reference			Reference		
N1	2.57	1.53–4.31	<0.001	1.90	1.10- 3.28	0.021
M stage						
M0	Reference			Reference		
M1	2.67	1.96–3.65	<0.001	2.09	1.44 –3.02	<0.001
Surgery						
Yes	Reference			Reference		
No	2.00	1.48–2.72	<0.001	1.61	1.14 –2.26	0.007
Radiation						
Yes	Reference			Reference		
No/Unknown	0.85	0.63–1.15	0.291	2.11	1.09–4.08	0.027
Chemotherapy						
Yes	Reference					
No/Unknown	2.02	1.06–3.82	0.032			
Primary site						
Extremity	Reference					
Axial	1.30	0.95–1.77	0.098			

**Figure 4:**
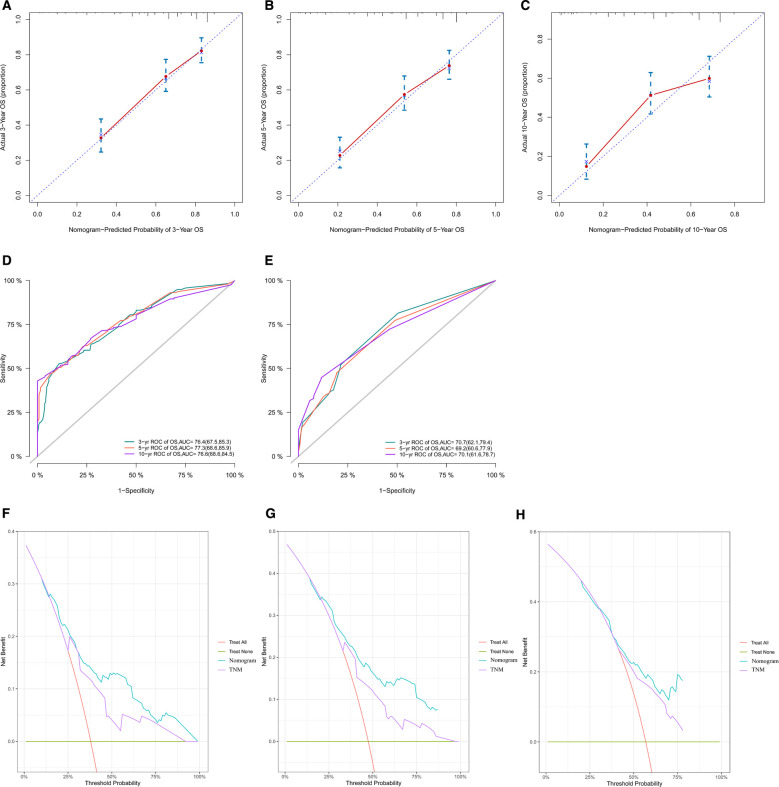
Calibration curves of our nomogram in all cohorts for 3-year (**A**), 5-year (**B**), and 10-year (**C**) OS. This shows that the OS predicted by our nomogram is highly consistent with the actual survival rate and has a high level of calibration. ROC curves of our nomogram (**D**) and the TNM nomogram (**E**) for 3-year, 5-year and 10-year OS. The AUC values of our nomogram for predicting 3-, 5- and 10-year OS were 76.4, 77.3, and 76.6, respectively; the AUC values of the TNM nomogram for predicting 3-, 5- and 10-year OS were 70.7, 69.2, and 70.1, respectively. According to the results of ROC curves and AUC values, it can be concluded that our nomogram has better predictive ability than the TNM nomogram. Comparison of our nomogram with the TNM nomogram by DCA for OS at 3 years (**F**), 5 years (**G**) and 10 years (**H**). Comparison of DCA curves of the two nomograms shows that our nomogram has greater clinical benefits. OS, overall survival; ROC, receiver operating characteristic; AUC, areas under the curve; DCA, decision curve analysis.

We constructed calibration curves for 3-year, 5-year, and 10-year OS, which showed good consistency between the predicted and observed probabilities of OS (Figures 4A–C). Indeed, our nomogram was able to produce precise predictions in a variety of situations, with high AUC values [3-year ROC of OS, AUC = 76.4 (67.5, 85.3); 5-year, AUC = 77.3 (68.6, 85.9); 10-year, AUC = 76.6 (68.6, 84.5)] [Figure 4(D)], and performed better than the TNM nomogram [3-year ROC of OS, AUC = 70.7 (62.1, 79.4); 5-year ROC of OS, AUC = 69.2 (60.6, 77.9); 10-year OS, AUC = 70.1 (61.6, 78.7)] [Figure 4(E)]. DCA results for our nomogram were compared with those of the TNM nomogram, and our nomogram showed a more considerable net benefit regarding 3-, 5- and 10-year OS (Figures 4F–H), indicating that our model was more accurate at predicting the OS of adult patients with ES of bone.

## Discussion

ES is the second most frequent bone tumor in children and teens (12, 13). Many articles have discussed independent factors of ES, but few articles have presented comparisons of OS between pediatric and adult patients with ES of bone and constructed nomograms for adults. Based on our study, age, TNM stage, surgery, and chemotherapy are independent prognostic factors in adult patients with ES of bone.

Age is a significant predictor of survival in patients with ES. In previous studies, younger patients had more favorable survival, and older age was linked to poorer clinical outcomes (14, 15). However, the exact mechanism remains unknown. It is possible that some explanations have not been considered, such as seeking care, slow expression, the quality of care and less aggressive treatment; additionally, some explanations of why older patients have worse OS than young people may have been overlooked (16). It has also been thought that older patients with ES may have multiple comorbidities, including diabetes, hypertension, and secondary cancers, which may lead to worse prognosis (17, 18). In many cases, children may have access to high medical care relative to adult patients, regardless of their socioeconomic status (16). Abha A Gupta et al. considered that factors not assessed in most similar studies (e.g., the duration of topical treatments) may be prognostic (19). In our study, age was an independent prognostic factor for adult ES of bone.

Use of radiation therapy in patients with ES has been a point of debate. Many studies, such as ours, have shown no significant relationship between radiotherapy use and prognosis in ES patients (20–24). ES is considered radiation sensitive, whereas radiation therapy is controversial, and the proportion of patients receiving radiation alone has been steadily decreasing. This may be attributable to advances in orthopedic surgery and chemotherapy, as well as late effects of radiotherapy in children, such as secondary malignancies and growth disorders (25). Our study found that patients who underwent surgery had a better prognosis than those who did not, and many studies have reported the same findings (16, 22, 23). With the advances in diagnosis and treatment technology, more precise individualized treatment strategies can effectively improve the prognosis of patients with ES (26). A recent systematic review by J. Werier et al. found that, regarding the optimal local treatment strategy for localized ES, surgery alone (if negative margins can be achieved) is a reasonable treatment option and should be decided on the basis of patient clinical characteristics, side effects, and patient preference for optimal local treatment (27).

In our study, chemotherapy was an independent prognostic factor, which was the same as in previous research (22). However, chemotherapy strategies are not necessarily the same in each study, and chemotherapy drugs, doses, and combinations vary from country to country. Accordingly, heterogeneity in the treatment of patients exists in different studies. Although all patients received neoadjuvant therapy, followed by local treatment of the primary tumor and adjuvant chemotherapy, the existence of heterogeneity between treatments will increase risk of bias, thus affecting the quality of research results (27). Based on the previous studies above, further large-scale prospective clinical trials are needed to explore the prognosis related to various treatment strategies.

T stage is one of the prognostic factors in our nomogram, and some previous studies have shown that a larger tumor volume is related to poor prognosis (28–30). M stage is also a prognostic factor, representing whether metastasis will affect the patient's OS. This result is the same as in previous work (17, 22, 31). Nevertheless, the prognosis of patients with metastatic or recurrent ES remains poor, and 20%–25% of patients develop metastases. The presence of metastatic disease is the most important adverse prognostic factor in ES, with a survival rate after metastasis of 30%. The most common sites of metastatic disease are the bone, bone marrow, and lung, but other sites are extremely rare (32–35). Among patients with metastases, those with lung metastases tend to have better survival than those with bone or combined lung and bone metastases (30).

Our nomogram including independent prognostic factors (age, TNM stage, surgery, and chemotherapy) may be effective in predicting prognosis. However, the present personalized nomogram of ES is not highly efficient. Our research seeks to create a realistic survival prediction model for customized prediction regarding the OS of adult patients with ES of bone. Our model is predictive, with high AUC values. Calibration curves revealed good correlation between predicted survival and actual survival, ensuring our nomogram's repeatability and reliability. Additionally, we were able to more precisely analyze and predict OS when using our nomogram. Wang J built a model for predicting ES mortality using least absolute shrinkage and selection operator (LASSO) analysis and multivariate logistic analysis with five factors (age, tumor size, primary site, tumor extension, and other site metastasis). However, that nomogram does not include treatment approach (28). Chen L created a nomogram to predict the OS of pelvic ES with four factors (age, race, tumor stage, and surgery) (9).

Our study developed the first nomograms capable of predicting OS in adult patients with ES of bone. Using the scoring system, both clinicians and patients may understand individual survival expectations. For example, a 55-year-old patient was diagnosed with ES of bone at stage T1, stage N0, and stage M0 and was treated with surgery and chemotherapy. This patient received 140 points according to our nomogram. Therefore, the estimated 3-, 5- and 10-year OS probabilities would be approximately 60%–70%, 50%–60%, and 40%–50%, respectively. This prognostic model may serve as a tool for clinical research and decision-making, including patient classification and treatment recommendations. In general, with the advancement of complete therapies for ES, new therapeutic techniques are needed to enhance survival in patients.

There are some limitations in our study. First, the SEER database contains retrospective cohort data, which may have included selection bias and unavoidably involved missing data. Second, as the primary endpoints, we only focused on the three-, five-, and ten-year OS rates, and we only included data from 2004 to 2015 in our analysis. Additionally, SEER data do not include some details, such as adjuvant or neoadjuvant treatment, proportion of subtypes, local recurrence, detailed radiotherapy regimen, surgical margin status, and postoperative complications. Finally, the prognostic nomogram requires external data for verification and support. However, due to our study's lack of external data, only internal verification can be done. A multicenter analysis of a large population should be used to verify the prognostic nomogram in our study. Although it has certain limitations, the nomogram was built using a large population, ultimately yielding a therapeutically effective tool for predicting the OS of adult patients with ES of bone.

## Conclusions

In conclusion, we identified patients with ES of bone with poor OS and constructed a nomogram for these patients. The nomogram showed relatively good performance and may be considered a practical tool to predict the individual prognosis of adult patients with ES of bone.

## Data Availability

The original contributions presented in the study are included in the article/**Supplementary Materials**, further inquiries can be directed to the corresponding author/s.
