# Supplementary material for: Overall survival comparison between pediatric and adult Ewing sarcoma of bone and adult nomogram construction: a large population-based analysis
Source: Front Pediatr. 2023 May 23;11:1103565. doi: 10.3389/fped.2023.1103565 (PMC10242502; doi:10.3389/fped.2023.1103565)
Supplement: Supplementary file 1 [file Datasheet1.pdf]

Year of diagnosis	Race recode (W, B, AI, API)	Diagnostic Confirmation
2002	White	Positive exfoliative cytology, no positive histology
2004	White	Positive histology
2001	White	Positive histology
2001	Black	Positive histology
2000	White	Positive exfoliative cytology, no positive histology
2000	Asian or Pacific Islander	Positive histology
2001	White	Positive histology
2002	Asian or Pacific Islander	Positive histology
2001	Asian or Pacific Islander	Positive histology
2002	White	Positive histology
2002	White	Positive histology
2002	White	Positive histology
2002	White	Positive histology
2004	Asian or Pacific Islander	Positive histology
2004	White	Positive histology
2003	American Indian/Alaska Nati	Positive histology
2004	White	Positive histology
2004	White	Positive histology
2004	White	Positive histology
2008	White	Positive histology
2004	White	Positive histology
2004	White	Positive histology
2005	White	Positive histology
2005	Asian or Pacific Islander	Positive histology
2005	White	Positive histology
2006	White	Positive histology
2006	White	Positive histology
2006	Asian or Pacific Islander	Positive histology
2006	White	Positive histology
2006	White	Positive histology
2007	White	Positive histology
2008	White	Positive histology
2008	White	Positive histology
2009	Asian or Pacific Islander	Positive histology
2008	White	Positive histology
2008	White	Positive histology
2009	Asian or Pacific Islander	Positive histology
2009	Asian or Pacific Islander	Positive histology
2009	White	Positive histology
2009	White	Positive histology
2009	Asian or Pacific Islander	Positive histology
2010	White	Positive histology
2010	White	Positive histology
2009	White	Positive histology
2010	White	Positive histology
2011	White	Positive histology
2011	White	Positive histology
2011	White	Positive histology
2011	White	Positive histology
2011	Asian or Pacific Islander	Positive histology
2011	White	Positive histology
2011	White	Positive histology
2011	Asian or Pacific Islander	Positive histology
2019	White	Positive histology
2013	White	Positive histology
2013	White	Positive histology
2013	Asian or Pacific Islander	Positive histology

2013	White	Positive histology
2013	Asian or Pacific Islander	Positive histology
2014	Asian or Pacific Islander	Positive histology
2014	White	Positive histology
2012	White	Positive histology
2015	White	Positive histology
2015	White	Positive histology
2016	Asian or Pacific Islander	Positive histology
2016	White	Positive histology
2015	White	Positive histology
2017	White	Positive histology
2017	Asian or Pacific Islander	Positive histology
2019	White	Positive histology
2017	White	Positive exfoliative cytology, no positive histology
2017	White	Positive histology
2017	White	Positive histology
2017	White	Positive histology
2018	White	Positive histology
2018	White	Positive histology
2018	White	Positive histology
2019	Asian or Pacific Islander	Positive histology
2019	White	Positive histology
2018	White	Positive histology
2019	White	Positive histology
2019	White	Positive histology
2019	White	Positive histology
2000	White	Positive histology
2001	White	Positive histology
2001	White	Positive histology
2017	White	Positive histology
2001	White	Positive histology
2001	White	Positive histology
2001	White	Positive histology
2001	White	Positive histology
2001	White	Positive histology
2002	White	Positive histology
2003	White	Positive histology
2003	White	Positive histology
2004	White	Positive histology
2005	Black	Positive histology
2005	White	Positive histology
2005	White	Positive histology
2006	White	Positive histology
2006	White	Positive histology
2006	White	Positive exfoliative cytology, no positive histology
2006	White	Positive histology
2006	White	Positive histology
2007	White	Positive histology
2007	White	Positive histology
2001	American Indian/Alaska Nati	Positive histology
2007	White	Positive histology
2007	White	Positive histology
2008	White	Positive histology
2008	White	Positive histology
2008	White	Positive histology
2008	White	Positive histology
2009	White	Positive histology
2009	White	Positive histology

2009	White	Positive histology
2009	White	Positive histology
2009	White	Positive histology
2009	White	Positive histology
2010	White	Positive histology
2019	White	Positive histology
2010	White	Positive histology
2010	White	Positive histology
2010	White	Positive histology
2010	White	Positive histology
2018	White	Positive histology
2011	White	Positive histology
2011	White	Positive histology
2011	White	Positive histology
2011	White	Positive histology
2011	Asian or Pacific Islander	Positive histology
2011	White	Positive histology
2012	White	Positive histology
2012	White	Positive histology
2012	Asian or Pacific Islander	Positive histology
2013	White	Positive histology
2013	White	Positive histology
2013	White	Positive histology
2013	White	Positive histology
2013	White	Positive histology
2014	White	Positive histology
2014	White	Positive histology
2014	White	Positive histology
2014	Unknown	Positive histology
2014	Black	Positive histology
2014	White	Positive histology
2015	White	Positive histology
2015	White	Positive histology
2015	White	Positive histology
2015	White	Positive histology
2015	White	Positive histology
2015	White	Positive histology
2016	White	Positive histology
2016	White	Positive histology
2016	White	Positive histology
2016	White	Positive histology
2016	White	Positive histology
2016	White	Positive histology
2017	White	Positive histology
2012	White	Positive histology
2018	White	Positive histology
2018	White	Positive histology
2018	White	Positive histology
2019	White	Positive histology
2019	White	Positive histology
2019	White	Positive histology
2019	Unknown	Positive histology
2019	White	Positive histology
2000	Asian or Pacific Islander	Positive histology
2000	White	Positive exfoliative cytology, no positive histology
2002	Asian or Pacific Islander	Positive histology
2003	White	Positive histology
2004	Asian or Pacific Islander	Positive histology

2007	Asian or Pacific Islander	Positive histology
2007	White	Positive histology
2008	Asian or Pacific Islander	Positive histology
2009	Asian or Pacific Islander	Positive histology
2010	Asian or Pacific Islander	Positive histology
2011	Asian or Pacific Islander	Positive histology
2013	Asian or Pacific Islander	Positive histology
2012	White	Positive histology
2013	Asian or Pacific Islander	Positive histology
2015	Asian or Pacific Islander	Positive histology
2015	White	Positive histology
2016	Asian or Pacific Islander	Positive histology
2016	Asian or Pacific Islander	Positive histology
2019	Asian or Pacific Islander	Positive histology
2017	Asian or Pacific Islander	Positive histology
2018	White	Positive histology
2000	White	Positive histology
2000	White	Positive histology
2000	White	Positive histology
2000	White	Positive histology
2000	White	Positive histology
2001	White	Positive exfoliative cytology, no positive histology
2001	White	Positive histology
2001	White	Positive histology
2001	White	Positive histology
2001	White	Positive histology
2001	White	Positive histology
2001	White	Positive histology
2002	White	Positive histology
2002	White	Positive histology
2002	White	Positive histology
2001	White	Positive histology
2002	White	Positive histology
2002	White	Positive histology
2003	White	Positive histology
2003	White	Positive histology
2003	White	Positive histology
2012	White	Positive histology
2003	White	Positive histology
2004	White	Positive histology
2004	White	Positive histology
2005	White	Positive histology
2005	White	Positive histology
2005	White	Positive histology
2005	White	Positive histology
2005	White	Positive histology
2006	White	Positive exfoliative cytology, no positive histology
2006	White	Positive histology
2005	White	Positive histology
2010	White	Positive exfoliative cytology, no positive histology
2005	White	Positive histology
2006	White	Positive histology
2006	White	Positive histology
2006	White	Positive histology
2007	White	Positive histology
2006	White	Positive histology
2007	White	Positive histology
2007	White	Positive exfoliative cytology, no positive histology

2008	White	Positive histology
2008	White	Positive histology
2008	White	Positive histology
2008	White	Positive exfoliative cytology, no positive histology
2009	White	Positive histology
2009	White	Positive histology
2009	White	Positive histology
2009	White	Positive histology
2009	White	Positive histology
2010	White	Positive histology
2010	White	Positive histology
2010	White	Positive histology
2010	White	Positive histology
2011	White	Positive histology
2011	White	Positive histology
2011	White	Positive histology
2010	White	Positive histology
2011	White	Positive histology
2011	White	Positive histology
2011	White	Positive histology
2012	White	Positive histology
2012	White	Positive histology
2013	White	Positive histology
2013	White	Positive histology
2013	White	Positive histology
2014	White	Positive histology
2014	White	Positive histology
2014	White	Positive histology
2017	White	Positive histology
2014	White	Positive histology
2013	White	Positive histology
2014	White	Positive histology
2015	White	Positive histology
2017	White	Positive histology
2017	White	Positive histology
2017	Unknown	Positive histology
2017	White	Positive histology
2017	White	Positive histology
2017	White	Positive histology
2017	White	Positive histology
2018	White	Positive histology
2017	White	Positive histology
2018	White	Positive histology
2018	White	Positive histology
2018	White	Positive histology
2019	White	Positive histology
2019	White	Positive histology
2019	White	Positive histology
2019	White	Positive histology
2017	White	Positive histology
2000	White	Positive histology
2000	American Indian/Alaska Nati	Positive histology
2001	White	Positive histology
2001	White	Positive histology
2002	American Indian/Alaska Nati	Positive histology
2002	White	Positive histology
2004	White	Positive histology
2003	White	Positive histology

2003	White	Positive histology
2003	White	Positive histology
2004	Asian or Pacific Islander	Positive histology
2004	American Indian/Alaska Nati	Positive histology
2005	White	Positive histology
2006	White	Positive histology
2007	White	Positive histology
2008	White	Positive histology
2007	White	Positive histology
2008	White	Positive histology
2008	White	Positive histology
2009	White	Positive histology
2009	White	Positive histology
2010	White	Radiography without microscopic confirm
2010	White	Positive histology
2011	White	Positive histology
2011	American Indian/Alaska Nati	Positive histology
2012	White	Positive histology
2012	White	Positive histology
2011	White	Positive histology
2013	White	Positive histology
2013	White	Positive histology
2013	White	Positive histology
2011	White	Positive histology
2014	White	Positive histology
2015	White	Positive histology
2015	American Indian/Alaska Nati	Positive histology
2016	White	Positive histology
2016	White	Positive histology
2016	White	Positive histology
2016	White	Positive histology
2016	White	Positive histology
2017	White	Positive histology
2016	White	Positive histology
2018	White	Positive histology
2018	White	Unknown
2019	White	Positive histology
2006	White	Positive histology
2000	White	Positive histology
2000	White	Positive histology
2000	White	Positive histology
2000	White	Positive histology
2000	White	Positive histology
2001	White	Positive histology
2001	White	Positive histology
2000	White	Positive histology
2001	White	Positive histology
2001	White	Positive histology
2001	White	Positive histology
2001	White	Positive histology
2002	White	Positive histology
2003	White	Positive histology
2003	White	Positive histology
2003	White	Positive histology
2004	White	Positive histology
2004	White	Positive histology
2008	White	Unknown
2004	White	Positive histology

2005	Asian or Pacific Islander	Positive histology
2005	White	Positive histology
2005	White	Positive histology
2005	White	Positive histology
2005	White	Positive histology
2006	White	Positive histology
2006	White	Positive histology
2005	White	Positive histology
2006	White	Positive histology
2006	American Indian/Alaska Nati	Positive histology
2006	White	Positive histology
2006	White	Positive histology
2006	White	Positive histology
2007	White	Positive histology
2007	White	Positive histology
2007	White	Positive histology
2007	Asian or Pacific Islander	Positive histology
2008	White	Positive histology
2008	White	Positive histology
2008	White	Positive histology
2008	White	Positive histology
2008	White	Positive histology
2009	White	Positive histology
2008	White	Positive histology
2008	White	Positive histology
2009	White	Positive histology
2009	White	Positive histology
2009	White	Positive histology
2009	White	Positive histology
2009	White	Positive histology
2009	White	Positive histology
2009	White	Positive histology
2010	White	Positive histology
2010	White	Positive histology
2010	White	Positive histology
2010	White	Positive histology
2010	White	Positive histology
2011	Asian or Pacific Islander	Positive histology
2011	White	Positive histology
2011	White	Positive histology
2011	White	Positive histology
2011	White	Positive histology
2012	White	Positive histology
2012	White	Positive histology
2012	White	Positive histology
2012	White	Positive histology
2012	Asian or Pacific Islander	Positive histology
2012	White	Positive histology
2013	White	Positive histology
2013	White	Positive histology
2013	White	Positive histology
2013	White	Positive histology
2013	Black	Positive histology
2013	American Indian/Alaska Nati	Positive histology
2014	White	Positive histology
2014	White	Positive histology
2014	White	Positive histology
2014	White	Positive histology

2014	White	Positive histology
2014	White	Positive histology
2014	White	Positive histology
2015	White	Positive histology
2015	White	Positive histology
2015	White	Positive histology
2015	White	Positive histology
2015	White	Positive histology
2016	White	Positive histology
2016	White	Positive histology
2016	Asian or Pacific Islander	Positive histology
2016	White	Positive histology
2016	White	Positive histology
2017	Black	Positive histology
2017	Asian or Pacific Islander	Positive histology
2017	White	Positive histology
2018	White	Positive histology
2018	Asian or Pacific Islander	Positive histology
2019	Asian or Pacific Islander	Positive histology
2019	White	Positive histology
2019	White	Positive histology
2019	Black	Positive histology
2019	White	Positive histology
2019	White	Positive histology
2019	White	Positive histology
2019	White	Positive histology
2000	White	Positive histology
2000	White	Positive histology
2001	White	Positive histology
2001	White	Positive histology
2001	White	Positive histology
2001	White	Positive histology
2001	White	Positive histology
2002	White	Positive histology
2003	White	Positive histology
2003	White	Positive histology
2003	White	Positive histology
2003	White	Positive histology
2004	White	Positive histology
2005	White	Positive histology
2005	White	Positive histology
2005	White	Positive histology
2006	White	Positive histology
2006	White	Positive histology
2006	White	Positive histology
2006	White	Positive histology
2006	White	Positive histology
2014	White	Positive histology
2006	White	Positive histology
2004	White	Positive histology
2007	White	Positive histology
2007	White	Positive histology
2008	White	Positive histology
2008	American Indian/Alaska Nati	Positive histology
2008	White	Positive histology
2008	White	Positive histology
2008	White	Positive histology
2008	White	Positive histology

2009	White	Positive histology
2009	White	Positive histology
2009	White	Positive histology
2009	White	Positive histology
2009	White	Positive histology
2010	White	Positive histology
2010	White	Positive histology
2010	White	Positive histology
2010	White	Positive histology
2011	White	Positive histology
2011	White	Positive histology
2010	White	Positive histology
2007	White	Positive histology
2008	White	Positive histology
2008	White	Positive histology
2008	White	Positive histology
2011	White	Positive histology
2014	White	Positive histology
2012	White	Positive histology
2012	White	Positive histology
2012	White	Positive histology
2012	White	Positive histology
2013	White	Positive exfoliative cytology, no positive histology
2012	White	Positive histology
2012	White	Positive histology
2013	White	Positive histology
2012	White	Positive histology
2012	White	Positive histology
2012	White	Positive histology
2013	White	Positive histology
2013	White	Positive histology
2013	White	Positive histology
2013	White	Positive histology
2014	White	Positive histology
2014	White	Positive histology
2014	White	Positive histology
2014	White	Positive histology
2014	White	Positive histology
2015	White	Positive histology
2015	White	Positive histology
2015	White	Positive histology
2015	White	Positive histology
2015	White	Positive histology
2015	White	Positive histology
2016	White	Positive histology
2016	White	Positive histology
2016	White	Positive histology
2016	White	Positive histology
2016	White	Positive histology
2016	White	Positive histology
2016	American Indian/Alaska Nati	Positive histology
2016	White	Positive histology
2016	White	Positive histology
2017	White	Positive histology
2017	American Indian/Alaska Nati	Positive histology
2017	Black	Positive histology
2017	White	Positive histology
2018	White	Positive histology

2018	Asian or Pacific Islander	Positive histology
2018	White	Positive histology
2018	White	Positive histology
2018	White	Positive histology
2018	White	Positive histology
2018	White	Positive histology
2019	White	Positive histology
2019	White	Positive histology
2019	White	Positive histology
2018	White	Positive histology
2019	White	Positive histology
2019	White	Positive histology
2019	White	Positive histology
2000	White	Positive histology
2000	White	Positive histology
2000	White	Positive histology
2000	White	Positive histology
2001	White	Positive histology
2001	White	Positive histology
2001	White	Positive histology
2002	White	Positive histology
2002	White	Positive exfoliative cytology, no positive histology
2001	White	Positive histology
2002	White	Positive histology
2002	White	Positive histology
2002	Black	Positive histology
2002	White	Positive histology
2003	White	Positive histology
2003	White	Positive histology
2003	White	Positive histology
2004	White	Positive histology
2005	White	Positive histology
2005	White	Positive histology
2005	White	Positive histology
2006	Black	Positive histology
2006	White	Positive histology
2005	Black	Positive histology
2006	White	Positive histology
2006	White	Positive histology
2006	White	Positive histology
2008	Black	Positive histology
2008	White	Positive histology
2009	Black	Positive histology
2010	Black	Positive histology
2011	White	Positive histology
2009	White	Positive histology
2011	White	Positive histology
2011	White	Positive histology
2011	White	Positive histology
2012	White	Positive histology
2013	Black	Positive histology
2013	White	Positive histology
2013	White	Positive histology
2014	White	Positive histology
2014	Asian or Pacific Islander	Positive histology
2015	White	Positive histology
2014	White	Positive histology
2015	Asian or Pacific Islander	Positive histology

2016	White	Positive histology
2016	Black	Positive exfoliative cytology, no positive histology
2015	Asian or Pacific Islander	Positive histology
2015	White	Positive histology
2016	White	Positive histology
2016	Asian or Pacific Islander	Positive histology
2015	White	Positive histology
2017	White	Positive histology
2016	Black	Positive histology
2017	White	Positive histology
2018	Black	Positive histology
2018	White	Positive histology
2018	White	Positive histology
2018	Unknown	Positive histology
2019	White	Positive histology
2019	White	Positive histology
2019	White	Positive histology
2019	White	Positive histology
2019	White	Positive histology
2005	American Indian/Alaska Nati	Positive histology
2005	American Indian/Alaska Nati	Positive histology
2011	American Indian/Alaska Nati	Positive histology
2012	American Indian/Alaska Nati	Positive histology
2012	American Indian/Alaska Nati	Positive histology
2013	American Indian/Alaska Nati	Positive histology
2014	American Indian/Alaska Nati	Positive histology
2016	American Indian/Alaska Nati	Positive exfoliative cytology, no positive histology
2000	Asian or Pacific Islander	Positive histology
2000	White	Positive histology
2002	White	Positive histology
2003	White	Positive histology
2001	White	Positive histology
2002	White	Positive histology
2004	White	Positive histology
2003	White	Positive histology
2005	White	Positive histology
2005	White	Positive histology
2006	White	Positive histology
2006	White	Positive histology
2006	Black	Positive histology
2006	White	Positive histology
2006	White	Positive histology
2016	White	Positive histology
2007	White	Positive histology
2007	White	Positive histology
2007	White	Positive histology
2007	White	Positive histology
2008	White	Positive histology
2008	White	Positive histology
2008	White	Positive histology
2008	White	Positive histology
2008	White	Positive histology
2008	White	Positive histology
2009	Black	Positive histology
2009	White	Positive histology
2009	White	Positive histology
2009	White	Positive histology
2010	Asian or Pacific Islander	Positive histology

2010	White	Positive histology
2010	White	Positive histology
2010	White	Positive histology
2009	Black	Positive histology
2011	White	Positive histology
2011	White	Positive histology
2010	White	Positive histology
2011	White	Positive histology
2013	Asian or Pacific Islander	Positive histology
2013	White	Positive histology
2013	White	Positive histology
2014	White	Positive histology
2015	Asian or Pacific Islander	Positive histology
2015	White	Positive histology
2015	White	Positive histology
2016	White	Positive histology
2016	White	Positive histology
2016	White	Positive histology
2016	Asian or Pacific Islander	Positive histology
2017	White	Positive histology
2017	White	Positive histology
2018	White	Positive histology
2018	White	Positive histology
2019	Asian or Pacific Islander	Positive histology
2019	White	Positive histology
2019	White	Positive histology
2019	White	Positive histology
2019	White	Positive histology
2002	White	Positive histology
2007	White	Positive histology
2002	White	Positive histology
2001	Asian or Pacific Islander	Positive histology
2001	White	Positive histology
2001	White	Positive histology
2001	White	Positive histology
2000	White	Positive histology
2000	White	Positive histology
2001	White	Positive histology
2000	White	Positive histology
2001	White	Positive histology
2002	Asian or Pacific Islander	Positive histology
2000	White	Positive histology
2001	White	Positive histology
2000	White	Positive histology
2001	White	Positive histology
2000	White	Positive histology
2011	White	Positive histology
2001	White	Positive histology
2002	White	Positive histology
2000	White	Positive histology
2002	White	Positive histology
2001	White	Positive exfoliative cytology, no positive histology
2002	White	Positive histology
2002	White	Positive histology
2003	White	Positive histology
2002	White	Positive histology
2002	White	Positive histology
2003	White	Positive histology

2002	White	Positive histology
2002	White	Positive histology
2003	White	Positive histology
2001	Black	Positive histology
2003	White	Positive histology
2003	White	Positive histology
2004	White	Positive histology
2004	White	Positive histology
2004	White	Positive histology
2004	White	Positive histology
2004	White	Positive histology
2004	White	Positive histology
2004	White	Positive histology
2004	White	Positive histology
2004	White	Positive histology
2004	White	Positive histology
2004	White	Positive histology
2004	White	Positive histology
2003	White	Positive histology
2013	White	Positive histology
2005	White	Positive histology
2005	White	Positive histology
2005	White	Positive histology
2005	White	Positive exfoliative cytology, no positive histology
2005	White	Positive histology
2005	White	Positive histology
2005	White	Positive histology
2005	White	Positive histology
2006	White	Positive histology
2006	White	Positive histology
2006	White	Positive histology
2006	White	Positive histology
2006	White	Positive histology
2006	White	Positive histology
2006	White	Positive histology
2006	White	Positive histology
2015	White	Positive histology
2006	White	Positive histology
2006	White	Positive histology
2006	White	Positive histology
2006	White	Positive histology
2006	White	Positive histology
2007	Asian or Pacific Islander	Positive histology
2007	Black	Positive histology
2007	White	Positive histology
2007	White	Positive histology
2007	White	Positive histology
2007	White	Positive histology
2007	White	Positive histology
2007	White	Positive histology
2007	White	Positive histology
2007	White	Positive histology
2007	White	Positive histology
2007	White	Positive histology
2008	White	Positive histology
2008	White	Positive histology
2008	White	Positive histology
2004	White	Unknown

2008	White	Positive histology
2008	White	Positive histology
2008	White	Positive histology
2008	White	Positive histology
2008	White	Positive histology
2008	White	Positive histology
2008	White	Positive histology
2008	White	Positive histology
2007	White	Unknown
2009	White	Positive histology
2001	White	Positive histology
2009	White	Positive histology
2009	White	Positive histology
2009	White	Positive histology
2009	Asian or Pacific Islander	Positive histology
2009	White	Positive histology
2009	White	Positive histology
2010	White	Positive histology
2010	White	Positive histology
2010	White	Positive histology
2010	White	Positive histology
2010	White	Positive histology
2010	White	Positive histology
2010	White	Positive histology
2010	White	Positive histology
2017	White	Positive histology
2011	White	Positive histology
2011	White	Positive histology
2010	White	Positive histology
2011	Black	Positive histology
2011	White	Positive histology
2011	White	Positive histology
2011	White	Positive histology
2011	White	Positive histology
2011	White	Positive histology
2011	White	Positive histology
2011	White	Positive histology
2011	White	Positive histology
2011	White	Positive histology
2012	White	Positive histology
2012	White	Positive histology
2015	White	Positive histology
2006	White	Positive histology
2013	White	Positive histology
2013	White	Positive histology
2013	White	Positive histology
2013	White	Positive histology
2006	White	Positive histology
2013	White	Positive histology
2013	White	Positive histology
2013	White	Positive histology
2019	White	Positive histology
2013	White	Positive histology
2014	White	Positive histology
2013	White	Positive histology
2013	White	Positive histology
2014	White	Positive histology
2014	Black	Positive histology

2014	White	Positive histology
2014	White	Positive histology
2014	White	Positive histology
2014	Asian or Pacific Islander	Positive histology
2014	Asian or Pacific Islander	Positive histology
2013	White	Unknown
2014	White	Positive histology
2015	White	Positive histology
2015	White	Positive histology
2015	White	Positive histology
2015	White	Positive histology
2015	White	Positive histology
2015	White	Positive histology
2015	White	Positive histology
2015	Asian or Pacific Islander	Positive histology
2015	White	Positive histology
2015	White	Positive histology
2015	Asian or Pacific Islander	Positive histology
2014	White	Unknown
2014	White	Positive histology
2016	White	Positive histology
2016	White	Positive histology
2016	White	Positive histology
2016	White	Positive histology
2016	White	Positive histology
2016	White	Positive histology
2016	White	Positive histology
2016	White	Positive histology
2016	White	Positive histology
2016	White	Positive histology
2016	Black	Positive histology
2017	White	Positive histology
2017	White	Positive histology
2017	White	Positive histology
2017	White	Positive histology
2017	White	Positive histology
2017	White	Positive histology
2017	American Indian/Alaska Nati	Positive histology
2017	White	Positive histology
2017	White	Positive histology
2017	White	Positive histology
2018	White	Positive histology
2018	Black	Positive histology
2018	Black	Positive histology
2018	White	Positive histology
2018	White	Positive histology
2018	White	Positive histology
2018	White	Positive histology
2018	White	Positive histology
2018	Black	Positive histology
2018	White	Positive histology
2018	White	Positive histology
2019	White	Positive histology
2019	White	Positive histology
2019	White	Positive histology
2019	White	Positive histology
2019	White	Positive histology
2019	Asian or Pacific Islander	Positive histology

2019	White	Positive histology
2019	Black	Positive histology
2019	White	Unknown
2002	White	Positive histology
2014	White	Positive histology
2015	White	Positive histology
2018	White	Positive histology
2000	White	Positive histology
2000	White	Positive histology
2001	White	Positive histology
2001	White	Positive histology
2000	White	Positive histology
2000	Asian or Pacific Islander	Positive histology
2000	White	Positive histology
2000	White	Positive histology
2000	White	Positive histology
2001	White	Positive histology
2000	White	Positive histology
2001	White	Positive exfoliative cytology, no positive histology
2007	White	Positive histology
2000	White	Positive histology
2000	White	Positive histology
2001	White	Positive histology
2001	White	Positive histology
2002	White	Positive histology
2001	White	Positive histology
2000	White	Positive histology
2000	White	Positive histology
2010	White	Positive histology
2000	American Indian/Alaska Nati	Positive histology
2000	White	Positive exfoliative cytology, no positive histology
2000	White	Positive histology
2001	White	Positive histology
2001	White	Positive histology
2001	White	Positive histology
2001	White	Positive histology
2002	White	Positive histology
2002	White	Positive histology
2013	White	Positive histology
2002	White	Positive histology
2001	Asian or Pacific Islander	Positive histology
2002	White	Positive histology
2010	White	Positive histology
2000	White	Positive exfoliative cytology, no positive histology
2000	White	Positive histology
2000	White	Positive histology
2001	White	Positive histology
2001	White	Positive histology
2011	White	Positive histology
2000	White	Positive histology
2001	White	Positive histology
2000	White	Positive histology
2000	White	Positive histology
2000	White	Positive histology
2000	White	Positive histology
2000	White	Positive histology
2000	White	Positive histology
2001	White	Positive exfoliative cytology, no positive histology

2001	White	Positive histology
2001	White	Positive histology
2001	White	Positive histology
2002	White	Positive histology
2001	White	Positive histology
2002	White	Positive histology
2001	White	Positive histology
2002	White	Positive histology
2001	Asian or Pacific Islander	Positive histology
2002	White	Positive histology
2002	White	Positive histology
2002	White	Positive histology
2002	White	Positive exfoliative cytology, no positive histology
2002	White	Positive histology
2002	White	Positive histology
2002	White	Positive histology
2002	White	Positive histology
2000	White	Positive histology
2002	White	Positive histology
2002	White	Positive histology
2003	White	Positive histology
2003	White	Positive histology
2003	White	Positive histology
2002	White	Positive histology
2012	White	Positive histology
2003	White	Positive histology
2002	White	Positive histology
2002	White	Positive histology
2003	White	Positive histology
2003	White	Positive histology
2003	White	Positive histology
2003	Asian or Pacific Islander	Positive histology
2003	White	Positive histology
2003	White	Positive histology
2002	White	Positive histology
2003	Asian or Pacific Islander	Positive histology
2003	White	Positive histology
2003	American Indian/Alaska Nati	Positive histology
2003	White	Positive exfoliative cytology, no positive histology
2003	White	Positive histology
2008	Asian or Pacific Islander	Positive histology
2003	White	Positive exfoliative cytology, no positive histology
2003	White	Positive histology
2003	White	Positive histology
2003	White	Positive histology
2004	White	Positive histology
2004	White	Positive histology
2004	White	Positive histology
2004	White	Positive histology
2004	White	Positive histology
2004	White	Positive histology
2019	White	Radiography without microscopic confirm
2004	Asian or Pacific Islander	Positive histology
2004	White	Positive histology
2004	White	Positive histology
2004	White	Positive histology
2004	White	Positive histology
2004	White	Positive histology

2004	White	Positive histology
2004	White	Radiography without microscopic confirm
2004	White	Positive histology
2004	White	Positive histology
2004	White	Positive histology
2004	White	Positive histology
2004	White	Positive histology
2005	White	Positive histology
2004	White	Positive histology
2004	White	Positive histology
2004	White	Positive histology
2005	White	Positive histology
2005	White	Positive histology
2005	White	Positive histology
2005	White	Positive histology
2005	White	Positive histology
2005	White	Positive histology
2005	White	Positive histology
2005	White	Positive histology
2005	Asian or Pacific Islander	Positive histology
2005	White	Positive histology
2005	Asian or Pacific Islander	Positive histology
2005	White	Positive histology
2005	White	Positive histology
2005	White	Positive histology
2005	White	Positive histology
2005	Asian or Pacific Islander	Positive histology
2005	White	Positive histology
2005	White	Positive histology
2002	White	Unknown
2005	White	Positive histology
2003	White	Radiography without microscopic confirm
2006	White	Positive histology
2006	White	Positive histology
2006	White	Positive histology
2006	White	Positive histology
2006	White	Positive histology
2006	White	Positive histology
2006	Asian or Pacific Islander	Positive histology
2006	White	Positive histology
2006	White	Positive histology
2006	White	Positive histology
2006	White	Positive histology
2006	Asian or Pacific Islander	Positive histology
2005	White	Positive histology
2007	White	Positive histology
2006	White	Positive histology
2006	White	Positive histology
2007	White	Positive histology
2007	White	Positive histology
2006	White	Positive histology
2006	White	Positive histology
2006	White	Positive histology
2006	White	Positive histology
2006	White	Positive histology
2007	White	Positive histology
2007	White	Positive histology
2007	White	Positive histology

2007	White	Positive exfoliative cytology, no positive histology
2007	White	Positive histology
2007	Asian or Pacific Islander	Positive histology
2007	White	Positive histology
2007	White	Positive histology
2007	White	Positive histology
2007	White	Positive histology
2007	White	Positive histology
2007	White	Positive histology
2007	Black	Positive histology
2007	White	Positive histology
2002	White	Positive histology
2008	White	Radiography without microscopic confirm
2007	White	Positive histology
2007	White	Positive histology
2014	White	Positive histology
2007	White	Positive histology
2008	White	Positive histology
2008	Black	Positive histology
2001	White	Positive histology
2007	White	Positive exfoliative cytology, no positive histology
2007	White	Positive histology
2008	White	Positive histology
2010	White	Positive histology
2008	White	Radiography without microscopic confirm
2008	White	Positive histology
2008	White	Positive histology
2008	White	Positive histology
2008	White	Positive histology
2008	White	Positive histology
2008	White	Positive histology
2008	White	Positive histology
2008	White	Positive histology
2009	White	Positive histology
2008	White	Positive histology
2008	Asian or Pacific Islander	Positive histology
2009	White	Positive histology
2009	White	Positive histology
2009	White	Positive histology
2009	White	Positive histology
2009	White	Positive histology
2007	White	Positive histology
2008	Asian or Pacific Islander	Positive histology
2009	White	Positive histology
2009	White	Positive histology
2009	White	Positive histology
2009	White	Positive histology
2009	Black	Positive histology
2009	White	Positive histology
2009	White	Positive histology
2009	White	Positive histology
2009	Asian or Pacific Islander	Unknown
2009	White	Positive histology
2009	White	Positive histology
2009	White	Positive exfoliative cytology, no positive histology
2010	White	Positive histology
2010	White	Positive histology
2006	White	Positive histology

2010	White	Positive histology
2010	White	Positive histology
2010	White	Positive histology
2010	White	Positive histology
2010	White	Positive histology
2010	White	Positive histology
2010	White	Positive histology
2010	White	Positive histology
2010	White	Positive histology
2010	White	Positive histology
2011	White	Positive histology
2011	White	Positive histology
2010	Asian or Pacific Islander	Positive histology
2011	White	Positive histology
2011	White	Positive histology
2011	White	Positive histology
2010	White	Positive histology
2011	White	Positive histology
2011	Asian or Pacific Islander	Positive histology
2010	White	Positive histology
2010	White	Positive histology
2011	White	Positive histology
2011	White	Positive histology
2011	White	Positive histology
2011	White	Positive histology
2010	Unknown	Positive histology
2011	White	Positive histology
2010	White	Positive histology
2011	White	Positive histology
2011	White	Positive histology
2011	White	Positive histology
2011	Asian or Pacific Islander	Positive histology
2011	White	Positive histology
2011	White	Positive histology
2012	White	Positive histology
2012	White	Positive histology
2011	White	Positive histology
2012	White	Positive histology
2012	White	Positive histology
2012	White	Positive histology
2012	White	Positive histology
2012	White	Positive histology
2011	Black	Positive histology
2012	White	Positive histology
2011	White	Positive histology
2012	White	Positive histology
2012	Asian or Pacific Islander	Positive histology
2012	White	Positive histology
2012	White	Positive histology
2012	Asian or Pacific Islander	Positive histology
2012	White	Positive histology
2012	Asian or Pacific Islander	Positive histology
2012	White	Positive histology
2012	White	Positive histology
2012	White	Positive histology
2012	White	Positive histology
2012	White	Positive histology
2013	White	Positive histology

2013	White	Positive histology
2013	White	Positive histology
2013	Asian or Pacific Islander	Positive histology
2013	White	Positive histology
2013	White	Positive histology
2012	American Indian/Alaska Nati	Positive histology
2013	White	Positive histology
2013	American Indian/Alaska Nati	Unknown
2013	White	Positive histology
2013	White	Positive histology
2013	White	Positive histology
2013	Black	Positive histology
2013	White	Positive histology
2013	White	Positive histology
2013	White	Positive histology
2013	White	Positive histology
2013	White	Positive histology
2013	White	Positive histology
2014	White	Positive histology
2014	White	Positive histology
2014	White	Positive histology
2014	White	Positive histology
2014	White	Positive histology
2014	White	Positive histology
2014	White	Positive histology
2014	White	Positive histology
2014	White	Positive histology
2014	White	Positive histology
2014	White	Positive histology
2014	White	Positive histology
2018	White	Positive histology
2014	White	Positive histology
2014	White	Positive histology
2013	White	Positive histology
2014	White	Positive histology
2013	White	Positive histology
2014	White	Positive histology
2014	Asian or Pacific Islander	Positive histology
2014	Black	Positive histology
2015	White	Positive histology
2014	White	Positive histology
2014	White	Positive histology
2014	White	Positive histology
2015	White	Positive histology
2014	White	Positive histology
2015	White	Positive histology
2014	White	Positive histology
2015	Asian or Pacific Islander	Positive histology
2015	White	Positive histology
2015	White	Positive histology
2015	White	Positive histology
2014	White	Positive histology
2015	White	Radiography without microscopic confirm
2015	White	Positive histology
2015	White	Positive histology
2015	White	Positive histology
2015	White	Positive histology
2015	White	Positive histology

2014	White	Positive histology
2015	Asian or Pacific Islander	Positive histology
2015	White	Positive histology
2015	White	Positive histology
2013	White	Positive histology
2015	White	Positive histology
2015	White	Positive histology
2015	White	Positive histology
2018	White	Radiography without microscopic confirm
2015	White	Positive histology
2015	White	Positive histology
2015	White	Positive histology
2015	White	Positive histology
2015	White	Positive histology
2015	Unknown	Positive histology
2015	White	Positive histology
2015	Asian or Pacific Islander	Positive histology
2013	White	Unknown
2016	White	Positive histology
2018	White	Positive histology
2016	White	Positive histology
2016	White	Positive histology
2016	White	Positive histology
2016	White	Positive histology
2016	White	Positive histology
2016	White	Positive histology
2016	White	Positive histology
2016	White	Positive histology
2003	White	Positive histology
2016	White	Positive histology
2015	White	Positive histology
2016	White	Positive histology
2016	White	Positive histology
2016	White	Positive histology
2016	White	Positive histology
2016	White	Positive histology
2016	White	Positive histology
2016	White	Positive histology
2016	White	Positive histology
2017	White	Positive histology
2016	White	Positive histology
2016	Unknown	Positive histology
2016	White	Positive histology
2016	White	Positive histology
2017	White	Positive histology
2017	Asian or Pacific Islander	Positive histology
2017	White	Positive histology
2017	White	Positive histology
2017	Asian or Pacific Islander	Positive histology
2017	White	Positive histology
2017	White	Positive histology
2017	White	Positive histology
2017	White	Positive histology
2017	White	Positive histology
2017	White	Positive histology
2017	White	Positive histology
2017	Asian or Pacific Islander	Positive histology
2017	White	Positive histology

2017	White	Positive histology
2017	White	Positive histology
2017	White	Positive histology
2014	White	Unknown
2017	White	Positive histology
2017	White	Positive histology
2017	White	Radiography without microscopic confirm
2016	Unknown	Positive histology
2012	White	Positive histology
2018	White	Positive histology
2018	White	Positive histology
2018	White	Positive histology
2018	White	Positive histology
2018	White	Positive histology
2018	White	Positive histology
2018	White	Positive microscopic confirm, method not specified
2018	Asian or Pacific Islander	Positive histology
2018	Asian or Pacific Islander	Positive histology
2018	White	Positive histology
2018	White	Positive histology
2018	Asian or Pacific Islander	Positive histology
2018	Asian or Pacific Islander	Positive histology
2018	White	Positive histology
2019	White	Positive histology
2018	White	Positive histology
2018	White	Positive histology
2018	White	Positive histology
2018	White	Positive histology
2018	White	Positive histology
2018	Asian or Pacific Islander	Positive histology
2019	Asian or Pacific Islander	Positive histology
2019	White	Positive histology
2018	White	Positive histology
2019	White	Positive histology
2019	White	Positive histology
2019	White	Positive histology
2019	Black	Positive histology
2018	White	Positive histology
2019	White	Positive histology
2019	Asian or Pacific Islander	Positive histology
2019	White	Positive histology
2019	White	Positive histology
2019	White	Positive histology
2018	White	Positive histology
2018	White	Positive histology
2019	White	Positive histology
2019	White	Positive histology
2019	White	Positive histology
2019	Asian or Pacific Islander	Positive histology
2019	White	Positive histology
2019	White	Positive histology
2019	Asian or Pacific Islander	Positive histology
2019	White	Positive histology
2019	White	Positive histology
2019	White	Positive histology
2019	White	Positive histology
2000	White	Positive histology
2001	White	Positive histology

2000	White	Positive histology
2001	White	Positive histology
2001	White	Positive histology
2002	White	Positive histology
2000	Black	Positive histology
2002	White	Positive histology
2002	White	Positive histology
2001	White	Positive histology
2003	White	Radiography without microscopic confirm
2003	White	Positive histology
2003	White	Positive histology
2003	White	Positive histology
2003	White	Positive histology
2017	White	Positive histology
2003	White	Positive histology
2004	White	Positive histology
2003	White	Positive histology
2003	White	Positive histology
2004	White	Positive histology
2004	White	Positive histology
2004	White	Positive histology
2003	White	Positive histology
2001	White	Positive histology
2005	White	Positive histology
2005	White	Positive histology
2005	White	Positive histology
2006	White	Positive histology
2006	White	Positive histology
2007	White	Positive histology
2006	White	Positive histology
2006	White	Positive histology
2007	White	Positive histology
2007	White	Positive histology
2007	White	Positive histology
2008	White	Positive histology
2008	White	Positive histology
2008	White	Positive histology
2008	White	Positive histology
2009	White	Positive histology
2002	White	Positive histology
2008	White	Positive histology
2007	White	Positive histology
2008	White	Positive histology
2007	White	Positive histology
2010	White	Positive histology
2010	White	Positive histology
2009	White	Positive histology
2010	Black	Positive histology
2010	White	Positive histology
2011	White	Positive histology
2011	White	Positive histology
2011	White	Positive histology
2011	White	Positive histology
2011	White	Positive histology
2011	White	Positive histology
2012	White	Positive histology
2011	White	Positive histology
2012	White	Positive histology

2012	White	Positive histology
2012	White	Positive histology
2012	White	Positive histology
2012	White	Positive histology
2012	White	Positive histology
2012	White	Positive histology
2012	White	Positive exfoliative cytology, no positive histology
2012	White	Positive histology
2013	White	Positive histology
2013	White	Positive histology
2012	White	Positive histology
2012	White	Positive histology
2013	White	Positive histology
2004	Black	Positive histology
2015	White	Positive histology
2014	White	Positive exfoliative cytology, no positive histology
2014	White	Positive histology
2014	American Indian/Alaska Nati	Positive histology
2013	White	Positive histology
2014	White	Positive histology
2014	White	Positive histology
2015	White	Positive histology
2015	White	Positive histology
2015	White	Positive histology
2016	White	Positive histology
2016	White	Positive histology
2016	White	Positive exfoliative cytology, no positive histology
2015	White	Positive histology
2016	White	Positive histology
2016	White	Positive histology
2016	White	Positive histology
2016	White	Positive histology
2016	White	Positive histology
2016	White	Positive histology
2015	White	Positive histology
2015	Unknown	Unknown
2017	White	Positive histology
2017	White	Positive histology
2017	White	Positive histology
2017	White	Positive histology
2017	White	Positive histology
2017	White	Positive histology
2017	White	Positive histology
2018	White	Positive histology
2018	White	Positive histology
2018	Unknown	Positive histology
2019	White	Positive histology
2019	White	Positive histology
2000	Asian or Pacific Islander	Positive histology
2000	White	Positive histology
2000	White	Positive histology
2001	White	Positive histology
2002	White	Positive histology
2002	White	Positive histology
2003	Black	Positive histology
2003	White	Positive histology
2003	White	Positive histology
2004	White	Positive histology

2007	White	Positive histology
2007	White	Positive histology
2007	Black	Positive histology
2008	White	Positive histology
2004	White	Positive histology
2006	White	Positive histology
2002	White	Positive histology
2008	White	Positive histology
2007	White	Positive histology
2000	White	Positive histology
2003	White	Positive histology
2006	White	Positive histology
2007	White	Positive histology
2005	White	Positive histology
2003	White	Positive histology
2005	Black	Positive histology
2001	White	Positive histology
2001	White	Positive histology
2008	White	Positive histology
2008	White	Positive histology
2008	White	Positive histology
2009	White	Positive histology
2009	White	Positive histology
2009	White	Positive histology
2013	White	Positive histology
2009	White	Positive histology
2010	Black	Positive histology
2010	White	Positive histology
2010	White	Positive histology
2011	Asian or Pacific Islander	Positive histology
2011	Black	Positive histology
2010	Black	Positive histology
2011	White	Positive histology
2011	Black	Positive histology
2012	White	Positive histology
2011	White	Positive histology
2012	White	Positive histology
2011	White	Positive histology
2013	White	Positive histology
2012	American Indian/Alaska Nati	Positive histology
2012	White	Positive histology
2011	White	Positive histology
2012	White	Positive histology
2014	White	Positive histology
2014	White	Positive histology
2014	White	Positive histology
2015	White	Positive histology
2013	White	Positive histology
2014	White	Positive histology
2015	White	Positive histology
2014	White	Positive histology
2014	White	Positive histology
2014	White	Positive histology
2001	White	Positive histology
2013	Unknown	Positive histology
2016	White	Positive histology
2016	White	Positive histology
2016	White	Positive histology

2016	White	Positive histology
2016	White	Positive histology
2016	White	Positive histology
2016	White	Positive histology
2016	White	Positive histology
2017	White	Positive histology
2017	White	Positive histology
2018	White	Positive histology
2017	White	Positive histology
2018	White	Positive histology
2017	White	Positive histology
2018	White	Positive histology
2019	White	Positive histology
2018	White	Positive histology
2018	White	Positive histology
2018	White	Positive histology
2018	White	Positive histology
2019	White	Positive histology
2019	White	Positive histology
2019	White	Positive histology
2019	White	Positive histology
2007	White	Positive histology
2008	White	Positive histology
2011	White	Positive histology
2000	White	Positive histology
2012	White	Positive histology
2000	White	Positive histology
2000	White	Positive histology
2000	White	Positive histology
2000	White	Positive histology
2000	White	Positive histology
2000	White	Positive exfoliative cytology, no positive histology
2000	Black	Positive histology
2001	White	Positive histology
2001	White	Positive histology
2001	White	Positive histology
2001	White	Positive histology
2001	White	Positive histology
2001	White	Positive histology
2001	White	Positive histology
2001	White	Positive histology
2001	White	Positive histology
2001	White	Positive histology
2002	White	Positive histology
2002	White	Positive histology
2002	Asian or Pacific Islander	Positive histology
2002	White	Positive histology
2001	White	Positive histology
2001	White	Positive histology
2002	Black	Positive histology
2002	White	Positive histology
2002	White	Positive histology
2002	White	Positive histology
2002	Asian or Pacific Islander	Positive histology
2002	Black	Positive histology
2002	White	Positive histology
2002	White	Positive histology
2002	White	Positive histology

2002	White	Positive histology
2003	White	Positive histology
2002	White	Positive histology
2002	White	Positive histology
2000	Black	Positive histology
2003	White	Positive histology
2003	White	Positive histology
2003	White	Positive histology
2018	White	Positive histology
2003	White	Positive histology
2003	White	Positive histology
2003	White	Positive histology
2002	White	Positive histology
2001	White	Positive histology
2003	White	Positive histology
2004	White	Positive histology
2003	White	Positive histology
2003	White	Positive histology
2004	White	Positive histology
2002	White	Positive histology
2004	Black	Positive histology
2003	White	Positive histology
2003	White	Positive histology
2005	Asian or Pacific Islander	Positive histology
2004	White	Positive histology
2004	White	Positive histology
2004	Asian or Pacific Islander	Positive histology
2014	White	Positive histology
2005	White	Positive histology
2005	White	Positive histology
2005	White	Positive histology
2005	White	Positive histology
2005	White	Positive histology
2005	White	Positive histology
2005	White	Positive histology
2004	White	Positive histology
2004	White	Positive histology
2004	White	Positive histology
2005	White	Positive histology
2005	White	Positive histology
2013	White	Positive histology
2006	White	Positive histology
2005	White	Positive histology
2005	White	Positive histology
2006	White	Positive histology
2011	White	Positive histology
2006	White	Positive histology
2006	White	Positive histology
2006	Asian or Pacific Islander	Positive histology
2006	White	Positive histology
2006	White	Positive histology
2004	White	Positive histology
2006	White	Positive histology
2007	White	Positive histology
2007	White	Positive histology
2007	White	Positive histology
2007	White	Positive histology
2006	White	Positive histology

2006	White	Positive histology
2005	White	Positive histology
2007	White	Positive histology
2007	White	Positive histology
2007	White	Positive histology
2007	White	Positive histology
2008	White	Positive histology
2007	White	Positive histology
2008	White	Positive histology
2008	White	Positive histology
2008	White	Positive histology
2008	White	Positive histology
2008	White	Positive histology
2008	White	Positive histology
2007	White	Positive histology
2008	White	Positive histology
2008	Asian or Pacific Islander	Positive histology
2008	White	Positive histology
2007	Asian or Pacific Islander	Positive histology
2008	White	Positive histology
2008	White	Positive histology
2008	White	Positive histology
2016	White	Positive histology
2009	White	Positive histology
2009	White	Positive histology
2009	White	Positive histology
2008	White	Positive histology
2009	White	Positive histology
2009	White	Positive histology
2009	White	Positive histology
2009	White	Positive histology
2009	White	Positive histology
2009	White	Positive histology
2009	White	Positive histology
2009	White	Positive histology
2010	White	Radiography without microscopic confirm
2010	Black	Positive histology
2009	White	Positive histology
2010	White	Positive histology
2010	White	Positive histology
2010	White	Positive histology
2010	White	Positive histology
2010	White	Positive histology
2010	White	Positive histology
2011	White	Positive histology
2010	White	Positive histology
2011	White	Positive histology
2011	White	Positive histology
2009	White	Positive histology
2011	White	Positive histology
2010	White	Positive histology
2010	White	Positive histology
2010	White	Positive histology
2011	White	Positive histology
2011	White	Positive histology
2010	White	Positive histology
2011	White	Positive histology
2011	Asian or Pacific Islander	Positive histology

2011	White	Positive histology
2011	White	Positive histology
2011	White	Positive histology
2011	White	Positive histology
2011	White	Positive histology
2013	White	Positive histology
2012	White	Positive histology
2012	White	Positive histology
2012	White	Positive histology
2012	White	Positive histology
2012	White	Positive histology
2012	White	Positive histology
2012	White	Positive histology
2013	White	Positive histology
2012	White	Positive histology
2012	White	Positive histology
2013	White	Positive histology
2013	Black	Positive histology
2013	White	Positive histology
2018	White	Positive histology
2013	White	Positive histology
2013	White	Positive histology
2013	White	Positive histology
2014	White	Positive exfoliative cytology, no positive histology
2013	White	Positive histology
2013	White	Positive histology
2014	Black	Positive histology
2014	White	Positive histology
2014	White	Positive histology
2015	White	Positive histology
2014	White	Positive histology
2014	Asian or Pacific Islander	Positive histology
2013	White	Positive histology
2014	White	Positive histology
2014	White	Positive histology
2012	Black	Positive histology
2014	White	Positive histology
2014	White	Positive histology
2015	White	Positive histology
2014	White	Positive histology
2015	White	Positive histology
2014	White	Positive histology
2015	White	Positive histology
2015	Black	Positive histology
2015	White	Positive histology
2015	White	Positive histology
2015	White	Positive exfoliative cytology, no positive histology
2015	White	Positive histology
2015	White	Positive histology
2015	White	Positive histology
2016	White	Positive histology
2016	White	Positive histology
2016	White	Positive histology
2016	Black	Positive histology
2016	Unknown	Positive histology
2016	White	Positive histology
2016	White	Positive histology
2016	White	Positive histology

2016	White	Positive histology
2016	Asian or Pacific Islander	Positive histology
2016	White	Positive histology
2017	White	Positive histology
2016	White	Positive histology
2016	White	Positive histology
2016	White	Positive histology
2016	White	Positive histology
2016	White	Positive histology
2017	White	Positive histology
2016	White	Positive histology
2016	Asian or Pacific Islander	Positive histology
2017	White	Positive histology
2017	White	Positive histology
2017	White	Positive histology
2017	Asian or Pacific Islander	Positive histology
2017	White	Positive histology
2015	Unknown	Positive histology
2018	White	Positive histology
2018	White	Positive histology
2017	White	Positive histology
2017	White	Positive histology
2017	Asian or Pacific Islander	Positive histology
2018	White	Positive histology
2018	White	Positive histology
2018	Black	Positive histology
2018	White	Positive histology
2018	White	Positive histology
2018	White	Positive exfoliative cytology, no positive histology
2018	Asian or Pacific Islander	Positive histology
2018	White	Positive histology
2019	White	Positive histology
2005	Unknown	Positive histology
2018	Asian or Pacific Islander	Positive histology
2018	White	Positive histology
2019	White	Positive histology
2019	Unknown	Positive histology
2019	White	Positive histology
2019	White	Positive histology
2019	White	Positive histology
2019	White	Positive histology
2019	White	Positive histology
2019	Asian or Pacific Islander	Positive histology
2019	White	Positive histology
2019	Unknown	Positive histology
2019	White	Positive histology
2019	White	Positive histology
2019	Asian or Pacific Islander	Positive histology
2000	White	Positive histology
2000	White	Positive histology
2000	White	Positive histology
2000	White	Positive histology
2000	White	Positive histology
2000	White	Positive histology
2001	White	Positive histology
2001	Asian or Pacific Islander	Positive histology
2001	White	Positive histology
2001	White	Positive histology

2001	White	Positive histology
2001	Black	Positive histology
2001	White	Positive histology
2003	White	Positive histology
2012	White	Unknown
2002	White	Positive histology
2000	White	Positive histology
2002	White	Positive histology
2001	White	Positive histology
2001	Black	Positive histology
2002	White	Positive histology
2002	White	Positive histology
2002	Asian or Pacific Islander	Positive histology
2002	White	Positive histology
2003	Black	Positive histology
2001	White	Positive histology
2003	White	Positive histology
2004	White	Positive histology
2004	White	Positive histology
2003	Asian or Pacific Islander	Positive histology
2005	White	Positive histology
2005	White	Positive histology
2005	White	Positive histology
2005	Black	Positive histology
2005	White	Positive histology
2005	Black	Radiography without microscopic confirm
2005	Black	Positive histology
2005	White	Positive histology
2006	White	Positive histology
2004	White	Positive histology
2004	White	Positive histology
2006	White	Positive histology
2011	White	Positive histology
2006	White	Positive histology
2006	White	Positive exfoliative cytology, no positive histology
2006	Black	Positive histology
2006	White	Positive histology
2006	White	Positive histology
2005	White	Positive histology
2005	White	Positive histology
2006	White	Positive histology
2006	White	Positive histology
2007	White	Positive histology
2007	White	Positive histology
2007	White	Positive histology
2007	White	Positive histology
2007	White	Positive histology
2007	White	Positive histology
2008	White	Positive histology
2008	White	Positive histology
2008	White	Positive histology
2008	White	Positive histology
2008	White	Positive histology
2005	Black	Positive histology
2009	White	Positive histology
2010	White	Positive histology
2008	White	Positive histology
2010	White	Positive histology

2009	White	Positive histology
2009	White	Positive histology
2009	White	Positive histology
2010	White	Positive histology
2011	White	Positive histology
2011	White	Positive histology
2011	White	Positive histology
2010	White	Positive histology
2011	White	Positive histology
2010	White	Positive histology
2010	White	Positive histology
2011	White	Positive histology
2011	White	Positive histology
2011	White	Positive histology
2011	White	Positive histology
2011	White	Positive histology
2012	White	Positive histology
2012	Black	Positive histology
2012	White	Positive histology
2012	White	Positive histology
2012	White	Positive histology
2012	Black	Positive histology
2012	White	Positive histology
2012	White	Positive histology
2012	White	Positive histology
2012	White	Positive histology
2012	White	Positive histology
2012	White	Positive histology
2012	White	Positive histology
2012	White	Positive histology
2013	White	Positive histology
2013	White	Positive histology
2013	White	Positive histology
2013	White	Positive histology
2016	White	Positive histology
2013	White	Positive histology
2015	White	Positive histology
2014	White	Positive histology
2014	White	Positive histology
2014	White	Positive histology
2014	White	Positive histology
2013	White	Positive histology
2011	White	Positive histology
2014	White	Positive histology
2014	Black	Positive histology
2014	White	Positive histology
2014	White	Positive histology
2014	White	Positive histology
2014	White	Positive histology
2015	White	Positive histology
2015	Asian or Pacific Islander	Positive histology
2014	White	Positive histology
2015	White	Positive histology
2014	White	Positive histology
2015	White	Positive histology
2015	White	Positive histology
2015	Black	Positive histology
2016	White	Positive histology

2016	White	Positive histology
2017	White	Positive histology
2017	Black	Positive histology
2017	White	Positive histology
2017	White	Positive histology
2018	White	Positive histology
2018	White	Positive histology
2017	Black	Positive histology
2018	Asian or Pacific Islander	Positive histology
2019	White	Positive histology
2019	White	Positive histology
2019	White	Positive histology
2019	White	Positive histology
2019	Asian or Pacific Islander	Positive histology
2019	White	Positive histology
2019	White	Positive histology
2019	White	Positive histology

Sex	Age	T stage	N stage	M stage	Surg Prim Site	Radiation recode
Male	57 years	Blank(s)	Blank(s)	Blank(s)	0	Beam radiation
Female	08 years	T1	N0	M0	25	Beam radiation
Male	17 years	Blank(s)	Blank(s)	Blank(s)	30	None/Unknown
Female	12 years	Blank(s)	Blank(s)	Blank(s)	0	Beam radiation
Male	10 years	Blank(s)	Blank(s)	Blank(s)	0	Beam radiation
Male	14 years	Blank(s)	Blank(s)	Blank(s)	0	Beam radiation
Male	28 years	Blank(s)	Blank(s)	Blank(s)	30	None/Unknown
Female	50 years	Blank(s)	Blank(s)	Blank(s)	0	None/Unknown
Female	16 years	Blank(s)	Blank(s)	Blank(s)	0	None/Unknown
Female	16 years	Blank(s)	Blank(s)	Blank(s)	0	Beam radiation
Female	20 years	Blank(s)	Blank(s)	Blank(s)	0	None/Unknown
Female	18 years	Blank(s)	Blank(s)	Blank(s)	25	None/Unknown
Female	00 years	Blank(s)	Blank(s)	Blank(s)	25	None/Unknown
Female	16 years	TX	N0	M1b	0	Beam radiation
Male	06 years	TX	N0	M0	0	Beam radiation
Female	11 years	Blank(s)	Blank(s)	Blank(s)	0	Beam radiation
Male	12 years	T2	N0	M0	0	Beam radiation
Female	14 years	T3	N0	M1b	0	Beam radiation
Male	14 years	T2	N0	M1b	0	Beam radiation
Female	49 years	T1	N0	M0	30	Beam radiation
Male	45 years	T2	N0	M1a	41	None/Unknown
Male	15 years	T1	N0	M0	26	None/Unknown
Female	16 years	T1	N0	M0	0	Beam radiation
Male	11 years	T2	N0	M0	0	Beam radiation
Female	52 years	T3	N0	M1a	0	None/Unknown
Male	22 years	T1	N0	M0	26	Beam radiation
Male	17 years	T2	N1	M0	30	None/Unknown
Male	22 years	T2	N0	M0	0	Beam radiation
Male	11 years	T1	N0	M0	30	None/Unknown
Male	19 years	T2	N0	M1b	0	None/Unknown
Female	28 years	T1	N0	M0	30	None/Unknown
Male	11 years	T1	N1	M0	25	Beam radiation
Male	16 years	T1	N0	M0	0	Beam radiation
Male	77 years	T0	NX	M1b	0	None/Unknown
Female	06 years	T2	N0	M1b	30	Beam radiation
Male	10 years	T2	N0	M0	0	Beam radiation
Female	13 years	TX	N0	M0	0	Beam radiation
Male	12 years	T1	N0	M0	0	Beam radiation
Male	20 years	T2	N0	M0	30	Beam radiation
Female	24 years	T1	N0	M1a	30	Beam radiation
Male	04 years	T1	N0	M0	0	Beam radiation
Female	24 years	T1	N0	M0	41	None/Unknown
Female	27 years	T2	N0	M0	0	Radiation, NOS me
Female	09 years	T1	N0	M1a	26	Beam radiation
Female	14 years	T2	N0	M0	30	None/Unknown
Male	19 years	T1	N0	M1a	30	Beam radiation
Male	19 years	T2	N0	M0	30	None/Unknown
Male	12 years	T2	N1	M1b	0	Beam radiation
Male	24 years	T2	N0	M0	0	Beam radiation
Female	15 years	T2	N0	M1b	0	Beam radiation
Male	24 years	T2	N0	M1b	0	Beam radiation
Female	46 years	T1	N0	M0	30	None/Unknown
Female	11 years	T1	N0	M0	0	None/Unknown
Female	52 years	Blank(s)	Blank(s)	Blank(s)	0	Beam radiation
Male	11 years	T3	N0	M1NOS	0	Beam radiation
Male	01 years	T1	N0	M0	0	Beam radiation
Male	16 years	T2	N0	M0	0	Beam radiation

Male	19 years	T2	N0	M0	41	None/Unknown
Female	16 years	TX	N0	M0	0	Beam radiation
Female	10 years	T2	N0	M1b	0	Beam radiation
Female	18 years	T1	N0	M0	0	None/Unknown
Male	30 years	T3	N0	M1a	41	Beam radiation
Male	09 years	T1	N0	M0	0	Beam radiation
Male	15 years	T2	N0	M1b	0	Beam radiation
Male	31 years	Blank(s)	Blank(s)	Blank(s)	25	Beam radiation
Female	16 years	Blank(s)	Blank(s)	Blank(s)	0	Beam radiation
Female	15 years	TX	NX	MX	0	None/Unknown
Female	28 years	Blank(s)	Blank(s)	Blank(s)	0	Beam radiation
Male	28 years	Blank(s)	Blank(s)	Blank(s)	0	Beam radiation
Male	67 years	Blank(s)	Blank(s)	Blank(s)	0	None/Unknown
Male	29 years	Blank(s)	Blank(s)	Blank(s)	30	Beam radiation
Male	57 years	Blank(s)	Blank(s)	Blank(s)	30	None/Unknown
Male	50 years	Blank(s)	Blank(s)	Blank(s)	30	None/Unknown
Male	17 years	Blank(s)	Blank(s)	Blank(s)	0	Beam radiation
Male	32 years	Blank(s)	Blank(s)	Blank(s)	0	Beam radiation
Male	20 years	Blank(s)	Blank(s)	Blank(s)	25	Beam radiation
Female	29 years	Blank(s)	Blank(s)	Blank(s)	30	None/Unknown
Male	17 years	Blank(s)	Blank(s)	Blank(s)	30	None/Unknown
Male	15 years	Blank(s)	Blank(s)	Blank(s)	25	Beam radiation
Male	24 years	Blank(s)	Blank(s)	Blank(s)	0	Beam radiation
Female	55 years	Blank(s)	Blank(s)	Blank(s)	0	Beam radiation
Male	16 years	Blank(s)	Blank(s)	Blank(s)	0	Beam radiation
Female	08 years	Blank(s)	Blank(s)	Blank(s)	25	None/Unknown
Female	09 years	Blank(s)	Blank(s)	Blank(s)	30	None/Unknown
Female	06 years	Blank(s)	Blank(s)	Blank(s)	0	Beam radiation
Male	19 years	Blank(s)	Blank(s)	Blank(s)	90	None/Unknown
Female	62 years	Blank(s)	Blank(s)	Blank(s)	30	Recommended, un
Male	11 years	Blank(s)	Blank(s)	Blank(s)	0	Beam radiation
Male	20 years	Blank(s)	Blank(s)	Blank(s)	30	None/Unknown
Male	24 years	Blank(s)	Blank(s)	Blank(s)	90	None/Unknown
Male	29 years	Blank(s)	Blank(s)	Blank(s)	0	Radioisotopes (198
Male	19 years	Blank(s)	Blank(s)	Blank(s)	0	None/Unknown
Male	21 years	Blank(s)	Blank(s)	Blank(s)	30	None/Unknown
Male	50 years	Blank(s)	Blank(s)	Blank(s)	0	Beam radiation
Male	41 years	Blank(s)	Blank(s)	Blank(s)	25	Beam radiation
Female	11 years	TX	N0	M0	0	Beam radiation
Male	17 years	T2	N0	M0	0	Beam radiation
Female	15 years	T1	N0	M0	25	Beam radiation
Female	18 years	T1	N0	M0	30	Beam radiation
Female	11 years	TX	N0	M0	26	Beam radiation
Male	18 years	T1	N0	M0	0	Beam radiation
Female	51 years	TX	N0	M1b	0	None/Unknown
Male	24 years	TX	N0	M1b	0	Beam radiation
Male	09 years	TX	N0	M0	25	None/Unknown
Male	19 years	TX	NX	MX	0	Beam radiation
Male	19 years	T1	N0	M0	25	Beam radiation
Male	37 years	Blank(s)	Blank(s)	Blank(s)	99	Beam radiation
Male	08 years	T1	N0	M0	25	None/Unknown
Male	10 years	T1	N0	M0	30	None/Unknown
Male	29 years	T2	NX	MX	0	Recommended, un
Female	20 years	T1	N0	M0	30	None/Unknown
Male	11 years	TX	N1	M1b	0	None/Unknown
Male	22 years	T2	N0	M1b	0	Beam radiation
Female	13 years	T2	N0	M0	26	Beam radiation
Male	08 years	TX	NX	M0	0	Beam radiation

Female	25 years	T1	N0	M0	30	None/Unknown
Female	20 years	TX	N0	M0	0	None/Unknown
Male	52 years	T1	N0	M0	30	Beam radiation
Male	12 years	T2	N0	M0	0	None/Unknown
Male	12 years	T1	NX	M1a	0	None/Unknown
Male	50 years	Blank(s)	Blank(s)	Blank(s)	26	Beam radiation
Male	10 years	TX	NX	M0	53	None/Unknown
Male	25 years	T1	N0	M0	26	None/Unknown
Female	09 years	T1	N0	M0	25	Beam radiation
Male	35 years	T1	N0	M1NOS	0	None/Unknown
Female	59 years	Blank(s)	Blank(s)	Blank(s)	0	Beam radiation
Male	14 years	TX	N0	M0	0	Beam radiation
Male	33 years	T2	N0	M0	30	Beam radiation
Female	11 years	T3	N0	M1b	0	None/Unknown
Female	14 years	T1	N0	M0	30	None/Unknown
Male	07 years	TX	NX	M0	30	None/Unknown
Female	03 years	TX	N0	M1b	40	Beam radiation
Female	42 years	T1	N0	M0	26	Beam radiation
Male	12 years	T3	N0	M1b	0	Beam radiation
Male	31 years	T1	NX	M0	26	None/Unknown
Male	16 years	T1	N0	M0	30	Beam radiation
Male	21 years	T1	N0	M0	30	None/Unknown
Female	10 years	T1	N0	M1NOS	0	Beam radiation
Male	25 years	TX	N0	M1b	0	Beam radiation
Female	11 years	T2	N0	M0	41	None/Unknown
Male	23 years	T1	N0	M0	30	None/Unknown
Male	12 years	T1	N0	M0	30	Recommended, un
Male	18 years	TX	N0	M1NOS	0	Beam radiation
Female	20 years	T1	N0	M0	30	None/Unknown
Male	18 years	T2	N0	M0	30	Beam radiation
Female	12 years	T1	N0	M0	25	Beam radiation
Male	19 years	T2	N0	M0	30	None/Unknown
Male	12 years	T2	N0	M1b	0	Beam radiation
Female	22 years	T2	N0	M1a	0	Combination of be
Female	15 years	T1	N0	M1a	30	None/Unknown
Female	13 years	T1	N0	M0	30	None/Unknown
Male	07 years	T1	N0	M0	30	None/Unknown
Female	38 years	Blank(s)	Blank(s)	Blank(s)	0	None/Unknown
Female	77 years	Blank(s)	Blank(s)	Blank(s)	0	None/Unknown
Male	14 years	Blank(s)	Blank(s)	Blank(s)	0	None/Unknown
Male	12 years	Blank(s)	Blank(s)	Blank(s)	30	Beam radiation
Male	20 years	Blank(s)	Blank(s)	Blank(s)	0	Beam radiation
Male	15 years	Blank(s)	Blank(s)	Blank(s)	0	Combination of be
Female	08 years	Blank(s)	Blank(s)	Blank(s)	30	Beam radiation
Male	25 years	TX	N0	M1a	0	Beam radiation
Female	12 years	Blank(s)	Blank(s)	Blank(s)	30	None/Unknown
Female	16 years	Blank(s)	Blank(s)	Blank(s)	30	Beam radiation
Male	33 years	Blank(s)	Blank(s)	Blank(s)	30	Beam radiation
Male	22 years	Blank(s)	Blank(s)	Blank(s)	30	Beam radiation
Male	17 years	Blank(s)	Blank(s)	Blank(s)	26	Beam radiation
Male	58 years	Blank(s)	Blank(s)	Blank(s)	30	None/Unknown
Male	04 years	Blank(s)	Blank(s)	Blank(s)	0	Beam radiation
Female	05 years	Blank(s)	Blank(s)	Blank(s)	25	Beam radiation
Female	43 years	Blank(s)	Blank(s)	Blank(s)	0	None/Unknown
Male	29 years	Blank(s)	Blank(s)	Blank(s)	0	Beam radiation
Female	07 years	Blank(s)	Blank(s)	Blank(s)	0	Beam radiation
Male	23 years	Blank(s)	Blank(s)	Blank(s)	0	Recommended, un
Male	15 years	T1	N0	M0	26	Beam radiation

Female	29 years	TX	NX	MX	0	Beam radiation
Female	05 years	T3	NX	M1b	30	None/Unknown
Male	16 years	T1	N1	M0	30	None/Unknown
Male	18 years	TX	N0	M1b	0	Beam radiation
Female	06 years	T1	N0	M0	26	None/Unknown
Male	18 years	T3	N0	M1a	0	Beam radiation
Female	38 years	T1	N1	M1a	40	None/Unknown
Male	11 years	T2	N1	M0	0	None/Unknown
Female	08 years	T1	N0	M1NOS	0	Beam radiation
Male	16 years	T2	N0	M1a	30	None/Unknown
Male	26 years	T3	N0	M1b	0	None/Unknown
Male	17 years	Blank(s)	Blank(s)	Blank(s)	50	None/Unknown
Female	03 years	Blank(s)	Blank(s)	Blank(s)	30	None/Unknown
Female	32 years	Blank(s)	Blank(s)	Blank(s)	41	None/Unknown
Male	17 years	Blank(s)	Blank(s)	Blank(s)	30	None/Unknown
Female	00 years	Blank(s)	Blank(s)	Blank(s)	25	None/Unknown
Male	06 years	Blank(s)	Blank(s)	Blank(s)	30	None/Unknown
Male	13 years	Blank(s)	Blank(s)	Blank(s)	30	Beam radiation
Male	11 years	Blank(s)	Blank(s)	Blank(s)	26	None/Unknown
Male	09 years	Blank(s)	Blank(s)	Blank(s)	25	None/Unknown
Male	09 years	Blank(s)	Blank(s)	Blank(s)	26	Beam radiation
Male	11 years	Blank(s)	Blank(s)	Blank(s)	0	Beam radiation
Male	09 years	Blank(s)	Blank(s)	Blank(s)	41	None/Unknown
Male	18 years	Blank(s)	Blank(s)	Blank(s)	26	None/Unknown
Male	15 years	Blank(s)	Blank(s)	Blank(s)	26	None/Unknown
Male	19 years	Blank(s)	Blank(s)	Blank(s)	41	None/Unknown
Male	16 years	Blank(s)	Blank(s)	Blank(s)	41	Beam radiation
Male	10 years	Blank(s)	Blank(s)	Blank(s)	26	Beam radiation
Female	12 years	Blank(s)	Blank(s)	Blank(s)	0	None/Unknown
Male	07 years	Blank(s)	Blank(s)	Blank(s)	41	None/Unknown
Male	11 years	Blank(s)	Blank(s)	Blank(s)	30	None/Unknown
Female	17 years	Blank(s)	Blank(s)	Blank(s)	30	None/Unknown
Male	07 years	Blank(s)	Blank(s)	Blank(s)	25	None/Unknown
Female	08 years	Blank(s)	Blank(s)	Blank(s)	30	None/Unknown
Female	16 years	Blank(s)	Blank(s)	Blank(s)	30	Beam radiation
Male	07 years	Blank(s)	Blank(s)	Blank(s)	0	Beam radiation
Female	09 years	Blank(s)	Blank(s)	Blank(s)	26	None/Unknown
Female	33 years	T1	N0	M0	0	None/Unknown
Male	07 years	Blank(s)	Blank(s)	Blank(s)	30	Beam radiation
Female	48 years	T1	N0	M0	0	Beam radiation
Male	21 years	TX	N0	M1a	0	None/Unknown
Male	26 years	T1	NX	M1a	26	Beam radiation
Female	13 years	TX	N0	M0	30	None/Unknown
Female	13 years	T1	N0	M0	30	None/Unknown
Male	05 years	T1	N0	M0	30	None/Unknown
Male	23 years	T2	N0	M0	0	None/Unknown
Female	12 years	T2	N1	M1b	0	Beam radiation
Male	14 years	T2	N0	M1b	0	None/Unknown
Male	05 years	T1	N0	M0	52	None/Unknown
Female	47 years	T2	N0	M1a	25	Beam radiation
Female	14 years	T1	N0	M0	30	None/Unknown
Male	32 years	T0	NX	M1b	0	None/Unknown
Female	02 years	T1	N0	M0	30	None/Unknown
Male	18 years	TX	N0	M0	26	Beam radiation
Male	14 years	T3	NX	M1b	0	Beam radiation
Male	42 years	T1	N0	M0	25	Beam radiation
Female	02 years	T1	N0	M0	42	None/Unknown
Female	11 years	T2	N0	M0	0	None/Unknown

Male	18 years	T2	N0	M1b	0	None/Unknown
Male	21 years	T1	N0	M0	25	None/Unknown
Female	39 years	TX	N0	M0	26	Beam radiation
Female	17 years	T2	N0	M0	30	Beam radiation
Male	11 years	T2	N0	M0	30	None/Unknown
Male	08 years	T1	N0	M0	30	None/Unknown
Female	07 years	TX	NX	M1b	0	Beam radiation
Male	17 years	T2	N0	M0	0	Beam radiation
Female	08 years	TX	N0	M1b	90	Beam radiation
Male	14 years	T2	N0	M0	0	Beam radiation
Male	15 years	T1	N0	M1b	26	Beam radiation
Female	01 years	TX	N0	M0	0	Beam radiation
Male	15 years	T2	N0	M0	0	Beam radiation
Male	15 years	T2	N0	M1a	30	Beam radiation
Female	74 years	T1	N0	M0	25	None/Unknown
Male	15 years	TX	N0	M1b	26	Beam radiation
Female	62 years	T1	N0	M0	0	Beam radiation
Female	23 years	T2	N0	M0	0	Beam radiation
Female	12 years	T2	N0	M0	30	None/Unknown
Male	16 years	TX	N0	M1NOS	0	Beam radiation
Male	19 years	T2	N0	M0	41	None/Unknown
Male	25 years	TX	N0	M0	25	Beam radiation
Male	18 years	TX	NX	M1a	30	None/Unknown
Male	20 years	T1	N0	M0	30	None/Unknown
Male	19 years	T1	N1	M1b	30	None/Unknown
Male	21 years	T1	N0	M1b	0	None/Unknown
Male	14 years	T3	N0	M1b	0	Beam radiation
Female	31 years	T2	N0	M0	30	None/Unknown
Female	79 years	Blank(s)	Blank(s)	Blank(s)	25	Refused (1988+)
Male	09 years	T1	NX	M0	25	None/Unknown
Female	04 years	T1	N0	M0	30	None/Unknown
Male	13 years	T2	N0	M0	30	None/Unknown
Male	15 years	T2	N0	M0	30	Beam radiation
Male	15 years	Blank(s)	Blank(s)	Blank(s)	30	None/Unknown
Female	70 years	Blank(s)	Blank(s)	Blank(s)	0	Beam radiation
Female	10 years	Blank(s)	Blank(s)	Blank(s)	30	None/Unknown
Male	17 years	Blank(s)	Blank(s)	Blank(s)	30	Beam radiation
Male	13 years	Blank(s)	Blank(s)	Blank(s)	41	None/Unknown
Male	12 years	Blank(s)	Blank(s)	Blank(s)	30	None/Unknown
Female	17 years	Blank(s)	Blank(s)	Blank(s)	26	Beam radiation
Male	27 years	Blank(s)	Blank(s)	Blank(s)	0	None/Unknown
Female	65 years	Blank(s)	Blank(s)	Blank(s)	0	Beam radiation
Male	06 years	Blank(s)	Blank(s)	Blank(s)	30	None/Unknown
Male	16 years	Blank(s)	Blank(s)	Blank(s)	30	None/Unknown
Male	11 years	Blank(s)	Blank(s)	Blank(s)	25	Beam radiation
Female	26 years	Blank(s)	Blank(s)	Blank(s)	0	Beam radiation
Male	25 years	Blank(s)	Blank(s)	Blank(s)	41	Beam radiation
Male	10 years	Blank(s)	Blank(s)	Blank(s)	26	None/Unknown
Male	15 years	Blank(s)	Blank(s)	Blank(s)	26	Beam radiation
Female	22 years	Blank(s)	Blank(s)	Blank(s)	25	None/Unknown
Male	31 years	Blank(s)	Blank(s)	Blank(s)	0	None/Unknown
Female	07 years	Blank(s)	Blank(s)	Blank(s)	90	None/Unknown
Male	24 years	Blank(s)	Blank(s)	Blank(s)	26	None/Unknown
Male	16 years	Blank(s)	Blank(s)	Blank(s)	0	None/Unknown
Male	13 years	Blank(s)	Blank(s)	Blank(s)	30	Beam radiation
Male	19 years	Blank(s)	Blank(s)	Blank(s)	19	None/Unknown
Female	11 years	T2	NX	M1NOS	0	Beam radiation
Female	14 years	Blank(s)	Blank(s)	Blank(s)	0	Beam radiation

Female	11 years	Blank(s)	Blank(s)	Blank(s)	19	Beam radiation	
Male	22 years	Blank(s)	Blank(s)	Blank(s)	0	None/Unknown	
Female	13 years	T1	NX	M0	25	None/Unknown	
Female	16 years	T1	N0	M0	30	None/Unknown	
Female	20 years	T1	N0	M0	90	None/Unknown	
Male	30 years	T3	N0	M1b	0	Beam radiation	
Female	17 years	TX	N0	M0	30	None/Unknown	
Male	15 years	T2	N0	M0	26	Beam radiation	
Male	26 years	TX	NX	M0	0	Radiation, NOS	me
Male	11 years	TX	NX	MX	40	None/Unknown	
Male	10 years	TX	NX	MX	0	None/Unknown	
Male	14 years	T1	N0	M0	0	None/Unknown	
Female	07 years	TX	NX	MX	30	None/Unknown	
Male	20 years	T2	NX	MX	0	None/Unknown	
Female	34 years	TX	N0	M0	30	Beam radiation	
Female	26 years	TX	N0	M1b	0	Beam radiation	
Female	07 years	TX	NX	MX	53	None/Unknown	
Male	18 years	T2	N0	MX	30	None/Unknown	
Female	16 years	T1	NX	M1b	0	None/Unknown	
Male	30 years	TX	NX	MX	25	Beam radiation	
Male	46 years	T1	N0	M0	0	Beam radiation	
Female	10 years	T2	N0	M0	30	None/Unknown	
Female	17 years	T2	N0	M0	30	None/Unknown	
Female	23 years	TX	NX	MX	99	None/Unknown	
Male	06 years	T2	N0	M0	0	None/Unknown	
Male	07 years	T2	N0	M1b	0	None/Unknown	
Male	13 years	T3	N0	M0	25	None/Unknown	
Female	45 years	Blank(s)	Blank(s)	Blank(s)	30	None/Unknown	
Male	11 years	Blank(s)	Blank(s)	Blank(s)	25	Beam radiation	
Female	36 years	Blank(s)	Blank(s)	Blank(s)	0	None/Unknown	
Female	26 years	Blank(s)	Blank(s)	Blank(s)	30	None/Unknown	
Female	07 years	Blank(s)	Blank(s)	Blank(s)	0	Beam radiation	
Male	15 years	Blank(s)	Blank(s)	Blank(s)	30	None/Unknown	
Female	19 years	Blank(s)	Blank(s)	Blank(s)	99	None/Unknown	
Female	07 years	Blank(s)	Blank(s)	Blank(s)	0	None/Unknown	
Male	30 years	Blank(s)	Blank(s)	Blank(s)	99	None/Unknown	
Male	59 years	Blank(s)	Blank(s)	Blank(s)	0	None/Unknown	
Male	57 years	T2	N0	M1b	0	Beam radiation	
Male	09 years	Blank(s)	Blank(s)	Blank(s)	30	None/Unknown	
Female	31 years	Blank(s)	Blank(s)	Blank(s)	30	Beam radiation	
Female	25 years	Blank(s)	Blank(s)	Blank(s)	30	None/Unknown	
Male	22 years	Blank(s)	Blank(s)	Blank(s)	30	Beam radiation	
Male	19 years	Blank(s)	Blank(s)	Blank(s)	0	Beam radiation	
Female	15 years	Blank(s)	Blank(s)	Blank(s)	30	None/Unknown	
Male	56 years	Blank(s)	Blank(s)	Blank(s)	41	None/Unknown	
Female	12 years	Blank(s)	Blank(s)	Blank(s)	0	Beam radiation	
Male	11 years	Blank(s)	Blank(s)	Blank(s)	30	Beam radiation	
Female	18 years	Blank(s)	Blank(s)	Blank(s)	30	Beam radiation	
Female	27 years	Blank(s)	Blank(s)	Blank(s)	0	None/Unknown	
Male	17 years	Blank(s)	Blank(s)	Blank(s)	0	Refused (1988+)	
Male	26 years	Blank(s)	Blank(s)	Blank(s)	30	None/Unknown	
Male	23 years	Blank(s)	Blank(s)	Blank(s)	26	Beam radiation	
Female	13 years	Blank(s)	Blank(s)	Blank(s)	41	None/Unknown	
Male	14 years	Blank(s)	Blank(s)	Blank(s)	30	None/Unknown	
Female	09 years	TX	N0	M0	41	None/Unknown	
Female	08 years	T1	N0	M0	30	Beam radiation	
Male	62 years	TX	NX	MX	99	None/Unknown	
Female	18 years	T1	N0	M1b	30	Beam radiation	

Male	15 years	T1	N0	M0	30	Beam radiation
Male	17 years	TX	N1	M1b	0	Beam radiation
Male	20 years	T2	N0	M0	30	None/Unknown
Male	12 years	TX	N0	M0	26	Beam radiation
Male	25 years	T2	N0	M0	30	Beam radiation
Male	12 years	T1	N0	M0	0	Beam radiation
Male	09 years	TX	N0	M1a	30	None/Unknown
Female	15 years	TX	N0	M0	30	None/Unknown
Male	19 years	T1	NX	M1b	0	None/Unknown
Female	15 years	T2	N0	M1a	30	Beam radiation
Male	14 years	T1	N0	M0	0	Beam radiation
Male	15 years	TX	N0	M0	41	None/Unknown
Female	15 years	TX	N0	M0	30	None/Unknown
Female	24 years	T1	N0	M0	30	Beam radiation
Female	14 years	T1	N0	M0	0	None/Unknown
Male	09 years	T1	N0	M0	0	None/Unknown
Male	33 years	T3	NX	M0	25	None/Unknown
Female	18 years	TX	NX	M1b	0	Beam radiation
Male	21 years	T2	N0	M1b	0	Beam radiation
Male	12 years	T1	N0	M0	30	None/Unknown
Male	20 years	T2	N0	M1b	30	Beam radiation
Male	55 years	T2	N0	M0	30	Beam radiation
Male	11 years	TX	N0	M0	30	Beam radiation
Male	13 years	TX	N0	M0	30	None/Unknown
Female	13 years	T1	N0	M0	30	None/Unknown
Female	47 years	T1	N0	M1a	0	None/Unknown
Male	05 years	T2	N0	M0	0	Beam radiation
Female	31 years	T1	N0	M0	0	Beam radiation
Male	27 years	T2	N0	M0	0	Beam radiation
Female	05 years	T1	N0	M0	30	None/Unknown
Male	06 years	T1	N0	M1a	25	Beam radiation
Male	25 years	T1	N0	M0	30	None/Unknown
Male	17 years	TX	N0	M0	30	None/Unknown
Female	22 years	T2	N0	M0	30	None/Unknown
Male	13 years	T2	N0	M0	30	Beam radiation
Female	26 years	T1	N0	M0	30	Beam radiation
Male	22 years	TX	N0	M0	25	Beam radiation
Male	16 years	TX	N0	M0	30	Beam radiation
Male	20 years	T2	N0	M0	30	None/Unknown
Male	10 years	T1	N0	M0	30	None/Unknown
Female	12 years	TX	N0	M0	0	Beam radiation
Male	12 years	T2	N0	M0	30	None/Unknown
Male	18 years	T1	N0	M0	30	None/Unknown
Male	18 years	T2	N0	M0	0	Beam radiation
Male	18 years	T1	N0	M1a	30	Beam radiation
Female	15 years	T1	N0	M0	30	Beam radiation
Male	19 years	T2	N0	M0	0	Beam radiation
Male	19 years	T2	N0	M0	30	Beam radiation
Male	23 years	TX	N0	M0	30	Beam radiation
Female	14 years	T2	N0	M0	30	None/Unknown
Male	06 years	T1	N0	M0	26	Beam radiation
Male	12 years	T1	N0	M0	30	Recommended, un
Female	10 years	TX	N0	M0	30	None/Unknown
Male	18 years	T2	N0	M1b	0	Beam radiation
Female	14 years	T2	N0	M0	0	Beam radiation
Female	11 years	T2	N0	M0	30	None/Unknown
Male	09 years	T2	N0	M0	30	None/Unknown
Male	56 years	T2	N1	M0	30	Beam radiation

Female	12 years	T1	N0	M0	0	Beam radiation
Male	12 years	T1	N0	M0	26	None/Unknown
Male	50 years	T2	N0	M1b	0	Beam radiation
Female	12 years	TX	N0	M1NOS	0	Refused (1988+)
Male	53 years	T2	N0	M0	0	Beam radiation
Male	33 years	TX	N0	M0	25	Beam radiation
Male	14 years	T1	N0	M0	30	None/Unknown
Male	14 years	T2	N0	M0	30	None/Unknown
Male	23 years	Blank(s)	Blank(s)	Blank(s)	0	None/Unknown
Male	27 years	Blank(s)	Blank(s)	Blank(s)	30	None/Unknown
Male	27 years	Blank(s)	Blank(s)	Blank(s)	0	Beam radiation
Male	16 years	Blank(s)	Blank(s)	Blank(s)	0	Beam radiation
Female	01 years	Blank(s)	Blank(s)	Blank(s)	0	Beam radiation
Female	13 years	Blank(s)	Blank(s)	Blank(s)	30	None/Unknown
Male	23 years	Blank(s)	Blank(s)	Blank(s)	0	None/Unknown
Male	21 years	Blank(s)	Blank(s)	Blank(s)	0	Beam radiation
Male	23 years	Blank(s)	Blank(s)	Blank(s)	30	None/Unknown
Female	11 years	Blank(s)	Blank(s)	Blank(s)	0	Beam radiation
Female	21 years	Blank(s)	Blank(s)	Blank(s)	0	Beam radiation
Male	16 years	Blank(s)	Blank(s)	Blank(s)	30	None/Unknown
Male	19 years	Blank(s)	Blank(s)	Blank(s)	0	Beam radiation
Male	19 years	Blank(s)	Blank(s)	Blank(s)	0	Beam radiation
Female	09 years	Blank(s)	Blank(s)	Blank(s)	25	None/Unknown
Male	17 years	Blank(s)	Blank(s)	Blank(s)	41	None/Unknown
Female	11 years	Blank(s)	Blank(s)	Blank(s)	30	None/Unknown
Male	61 years	Blank(s)	Blank(s)	Blank(s)	40	Beam radiation
Female	37 years	Blank(s)	Blank(s)	Blank(s)	41	None/Unknown
Female	18 years	Blank(s)	Blank(s)	Blank(s)	0	Beam radiation
Male	15 years	Blank(s)	Blank(s)	Blank(s)	30	Beam radiation
Female	21 years	Blank(s)	Blank(s)	Blank(s)	0	Beam radiation
Male	14 years	Blank(s)	Blank(s)	Blank(s)	30	Beam radiation
Male	11 years	Blank(s)	Blank(s)	Blank(s)	30	Beam radiation
Female	44 years	Blank(s)	Blank(s)	Blank(s)	26	None/Unknown
Male	16 years	Blank(s)	Blank(s)	Blank(s)	53	Beam radiation
Male	19 years	Blank(s)	Blank(s)	Blank(s)	30	None/Unknown
Male	32 years	Blank(s)	Blank(s)	Blank(s)	26	Beam radiation
Female	19 years	Blank(s)	Blank(s)	Blank(s)	30	None/Unknown
Female	25 years	Blank(s)	Blank(s)	Blank(s)	0	Beam radiation
Male	17 years	T2	N0	M1b	30	Beam radiation
Female	12 years	TX	N0	M0	30	Beam radiation
Female	19 years	T1	N0	M0	25	None/Unknown
Male	30 years	T2	NX	M0	30	None/Unknown
Female	11 years	TX	N0	M0	30	Beam radiation
Female	07 years	TX	N0	M0	25	Beam radiation
Male	70 years	T1	N0	M1b	0	Beam radiation
Male	15 years	TX	N0	M0	0	Beam radiation
Male	27 years	T2	N0	M1b	0	Beam radiation
Male	35 years	T2	N0	M1a	30	Beam radiation
Male	36 years	T2	N0	M0	30	Beam radiation
Male	24 years	TX	N0	M0	25	Beam radiation
Male	16 years	T2	N1	M1b	0	Beam radiation
Male	19 years	TX	NX	M0	30	Beam radiation
Male	20 years	TX	N0	M0	0	None/Unknown
Male	13 years	T2	N0	M0	30	None/Unknown
Male	27 years	T1	N0	M0	25	None/Unknown
Female	15 years	TX	N0	M0	30	None/Unknown
Male	09 years	T3	N0	M0	30	Beam radiation
Male	09 years	TX	N0	M1a	41	None/Unknown

Male	21 years	T2	N0	M1a	0	None/Unknown
Male	09 years	T2	N0	M0	41	Beam radiation
Female	14 years	T1	N1	M0	0	Beam radiation
Male	21 years	T2	N0	M1a	0	Beam radiation
Male	12 years	T2	N0	M0	26	Beam radiation
Female	14 years	T2	N0	M1b	0	Beam radiation
Female	06 years	T2	N0	M0	30	None/Unknown
Female	12 years	T1	N0	M0	25	Beam radiation
Female	14 years	T2	N0	M0	53	None/Unknown
Male	11 years	T2	N0	M0	19	None/Unknown
Female	07 years	T1	N0	M0	26	Beam radiation
Female	63 years	TX	N0	M0	25	Beam radiation
Female	19 years	T3	N0	M1b	26	Beam radiation
Female	03 years	TX	N0	M0	0	Beam radiation
Female	16 years	T1	N0	M0	30	Beam radiation
Male	16 years	T1	N0	M0	30	None/Unknown
Female	25 years	T2	N0	M1b	0	Beam radiation
Female	28 years	T0	NX	M1b	0	Beam radiation
Male	15 years	T2	N0	M0	41	None/Unknown
Male	17 years	T1	N0	M0	30	None/Unknown
Male	08 years	T1	N0	M0	25	Beam radiation
Male	18 years	T1	N0	M0	30	None/Unknown
Male	23 years	T0	NX	M1b	0	None/Unknown
Male	06 years	T1	N0	M0	0	Beam radiation
Male	10 years	T1	N0	M0	41	None/Unknown
Female	85 years	T1	N0	M0	0	Beam radiation
Female	25 years	TX	N0	M0	30	None/Unknown
Male	13 years	T2	N1	M1b	0	Beam radiation
Male	29 years	T1	N0	M1b	0	Beam radiation
Male	16 years	T1	N0	M0	0	Beam radiation
Female	11 years	T2	N1	M1b	0	None/Unknown
Male	31 years	T2	N0	M0	0	Beam radiation
Male	12 years	T2	N0	M0	0	Beam radiation
Male	17 years	T2	N0	M1NOS	30	Beam radiation
Male	21 years	T1	N0	M1b	0	Beam radiation
Male	18 years	T1	N0	M0	25	None/Unknown
Female	10 years	T2	N0	M0	30	Beam radiation
Male	20 years	T2	N0	M0	30	Beam radiation
Female	06 years	T1	N0	M0	30	None/Unknown
Female	18 years	T1	N0	M1b	30	None/Unknown
Female	00 years	T3	N0	M0	30	None/Unknown
Male	23 years	T1	N0	M0	30	None/Unknown
Male	65 years	T1	N0	M0	30	Recommended, un
Male	16 years	T1	N0	M0	0	None/Unknown
Male	17 years	Blank(s)	Blank(s)	Blank(s)	30	None/Unknown
Male	17 years	Blank(s)	Blank(s)	Blank(s)	0	Beam radiation
Female	07 years	Blank(s)	Blank(s)	Blank(s)	0	Beam radiation
Female	60 years	Blank(s)	Blank(s)	Blank(s)	0	None/Unknown
Female	12 years	Blank(s)	Blank(s)	Blank(s)	30	None/Unknown
Male	12 years	Blank(s)	Blank(s)	Blank(s)	0	Beam radiation
Male	11 years	Blank(s)	Blank(s)	Blank(s)	0	Beam radiation
Female	28 years	Blank(s)	Blank(s)	Blank(s)	30	Beam radiation
Male	12 years	Blank(s)	Blank(s)	Blank(s)	41	None/Unknown
Male	13 years	Blank(s)	Blank(s)	Blank(s)	30	None/Unknown
Female	15 years	Blank(s)	Blank(s)	Blank(s)	30	Beam radiation
Male	15 years	Blank(s)	Blank(s)	Blank(s)	30	None/Unknown
Male	41 years	Blank(s)	Blank(s)	Blank(s)	0	Beam radiation
Male	18 years	Blank(s)	Blank(s)	Blank(s)	30	Beam radiation

Male	02 years	Blank(s)	Blank(s)	Blank(s)	41	None/Unknown	
Female	29 years	Blank(s)	Blank(s)	Blank(s)	0	Beam radiation	
Male	10 years	Blank(s)	Blank(s)	Blank(s)	30	None/Unknown	
Female	13 years	Blank(s)	Blank(s)	Blank(s)	30	None/Unknown	
Female	06 years	Blank(s)	Blank(s)	Blank(s)	30	None/Unknown	
Female	14 years	Blank(s)	Blank(s)	Blank(s)	0	Beam radiation	
Male	30 years	Blank(s)	Blank(s)	Blank(s)	0	Beam radiation	
Male	14 years	Blank(s)	Blank(s)	Blank(s)	0	Beam radiation	
Male	13 years	Blank(s)	Blank(s)	Blank(s)	41	None/Unknown	
Female	18 years	Blank(s)	Blank(s)	Blank(s)	30	Beam radiation	
Female	44 years	Blank(s)	Blank(s)	Blank(s)	25	Beam radiation	
Female	12 years	Blank(s)	Blank(s)	Blank(s)	30	None/Unknown	
Male	25 years	Blank(s)	Blank(s)	Blank(s)	30	None/Unknown	
Male	16 years	Blank(s)	Blank(s)	Blank(s)	25	None/Unknown	
Male	15 years	Blank(s)	Blank(s)	Blank(s)	26	Beam radiation	
Male	05 years	Blank(s)	Blank(s)	Blank(s)	0	None/Unknown	
Male	20 years	Blank(s)	Blank(s)	Blank(s)	0	None/Unknown	
Female	16 years	Blank(s)	Blank(s)	Blank(s)	0	Beam radiation	
Female	16 years	Blank(s)	Blank(s)	Blank(s)	0	Beam radiation	
Male	63 years	Blank(s)	Blank(s)	Blank(s)	30	Beam radiation	
Male	12 years	Blank(s)	Blank(s)	Blank(s)	0	Beam radiation	
Female	17 years	Blank(s)	Blank(s)	Blank(s)	0	Beam radiation	
Male	38 years	Blank(s)	Blank(s)	Blank(s)	90	None/Unknown	
Female	16 years	Blank(s)	Blank(s)	Blank(s)	25	Beam radiation	
Male	20 years	Blank(s)	Blank(s)	Blank(s)	0	None/Unknown	
Male	39 years	Blank(s)	Blank(s)	Blank(s)	90	None/Unknown	
Female	02 years	Blank(s)	Blank(s)	Blank(s)	0	Beam radiation	
Male	05 years	Blank(s)	Blank(s)	Blank(s)	30	None/Unknown	
Male	06 years	Blank(s)	Blank(s)	Blank(s)	30	None/Unknown	
Male	16 years	Blank(s)	Blank(s)	Blank(s)	0	Beam radiation	
Male	16 years	T3	NX	M1b	0	None/Unknown	
Male	17 years	T2	N0	M0	0	None/Unknown	
Female	14 years	T1	N0	M1a	0	Beam radiation	
Female	15 years	TX	N0	M1b	0	Beam radiation	
Male	25 years	T1	N0	M0	30	Beam radiation	
Female	51 years	TX	N0	M0	0	Beam radiation	
Female	49 years	T1	N0	M0	26	None/Unknown	
Female	17 years	TX	N0	M0	26	Beam radiation	
Male	12 years	TX	N0	M0	30	None/Unknown	
Female	27 years	T2	N0	M0	30	None/Unknown	
Female	45 years	T2	N0	M0	30	Beam radiation	
Male	10 years	T2	N0	M1b	30	Beam radiation	
Male	12 years	T1	NX	M1b	0	Beam radiation	
Male	12 years	TX	N0	M0	0	Beam radiation	
Female	02 years	T2	N0	M1a	0	Beam radiation	
Male	31 years	T2	N0	M0	30	Beam radiation	
Male	24 years	T2	N0	M0	0	Beam radiation	
Female	14 years	T2	N0	M0	30	None/Unknown	
Male	18 years	T2	N0	M0	26	None/Unknown	
Male	67 years	T3	N0	M1b	0	None/Unknown	
Male	19 years	T1	N0	M0	0	Beam radiation	
Male	39 years	T1	N0	M0	30	None/Unknown	
Female	13 years	T1	N0	M0	30	None/Unknown	
Male	58 years	TX	N0	M0	0	Beam radiation	
Female	12 years	T2	N0	M1b	0	Beam radiation	
Female	17 years	T2	N0	M1a	25	Radiation, NOS	me
Female	13 years	T2	N0	M0	0	Beam radiation	
Male	22 years	T2	N0	M0	0	Radiation, NOS	me

Female	12 years	Blank(s)	Blank(s)	Blank(s)	30	Combination of be
Male	25 years	Blank(s)	Blank(s)	Blank(s)	0	None/Unknown
Female	15 years	T1	N0	M1a	90	Beam radiation
Male	13 years	T2	N0	M1b	0	Beam radiation
Male	39 years	Blank(s)	Blank(s)	Blank(s)	0	Beam radiation
Female	08 years	Blank(s)	Blank(s)	Blank(s)	30	Radiation, NOS me
Male	09 years	T2	N0	M1b	30	None/Unknown
Male	14 years	Blank(s)	Blank(s)	Blank(s)	30	Beam radiation
Female	09 years	Blank(s)	Blank(s)	Blank(s)	25	None/Unknown
Female	15 years	Blank(s)	Blank(s)	Blank(s)	25	Beam radiation
Female	21 years	Blank(s)	Blank(s)	Blank(s)	0	None/Unknown
Male	29 years	Blank(s)	Blank(s)	Blank(s)	0	Beam radiation
Male	21 years	Blank(s)	Blank(s)	Blank(s)	30	None/Unknown
Male	18 years	Blank(s)	Blank(s)	Blank(s)	0	None/Unknown
Female	63 years	Blank(s)	Blank(s)	Blank(s)	0	Beam radiation
Male	24 years	Blank(s)	Blank(s)	Blank(s)	0	None/Unknown
Female	09 years	Blank(s)	Blank(s)	Blank(s)	30	None/Unknown
Male	06 years	Blank(s)	Blank(s)	Blank(s)	0	Beam radiation
Male	25 years	Blank(s)	Blank(s)	Blank(s)	54	None/Unknown
Male	47 years	T1	N0	M0	30	None/Unknown
Male	14 years	T1	N0	M0	25	None/Unknown
Male	06 years	T2	N0	M0	26	Beam radiation
Male	21 years	T2	N1	M0	30	None/Unknown
Male	02 years	T1	N0	M0	30	None/Unknown
Male	21 years	TX	NX	M0	25	Beam radiation
Male	20 years	T1	N0	M0	30	Beam radiation
Female	39 years	Blank(s)	Blank(s)	Blank(s)	0	Beam radiation
Male	18 years	Blank(s)	Blank(s)	Blank(s)	0	None/Unknown
Male	16 years	Blank(s)	Blank(s)	Blank(s)	0	None/Unknown
Female	04 years	Blank(s)	Blank(s)	Blank(s)	0	Beam radiation
Male	16 years	Blank(s)	Blank(s)	Blank(s)	0	None/Unknown
Male	25 years	Blank(s)	Blank(s)	Blank(s)	0	Beam radiation
Male	10 years	Blank(s)	Blank(s)	Blank(s)	53	Beam radiation
Male	03 years	T2	N0	M0	30	None/Unknown
Female	16 years	Blank(s)	Blank(s)	Blank(s)	25	Beam radiation
Male	17 years	T3	NX	M1b	0	Beam radiation
Male	41 years	T2	N0	M0	0	Beam radiation
Male	19 years	T2	N0	M0	0	None/Unknown
Female	15 years	T1	N1	M0	0	Beam radiation
Male	19 years	T2	N0	M0	26	None/Unknown
Male	19 years	T2	N0	M0	30	None/Unknown
Male	23 years	T2	NX	M1b	25	Beam radiation
Female	70 years	Blank(s)	Blank(s)	Blank(s)	0	None/Unknown
Male	16 years	TX	NX	M1a	25	Beam radiation
Female	17 years	T2	N0	M0	0	Beam radiation
Male	13 years	T2	N0	M0	30	None/Unknown
Male	22 years	T1	N0	M1a	30	Beam radiation
Female	08 years	T2	N0	M0	26	None/Unknown
Male	04 years	T1	N0	M0	0	None/Unknown
Male	24 years	T2	NX	M1b	0	Beam radiation
Male	18 years	T2	NX	M0	26	None/Unknown
Male	03 years	TX	N0	M0	30	None/Unknown
Female	21 years	T2	N0	M0	30	Beam radiation
Male	12 years	T1	N0	M0	26	None/Unknown
Male	13 years	TX	N1	M1a	0	Beam radiation
Male	01 years	TX	N0	M0	0	Beam radiation
Male	09 years	T1	N0	M0	25	Beam radiation
Female	09 years	TX	N0	M0	30	None/Unknown

Male	21 years	T2	N0	M1b	25	Beam radiation
Male	15 years	T2	N1	M1b	0	Beam radiation
Female	72 years	T1	N0	M1NOS	0	None/Unknown
Male	35 years	TX	NX	M1a	0	None/Unknown
Female	20 years	T1	N0	M1b	0	Beam radiation
Male	02 years	T1	N0	M0	30	None/Unknown
Female	29 years	TX	NX	MX	90	Beam radiation
Female	51 years	TX	N0	MX	30	Beam radiation
Male	12 years	T1	N0	M0	0	Beam radiation
Male	12 years	T2	N0	M1a	0	Beam radiation
Male	24 years	T2	N1	M1b	0	Beam radiation
Male	12 years	T2	N0	M1a	25	Beam radiation
Male	10 years	T1	N0	M0	30	Beam radiation
Male	14 years	TX	N0	M0	0	Beam radiation
Male	09 years	T1	N0	M0	30	None/Unknown
Female	15 years	Blank(s)	Blank(s)	Blank(s)	0	Beam radiation
Male	05 years	Blank(s)	Blank(s)	Blank(s)	0	Beam radiation
Male	23 years	Blank(s)	Blank(s)	Blank(s)	30	None/Unknown
Female	57 years	Blank(s)	Blank(s)	Blank(s)	0	Beam radiation
Female	55 years	Blank(s)	Blank(s)	Blank(s)	26	Beam radiation
Male	15 years	Blank(s)	Blank(s)	Blank(s)	30	None/Unknown
Male	79 years	Blank(s)	Blank(s)	Blank(s)	0	Beam radiation
Male	21 years	Blank(s)	Blank(s)	Blank(s)	0	Beam radiation
Female	11 years	Blank(s)	Blank(s)	Blank(s)	0	Beam radiation
Male	12 years	Blank(s)	Blank(s)	Blank(s)	0	Beam radiation
Male	29 years	Blank(s)	Blank(s)	Blank(s)	30	None/Unknown
Male	08 years	Blank(s)	Blank(s)	Blank(s)	25	None/Unknown
Female	20 years	Blank(s)	Blank(s)	Blank(s)	0	None/Unknown
Male	18 years	Blank(s)	Blank(s)	Blank(s)	0	Recommended, un
Male	13 years	TX	N0	M0	0	None/Unknown
Female	17 years	Blank(s)	Blank(s)	Blank(s)	40	Beam radiation
Male	11 years	Blank(s)	Blank(s)	Blank(s)	90	Beam radiation
Male	32 years	Blank(s)	Blank(s)	Blank(s)	30	Beam radiation
Male	38 years	Blank(s)	Blank(s)	Blank(s)	0	Beam radiation
Male	14 years	Blank(s)	Blank(s)	Blank(s)	41	Beam radiation
Male	35 years	Blank(s)	Blank(s)	Blank(s)	30	Beam radiation
Male	23 years	Blank(s)	Blank(s)	Blank(s)	90	Recommended, un
Male	03 years	Blank(s)	Blank(s)	Blank(s)	30	None/Unknown
Female	24 years	Blank(s)	Blank(s)	Blank(s)	19	Beam radiation
Male	24 years	Blank(s)	Blank(s)	Blank(s)	0	Beam radiation
Male	04 years	Blank(s)	Blank(s)	Blank(s)	30	None/Unknown
Male	04 years	Blank(s)	Blank(s)	Blank(s)	0	None/Unknown
Female	23 years	Blank(s)	Blank(s)	Blank(s)	0	None/Unknown
Male	15 years	Blank(s)	Blank(s)	Blank(s)	19	Beam radiation
Female	15 years	Blank(s)	Blank(s)	Blank(s)	30	None/Unknown
Male	12 years	Blank(s)	Blank(s)	Blank(s)	41	Beam radiation
Female	63 years	T0	N1	M0	0	None/Unknown
Female	17 years	Blank(s)	Blank(s)	Blank(s)	0	None/Unknown
Male	46 years	Blank(s)	Blank(s)	Blank(s)	30	Beam radiation
Male	21 years	Blank(s)	Blank(s)	Blank(s)	41	Beam radiation
Female	17 years	Blank(s)	Blank(s)	Blank(s)	19	Beam radiation
Male	17 years	Blank(s)	Blank(s)	Blank(s)	0	Beam radiation
Female	11 years	Blank(s)	Blank(s)	Blank(s)	0	Beam radiation
Male	13 years	Blank(s)	Blank(s)	Blank(s)	30	Beam radiation
Male	45 years	Blank(s)	Blank(s)	Blank(s)	0	None/Unknown
Male	19 years	Blank(s)	Blank(s)	Blank(s)	0	None/Unknown
Male	46 years	Blank(s)	Blank(s)	Blank(s)	0	Beam radiation
Male	34 years	Blank(s)	Blank(s)	Blank(s)	30	None/Unknown

Male	18 years	Blank(s)	Blank(s)	Blank(s)	0	Beam radiation
Male	28 years	Blank(s)	Blank(s)	Blank(s)	90	Beam radiation
Female	48 years	Blank(s)	Blank(s)	Blank(s)	40	None/Unknown
Female	51 years	Blank(s)	Blank(s)	Blank(s)	90	None/Unknown
Female	04 years	Blank(s)	Blank(s)	Blank(s)	30	Beam radiation
Male	19 years	Blank(s)	Blank(s)	Blank(s)	0	None/Unknown
Female	30 years	T2	N0	M0	51	Beam radiation
Male	21 years	T2	N0	M0	26	Beam radiation
Male	19 years	T2	N0	M0	30	Beam radiation
Male	12 years	T1	N0	M1b	0	None/Unknown
Male	64 years	T1	NX	M0	0	Beam radiation
Male	24 years	T2	NX	M0	0	Beam radiation
Female	05 years	T2	N0	M0	30	Beam radiation
Female	24 years	TX	N1	M0	0	Beam radiation
Female	17 years	TX	N0	M0	0	Beam radiation
Female	17 years	T2	N0	M0	30	Beam radiation
Male	12 years	TX	N0	M0	30	Beam radiation
Male	16 years	T2	N0	M1a	0	Beam radiation
Male	66 years	Blank(s)	Blank(s)	Blank(s)	25	None/Unknown
Male	76 years	TX	N0	M0	25	None/Unknown
Female	22 years	T1	N0	M0	30	Beam radiation
Male	17 years	T1	N0	M1a	26	None/Unknown
Male	28 years	T1	N0	M0	30	Beam radiation
Male	10 years	TX	N0	M0	0	Beam radiation
Female	49 years	TX	NX	M1a	0	None/Unknown
Male	07 years	TX	NX	M0	30	None/Unknown
Female	19 years	T1	N0	M0	30	Beam radiation
Female	16 years	T2	N0	M0	30	Beam radiation
Male	12 years	T2	N1	M0	40	None/Unknown
Male	18 years	T2	N0	M0	0	Beam radiation
Female	47 years	T1	N0	M1b	25	Beam radiation
Female	23 years	TX	N0	M0	30	None/Unknown
Female	28 years	T2	N0	M1b	41	Beam radiation
Male	05 years	T1	N1	M0	25	Beam radiation
Male	12 years	T2	N0	M0	30	None/Unknown
Male	02 years	T2	N0	M0	42	None/Unknown
Male	13 years	T2	N0	M0	0	None/Unknown
Female	12 years	TX	N0	M0	54	None/Unknown
Male	16 years	TX	NX	MX	0	None/Unknown
Male	04 years	TX	N0	M0	30	None/Unknown
Male	04 years	T1	N0	M0	26	None/Unknown
Male	07 years	T2	N1	M0	53	Beam radiation
Male	04 years	T1	N0	M0	0	None/Unknown
Male	14 years	TX	N0	M1b	25	Beam radiation
Male	42 years	TX	NX	M1b	0	None/Unknown
Male	22 years	T2	N0	M0	30	Beam radiation
Male	18 years	T1	N0	M0	26	Beam radiation
Male	13 years	TX	N0	M0	42	None/Unknown
Male	14 years	T2	N0	M1b	0	Beam radiation
Male	44 years	TX	N0	M1b	0	Beam radiation
Male	19 years	T1	N0	M0	30	Beam radiation
Female	10 years	TX	N0	M1a	30	None/Unknown
Male	32 years	T2	N0	MX	90	None/Unknown
Male	19 years	T2	N1	M0	41	None/Unknown
Male	44 years	TX	N0	M1b	0	None/Unknown
Female	08 years	TX	N0	M0	30	Beam radiation
Male	28 years	T2	NX	M1b	0	Beam radiation
Male	14 years	TX	NX	MX	0	None/Unknown

Male	19 years	T2	N0	M0	90	Beam radiation	
Female	12 years	TX	N0	M1b	54	None/Unknown	
Female	09 years	T2	N1	M1b	30	None/Unknown	
Male	17 years	T2	N0	M0	41	None/Unknown	
Female	14 years	T2	N0	M0	41	None/Unknown	
Female	12 years	T1	N0	M0	30	None/Unknown	
Female	46 years	TX	N0	M0	30	Radiation, NOS	me
Male	25 years	T1	N0	M0	25	Beam radiation	
Male	71 years	TX	NX	MX	0	None/Unknown	
Female	14 years	T1	N0	M0	0	None/Unknown	
Male	26 years	Blank(s)	Blank(s)	Blank(s)	90	Radiation, NOS	me
Male	13 years	TX	N0	M0	30	Beam radiation	
Male	31 years	TX	N0	M0	30	Beam radiation	
Male	09 years	T2	N0	M0	0	Beam radiation	
Female	18 years	T1	N0	M0	26	None/Unknown	
Male	16 years	T3	N0	M1b	0	None/Unknown	
Male	15 years	T1	N0	M0	41	None/Unknown	
Female	17 years	T2	N0	M1a	30	Beam radiation	
Male	14 years	T2	N0	M0	0	Beam radiation	
Male	25 years	T2	N0	M1b	0	Beam radiation	
Male	27 years	TX	N0	M1a	0	Beam radiation	
Female	13 years	T1	N0	M0	30	None/Unknown	
Female	20 years	T1	N0	M0	41	None/Unknown	
Female	05 years	TX	N0	M0	26	Beam radiation	
Male	10 years	T1	N0	M0	26	Beam radiation	
Male	49 years	Blank(s)	Blank(s)	Blank(s)	99	None/Unknown	
Female	24 years	T1	N0	M0	30	None/Unknown	
Female	26 years	TX	N0	M0	25	Beam radiation	
Male	44 years	TX	N1	M1b	0	None/Unknown	
Female	28 years	T2	N0	M1a	0	None/Unknown	
Male	03 years	TX	N0	M0	25	Refused (1988+)	
Male	21 years	T2	N0	M0	0	None/Unknown	
Male	10 years	T1	N0	M0	30	None/Unknown	
Female	56 years	T3	N0	M0	0	None/Unknown	
Male	34 years	T1	N0	M0	25	None/Unknown	
Male	21 years	TX	N0	M1b	0	Beam radiation	
Female	13 years	T2	N0	M0	30	Beam radiation	
Male	18 years	T2	N0	M1b	0	None/Unknown	
Female	35 years	T2	N0	M1b	0	None/Unknown	
Female	03 years	TX	N0	M1a	30	None/Unknown	
Male	28 years	TX	N0	M0	0	None/Unknown	
Female	63 years	TX	N1	MX	0	None/Unknown	
Male	25 years	TX	NX	MX	99	None/Unknown	
Female	03 years	T1	N0	M0	30	None/Unknown	
Female	10 years	T1	N1	M0	0	None/Unknown	
Male	17 years	TX	NX	M1b	0	Beam radiation	
Female	18 years	T1	N0	M0	0	Beam radiation	
Male	26 years	TX	N0	M0	90	Beam radiation	
Male	46 years	T3	N0	M1b	0	None/Unknown	
Female	10 years	T1	N0	M0	30	None/Unknown	
Male	50 years	T2	N0	M0	41	None/Unknown	
Female	60 years	Blank(s)	Blank(s)	Blank(s)	30	None/Unknown	
Male	20 years	T2	N0	M1b	30	Beam radiation	
Female	03 years	T1	N0	M0	41	None/Unknown	
Female	06 years	TX	N0	M0	53	None/Unknown	
Female	15 years	T1	N1	M0	41	Beam radiation	
Male	12 years	T1	N0	M0	25	None/Unknown	
Male	12 years	T2	N0	M0	30	None/Unknown	

Male	14 years	TX	N0	M1a	41	Beam radiation
Male	78 years	T0	NX	M1b	0	None/Unknown
Male	10 years	T1	N0	M1b	30	Beam radiation
Male	07 years	TX	N0	M0	0	Beam radiation
Female	26 years	T2	N0	M0	0	None/Unknown
Female	49 years	TX	NX	M0	0	None/Unknown
Female	23 years	T1	N0	M0	0	None/Unknown
Male	15 years	T2	N0	M0	30	None/Unknown
Male	04 years	TX	N0	M1NOS	0	None/Unknown
Male	09 years	T2	N0	M0	30	None/Unknown
Female	21 years	TX	N0	M0	25	Recommended, un
Female	12 years	T1	N0	M0	25	None/Unknown
Female	14 years	T2	N0	M0	30	None/Unknown
Male	01 years	T1	N0	M0	30	None/Unknown
Male	11 years	T1	N0	M0	30	None/Unknown
Male	24 years	T2	N1	M1b	0	None/Unknown
Female	07 years	T2	N0	M0	0	Beam radiation
Male	07 years	T1	N0	M0	30	None/Unknown
Female	87 years	TX	NX	MX	0	None/Unknown
Female	98 years	TX	NX	M0	0	None/Unknown
Male	41 years	Blank(s)	Blank(s)	Blank(s)	0	None/Unknown
Male	09 years	Blank(s)	Blank(s)	Blank(s)	25	None/Unknown
Female	14 years	Blank(s)	Blank(s)	Blank(s)	30	None/Unknown
Male	05 years	Blank(s)	Blank(s)	Blank(s)	25	None/Unknown
Male	23 years	Blank(s)	Blank(s)	Blank(s)	0	Beam radiation
Male	13 years	Blank(s)	Blank(s)	Blank(s)	30	None/Unknown
Male	10 years	Blank(s)	Blank(s)	Blank(s)	26	None/Unknown
Male	14 years	Blank(s)	Blank(s)	Blank(s)	0	None/Unknown
Male	21 years	Blank(s)	Blank(s)	Blank(s)	0	None/Unknown
Male	17 years	Blank(s)	Blank(s)	Blank(s)	0	Beam radiation
Male	20 years	Blank(s)	Blank(s)	Blank(s)	0	Beam radiation
Female	34 years	Blank(s)	Blank(s)	Blank(s)	0	Beam radiation
Male	22 years	Blank(s)	Blank(s)	Blank(s)	0	Beam radiation
Female	12 years	Blank(s)	Blank(s)	Blank(s)	0	Beam radiation
Female	10 years	Blank(s)	Blank(s)	Blank(s)	30	None/Unknown
Male	69 years	Blank(s)	Blank(s)	Blank(s)	0	None/Unknown
Female	29 years	Blank(s)	Blank(s)	Blank(s)	0	Beam radiation
Male	36 years	Blank(s)	Blank(s)	Blank(s)	25	None/Unknown
Male	65 years	Blank(s)	Blank(s)	Blank(s)	30	Beam radiation
Male	21 years	Blank(s)	Blank(s)	Blank(s)	0	None/Unknown
Male	17 years	Blank(s)	Blank(s)	Blank(s)	30	None/Unknown
Male	16 years	Blank(s)	Blank(s)	Blank(s)	0	Beam radiation
Male	49 years	Blank(s)	Blank(s)	Blank(s)	25	Beam radiation
Male	15 years	Blank(s)	Blank(s)	Blank(s)	0	Beam radiation
Male	09 years	Blank(s)	Blank(s)	Blank(s)	30	Beam radiation
Male	14 years	Blank(s)	Blank(s)	Blank(s)	0	Beam radiation
Male	17 years	Blank(s)	Blank(s)	Blank(s)	0	Beam radiation
Female	15 years	Blank(s)	Blank(s)	Blank(s)	30	Beam radiation
Male	10 years	Blank(s)	Blank(s)	Blank(s)	25	None/Unknown
Female	42 years	Blank(s)	Blank(s)	Blank(s)	30	None/Unknown
Male	14 years	Blank(s)	Blank(s)	Blank(s)	0	Beam radiation
Male	30 years	Blank(s)	Blank(s)	Blank(s)	30	Beam radiation
Female	14 years	Blank(s)	Blank(s)	Blank(s)	30	None/Unknown
Female	38 years	Blank(s)	Blank(s)	Blank(s)	0	Recommended, un
Male	10 years	Blank(s)	Blank(s)	Blank(s)	25	Beam radiation
Female	11 years	Blank(s)	Blank(s)	Blank(s)	0	Beam radiation
Male	27 years	Blank(s)	Blank(s)	Blank(s)	30	Beam radiation
Male	23 years	Blank(s)	Blank(s)	Blank(s)	42	Beam radiation

Male	17 years	Blank(s)	Blank(s)	Blank(s)	30	None/Unknown
Female	40 years	Blank(s)	Blank(s)	Blank(s)	0	None/Unknown
Female	37 years	Blank(s)	Blank(s)	Blank(s)	99	None/Unknown
Female	23 years	Blank(s)	Blank(s)	Blank(s)	0	None/Unknown
Male	31 years	T1	N0	M0	30	None/Unknown
Male	15 years	T2	N1	M1b	25	Beam radiation
Male	18 years	Blank(s)	Blank(s)	Blank(s)	30	None/Unknown
Female	10 years	Blank(s)	Blank(s)	Blank(s)	0	Beam radiation
Male	09 years	Blank(s)	Blank(s)	Blank(s)	0	Beam radiation
Female	20 years	Blank(s)	Blank(s)	Blank(s)	90	Beam radiation
Male	17 years	Blank(s)	Blank(s)	Blank(s)	30	Beam radiation
Male	54 years	Blank(s)	Blank(s)	Blank(s)	0	Recommended, un
Female	02 years	Blank(s)	Blank(s)	Blank(s)	0	None/Unknown
Female	13 years	Blank(s)	Blank(s)	Blank(s)	41	None/Unknown
Female	11 years	Blank(s)	Blank(s)	Blank(s)	30	Recommended, un
Male	03 years	Blank(s)	Blank(s)	Blank(s)	0	None/Unknown
Female	16 years	Blank(s)	Blank(s)	Blank(s)	0	Beam radiation
Female	06 years	Blank(s)	Blank(s)	Blank(s)	26	Beam radiation
Female	14 years	Blank(s)	Blank(s)	Blank(s)	0	Beam radiation
Female	47 years	T2	N1	M0	42	None/Unknown
Male	37 years	Blank(s)	Blank(s)	Blank(s)	30	Beam radiation
Male	19 years	Blank(s)	Blank(s)	Blank(s)	0	None/Unknown
Male	19 years	Blank(s)	Blank(s)	Blank(s)	30	Beam radiation
Female	08 years	Blank(s)	Blank(s)	Blank(s)	0	None/Unknown
Female	45 years	Blank(s)	Blank(s)	Blank(s)	0	None/Unknown
Female	07 years	Blank(s)	Blank(s)	Blank(s)	25	Beam radiation
Male	16 years	Blank(s)	Blank(s)	Blank(s)	0	None/Unknown
Male	53 years	Blank(s)	Blank(s)	Blank(s)	25	Beam radiation
Male	24 years	T1	N0	M0	26	Recommended, un
Male	15 years	Blank(s)	Blank(s)	Blank(s)	30	None/Unknown
Female	16 years	Blank(s)	Blank(s)	Blank(s)	0	None/Unknown
Male	12 years	Blank(s)	Blank(s)	Blank(s)	25	None/Unknown
Male	11 years	Blank(s)	Blank(s)	Blank(s)	26	Beam radiation
Female	09 years	Blank(s)	Blank(s)	Blank(s)	41	None/Unknown
Male	10 years	Blank(s)	Blank(s)	Blank(s)	90	Beam radiation
Male	14 years	Blank(s)	Blank(s)	Blank(s)	0	Beam radiation
Male	07 years	Blank(s)	Blank(s)	Blank(s)	30	None/Unknown
Male	13 years	Blank(s)	Blank(s)	Blank(s)	0	None/Unknown
Female	46 years	T1	N0	M0	53	Beam radiation
Male	11 years	Blank(s)	Blank(s)	Blank(s)	30	None/Unknown
Male	12 years	Blank(s)	Blank(s)	Blank(s)	0	Beam radiation
Female	22 years	Blank(s)	Blank(s)	Blank(s)	19	Beam radiation
Male	20 years	T1	N0	M0	25	Beam radiation
Male	18 years	Blank(s)	Blank(s)	Blank(s)	0	Beam radiation
Female	43 years	Blank(s)	Blank(s)	Blank(s)	0	Beam radiation
Male	23 years	Blank(s)	Blank(s)	Blank(s)	0	None/Unknown
Female	28 years	Blank(s)	Blank(s)	Blank(s)	0	Beam radiation
Male	42 years	Blank(s)	Blank(s)	Blank(s)	30	Beam radiation
Female	64 years	T1	N0	M0	0	Beam radiation
Female	52 years	Blank(s)	Blank(s)	Blank(s)	0	Beam radiation
Male	32 years	Blank(s)	Blank(s)	Blank(s)	0	None/Unknown
Male	19 years	Blank(s)	Blank(s)	Blank(s)	0	Beam radiation
Female	12 years	Blank(s)	Blank(s)	Blank(s)	26	Beam radiation
Male	16 years	Blank(s)	Blank(s)	Blank(s)	0	Beam radiation
Male	14 years	Blank(s)	Blank(s)	Blank(s)	30	None/Unknown
Female	12 years	Blank(s)	Blank(s)	Blank(s)	0	Beam radiation
Male	16 years	Blank(s)	Blank(s)	Blank(s)	0	Beam radiation
Male	19 years	Blank(s)	Blank(s)	Blank(s)	25	Beam radiation

Female	20 years	Blank(s)	Blank(s)	Blank(s)	26	Beam radiation
Male	24 years	Blank(s)	Blank(s)	Blank(s)	0	Beam radiation
Male	28 years	Blank(s)	Blank(s)	Blank(s)	90	Beam radiation
Female	10 years	Blank(s)	Blank(s)	Blank(s)	25	Beam radiation
Male	21 years	Blank(s)	Blank(s)	Blank(s)	90	Beam radiation
Female	45 years	Blank(s)	Blank(s)	Blank(s)	30	Beam radiation
Male	16 years	Blank(s)	Blank(s)	Blank(s)	40	None/Unknown
Male	14 years	Blank(s)	Blank(s)	Blank(s)	26	None/Unknown
Male	15 years	Blank(s)	Blank(s)	Blank(s)	30	Beam radiation
Female	13 years	Blank(s)	Blank(s)	Blank(s)	25	Beam radiation
Female	17 years	Blank(s)	Blank(s)	Blank(s)	30	None/Unknown
Male	27 years	Blank(s)	Blank(s)	Blank(s)	0	None/Unknown
Male	19 years	Blank(s)	Blank(s)	Blank(s)	0	None/Unknown
Male	12 years	Blank(s)	Blank(s)	Blank(s)	30	None/Unknown
Male	12 years	Blank(s)	Blank(s)	Blank(s)	25	None/Unknown
Male	03 years	Blank(s)	Blank(s)	Blank(s)	90	None/Unknown
Female	17 years	Blank(s)	Blank(s)	Blank(s)	53	None/Unknown
Female	36 years	Blank(s)	Blank(s)	Blank(s)	26	None/Unknown
Male	14 years	Blank(s)	Blank(s)	Blank(s)	53	None/Unknown
Female	07 years	Blank(s)	Blank(s)	Blank(s)	30	Beam radiation
Male	13 years	Blank(s)	Blank(s)	Blank(s)	0	Beam radiation
Female	07 years	Blank(s)	Blank(s)	Blank(s)	0	None/Unknown
Male	54 years	Blank(s)	Blank(s)	Blank(s)	0	Beam radiation
Female	39 years	Blank(s)	Blank(s)	Blank(s)	0	Recommended, un
Male	55 years	T1	N0	M0	0	None/Unknown
Male	21 years	Blank(s)	Blank(s)	Blank(s)	41	None/Unknown
Female	14 years	Blank(s)	Blank(s)	Blank(s)	0	Beam radiation
Female	06 years	Blank(s)	Blank(s)	Blank(s)	0	Beam radiation
Female	11 years	Blank(s)	Blank(s)	Blank(s)	30	None/Unknown
Female	30 years	Blank(s)	Blank(s)	Blank(s)	30	Beam radiation
Male	02 years	Blank(s)	Blank(s)	Blank(s)	0	Beam radiation
Female	30 years	Blank(s)	Blank(s)	Blank(s)	0	Beam radiation
Male	24 years	Blank(s)	Blank(s)	Blank(s)	0	None/Unknown
Female	12 years	Blank(s)	Blank(s)	Blank(s)	0	Beam radiation
Male	01 years	Blank(s)	Blank(s)	Blank(s)	30	None/Unknown
Female	64 years	Blank(s)	Blank(s)	Blank(s)	90	None/Unknown
Male	35 years	Blank(s)	Blank(s)	Blank(s)	30	None/Unknown
Female	22 years	Blank(s)	Blank(s)	Blank(s)	0	Beam radiation
Male	19 years	Blank(s)	Blank(s)	Blank(s)	0	Refused (1988+)
Male	13 years	Blank(s)	Blank(s)	Blank(s)	0	Beam radiation
Female	04 years	T1	N0	M0	0	Beam radiation
Male	53 years	Blank(s)	Blank(s)	Blank(s)	0	None/Unknown
Male	12 years	Blank(s)	Blank(s)	Blank(s)	30	None/Unknown
Female	06 years	Blank(s)	Blank(s)	Blank(s)	30	None/Unknown
Male	11 years	Blank(s)	Blank(s)	Blank(s)	53	None/Unknown
Male	20 years	T2	N0	M0	30	None/Unknown
Male	09 years	T2	N0	M0	0	None/Unknown
Male	16 years	TX	NX	MX	30	Beam radiation
Male	17 years	TX	N0	M0	30	Beam radiation
Male	23 years	TX	N0	M0	0	None/Unknown
Male	15 years	T2	N0	M1a	25	Beam radiation
Male	30 years	Blank(s)	Blank(s)	Blank(s)	0	None/Unknown
Female	28 years	T2	NX	M1b	41	Beam radiation
Female	04 years	T1	N0	M0	26	Beam radiation
Female	04 years	T2	N0	M0	26	None/Unknown
Male	19 years	TX	N0	M1b	0	None/Unknown
Male	13 years	T1	NX	M1a	0	Beam radiation
Female	00 years	T1	N0	M0	26	Beam radiation

Male	12 years	TX	N0	M0	0	None/Unknown
Male	15 years	TX	N0	M0	0	None/Unknown
Female	15 years	TX	N0	M0	0	Beam radiation
Male	16 years	T2	N0	M1b	30	Beam radiation
Male	16 years	T1	N0	M0	30	None/Unknown
Female	07 years	T1	N0	M0	26	Beam radiation
Male	15 years	T2	N1	M1a	0	None/Unknown
Male	01 years	T1	NX	M0	30	None/Unknown
Male	17 years	T3	N0	M1NOS	0	None/Unknown
Female	16 years	TX	N0	M0	30	None/Unknown
Male	16 years	T3	N1	M1b	30	None/Unknown
Male	12 years	T1	N0	M0	0	Beam radiation
Female	14 years	T1	N0	M1b	30	Beam radiation
Male	22 years	T1	N0	M0	30	None/Unknown
Male	13 years	TX	N0	M0	26	Beam radiation
Male	15 years	TX	N0	M0	0	Beam radiation
Female	24 years	T1	N0	M0	30	None/Unknown
Female	39 years	TX	N0	M0	26	Beam radiation
Male	01 years	T1	N0	M0	0	None/Unknown
Male	16 years	TX	NX	M1b	0	None/Unknown
Female	08 years	TX	N0	M1a	30	None/Unknown
Male	18 years	TX	N0	M0	0	None/Unknown
Female	73 years	T2	N0	M0	30	Beam radiation
Male	20 years	T2	NX	M1a	0	None/Unknown
Female	12 years	TX	N0	M1b	26	Beam radiation
Female	12 years	TX	N0	M0	30	None/Unknown
Female	19 years	T3	N1	M1b	0	Beam radiation
Female	15 years	TX	N0	M0	30	Beam radiation
Male	22 years	T2	N0	M0	30	None/Unknown
Male	24 years	Blank(s)	Blank(s)	Blank(s)	99	None/Unknown
Male	14 years	T1	N0	M0	30	None/Unknown
Male	09 years	Blank(s)	Blank(s)	Blank(s)	0	None/Unknown
Male	19 years	T3	N0	M1b	0	None/Unknown
Male	37 years	T3	N0	M1b	0	None/Unknown
Male	18 years	TX	N0	M1b	0	None/Unknown
Male	18 years	T2	N0	M0	54	Beam radiation
Male	06 years	TX	N1	M0	30	Beam radiation
Female	30 years	T3	N0	M1b	0	None/Unknown
Male	15 years	T2	N0	M0	42	None/Unknown
Male	08 years	T1	N0	M1a	0	Beam radiation
Male	11 years	T1	N1	M0	0	None/Unknown
Female	18 years	T3	NX	M1b	25	Beam radiation
Female	07 years	T1	N0	M0	30	None/Unknown
Female	35 years	T1	N0	M0	41	Beam radiation
Female	09 years	T3	NX	M1a	0	Beam radiation
Male	05 years	TX	N0	M1b	30	None/Unknown
Male	13 years	TX	N0	M0	0	None/Unknown
Male	31 years	T2	N0	M0	41	Beam radiation
Male	30 years	T2	N1	M1a	42	None/Unknown
Male	03 years	TX	NX	M0	25	Beam radiation
Male	13 years	T2	N0	M1b	30	Beam radiation
Male	19 years	T2	N0	M0	0	Beam radiation
Male	15 years	T2	N0	M0	25	None/Unknown
Female	09 years	T1	N0	M0	0	None/Unknown
Male	10 years	T1	N0	M0	30	None/Unknown
Male	11 years	T1	N0	M0	25	Beam radiation
Male	12 years	T2	N0	M0	0	Beam radiation
Male	22 years	T3	N0	M1b	0	Beam radiation

Female	22 years	TX	NX	M0	0	Beam radiation	
Male	17 years	T1	NX	M1b	25	Beam radiation	
Female	01 years	T1	N0	M0	30	Beam radiation	
Male	09 years	T1	N0	M1b	30	None/Unknown	
Male	22 years	T2	N0	M0	30	Beam radiation	
Female	14 years	T1	NX	M0	30	Beam radiation	
Male	15 years	T1	N0	M1b	25	Beam radiation	
Male	67 years	T1	N0	M0	41	None/Unknown	
Male	12 years	T2	N0	M0	41	None/Unknown	
Male	17 years	T2	N0	M0	30	None/Unknown	
Male	15 years	T2	N0	M0	30	None/Unknown	
Male	07 years	Blank(s)	Blank(s)	Blank(s)	30	None/Unknown	
Male	05 years	TX	N0	M1b	0	None/Unknown	
Female	10 years	T2	N0	M0	30	None/Unknown	
Male	34 years	T1	N0	M0	0	Beam radiation	
Female	50 years	T1	N0	M0	0	None/Unknown	
Male	09 years	TX	N1	M0	0	Beam radiation	
Male	11 years	T1	N0	M1a	0	Beam radiation	
Male	35 years	TX	NX	M1a	30	None/Unknown	
Male	09 years	Blank(s)	Blank(s)	Blank(s)	0	None/Unknown	
Female	04 years	T1	NX	M0	0	Beam radiation	
Male	00 years	TX	NX	M1b	0	None/Unknown	
Male	14 years	TX	N0	M0	26	Beam radiation	
Male	03 years	T1	N0	M0	0	None/Unknown	
Female	08 years	T1	N0	M0	0	None/Unknown	
Female	23 years	T1	N0	M0	0	Radiation, NOS	me
Female	17 years	TX	N0	M1a	0	Beam radiation	
Male	10 years	T2	N0	M1b	26	None/Unknown	
Male	21 years	T2	N1	M0	30	Beam radiation	
Female	08 years	T2	N0	M0	30	None/Unknown	
Female	13 years	T2	N0	M0	30	None/Unknown	
Female	16 years	T2	N0	M0	30	None/Unknown	
Male	10 years	T2	N0	M0	30	None/Unknown	
Female	12 years	T1	N0	M0	0	Beam radiation	
Male	11 years	T2	N0	M0	30	Beam radiation	
Male	18 years	T2	N0	M0	0	Beam radiation	
Male	06 years	T1	N0	M0	30	None/Unknown	
Male	14 years	T2	N0	M0	30	Beam radiation	
Female	11 years	T2	N0	M1b	30	Beam radiation	
Male	19 years	T1	N0	M0	0	Beam radiation	
Male	23 years	T2	N0	M1b	0	Beam radiation	
Male	52 years	T2	N0	M0	30	None/Unknown	
Male	15 years	TX	N0	M0	0	Beam radiation	
Male	19 years	T2	N0	M0	25	Beam radiation	
Female	18 years	TX	N0	M1b	0	Beam radiation	
Male	16 years	T2	N0	M0	30	None/Unknown	
Male	73 years	TX	NX	M1b	0	None/Unknown	
Female	19 years	T2	N0	M0	30	None/Unknown	
Male	16 years	T1	N0	M1b	30	Beam radiation	
Male	20 years	T1	N0	M1a	0	Beam radiation	
Female	19 years	T2	N0	M0	30	None/Unknown	
Male	56 years	TX	N0	M1b	15	None/Unknown	
Female	25 years	T2	N0	M1b	0	Beam radiation	
Female	06 years	T1	N0	M0	30	None/Unknown	
Female	15 years	T1	N0	M0	25	None/Unknown	
Male	27 years	T2	N0	M0	0	Radiation, NOS	me
Female	13 years	T1	N0	M0	25	None/Unknown	
Female	07 years	TX	N0	M0	30	None/Unknown	

Male	21 years	T3	N0	M1NOS	0	Beam radiation	
Male	06 years	T1	N0	M0	25	None/Unknown	
Female	55 years	T1	N0	M0	0	Beam radiation	
Female	21 years	TX	N0	M0	26	None/Unknown	
Male	38 years	T1	N0	M0	30	None/Unknown	
Female	38 years	T1	N0	M0	0	Beam radiation	
Female	27 years	TX	N0	M1NOS	90	None/Unknown	
Female	07 years	T2	N0	M1b	30	Beam radiation	
Female	07 years	T2	N0	M0	30	None/Unknown	
Male	10 years	T2	N0	M0	54	None/Unknown	
Female	18 years	T1	N0	M0	30	None/Unknown	
Female	18 years	TX	N0	M1b	0	Beam radiation	
Female	24 years	T1	N0	M0	0	Beam radiation	
Male	10 years	TX	N0	M1b	0	Beam radiation	
Male	33 years	T1	N0	M1b	0	Beam radiation	
Male	17 years	T2	N0	M1a	54	None/Unknown	
Male	18 years	T2	N0	M0	30	None/Unknown	
Male	04 years	TX	N0	M0	30	Beam radiation	
Male	18 years	T2	N0	M0	30	None/Unknown	
Male	21 years	T1	N0	M0	30	Beam radiation	
Male	22 years	T2	N0	M1NOS	0	Radiation, NOS	me
Male	72 years	T2	N0	M0	30	Beam radiation	
Male	32 years	T2	N0	M0	0	Beam radiation	
Male	08 years	T2	NX	M1a	26	None/Unknown	
Female	04 years	T2	N0	M1a	0	None/Unknown	
Male	28 years	TX	NX	MX	0	None/Unknown	
Male	17 years	T2	N0	M1a	25	Beam radiation	
Male	20 years	T2	N0	M1a	41	None/Unknown	
Male	17 years	T1	N0	M0	30	None/Unknown	
Male	28 years	T2	N0	M1a	0	Beam radiation	
Male	13 years	T2	N1	M0	30	Beam radiation	
Male	11 years	T1	N0	M1NOS	0	Beam radiation	
Male	20 years	T2	N0	M1b	0	Beam radiation	
Female	03 years	TX	N0	M0	0	Beam radiation	
Male	13 years	T2	N0	M1a	0	None/Unknown	
Male	15 years	TX	N0	M1a	0	Beam radiation	
Male	13 years	T1	N0	M1NOS	30	Beam radiation	
Male	11 years	TX	N0	M1b	0	None/Unknown	
Male	15 years	T2	N0	M1b	30	None/Unknown	
Male	20 years	TX	N0	M0	0	None/Unknown	
Female	11 years	T2	N1	M1a	30	None/Unknown	
Female	18 years	T1	N0	M0	0	Beam radiation	
Female	29 years	T2	N0	M1b	0	Beam radiation	
Male	15 years	T2	N1	M0	0	Beam radiation	
Female	32 years	T1	N1	M1b	25	Beam radiation	
Male	15 years	T3	NX	M1b	0	Beam radiation	
Male	15 years	T2	N0	M1NOS	0	Beam radiation	
Male	41 years	T1	N0	M0	26	Beam radiation	
Male	09 years	T1	N0	M0	25	None/Unknown	
Male	49 years	T2	N0	M1b	0	Beam radiation	
Male	20 years	T1	N0	M0	0	Beam radiation	
Male	47 years	TX	N0	M1b	0	None/Unknown	
Male	13 years	T2	N0	M0	0	Beam radiation	
Female	17 years	T2	N0	M1a	0	Beam radiation	
Female	10 years	T1	N0	M0	30	None/Unknown	
Male	06 years	TX	N0	M0	0	Beam radiation	
Male	16 years	T2	N1	M1b	0	None/Unknown	
Female	41 years	T1	N0	M0	26	Beam radiation	

Male	27 years	T1	N0	M0	26 Beam radiation
Female	19 years	T2	N0	M1a	0 None/Unknown
Male	24 years	T2	N0	M0	0 Beam radiation
Female	23 years	T1	N0	M0	30 Recommended, un
Female	21 years	T2	N0	M0	30 None/Unknown
Male	48 years	TX	N0	M1b	25 Recommended, un
Female	06 years	T1	N0	M0	0 Beam radiation
Male	48 years	TX	NX	MX	0 None/Unknown
Female	35 years	T1	N0	M0	25 Beam radiation
Male	12 years	T2	N0	M0	25 None/Unknown
Male	13 years	T2	N1	M1a	0 Beam radiation
Female	15 years	TX	NX	M0	0 None/Unknown
Female	14 years	T2	N1	M1b	30 Beam radiation
Female	11 years	T3	N0	M1NOS	0 Beam radiation
Female	13 years	T2	N0	M0	25 None/Unknown
Male	11 years	T1	N0	M0	0 Beam radiation
Male	67 years	TX	N0	M1a	0 Beam radiation
Female	16 years	T2	N0	M0	25 None/Unknown
Male	32 years	T1	N0	M0	26 Beam radiation
Female	07 years	T1	N0	M1a	30 None/Unknown
Male	05 years	T1	N0	M0	26 None/Unknown
Female	14 years	T1	N0	M0	30 None/Unknown
Male	15 years	T2	N0	M0	0 Beam radiation
Female	11 years	TX	N0	M0	30 None/Unknown
Male	21 years	TX	N0	M1a	0 None/Unknown
Male	56 years	T1	N0	M0	30 None/Unknown
Male	60 years	T3	N0	M1b	26 None/Unknown
Male	31 years	T1	N0	M1b	0 None/Unknown
Female	15 years	TX	N0	M1b	0 Beam radiation
Male	23 years	TX	N0	M0	0 Beam radiation
Female	58 years	Blank(s)	Blank(s)	Blank(s)	42 None/Unknown
Male	14 years	T2	N0	M1b	0 Beam radiation
Male	14 years	T2	N0	M1a	0 Recommended, un
Male	08 years	TX	NX	M0	30 None/Unknown
Male	57 years	T1	N0	M0	25 Recommended, un
Male	19 years	T3	N0	M0	0 None/Unknown
Male	42 years	TX	N0	M1b	0 Beam radiation
Female	17 years	T1	N0	M0	0 Beam radiation
Male	20 years	T1	N0	M0	25 None/Unknown
Female	11 years	T2	N0	M0	0 Beam radiation
Female	09 years	TX	NX	M1b	0 None/Unknown
Female	45 years	T2	N0	M0	0 Beam radiation
Female	55 years	T2	N0	M0	42 None/Unknown
Male	24 years	T2	N0	M0	26 Beam radiation
Female	58 years	T2	N1	M1b	0 None/Unknown
Male	10 years	T1	N0	M0	25 None/Unknown
Male	16 years	T0	N0	M1b	0 Beam radiation
Female	16 years	T1	N0	M1NOS	0 Beam radiation
Female	06 years	TX	N0	M0	30 None/Unknown
Female	12 years	T1	N1	M1a	0 Beam radiation
Female	46 years	T1	N0	M0	30 Beam radiation
Male	08 years	T1	N0	M0	0 Beam radiation
Male	09 years	T2	N0	M0	0 Beam radiation
Male	09 years	T1	N0	M0	0 Beam radiation
Male	15 years	T2	N0	M0	0 Beam radiation
Male	20 years	T2	N0	M0	0 Beam radiation
Female	81 years	T2	N0	M0	0 None/Unknown
Male	52 years	T1	N0	M0	0 Beam radiation

Female	04 years	T2	N0	M0	30	Beam radiation
Male	13 years	T1	N0	M0	25	None/Unknown
Male	25 years	T1	N0	M0	30	None/Unknown
Male	19 years	T3	N0	M1b	0	Beam radiation
Male	27 years	T2	NX	M0	30	None/Unknown
Male	31 years	T2	N0	M0	30	None/Unknown
Male	21 years	T1	N0	M0	0	None/Unknown
Female	13 years	TX	N0	M0	0	Beam radiation
Female	24 years	Blank(s)	Blank(s)	Blank(s)	0	None/Unknown
Female	43 years	T3	N0	M0	30	Beam radiation
Female	17 years	T1	N0	M0	26	None/Unknown
Female	47 years	T2	N0	M1a	0	None/Unknown
Male	19 years	T1	N0	M0	25	None/Unknown
Male	13 years	T1	N0	M0	54	None/Unknown
Female	12 years	T2	N1	M1b	0	Beam radiation
Male	11 years	T1	N0	M0	30	None/Unknown
Male	27 years	T2	N0	M0	30	None/Unknown
Female	95 years	TX	NX	MX	0	None/Unknown
Male	20 years	Blank(s)	Blank(s)	Blank(s)	0	None/Unknown
Male	31 years	Blank(s)	Blank(s)	Blank(s)	0	None/Unknown
Male	23 years	Blank(s)	Blank(s)	Blank(s)	0	Beam radiation
Male	09 years	Blank(s)	Blank(s)	Blank(s)	30	None/Unknown
Female	04 years	Blank(s)	Blank(s)	Blank(s)	30	None/Unknown
Male	15 years	Blank(s)	Blank(s)	Blank(s)	30	Beam radiation
Male	06 years	Blank(s)	Blank(s)	Blank(s)	30	None/Unknown
Male	06 years	Blank(s)	Blank(s)	Blank(s)	26	None/Unknown
Male	17 years	Blank(s)	Blank(s)	Blank(s)	30	None/Unknown
Male	14 years	Blank(s)	Blank(s)	Blank(s)	30	None/Unknown
Male	37 years	Blank(s)	Blank(s)	Blank(s)	30	Beam radiation
Male	15 years	Blank(s)	Blank(s)	Blank(s)	25	Beam radiation
Female	07 years	T2	N0	M1a	30	None/Unknown
Male	16 years	Blank(s)	Blank(s)	Blank(s)	25	Beam radiation
Female	10 years	Blank(s)	Blank(s)	Blank(s)	0	Beam radiation
Male	04 years	Blank(s)	Blank(s)	Blank(s)	30	Beam radiation
Male	07 years	Blank(s)	Blank(s)	Blank(s)	0	Beam radiation
Female	14 years	Blank(s)	Blank(s)	Blank(s)	0	Beam radiation
Female	10 years	Blank(s)	Blank(s)	Blank(s)	0	Beam radiation
Male	12 years	Blank(s)	Blank(s)	Blank(s)	30	Beam radiation
Female	15 years	Blank(s)	Blank(s)	Blank(s)	30	Recommended, un
Male	10 years	Blank(s)	Blank(s)	Blank(s)	0	Beam radiation
Male	22 years	Blank(s)	Blank(s)	Blank(s)	30	None/Unknown
Male	19 years	Blank(s)	Blank(s)	Blank(s)	0	Beam radiation
Female	16 years	Blank(s)	Blank(s)	Blank(s)	25	Beam radiation
Female	56 years	Blank(s)	Blank(s)	Blank(s)	0	None/Unknown
Female	18 years	Blank(s)	Blank(s)	Blank(s)	0	Beam radiation
Female	38 years	Blank(s)	Blank(s)	Blank(s)	0	None/Unknown
Male	13 years	Blank(s)	Blank(s)	Blank(s)	0	Beam radiation
Female	12 years	Blank(s)	Blank(s)	Blank(s)	0	Beam radiation
Female	24 years	Blank(s)	Blank(s)	Blank(s)	30	Recommended, un
Male	15 years	Blank(s)	Blank(s)	Blank(s)	26	Beam radiation
Male	04 years	Blank(s)	Blank(s)	Blank(s)	25	None/Unknown
Male	22 years	Blank(s)	Blank(s)	Blank(s)	0	Beam radiation
Female	14 years	Blank(s)	Blank(s)	Blank(s)	30	None/Unknown
Female	58 years	Blank(s)	Blank(s)	Blank(s)	41	None/Unknown
Female	67 years	Blank(s)	Blank(s)	Blank(s)	25	Beam radiation
Female	25 years	Blank(s)	Blank(s)	Blank(s)	0	Beam radiation
Male	16 years	Blank(s)	Blank(s)	Blank(s)	30	Beam radiation
Male	04 years	Blank(s)	Blank(s)	Blank(s)	25	Beam radiation

Male	10 years	Blank(s)	Blank(s)	Blank(s)	0	Beam radiation	
Male	20 years	Blank(s)	Blank(s)	Blank(s)	0	Beam radiation	
Female	33 years	Blank(s)	Blank(s)	Blank(s)	30	Beam radiation	
Male	14 years	TX	NX	M0	0	None/Unknown	
Female	12 years	Blank(s)	Blank(s)	Blank(s)	0	Beam radiation	
Female	31 years	Blank(s)	Blank(s)	Blank(s)	26	Beam radiation	
Male	14 years	Blank(s)	Blank(s)	Blank(s)	0	None/Unknown	
Male	45 years	Blank(s)	Blank(s)	Blank(s)	0	Beam radiation	
Male	34 years	TX	NX	MX	0	Radiation, NOS	me
Male	11 years	Blank(s)	Blank(s)	Blank(s)	30	None/Unknown	
Male	04 years	Blank(s)	Blank(s)	Blank(s)	30	None/Unknown	
Male	18 years	Blank(s)	Blank(s)	Blank(s)	30	None/Unknown	
Male	04 years	Blank(s)	Blank(s)	Blank(s)	30	Beam radiation	
Male	22 years	Blank(s)	Blank(s)	Blank(s)	0	Beam radiation	
Male	12 years	Blank(s)	Blank(s)	Blank(s)	41	None/Unknown	
Male	10 years	Blank(s)	Blank(s)	Blank(s)	25	Beam radiation	
Female	15 years	Blank(s)	Blank(s)	Blank(s)	30	None/Unknown	
Male	07 years	Blank(s)	Blank(s)	Blank(s)	0	Beam radiation	
Female	17 years	Blank(s)	Blank(s)	Blank(s)	25	Beam radiation	
Male	09 years	Blank(s)	Blank(s)	Blank(s)	0	Beam radiation	
Male	11 years	Blank(s)	Blank(s)	Blank(s)	26	None/Unknown	
Female	06 years	Blank(s)	Blank(s)	Blank(s)	25	None/Unknown	
Female	06 years	Blank(s)	Blank(s)	Blank(s)	0	Beam radiation	
Female	24 years	Blank(s)	Blank(s)	Blank(s)	26	Beam radiation	
Male	10 years	Blank(s)	Blank(s)	Blank(s)	0	None/Unknown	
Female	21 years	Blank(s)	Blank(s)	Blank(s)	0	None/Unknown	
Female	31 years	Blank(s)	Blank(s)	Blank(s)	30	None/Unknown	
Male	00 years	Blank(s)	Blank(s)	Blank(s)	26	Beam radiation	
Male	11 years	Blank(s)	Blank(s)	Blank(s)	41	None/Unknown	
Female	09 years	Blank(s)	Blank(s)	Blank(s)	0	Beam radiation	
Male	17 years	Blank(s)	Blank(s)	Blank(s)	25	None/Unknown	
Male	16 years	Blank(s)	Blank(s)	Blank(s)	30	Beam radiation	
Male	16 years	Blank(s)	Blank(s)	Blank(s)	30	None/Unknown	
Female	05 years	Blank(s)	Blank(s)	Blank(s)	30	None/Unknown	
Male	09 years	Blank(s)	Blank(s)	Blank(s)	0	Beam radiation	
Male	37 years	Blank(s)	Blank(s)	Blank(s)	30	None/Unknown	
Female	16 years	Blank(s)	Blank(s)	Blank(s)	0	Beam radiation	
Female	22 years	Blank(s)	Blank(s)	Blank(s)	25	None/Unknown	
Female	09 years	Blank(s)	Blank(s)	Blank(s)	0	None/Unknown	
Female	30 years	Blank(s)	Blank(s)	Blank(s)	26	Beam radiation	
Male	24 years	Blank(s)	Blank(s)	Blank(s)	0	Beam radiation	
Male	13 years	Blank(s)	Blank(s)	Blank(s)	26	Beam radiation	
Female	27 years	Blank(s)	Blank(s)	Blank(s)	0	Beam radiation	
Female	04 years	Blank(s)	Blank(s)	Blank(s)	25	None/Unknown	
Female	34 years	Blank(s)	Blank(s)	Blank(s)	0	None/Unknown	
Female	13 years	Blank(s)	Blank(s)	Blank(s)	25	None/Unknown	
Female	11 years	Blank(s)	Blank(s)	Blank(s)	40	Beam radiation	
Female	25 years	Blank(s)	Blank(s)	Blank(s)	0	None/Unknown	
Female	58 years	Blank(s)	Blank(s)	Blank(s)	41	Beam radiation	
Male	25 years	Blank(s)	Blank(s)	Blank(s)	0	None/Unknown	
Male	22 years	Blank(s)	Blank(s)	Blank(s)	0	Beam radiation	
Female	38 years	Blank(s)	Blank(s)	Blank(s)	25	Beam radiation	
Male	59 years	Blank(s)	Blank(s)	Blank(s)	0	Beam radiation	
Male	19 years	Blank(s)	Blank(s)	Blank(s)	0	Beam radiation	
Male	34 years	Blank(s)	Blank(s)	Blank(s)	0	Beam radiation	
Male	22 years	Blank(s)	Blank(s)	Blank(s)	30	None/Unknown	
Male	14 years	Blank(s)	Blank(s)	Blank(s)	53	None/Unknown	
Male	03 years	Blank(s)	Blank(s)	Blank(s)	30	None/Unknown	

Female	18 years	Blank(s)	Blank(s)	Blank(s)	90	None/Unknown
Male	20 years	Blank(s)	Blank(s)	Blank(s)	30	Beam radiation
Male	27 years	Blank(s)	Blank(s)	Blank(s)	0	None/Unknown
Female	19 years	Blank(s)	Blank(s)	Blank(s)	30	Recommended, un
Male	30 years	Blank(s)	Blank(s)	Blank(s)	0	Beam radiation
Female	26 years	Blank(s)	Blank(s)	Blank(s)	30	None/Unknown
Male	14 years	Blank(s)	Blank(s)	Blank(s)	30	Beam radiation
Male	12 years	Blank(s)	Blank(s)	Blank(s)	0	Beam radiation
Male	27 years	Blank(s)	Blank(s)	Blank(s)	99	Radiation, NOS me
Male	21 years	Blank(s)	Blank(s)	Blank(s)	0	None/Unknown
Female	40 years	Blank(s)	Blank(s)	Blank(s)	0	None/Unknown
Female	24 years	Blank(s)	Blank(s)	Blank(s)	99	None/Unknown
Female	35 years	Blank(s)	Blank(s)	Blank(s)	0	None/Unknown
Male	78 years	Blank(s)	Blank(s)	Blank(s)	0	Recommended, un
Male	31 years	Blank(s)	Blank(s)	Blank(s)	0	None/Unknown
Male	35 years	T3	N0	M0	30	None/Unknown
Male	14 years	Blank(s)	Blank(s)	Blank(s)	42	None/Unknown
Female	26 years	Blank(s)	Blank(s)	Blank(s)	40	None/Unknown
Male	20 years	T2	N0	M1b	0	None/Unknown
Male	14 years	TX	N0	M0	30	Beam radiation
Female	03 years	T1	N0	M0	25	None/Unknown
Male	10 years	Blank(s)	Blank(s)	Blank(s)	30	None/Unknown
Female	11 years	Blank(s)	Blank(s)	Blank(s)	90	None/Unknown
Male	11 years	TX	N0	M0	53	Beam radiation
Female	12 years	T1	N0	M0	42	Beam radiation
Male	19 years	TX	NX	M1b	0	None/Unknown
Female	23 years	T1	N0	M0	26	Beam radiation
Male	05 years	T1	N0	M0	26	Beam radiation
Male	11 years	T1	N0	M0	30	None/Unknown
Male	30 years	T2	N0	M0	30	None/Unknown
Female	09 years	TX	NX	MX	30	None/Unknown
Male	15 years	TX	N0	M1a	0	Beam radiation
Male	16 years	T3	N0	M1a	25	Beam radiation
Male	56 years	T3	N1	M1b	0	None/Unknown
Male	14 years	T2	N0	M0	30	None/Unknown
Female	07 years	T1	N0	M0	30	None/Unknown
Male	48 years	TX	N0	M0	26	Beam radiation
Female	16 years	T3	N0	M1b	0	Beam radiation
Female	26 years	TX	NX	MX	0	None/Unknown
Male	19 years	Blank(s)	Blank(s)	Blank(s)	30	Radiation, NOS me
Male	24 years	T2	N0	M0	30	None/Unknown
Male	16 years	T1	N0	M0	30	None/Unknown
Male	14 years	TX	N0	M0	0	None/Unknown
Male	16 years	TX	N0	M0	25	Beam radiation
Female	14 years	TX	N0	M1a	30	None/Unknown
Male	11 years	T1	N0	M0	30	None/Unknown
Male	22 years	T1	N0	M0	25	None/Unknown
Male	15 years	TX	N0	M0	30	Beam radiation
Male	07 years	T2	N1	M0	30	Combination of be
Female	24 years	T1	N0	M0	30	None/Unknown
Female	11 years	T3	N0	M0	30	Radioisotopes (198
Male	12 years	T2	NX	M1a	0	Beam radiation
Male	22 years	T2	N0	M0	0	Beam radiation
Female	13 years	TX	N0	M1a	26	Beam radiation
Female	12 years	TX	NX	MX	25	Beam radiation
Male	13 years	T1	N0	M0	30	None/Unknown
Female	35 years	T2	N0	M0	0	None/Unknown
Male	15 years	T1	N0	M0	30	None/Unknown

Male	04 years	T2	N0	M1a	30	Beam radiation	
Female	10 years	T1	N0	M0	30	None/Unknown	
Female	17 years	T1	N0	M0	26	None/Unknown	
Female	08 years	T1	N0	M0	26	Beam radiation	
Female	25 years	T1	N0	M0	0	None/Unknown	
Male	31 years	T2	N0	M0	0	None/Unknown	
Female	14 years	T2	N0	M1a	54	Beam radiation	
Male	27 years	T1	N0	M0	30	None/Unknown	
Male	04 years	T1	N0	M0	30	None/Unknown	
Male	21 years	T1	N0	M0	41	None/Unknown	
Male	16 years	T1	N0	M0	30	None/Unknown	
Male	09 years	T1	N0	M0	25	Beam radiation	
Female	08 years	T3	N0	M1b	0	Beam radiation	
Female	15 years	TX	N0	M0	0	None/Unknown	
Male	55 years	TX	N0	M1b	0	Beam radiation	
Male	11 years	T2	N0	M0	0	Beam radiation	
Female	09 years	T1	N0	M0	26	None/Unknown	
Female	26 years	T1	N0	M0	0	Beam radiation	
Male	09 years	T2	N0	M1b	30	Beam radiation	
Female	16 years	T2	N0	M0	30	None/Unknown	
Male	28 years	T1	N0	M0	30	None/Unknown	
Female	40 years	T1	N0	M0	26	None/Unknown	
Female	23 years	T1	N0	M1a	0	Beam radiation	
Male	03 years	T1	N1	M1a	53	Beam radiation	
Male	28 years	Blank(s)	Blank(s)	Blank(s)	99	None/Unknown	
Male	21 years	Blank(s)	Blank(s)	Blank(s)	0	Beam radiation	
Male	11 years	Blank(s)	Blank(s)	Blank(s)	30	None/Unknown	
Male	05 years	T1	N0	M0	0	Beam radiation	
Female	17 years	Blank(s)	Blank(s)	Blank(s)	0	Beam radiation	
Female	16 years	Blank(s)	Blank(s)	Blank(s)	0	Beam radiation	
Female	13 years	Blank(s)	Blank(s)	Blank(s)	30	None/Unknown	
Male	25 years	Blank(s)	Blank(s)	Blank(s)	0	Beam radiation	
Male	06 years	Blank(s)	Blank(s)	Blank(s)	0	Beam radiation	
Female	20 years	Blank(s)	Blank(s)	Blank(s)	51	None/Unknown	
Male	05 years	TX	NX	M0	0	Beam radiation	
Male	13 years	TX	NX	M0	0	Beam radiation	
Male	05 years	Blank(s)	Blank(s)	Blank(s)	30	Beam radiation	
Male	62 years	Blank(s)	Blank(s)	Blank(s)	0	None/Unknown	
Female	17 years	Blank(s)	Blank(s)	Blank(s)	25	Beam radiation	
Female	11 years	Blank(s)	Blank(s)	Blank(s)	0	Beam radiation	
Male	23 years	Blank(s)	Blank(s)	Blank(s)	30	None/Unknown	
Male	06 years	Blank(s)	Blank(s)	Blank(s)	26	Beam radiation	
Male	13 years	Blank(s)	Blank(s)	Blank(s)	30	None/Unknown	
Female	60 years	Blank(s)	Blank(s)	Blank(s)	26	None/Unknown	
Male	15 years	Blank(s)	Blank(s)	Blank(s)	30	None/Unknown	
Female	08 years	Blank(s)	Blank(s)	Blank(s)	30	Beam radiation	
Female	28 years	Blank(s)	Blank(s)	Blank(s)	30	None/Unknown	
Female	71 years	Blank(s)	Blank(s)	Blank(s)	0	Beam radiation	
Male	19 years	Blank(s)	Blank(s)	Blank(s)	0	None/Unknown	
Male	12 years	Blank(s)	Blank(s)	Blank(s)	0	None/Unknown	
Male	41 years	Blank(s)	Blank(s)	Blank(s)	0	None/Unknown	
Male	16 years	Blank(s)	Blank(s)	Blank(s)	0	Beam radiation	
Female	35 years	Blank(s)	Blank(s)	Blank(s)	0	None/Unknown	
Female	12 years	Blank(s)	Blank(s)	Blank(s)	0	Beam radiation	
Female	06 years	Blank(s)	Blank(s)	Blank(s)	0	None/Unknown	
Female	13 years	Blank(s)	Blank(s)	Blank(s)	0	None/Unknown	
Male	20 years	Blank(s)	Blank(s)	Blank(s)	90	Radiation, NOS	me
Male	20 years	TX	N0	M1b	0	None/Unknown	

Female	14 years	T1	N0	M0	30	None/Unknown
Male	20 years	T1	N0	M0	0	None/Unknown
Male	17 years	TX	N0	M1a	0	None/Unknown
Male	33 years	TX	N0	M0	0	Beam radiation
Female	22 years	TX	NX	M1b	0	None/Unknown
Male	17 years	T1	N0	M0	0	Beam radiation
Female	06 years	Blank(s)	Blank(s)	Blank(s)	0	None/Unknown
Female	10 years	TX	N0	M0	25	Beam radiation
Male	50 years	TX	NX	M1b	0	Radiation, NOS me
Female	17 years	Blank(s)	Blank(s)	Blank(s)	0	None/Unknown
Male	20 years	Blank(s)	Blank(s)	Blank(s)	0	None/Unknown
Male	11 years	TX	NX	MX	30	None/Unknown
Male	14 years	T1	N0	M1b	0	Beam radiation
Male	24 years	TX	N0	M0	0	Beam radiation
Female	18 years	Blank(s)	Blank(s)	Blank(s)	30	None/Unknown
Male	26 years	T1	N0	M0	30	None/Unknown
Male	03 years	Blank(s)	Blank(s)	Blank(s)	90	None/Unknown
Male	28 years	Blank(s)	Blank(s)	Blank(s)	0	None/Unknown
Female	23 years	T1	N0	M0	0	None/Unknown
Male	26 years	T2	N0	M0	30	None/Unknown
Male	55 years	T1	N0	M1a	30	None/Unknown
Male	09 years	TX	N1	M1b	0	None/Unknown
Female	11 years	T2	N0	M0	0	Beam radiation
Male	17 years	T2	N0	M0	30	Beam radiation
Female	59 years	T1	N0	M0	25	None/Unknown
Male	16 years	TX	N0	M0	0	None/Unknown
Male	06 years	TX	N0	M1b	0	None/Unknown
Male	21 years	TX	N0	M0	99	None/Unknown
Female	09 years	T1	N0	M0	26	Beam radiation
Male	15 years	T1	N0	M0	30	Beam radiation
Male	24 years	T1	N1	M1a	25	None/Unknown
Female	58 years	TX	N1	M1b	0	None/Unknown
Female	23 years	TX	N0	M1a	30	None/Unknown
Male	49 years	T1	N0	M0	30	Recommended, un
Male	10 years	TX	N0	M0	41	None/Unknown
Female	12 years	T2	N0	M1a	0	Beam radiation
Female	06 years	TX	N0	M1b	30	None/Unknown
Male	22 years	T1	N1	M0	30	None/Unknown
Female	11 years	T2	N0	M0	30	None/Unknown
Male	29 years	T2	NX	M0	0	Beam radiation
Male	66 years	T1	N0	M1a	0	None/Unknown
Male	06 years	TX	NX	MX	99	None/Unknown
Male	27 years	TX	NX	M1b	99	Beam radiation
Male	09 years	TX	N0	M0	30	Beam radiation
Male	18 years	T2	N0	M1b	0	None/Unknown
Male	17 years	T2	N0	M1b	0	Beam radiation
Female	13 years	T3	N0	M0	0	Beam radiation
Male	20 years	T2	N0	M0	30	None/Unknown
Male	26 years	TX	NX	MX	26	None/Unknown
Male	17 years	T1	N0	M0	25	None/Unknown
Male	23 years	T1	N0	M0	25	Beam radiation
Male	21 years	TX	N0	M0	0	None/Unknown
Male	61 years	T2	N0	M1b	0	None/Unknown
Male	21 years	Blank(s)	Blank(s)	Blank(s)	0	None/Unknown
Male	02 years	TX	NX	MX	90	None/Unknown
Female	15 years	Blank(s)	Blank(s)	Blank(s)	0	None/Unknown
Female	13 years	Blank(s)	Blank(s)	Blank(s)	0	Beam radiation
Female	09 years	Blank(s)	Blank(s)	Blank(s)	0	None/Unknown

Male	17 years	Blank(s)	Blank(s)	Blank(s)	0	None/Unknown
Male	14 years	Blank(s)	Blank(s)	Blank(s)	30	None/Unknown
Female	11 years	Blank(s)	Blank(s)	Blank(s)	0	Beam radiation
Male	19 years	Blank(s)	Blank(s)	Blank(s)	0	Beam radiation
Male	12 years	Blank(s)	Blank(s)	Blank(s)	40	None/Unknown
Male	43 years	Blank(s)	Blank(s)	Blank(s)	25	Beam radiation
Male	43 years	Blank(s)	Blank(s)	Blank(s)	0	Refused (1988+)
Female	07 years	Blank(s)	Blank(s)	Blank(s)	30	None/Unknown
Male	38 years	Blank(s)	Blank(s)	Blank(s)	0	None/Unknown
Female	06 years	Blank(s)	Blank(s)	Blank(s)	0	None/Unknown
Male	24 years	Blank(s)	Blank(s)	Blank(s)	0	None/Unknown
Male	18 years	Blank(s)	Blank(s)	Blank(s)	0	Beam radiation
Male	15 years	Blank(s)	Blank(s)	Blank(s)	0	None/Unknown
Female	20 years	Blank(s)	Blank(s)	Blank(s)	30	Beam radiation
Male	21 years	Blank(s)	Blank(s)	Blank(s)	0	Beam radiation
Male	16 years	Blank(s)	Blank(s)	Blank(s)	42	Beam radiation
Male	22 years	Blank(s)	Blank(s)	Blank(s)	0	Beam radiation
Female	25 years	Blank(s)	Blank(s)	Blank(s)	30	None/Unknown
Male	36 years	Blank(s)	Blank(s)	Blank(s)	0	Beam radiation
Male	04 years	Blank(s)	Blank(s)	Blank(s)	0	Beam radiation
Male	07 years	Blank(s)	Blank(s)	Blank(s)	0	None/Unknown
Male	20 years	TX	N0	M0	25	None/Unknown
Male	62 years	T2	N0	M0	30	None/Unknown
Male	45 years	T1	N0	M0	30	None/Unknown
Male	13 years	Blank(s)	Blank(s)	Blank(s)	90	None/Unknown
Male	25 years	TX	NX	M1NOS	0	None/Unknown
Female	21 years	Blank(s)	Blank(s)	Blank(s)	90	Beam radiation
Male	09 years	Blank(s)	Blank(s)	Blank(s)	19	Beam radiation
Male	08 years	Blank(s)	Blank(s)	Blank(s)	0	None/Unknown
Male	56 years	Blank(s)	Blank(s)	Blank(s)	90	None/Unknown
Male	11 years	Blank(s)	Blank(s)	Blank(s)	30	None/Unknown
Male	73 years	Blank(s)	Blank(s)	Blank(s)	0	None/Unknown
Female	22 years	Blank(s)	Blank(s)	Blank(s)	30	None/Unknown
Male	18 years	Blank(s)	Blank(s)	Blank(s)	41	None/Unknown
Male	15 years	Blank(s)	Blank(s)	Blank(s)	0	Combination of be
Male	46 years	Blank(s)	Blank(s)	Blank(s)	30	Beam radiation
Male	46 years	Blank(s)	Blank(s)	Blank(s)	30	Beam radiation
Male	15 years	Blank(s)	Blank(s)	Blank(s)	90	None/Unknown
Female	12 years	Blank(s)	Blank(s)	Blank(s)	30	None/Unknown
Male	11 years	Blank(s)	Blank(s)	Blank(s)	90	None/Unknown
Male	17 years	Blank(s)	Blank(s)	Blank(s)	0	None/Unknown
Female	14 years	Blank(s)	Blank(s)	Blank(s)	30	None/Unknown
Female	40 years	Blank(s)	Blank(s)	Blank(s)	19	Beam radiation
Female	13 years	Blank(s)	Blank(s)	Blank(s)	0	None/Unknown
Female	19 years	Blank(s)	Blank(s)	Blank(s)	19	None/Unknown
Male	15 years	Blank(s)	Blank(s)	Blank(s)	0	None/Unknown
Male	47 years	Blank(s)	Blank(s)	Blank(s)	19	Beam radiation
Male	30 years	Blank(s)	Blank(s)	Blank(s)	25	Beam radiation
Female	04 years	Blank(s)	Blank(s)	Blank(s)	90	None/Unknown
Male	20 years	Blank(s)	Blank(s)	Blank(s)	0	None/Unknown
Female	35 years	Blank(s)	Blank(s)	Blank(s)	30	Beam radiation
Female	37 years	Blank(s)	Blank(s)	Blank(s)	0	Beam radiation
Male	03 years	Blank(s)	Blank(s)	Blank(s)	90	Beam radiation
Male	42 years	Blank(s)	Blank(s)	Blank(s)	0	Beam radiation
Male	28 years	Blank(s)	Blank(s)	Blank(s)	90	None/Unknown
Male	11 years	Blank(s)	Blank(s)	Blank(s)	0	None/Unknown
Female	10 years	Blank(s)	Blank(s)	Blank(s)	19	Beam radiation
Male	17 years	Blank(s)	Blank(s)	Blank(s)	0	Beam radiation

Male	12 years	Blank(s)	Blank(s)	Blank(s)	99	None/Unknown
Female	23 years	Blank(s)	Blank(s)	Blank(s)	30	None/Unknown
Male	22 years	Blank(s)	Blank(s)	Blank(s)	53	Beam radiation
Male	13 years	Blank(s)	Blank(s)	Blank(s)	30	None/Unknown
Male	34 years	Blank(s)	Blank(s)	Blank(s)	0	None/Unknown
Male	17 years	Blank(s)	Blank(s)	Blank(s)	53	None/Unknown
Female	15 years	Blank(s)	Blank(s)	Blank(s)	30	None/Unknown
Male	23 years	Blank(s)	Blank(s)	Blank(s)	19	None/Unknown
Male	57 years	Blank(s)	Blank(s)	Blank(s)	0	None/Unknown
Female	22 years	Blank(s)	Blank(s)	Blank(s)	30	None/Unknown
Male	39 years	Blank(s)	Blank(s)	Blank(s)	0	Beam radiation
Female	10 years	Blank(s)	Blank(s)	Blank(s)	30	None/Unknown
Male	05 years	Blank(s)	Blank(s)	Blank(s)	30	None/Unknown
Male	36 years	Blank(s)	Blank(s)	Blank(s)	30	None/Unknown
Male	12 years	Blank(s)	Blank(s)	Blank(s)	30	Beam radiation
Male	10 years	T1	N0	M0	90	None/Unknown
Female	17 years	Blank(s)	Blank(s)	Blank(s)	19	None/Unknown
Male	40 years	Blank(s)	Blank(s)	Blank(s)	0	Beam radiation
Male	08 years	T2	N0	M0	41	None/Unknown
Female	16 years	Blank(s)	Blank(s)	Blank(s)	15	Beam radiation
Female	12 years	T1	N0	M0	0	Beam radiation
Male	02 years	Blank(s)	Blank(s)	Blank(s)	50	None/Unknown
Female	15 years	Blank(s)	Blank(s)	Blank(s)	30	Beam radiation
Male	15 years	T2	N0	M0	30	None/Unknown
Male	27 years	T1	N0	M0	30	None/Unknown
Female	10 years	TX	NX	M0	26	Beam radiation
Female	34 years	T2	N0	M0	90	Beam radiation
Female	58 years	T2	N0	M0	0	Beam radiation
Female	12 years	T2	N0	M0	30	None/Unknown
Male	11 years	TX	N0	M0	53	None/Unknown
Female	06 years	T2	N0	M0	90	None/Unknown
Female	71 years	TX	NX	M0	30	Beam radiation
Female	09 years	T1	N0	M0	90	None/Unknown
Female	26 years	T1	N0	M0	0	None/Unknown
Female	05 years	T1	N0	M0	0	Beam radiation
Male	06 years	T2	NX	M0	30	None/Unknown
Male	14 years	T1	N0	M0	30	Beam radiation
Male	45 years	TX	NX	MX	25	None/Unknown
Male	18 years	T2	N0	M1b	0	Beam radiation
Male	15 years	T2	N0	M0	30	None/Unknown
Female	62 years	TX	NX	MX	0	None/Unknown
Female	04 years	T1	N0	M0	25	None/Unknown
Female	22 years	T2	N0	M0	19	Beam radiation
Female	28 years	TX	N0	M1b	0	None/Unknown
Female	09 years	T1	N0	M0	30	None/Unknown
Female	62 years	T2	N0	M0	26	None/Unknown
Female	35 years	T1	N0	M0	30	None/Unknown
Female	18 years	T2	N0	M0	0	Beam radiation
Female	05 years	TX	N0	M0	30	None/Unknown
Male	13 years	T2	N0	M1b	30	Beam radiation
Male	02 years	T1	N0	M0	30	None/Unknown
Female	13 years	TX	NX	M1b	0	Beam radiation
Male	35 years	TX	NX	M0	26	None/Unknown
Male	13 years	T1	N0	M0	30	None/Unknown
Male	00 years	TX	NX	M1b	41	None/Unknown
Female	14 years	TX	N0	M0	25	Beam radiation
Female	16 years	TX	N0	M1b	0	Beam radiation
Female	56 years	T1	N0	M0	30	Beam radiation

Male	13 years	TX	N0	M0	50	Beam radiation
Male	16 years	TX	N0	M0	30	Beam radiation
Male	08 years	TX	N0	M0	26	None/Unknown
Male	15 years	TX	N0	M0	30	None/Unknown
Male	41 years	T2	N0	M0	25	None/Unknown
Male	02 years	T1	N0	M0	0	None/Unknown
Female	42 years	T1	N0	M0	25	Beam radiation
Male	11 years	TX	N0	M0	30	None/Unknown
Female	29 years	TX	NX	M0	0	Beam radiation
Male	14 years	T2	N0	M0	30	None/Unknown
Male	04 years	TX	N0	M0	26	Beam radiation
Male	23 years	T1	N0	M0	0	Beam radiation
Female	32 years	T1	N1	M1a	30	Beam radiation
Male	39 years	T2	N0	M1NOS	50	None/Unknown
Male	49 years	T1	N0	M0	40	None/Unknown
Male	27 years	T2	N0	M1a	41	None/Unknown
Female	16 years	TX	N0	M0	0	Beam radiation
Male	07 years	T1	N0	M0	30	None/Unknown
Female	13 years	T3	N0	M1b	30	Beam radiation
Male	08 years	TX	N0	M0	26	None/Unknown
Male	43 years	TX	N0	M0	25	Beam radiation
Male	10 years	T1	N0	M0	0	None/Unknown
Male	37 years	Blank(s)	Blank(s)	Blank(s)	26	Beam radiation
Female	10 years	T2	N0	M0	30	None/Unknown
Male	27 years	T2	N0	M1b	0	None/Unknown
Female	23 years	T2	N0	M1b	0	None/Unknown
Female	43 years	T1	N0	M0	0	Beam radiation
Male	14 years	T2	N0	M0	30	None/Unknown
Male	22 years	T1	N0	M0	25	None/Unknown
Male	33 years	T2	N0	M0	0	None/Unknown
Male	14 years	T1	N0	M0	0	Beam radiation
Male	22 years	TX	N1	M0	54	None/Unknown
Male	03 years	TX	NX	MX	30	None/Unknown
Female	12 years	T2	N0	M0	30	None/Unknown
Male	06 years	T1	N0	M1a	40	Beam radiation
Male	22 years	T2	N0	M1b	30	None/Unknown
Male	20 years	T3	N0	M1NOS	0	None/Unknown
Female	17 years	TX	NX	MX	0	None/Unknown
Male	12 years	T1	N0	M0	26	None/Unknown
Female	22 years	T2	N0	M0	0	Beam radiation
Female	16 years	T1	N0	M0	30	None/Unknown
Male	06 years	T2	N0	M1a	0	Beam radiation
Male	22 years	TX	N1	M1NOS	25	Beam radiation
Female	12 years	T2	N0	M0	30	None/Unknown
Male	30 years	TX	N0	MX	0	None/Unknown
Female	87 years	TX	N0	M1b	0	None/Unknown
Female	21 years	T2	N0	M0	30	None/Unknown
Male	40 years	T3	N1	M1NOS	0	Beam radiation
Male	16 years	T2	N0	M1b	0	None/Unknown
Female	55 years	T2	N0	M0	30	None/Unknown
Female	10 years	T2	N0	M0	30	None/Unknown
Female	13 years	TX	N0	M1b	0	Beam radiation
Male	24 years	TX	N0	M0	30	None/Unknown
Female	16 years	T2	N0	M0	0	Radioactive implan
Male	26 years	T1	N0	M1NOS	25	Recommended, un
Male	55 years	T1	N0	M0	30	Beam radiation
Male	29 years	T2	N0	M0	30	None/Unknown
Male	07 years	T1	N0	M0	26	None/Unknown

Male	15 years	T3	N0	M1b	25	Beam radiation
Female	15 years	T2	N0	M0	30	Beam radiation
Male	04 years	T1	N0	M0	26	None/Unknown
Female	11 years	TX	N0	M0	90	None/Unknown
Female	17 years	T2	N0	M0	26	Beam radiation
Male	51 years	TX	N0	M0	90	Beam radiation
Male	44 years	T1	NX	MX	0	None/Unknown
Male	64 years	T1	N0	M0	25	Beam radiation
Female	09 years	T1	N0	M0	54	None/Unknown
Male	32 years	T1	N0	M0	26	None/Unknown
Female	06 years	T1	N0	M0	30	None/Unknown
Female	14 years	TX	N0	M0	26	Beam radiation
Male	29 years	TX	NX	M1b	30	Beam radiation
Male	13 years	T2	N1	M1b	51	None/Unknown
Female	23 years	T1	N0	M0	19	None/Unknown
Male	15 years	T1	N0	M1b	0	Beam radiation
Male	15 years	T1	N0	M0	30	None/Unknown
Male	25 years	T2	N0	M0	51	None/Unknown
Male	09 years	T1	N0	M0	30	None/Unknown
Male	22 years	Blank(s)	Blank(s)	Blank(s)	30	None/Unknown
Female	29 years	TX	NX	M0	26	None/Unknown
Male	19 years	TX	N0	M1b	0	None/Unknown
Male	14 years	T1	N0	M0	25	Beam radiation
Female	35 years	T1	N0	M1b	0	Beam radiation
Male	23 years	T2	N0	M0	0	Beam radiation
Female	17 years	T1	N0	M0	0	Beam radiation
Female	54 years	T3	N1	M1b	0	None/Unknown
Female	04 years	T1	N0	M0	0	Beam radiation
Female	41 years	T1	N0	M0	30	None/Unknown
Female	67 years	T1	N0	M1b	0	Beam radiation
Female	15 years	T1	N0	M0	25	Beam radiation
Male	16 years	T2	N0	M0	0	None/Unknown
Female	13 years	TX	NX	M1a	0	None/Unknown
Male	20 years	T2	N1	M1b	0	Beam radiation
Male	17 years	T1	N1	M0	30	None/Unknown
Male	12 years	TX	NX	MX	90	Beam radiation
Female	05 years	T1	N0	M0	25	Beam radiation
Male	10 years	T1	N0	M0	0	Beam radiation
Male	30 years	T1	N0	M0	30	None/Unknown
Male	25 years	T2	NX	M1b	0	None/Unknown
Male	40 years	T3	N0	M1b	0	Beam radiation
Male	18 years	T1	N0	M0	0	None/Unknown
Male	10 years	T1	N0	M0	30	None/Unknown
Male	14 years	T2	N0	M0	0	Beam radiation
Male	22 years	T1	N0	M0	30	Beam radiation
Female	25 years	T1	N0	M0	30	None/Unknown
Male	26 years	T3	N1	M1b	0	Beam radiation
Male	14 years	TX	N0	M0	30	None/Unknown
Female	11 years	T2	N0	M0	0	Beam radiation
Male	35 years	TX	NX	M1NOS	0	None/Unknown
Male	67 years	Blank(s)	Blank(s)	Blank(s)	0	Radiation, NOS me
Male	13 years	Blank(s)	Blank(s)	Blank(s)	30	None/Unknown
Female	11 years	Blank(s)	Blank(s)	Blank(s)	30	Recommended, un
Female	58 years	Blank(s)	Blank(s)	Blank(s)	0	None/Unknown
Female	13 years	Blank(s)	Blank(s)	Blank(s)	30	None/Unknown
Male	05 years	Blank(s)	Blank(s)	Blank(s)	30	None/Unknown
Male	15 years	Blank(s)	Blank(s)	Blank(s)	0	Beam radiation
Male	41 years	Blank(s)	Blank(s)	Blank(s)	30	None/Unknown

Female	06 years	Blank(s)	Blank(s)	Blank(s)	90	None/Unknown
Female	13 years	Blank(s)	Blank(s)	Blank(s)	30	None/Unknown
Male	18 years	Blank(s)	Blank(s)	Blank(s)	30	None/Unknown
Male	07 years	Blank(s)	Blank(s)	Blank(s)	0	Beam radiation
Male	44 years	Blank(s)	Blank(s)	Blank(s)	30	None/Unknown
Male	24 years	Blank(s)	Blank(s)	Blank(s)	0	Beam radiation
Female	64 years	Blank(s)	Blank(s)	Blank(s)	0	Beam radiation
Female	22 years	Blank(s)	Blank(s)	Blank(s)	30	None/Unknown
Male	28 years	Blank(s)	Blank(s)	Blank(s)	0	Beam radiation
Female	09 years	Blank(s)	Blank(s)	Blank(s)	40	None/Unknown
Female	30 years	Blank(s)	Blank(s)	Blank(s)	30	None/Unknown
Female	14 years	Blank(s)	Blank(s)	Blank(s)	0	Beam radiation
Male	07 years	Blank(s)	Blank(s)	Blank(s)	30	Beam radiation
Male	12 years	Blank(s)	Blank(s)	Blank(s)	0	Beam radiation
Male	22 years	Blank(s)	Blank(s)	Blank(s)	0	None/Unknown
Male	70 years	Blank(s)	Blank(s)	Blank(s)	0	None/Unknown
Female	10 years	Blank(s)	Blank(s)	Blank(s)	90	Beam radiation
Female	16 years	TX	NX	M0	0	None/Unknown
Male	16 years	Blank(s)	Blank(s)	Blank(s)	30	None/Unknown
Female	08 years	Blank(s)	Blank(s)	Blank(s)	30	Beam radiation
Female	17 years	Blank(s)	Blank(s)	Blank(s)	25	Beam radiation
Male	19 years	Blank(s)	Blank(s)	Blank(s)	30	None/Unknown
Female	29 years	Blank(s)	Blank(s)	Blank(s)	0	None/Unknown
Male	11 years	Blank(s)	Blank(s)	Blank(s)	0	Beam radiation
Female	25 years	Blank(s)	Blank(s)	Blank(s)	30	None/Unknown
Male	13 years	Blank(s)	Blank(s)	Blank(s)	0	Beam radiation
Male	57 years	Blank(s)	Blank(s)	Blank(s)	0	Beam radiation
Male	27 years	Blank(s)	Blank(s)	Blank(s)	0	Beam radiation
Female	16 years	Blank(s)	Blank(s)	Blank(s)	25	None/Unknown
Female	10 years	Blank(s)	Blank(s)	Blank(s)	0	Beam radiation
Female	78 years	Blank(s)	Blank(s)	Blank(s)	30	None/Unknown
Male	11 years	Blank(s)	Blank(s)	Blank(s)	0	None/Unknown
Female	13 years	TX	NX	M0	90	None/Unknown
Female	03 years	Blank(s)	Blank(s)	Blank(s)	25	None/Unknown
Female	09 years	Blank(s)	Blank(s)	Blank(s)	26	Beam radiation
Female	17 years	Blank(s)	Blank(s)	Blank(s)	0	Beam radiation
Male	19 years	Blank(s)	Blank(s)	Blank(s)	41	None/Unknown
Male	18 years	Blank(s)	Blank(s)	Blank(s)	0	Beam radiation
Female	35 years	Blank(s)	Blank(s)	Blank(s)	0	None/Unknown
Male	26 years	Blank(s)	Blank(s)	Blank(s)	30	None/Unknown
Male	13 years	Blank(s)	Blank(s)	Blank(s)	0	None/Unknown
Male	13 years	Blank(s)	Blank(s)	Blank(s)	51	None/Unknown
Male	37 years	Blank(s)	Blank(s)	Blank(s)	30	Beam radiation
Male	20 years	Blank(s)	Blank(s)	Blank(s)	0	None/Unknown
Female	12 years	Blank(s)	Blank(s)	Blank(s)	30	None/Unknown
Female	16 years	Blank(s)	Blank(s)	Blank(s)	30	None/Unknown
Male	36 years	Blank(s)	Blank(s)	Blank(s)	50	Beam radiation
Male	09 years	Blank(s)	Blank(s)	Blank(s)	30	Beam radiation
Male	19 years	Blank(s)	Blank(s)	Blank(s)	30	None/Unknown
Female	14 years	Blank(s)	Blank(s)	Blank(s)	0	None/Unknown
Male	11 years	Blank(s)	Blank(s)	Blank(s)	0	Beam radiation
Female	05 years	Blank(s)	Blank(s)	Blank(s)	25	None/Unknown
Female	13 years	Blank(s)	Blank(s)	Blank(s)	30	None/Unknown
Female	21 years	Blank(s)	Blank(s)	Blank(s)	0	Beam radiation
Female	16 years	Blank(s)	Blank(s)	Blank(s)	0	Beam radiation
Male	10 years	Blank(s)	Blank(s)	Blank(s)	0	Beam radiation
Male	17 years	Blank(s)	Blank(s)	Blank(s)	0	Beam radiation
Male	15 years	Blank(s)	Blank(s)	Blank(s)	26	Beam radiation

Male	21 years	Blank(s)	Blank(s)	Blank(s)	0	None/Unknown
Male	05 years	Blank(s)	Blank(s)	Blank(s)	25	None/Unknown
Male	59 years	Blank(s)	Blank(s)	Blank(s)	0	None/Unknown
Male	71 years	Blank(s)	Blank(s)	Blank(s)	30	None/Unknown
Female	29 years	TX	NX	MX	0	None/Unknown
Male	03 years	Blank(s)	Blank(s)	Blank(s)	41	None/Unknown
Female	14 years	Blank(s)	Blank(s)	Blank(s)	0	None/Unknown
Male	15 years	Blank(s)	Blank(s)	Blank(s)	0	Beam radiation
Male	27 years	Blank(s)	Blank(s)	Blank(s)	25	None/Unknown
Male	12 years	Blank(s)	Blank(s)	Blank(s)	41	None/Unknown
Male	12 years	Blank(s)	Blank(s)	Blank(s)	53	None/Unknown
Male	09 years	Blank(s)	Blank(s)	Blank(s)	25	Beam radiation
Male	11 years	Blank(s)	Blank(s)	Blank(s)	30	Refused (1988+)
Female	37 years	Blank(s)	Blank(s)	Blank(s)	0	Beam radiation
Female	19 years	Blank(s)	Blank(s)	Blank(s)	30	Beam radiation
Female	41 years	Blank(s)	Blank(s)	Blank(s)	0	None/Unknown
Male	20 years	Blank(s)	Blank(s)	Blank(s)	30	None/Unknown
Female	07 years	TX	N0	M0	0	Beam radiation
Male	32 years	T2	N0	M0	0	None/Unknown
Male	00 years	Blank(s)	Blank(s)	Blank(s)	25	Beam radiation
Male	09 years	TX	N0	M0	30	None/Unknown
Male	27 years	T2	N0	M0	0	Beam radiation
Male	19 years	T2	N1	M1b	0	Beam radiation
Male	22 years	T2	N0	M0	0	None/Unknown
Male	16 years	TX	NX	M0	0	Beam radiation
Male	10 years	TX	NX	MX	99	None/Unknown
Male	31 years	TX	NX	M1b	0	None/Unknown
Male	31 years	T1	N0	M0	0	None/Unknown
Male	41 years	TX	N0	M0	0	Recommended, un
Female	09 years	TX	N0	M0	0	Beam radiation
Male	23 years	TX	N0	M1b	0	None/Unknown
Male	18 years	T1	N0	M0	26	None/Unknown
Male	23 years	TX	N0	M0	0	Beam radiation
Male	41 years	T2	N0	M0	25	None/Unknown
Male	19 years	TX	NX	MX	0	None/Unknown
Male	18 years	TX	N1	M1b	0	None/Unknown
Male	15 years	T1	N0	M1a	0	Beam radiation
Female	11 years	T1	N0	M0	41	None/Unknown
Female	10 years	T1	N0	M0	41	None/Unknown
Female	14 years	T1	N0	M0	41	None/Unknown
Male	14 years	T1	N0	M0	30	None/Unknown
Female	08 years	TX	N0	M0	0	Beam radiation
Female	12 years	T2	NX	M0	0	Beam radiation
Male	20 years	T1	N0	M0	0	None/Unknown
Male	22 years	TX	N0	M1a	0	Beam radiation
Male	01 years	T1	N0	M0	25	None/Unknown
Male	36 years	TX	N0	M0	30	None/Unknown
Male	19 years	T1	N0	M1b	0	Beam radiation
Female	04 years	TX	N0	M0	30	None/Unknown
Female	48 years	TX	NX	MX	25	None/Unknown
Male	15 years	T2	N0	M1a	30	None/Unknown
Male	16 years	T1	N0	M0	26	Beam radiation
Male	07 years	TX	N0	M0	0	Beam radiation
Female	09 years	TX	NX	M0	99	None/Unknown
Female	12 years	T2	N0	M0	30	None/Unknown
Female	15 years	T3	N0	M0	0	Beam radiation
Female	08 years	T1	N0	M0	25	None/Unknown
Male	15 years	TX	N0	M1a	0	Beam radiation

Male	15 years	T2	N0	M0	0	Beam radiation
Male	16 years	T2	N0	M0	30	None/Unknown
Female	03 years	T2	N0	M0	54	None/Unknown
Female	05 years	T2	N0	M0	40	Beam radiation
Female	31 years	T2	NX	M0	0	None/Unknown
Male	08 years	T1	N0	M0	26	Beam radiation
Male	37 years	T1	N0	M0	25	Beam radiation
Female	24 years	T1	N0	M0	30	None/Unknown
Female	11 years	T1	N0	M0	26	Beam radiation
Female	00 years	TX	N0	M0	30	None/Unknown
Male	10 years	T2	N0	M1a	30	Beam radiation
Male	11 years	T2	N0	M0	0	None/Unknown
Female	14 years	T2	N0	M0	30	None/Unknown
Male	16 years	T1	N0	M0	25	None/Unknown
Female	06 years	T1	N0	M1a	30	Beam radiation
Male	06 years	T1	N0	M0	30	None/Unknown
Female	26 years	T1	N0	M1NOS	90	Beam radiation
Male	13 years	T1	N0	M0	30	None/Unknown
Male	16 years	T3	N0	M1b	0	Beam radiation
Female	16 years	T2	N0	M0	30	None/Unknown
Female	13 years	T2	N0	M1b	0	Beam radiation
Male	05 years	TX	N0	M1a	0	Beam radiation
Female	16 years	T1	N0	M1b	0	Beam radiation
Female	15 years	T2	N0	M0	30	None/Unknown
Female	24 years	TX	NX	MX	0	Beam radiation
Male	03 years	T2	N0	M0	30	None/Unknown
Male	04 years	T2	N0	M1a	30	None/Unknown
Male	09 years	TX	NX	MX	25	Beam radiation
Male	05 years	TX	N0	M0	40	None/Unknown
Male	15 years	T2	N0	M0	40	None/Unknown
Male	08 years	T1	N0	M0	26	Beam radiation
Male	09 years	T1	N0	M0	0	Beam radiation
Female	12 years	TX	N0	M1b	0	None/Unknown
Male	13 years	T1	N0	M0	30	Beam radiation
Male	08 years	Blank(s)	Blank(s)	Blank(s)	0	Beam radiation
Male	10 years	T2	N0	M0	30	None/Unknown
Male	73 years	T2	N0	M1b	0	Beam radiation
Male	21 years	T2	N0	M0	30	None/Unknown
Male	13 years	TX	N0	M0	0	None/Unknown
Female	12 years	T2	N1	M1b	0	Beam radiation
Female	10 years	TX	NX	MX	90	None/Unknown
Female	15 years	T1	N0	M1b	0	Beam radiation
Female	11 years	TX	NX	MX	0	None/Unknown
Female	02 years	T1	N0	M0	0	Beam radiation
Female	36 years	T0	N0	M1a	0	None/Unknown
Male	13 years	T2	N0	M0	54	Beam radiation
Female	12 years	TX	N0	M1b	0	Beam radiation
Female	19 years	T2	N0	M1a	0	Beam radiation
Female	09 years	T1	N0	M0	25	None/Unknown
Male	14 years	T1	N0	M0	30	None/Unknown
Male	08 years	T2	N0	M0	0	Beam radiation
Male	16 years	TX	N0	M0	26	Beam radiation
Female	06 years	T2	N0	M1b	0	Beam radiation
Female	18 years	T1	N0	M0	30	None/Unknown
Female	05 years	T1	N0	M1b	0	Beam radiation
Male	48 years	T2	N0	M0	0	Beam radiation
Male	35 years	T2	N1	M1b	0	Beam radiation
Male	09 years	Blank(s)	Blank(s)	Blank(s)	30	None/Unknown

Female	02 years	Blank(s)	Blank(s)	Blank(s)	0	Beam radiation
Male	22 years	Blank(s)	Blank(s)	Blank(s)	0	Beam radiation
Male	24 years	Blank(s)	Blank(s)	Blank(s)	0	Beam radiation
Female	20 years	Blank(s)	Blank(s)	Blank(s)	42	None/Unknown
Female	07 years	Blank(s)	Blank(s)	Blank(s)	30	Beam radiation
Female	06 years	Blank(s)	Blank(s)	Blank(s)	30	Beam radiation
Female	73 years	Blank(s)	Blank(s)	Blank(s)	30	Beam radiation
Male	43 years	Blank(s)	Blank(s)	Blank(s)	0	None/Unknown
Female	16 years	Blank(s)	Blank(s)	Blank(s)	0	Beam radiation
Male	48 years	Blank(s)	Blank(s)	Blank(s)	0	Beam radiation
Male	16 years	Blank(s)	Blank(s)	Blank(s)	30	None/Unknown
Female	11 years	Blank(s)	Blank(s)	Blank(s)	0	Beam radiation
Male	13 years	Blank(s)	Blank(s)	Blank(s)	0	Beam radiation
Male	14 years	Blank(s)	Blank(s)	Blank(s)	0	Beam radiation
Male	15 years	Blank(s)	Blank(s)	Blank(s)	30	Beam radiation
Female	13 years	Blank(s)	Blank(s)	Blank(s)	0	None/Unknown
Male	36 years	Blank(s)	Blank(s)	Blank(s)	25	None/Unknown

Chemotherapy recode (yes, no/unk)	Sequence number	Survival months
Yes	2nd of 2 or more primaries	2
Yes	2nd of 2 or more primaries	191
Yes	One primary only	227
Yes	1st of 2 or more primaries	119
Yes	One primary only	231
Yes	One primary only	229
Yes	One primary only	21
Yes	One primary only	208
Yes	One primary only	31
Yes	One primary only	210
Yes	One primary only	13
Yes	One primary only	39
Yes	1st of 2 or more primaries	205
Yes	One primary only	10
Yes	1st of 2 or more primaries	138
Yes	One primary only	194
Yes	One primary only	72
Yes	One primary only	34
Yes	One primary only	166
Yes	2nd of 2 or more primaries	141
Yes	One primary only	7
Yes	One primary only	180
Yes	One primary only	88
Yes	One primary only	34
Yes	One primary only	13
Yes	One primary only	166
Yes	One primary only	50
Yes	One primary only	163
Yes	One primary only	160
Yes	One primary only	17
Yes	One primary only	77
Yes	1st of 2 or more primaries	91
Yes	One primary only	78
No/Unknown	2nd of 2 or more primaries	1
Yes	One primary only	61
Yes	One primary only	132
Yes	One primary only	130
Yes	One primary only	130
Yes	One primary only	126
Yes	One primary only	123
Yes	One primary only	121
Yes	One primary only	110
Yes	One primary only	113
Yes	One primary only	123
Yes	One primary only	49
Yes	One primary only	24
Yes	One primary only	85
Yes	One primary only	102
Yes	One primary only	47
Yes	One primary only	9
Yes	One primary only	17
Yes	One primary only	98
Yes	One primary only	97
Yes	2nd of 2 or more primaries	2
Yes	One primary only	74
Yes	1st of 2 or more primaries	81
Yes	One primary only	78

Yes	One primary only	37
Yes	One primary only	79
Yes	One primary only	65
No/Unknown	One primary only	31
Yes	One primary only	45
Yes	One primary only	55
Yes	One primary only	13
Yes	One primary only	43
Yes	One primary only	41
No/Unknown	One primary only	50
Yes	One primary only	35
Yes	One primary only	12
Yes	2nd of 2 or more primaries	0
Yes	One primary only	29
Yes	One primary only	27
Yes	One primary only	26
Yes	One primary only	19
Yes	One primary only	20
Yes	One primary only	19
Yes	One primary only	15
Yes	One primary only	10
Yes	One primary only	5
Yes	One primary only	13
Yes	One primary only	2
Yes	One primary only	1
Yes	One primary only	7
Yes	One primary only	236
Yes	One primary only	18
Yes	One primary only	8
No/Unknown	2nd of 2 or more primaries	27
Yes	One primary only	216
No/Unknown	One primary only	223
Yes	One primary only	218
Yes	One primary only	13
Yes	One primary only	12
Yes	One primary only	12
No/Unknown	One primary only	3
Yes	One primary only	22
Yes	One primary only	189
Yes	One primary only	67
Yes	One primary only	176
Yes	One primary only	172
Yes	One primary only	158
Yes	One primary only	161
Yes	One primary only	42
Yes	One primary only	19
Yes	One primary only	167
No/Unknown	1st of 2 or more primaries	42
Yes	One primary only	146
No/Unknown	One primary only	227
Yes	One primary only	146
Yes	One primary only	147
Yes	One primary only	28
Yes	One primary only	137
Yes	One primary only	13
Yes	One primary only	14
Yes	One primary only	96
Yes	One primary only	95

Yes	One primary only	13
Yes	One primary only	10
Yes	One primary only	17
Yes	One primary only	126
Yes	One primary only	119
Yes	2nd of 2 or more primaries	6
Yes	One primary only	43
Yes	One primary only	117
Yes	One primary only	111
Yes	One primary only	13
Yes	2nd of 2 or more primaries	23
Yes	One primary only	107
Yes	One primary only	102
Yes	One primary only	8
Yes	One primary only	73
Yes	One primary only	82
Yes	One primary only	95
Yes	One primary only	19
Yes	One primary only	18
Yes	One primary only	88
Yes	One primary only	83
Yes	One primary only	76
Yes	One primary only	82
Yes	One primary only	42
Yes	One primary only	69
Yes	One primary only	71
Yes	One primary only	64
Yes	2nd of 2 or more primaries	16
Yes	One primary only	67
Yes	One primary only	60
Yes	One primary only	62
Yes	One primary only	50
Yes	One primary only	51
Yes	One primary only	49
Yes	One primary only	59
Yes	One primary only	51
Yes	One primary only	16
Yes	One primary only	22
No/Unknown	One primary only	1
Yes	One primary only	42
Yes	One primary only	42
Yes	One primary only	37
Yes	One primary only	46
Yes	One primary only	32
Yes	One primary only	91
Yes	One primary only	7
Yes	One primary only	19
Yes	One primary only	14
Yes	One primary only	10
Yes	One primary only	4
Yes	One primary only	3
Yes	One primary only	1
Yes	One primary only	1
Yes	2nd of 2 or more primaries	235
Yes	One primary only	8
Yes	1st of 2 or more primaries	41
No/Unknown	One primary only	33
Yes	One primary only	186

Yes	One primary only	18
Yes	One primary only	36
Yes	One primary only	142
Yes	One primary only	4
Yes	One primary only	111
Yes	One primary only	28
Yes	One primary only	37
Yes	One primary only	86
Yes	One primary only	74
Yes	One primary only	10
Yes	One primary only	8
Yes	One primary only	11
Yes	One primary only	44
No/Unknown	2nd of 2 or more primaries	4
Yes	One primary only	16
Yes	One primary only	19
Yes	One primary only	237
Yes	One primary only	13
Yes	One primary only	234
Yes	One primary only	236
Yes	One primary only	48
Yes	One primary only	25
Yes	One primary only	59
Yes	One primary only	220
Yes	One primary only	217
Yes	One primary only	54
Yes	One primary only	29
Yes	One primary only	224
Yes	One primary only	214
Yes	One primary only	213
Yes	One primary only	212
Yes	One primary only	9
Yes	One primary only	212
Yes	One primary only	208
Yes	1st of 2 or more primaries	196
Yes	One primary only	31
Yes	One primary only	48
Yes	2nd of 2 or more primaries	92
Yes	One primary only	194
Yes	One primary only	189
Yes	One primary only	11
Yes	One primary only	23
Yes	One primary only	177
Yes	One primary only	178
Yes	One primary only	172
Yes	One primary only	174
Yes	One primary only	17
Yes	One primary only	10
Yes	One primary only	169
Yes	2nd of 2 or more primaries	117
Yes	One primary only	173
Yes	One primary only	3
Yes	One primary only	167
Yes	One primary only	160
Yes	One primary only	66
Yes	One primary only	157
Yes	One primary only	152
Yes	One primary only	148

Yes	One primary only	143
No/Unknown	One primary only	140
Yes	One primary only	137
Yes	One primary only	137
Yes	One primary only	125
Yes	One primary only	109
Yes	One primary only	18
Yes	One primary only	126
Yes	One primary only	131
Yes	One primary only	15
Yes	One primary only	115
Yes	One primary only	117
Yes	One primary only	112
Yes	One primary only	104
No/Unknown	2nd of 2 or more primaries	65
Yes	1st of 2 or more primaries	101
Yes	One primary only	119
Yes	One primary only	98
Yes	One primary only	96
Yes	One primary only	20
Yes	One primary only	6
Yes	1st of 2 or more primaries	91
Yes	One primary only	80
Yes	One primary only	35
Yes	One primary only	73
Yes	One primary only	16
Yes	One primary only	54
Yes	One primary only	65
Yes	2nd of 2 or more primaries	12
Yes	One primary only	66
Yes	One primary only	81
Yes	One primary only	60
Yes	One primary only	53
Yes	One primary only	14
Yes	2nd of 2 or more primaries	27
Yes	One primary only	33
Yes	One primary only	32
Yes	One primary only	29
Yes	One primary only	29
Yes	One primary only	27
Yes	One primary only	16
Yes	One primary only	25
Yes	One primary only	19
Yes	One primary only	16
Yes	One primary only	17
Yes	One primary only	5
Yes	One primary only	6
Yes	One primary only	4
Yes	One primary only	3
Yes	One primary only	35
Yes	One primary only	10
Yes	One primary only	233
Yes	One primary only	227
Yes	One primary only	219
Yes	One primary only	40
Yes	One primary only	31
Yes	One primary only	190
Yes	One primary only	189

Yes	1st of 2 or more primaries		132
Yes	One primary only		17
Yes	One primary only		178
Yes	One primary only		183
Yes	One primary only		117
Yes	One primary only		25
Yes	One primary only		147
Yes	One primary only		79
No/Unknown	One primary only		152
Yes	One primary only		29
Yes	One primary only		139
Yes	One primary only		123
Yes	One primary only		131
No/Unknown	One primary only		42
Yes	One primary only		49
Yes	One primary only		11
Yes	1st of 2 or more primaries		18
Yes	One primary only		91
No/Unknown	One primary only		2
Yes	One primary only		14
Yes	One primary only		56
Yes	One primary only		38
Yes	One primary only		75
No/Unknown	One primary only		102
Yes	One primary only		68
Yes	One primary only		31
Yes	One primary only		3
Yes	One primary only		8
Yes	One primary only		29
Yes	One primary only		9
Yes	One primary only		40
Yes	One primary only		16
Yes	One primary only		16
No/Unknown	One primary only		15
Yes	One primary only		8
No/Unknown	One primary only	Unknown	
Yes	One primary only		3
Yes	2nd of 2 or more primaries		4
Yes	One primary only		238
Yes	1st of 2 or more primaries		234
Yes	One primary only		234
Yes	One primary only		232
Yes	One primary only		55
Yes	One primary only		226
Yes	One primary only		227
Yes	One primary only		15
Yes	One primary only		227
Yes	One primary only		14
Yes	One primary only		11
No/Unknown	One primary only		17
Yes	One primary only		215
Yes	One primary only		65
Yes	One primary only		196
Yes	One primary only		194
Yes	One primary only		190
Yes	One primary only		13
No/Unknown	2nd of 2 or more primaries	Unknown	
Yes	One primary only		98

Yes	One primary only	177
Yes	One primary only	21
Yes	One primary only	39
Yes	One primary only	175
Yes	One primary only	170
Yes	1st of 2 or more primaries	167
Yes	One primary only	166
Yes	One primary only	169
Yes	One primary only	7
Yes	One primary only	28
Yes	One primary only	160
Yes	One primary only	75
Yes	One primary only	30
Yes	One primary only	152
Yes	One primary only	151
Yes	One primary only	150
Yes	One primary only	10
Yes	One primary only	10
Yes	One primary only	14
Yes	One primary only	137
Yes	One primary only	57
Yes	One primary only	132
Yes	One primary only	129
Yes	One primary only	134
Yes	One primary only	132
Yes	1st of 2 or more primaries	31
Yes	One primary only	127
Yes	One primary only	37
Yes	One primary only	16
Yes	One primary only	123
Yes	One primary only	123
No/Unknown	One primary only	120
Yes	One primary only	118
Yes	One primary only	116
Yes	One primary only	111
Yes	One primary only	102
Yes	One primary only	108
Yes	One primary only	28
Yes	One primary only	104
Yes	One primary only	102
Yes	One primary only	99
Yes	One primary only	44
Yes	One primary only	39
Yes	One primary only	90
Yes	One primary only	25
Yes	One primary only	33
Yes	One primary only	15
Yes	One primary only	20
Yes	One primary only	82
Yes	One primary only	81
Yes	One primary only	80
Yes	One primary only	70
Yes	One primary only	76
Yes	One primary only	75
Yes	One primary only	70
Yes	One primary only	66
Yes	One primary only	44
Yes	1st of 2 or more primaries	29

Yes	One primary only	61
Yes	One primary only	61
Yes	One primary only	27
Yes	One primary only	19
Yes	1st of 2 or more primaries	25
Yes	One primary only	25
Yes	One primary only	56
Yes	One primary only	54
No/Unknown	One primary only	47
Yes	One primary only	47
Yes	One primary only	45
Yes	One primary only	44
Yes	One primary only	36
Yes	One primary only	34
Yes	One primary only	17
Yes	One primary only	24
Yes	One primary only	20
Yes	One primary only	18
Yes	One primary only	8
Yes	One primary only	7
Yes	One primary only	7
Yes	One primary only	6
Yes	One primary only	6
Yes	One primary only	5
Yes	One primary only	3
Yes	One primary only	1
Yes	One primary only	3
Yes	One primary only	197
Yes	One primary only	54
Yes	One primary only	32
Yes	One primary only	219
Yes	One primary only	217
Yes	One primary only	16
Yes	One primary only	204
Yes	One primary only	12
Yes	One primary only	15
Yes	One primary only	194
Yes	One primary only	14
Yes	One primary only	58
Yes	One primary only	153
Yes	1st of 2 or more primaries	43
Yes	One primary only	9
Yes	One primary only	167
Yes	1st of 2 or more primaries	136
Yes	One primary only	18
Yes	One primary only	160
Yes	1st of 2 or more primaries	112
Yes	2nd of 2 or more primaries	20
Yes	One primary only	12
Yes	One primary only	24
Yes	One primary only	33
Yes	One primary only	153
Yes	One primary only	18
Yes	One primary only	136
Yes	One primary only	128
Yes	1st of 2 or more primaries	133
Yes	One primary only	133
Yes	One primary only	132

No/Unknown	One primary only	41
Yes	One primary only	81
Yes	One primary only	120
Yes	One primary only	120
Yes	One primary only	19
Yes	One primary only	11
Yes	One primary only	116
Yes	One primary only	114
Yes	One primary only	118
Yes	One primary only	107
Yes	One primary only	105
Yes	1st of 2 or more primaries	108
Yes	One primary only	35
Yes	One primary only	118
Yes	One primary only	142
Yes	One primary only	140
Yes	One primary only	15
Yes	2nd of 2 or more primaries	4
Yes	One primary only	92
Yes	One primary only	94
Yes	One primary only	64
Yes	One primary only	84
Yes	One primary only	6
Yes	One primary only	85
Yes	One primary only	94
No/Unknown	One primary only	5
Yes	1st of 2 or more primaries	68
Yes	One primary only	54
Yes	One primary only	12
Yes	One primary only	79
Yes	One primary only	7
Yes	One primary only	15
Yes	One primary only	53
Yes	One primary only	69
Yes	One primary only	32
Yes	One primary only	64
Yes	One primary only	71
Yes	One primary only	15
Yes	One primary only	58
Yes	One primary only	57
Yes	One primary only	51
No/Unknown	One primary only	57
Yes	One primary only	54
Yes	One primary only	51
Yes	One primary only	45
Yes	One primary only	42
Yes	One primary only	44
No/Unknown	One primary only	1
Yes	One primary only	40
Yes	One primary only	38
Yes	1st of 2 or more primaries	37
Yes	One primary only	39
Yes	One primary only	38
Yes	One primary only	32
Yes	One primary only	34
Yes	One primary only	25
Yes	One primary only	27
Yes	One primary only	22

Yes	One primary only	13
Yes	One primary only	10
Yes	One primary only	13
Yes	One primary only	22
Yes	One primary only	23
Yes	One primary only	21
Yes	One primary only	11
Yes	One primary only	8
Yes	One primary only	8
Yes	One primary only	14
Yes	One primary only	3
Yes	One primary only	5
Yes	One primary only	3
Yes	1st of 2 or more primaries	191
Yes	One primary only	235
Yes	One primary only	0
Yes	One primary only	10
Yes	One primary only	13
Yes	One primary only	212
Yes	1st of 2 or more primaries	215
Yes	One primary only	78
Yes	One primary only	214
Yes	One primary only	62
Yes	One primary only	210
Yes	One primary only	7
Yes	One primary only	11
Yes	One primary only	204
Yes	One primary only	203
Yes	One primary only	19
Yes	One primary only	194
Yes	One primary only	22
Yes	One primary only	178
Yes	One primary only	16
Yes	One primary only	176
Yes	One primary only	93
Yes	One primary only	27
No/Unknown	2nd of 2 or more primaries	17
Yes	One primary only	165
Yes	One primary only	40
Yes	One primary only	31
Yes	1st of 2 or more primaries	138
Yes	One primary only	20
Yes	2nd of 2 or more primaries	129
Yes	One primary only	118
Yes	One primary only	106
Yes	One primary only	33
Yes	One primary only	36
Yes	One primary only	86
Yes	One primary only	102
Yes	One primary only	4
Yes	One primary only	82
Yes	One primary only	74
Yes	One primary only	72
Yes	One primary only	62
Yes	One primary only	23
Yes	One primary only	48
Yes	One primary only	63
Yes	One primary only	54

Yes	One primary only	45
No/Unknown	2nd of 2 or more primaries	2
Yes	One primary only	58
Yes	One primary only	48
Yes	One primary only	46
Yes	One primary only	42
Yes	One primary only	25
Yes	One primary only	34
No/Unknown	One primary only	36
Yes	One primary only	31
No/Unknown	1st of 2 or more primaries	16
Yes	One primary only	14
Yes	One primary only	11
No/Unknown	One primary only	0
Yes	One primary only	9
Yes	One primary only	3
Yes	One primary only	4
Yes	One primary only	7
Yes	One primary only	1
Yes	One primary only	47
No/Unknown	One primary only	25
No/Unknown	One primary only	24
Yes	One primary only	93
Yes	One primary only	85
Yes	One primary only	32
Yes	One primary only	68
Yes	One primary only	10
Yes	One primary only	29
Yes	One primary only	87
Yes	One primary only	13
Yes	One primary only	59
Yes	One primary only	8
Yes	One primary only	214
Yes	One primary only	187
Yes	One primary only	46
Yes	One primary only	26
Yes	One primary only	15
Yes	One primary only	162
Yes	One primary only	43
Yes	One primary only	16
Yes	One primary only	37
Yes	One primary only	7
Yes	2nd of 2 or more primaries	3
Yes	One primary only	19
Yes	One primary only	154
Yes	One primary only	148
Yes	One primary only	147
Yes	One primary only	141
Yes	One primary only	140
Yes	One primary only	28
Yes	One primary only	135
Yes	One primary only	137
Yes	One primary only	135
Yes	One primary only	129
Yes	One primary only	39
Yes	One primary only	125
Yes	One primary only	26
Yes	One primary only	113

Yes	One primary only	64
Yes	One primary only	42
Yes	One primary only	11
No/Unknown	One primary only	17
Yes	One primary only	100
Yes	One primary only	96
Yes	One primary only	15
Yes	One primary only	19
Yes	One primary only	79
Yes	One primary only	72
Yes	One primary only	31
Yes	One primary only	62
Yes	One primary only	58
Yes	One primary only	55
Yes	One primary only	51
Yes	One primary only	35
Yes	One primary only	39
Yes	One primary only	43
Yes	One primary only	4
Yes	One primary only	25
Yes	One primary only	7
No/Unknown	One primary only	5
Yes	One primary only	21
Yes	One primary only	1
Yes	One primary only	9
Yes	One primary only	5
Yes	One primary only	4
Yes	One primary only	5
Yes	2nd of 2 or more primaries	199
Yes	2nd of 2 or more primaries	147
Yes	One primary only	30
Yes	One primary only	196
Yes	One primary only	76
Yes	One primary only	40
Yes	One primary only	31
Yes	One primary only	232
Yes	One primary only	7
Yes	1st of 2 or more primaries	28
Yes	One primary only	61
Yes	One primary only	227
Yes	One primary only	214
Yes	One primary only	5
Yes	One primary only	4
Yes	One primary only	19
Yes	One primary only	29
Yes	One primary only	233
Yes	2nd of 2 or more primaries	97
Yes	One primary only	220
Yes	One primary only	206
Yes	One primary only	235
Yes	One primary only	48
Yes	One primary only	215
Yes	One primary only	13
Yes	One primary only	210
Yes	One primary only	202
No/Unknown	One primary only	105
Yes	One primary only	54
Yes	2nd of 2 or more primaries	20

Yes	One primary only	10
Yes	One primary only	14
Yes	1st of 2 or more primaries	166
No/Unknown	1st of 2 or more primaries	39
Yes	One primary only	51
Yes	One primary only	195
Yes	One primary only	191
Yes	One primary only	101
Yes	One primary only	50
Yes	One primary only	10
Yes	1st of 2 or more primaries	113
Yes	One primary only	38
Yes	One primary only	181
Yes	One primary only	144
Yes	One primary only	181
Yes	One primary only	182
Yes	One primary only	182
Yes	One primary only	181
Yes	1st of 2 or more primaries	188
Yes	2nd of 2 or more primaries	68
Yes	One primary only	178
Yes	One primary only	3
Yes	One primary only	176
Yes	One primary only	170
Yes	One primary only	6
Yes	One primary only	150
Yes	One primary only	170
Yes	One primary only	170
Yes	One primary only	13
Yes	One primary only	54
Yes	One primary only	15
No/Unknown	One primary only	164
No/Unknown	One primary only	0
Yes	One primary only	161
Yes	1st of 2 or more primaries	77
Yes	1st of 2 or more primaries	161
Yes	2nd of 2 or more primaries	59
Yes	One primary only	161
Yes	One primary only	15
Yes	One primary only	158
Yes	One primary only	153
Yes	One primary only	20
Yes	One primary only	86
Yes	One primary only	154
Yes	One primary only	33
Yes	One primary only	154
No/Unknown	One primary only	10
Yes	One primary only	150
Yes	One primary only	47
Yes	One primary only	3
Yes	One primary only	150
Yes	One primary only	147
Yes	One primary only	78
Yes	One primary only	12
No/Unknown	One primary only	6
Yes	One primary only	138
Yes	One primary only	16
No/Unknown	One primary only	48

Yes	One primary only	11
Yes	One primary only	22
Yes	One primary only	43
Yes	One primary only	135
Yes	One primary only	128
Yes	One primary only	134
Yes	One primary only	108
Yes	One primary only	40
No/Unknown	One primary only	2
Yes	One primary only	127
Yes	One primary only	132
Yes	One primary only	126
Yes	One primary only	123
Yes	One primary only	123
Yes	One primary only	30
Yes	One primary only	119
Yes	One primary only	121
Yes	One primary only	119
Yes	One primary only	24
Yes	One primary only	70
Yes	One primary only	14
Yes	One primary only	116
Yes	One primary only	116
Yes	One primary only	113
Yes	One primary only	110
Yes	2nd of 2 or more primaries	14
Yes	One primary only	107
Yes	One primary only	101
Yes	One primary only	22
Yes	One primary only	102
No/Unknown	One primary only	75
Yes	One primary only	27
Yes	One primary only	102
Yes	1st of 2 or more primaries	20
Yes	One primary only	58
Yes	One primary only	38
Yes	2nd of 2 or more primaries	43
Yes	One primary only	95
Yes	One primary only	12
Yes	One primary only	10
No/Unknown	One primary only	0
No/Unknown	2nd of 2 or more primaries	0
No/Unknown	One primary only	161
Yes	One primary only	6
Yes	One primary only	78
Yes	One primary only	4
Yes	One primary only	78
Yes	One primary only	124
Yes	One primary only	19
Yes	One primary only	10
No/Unknown	One primary only	5
Yes	2nd of 2 or more primaries	9
Yes	1st of 2 or more primaries	42
Yes	One primary only	71
Yes	One primary only	76
Yes	One primary only	75
No/Unknown	2nd of 2 or more primaries	8
Yes	One primary only	67

Yes	One primary only	35
No/Unknown	One primary only	7
Yes	One primary only	64
Yes	One primary only	63
Yes	One primary only	9
No/Unknown	One primary only	1
Yes	One primary only	21
Yes	One primary only	55
Yes	One primary only	5
Yes	One primary only	43
Yes	One primary only	54
Yes	One primary only	53
Yes	One primary only	53
Yes	One primary only	51
Yes	One primary only	49
Yes	One primary only	18
Yes	One primary only	10
Yes	One primary only	51
No/Unknown	One primary only	0
No/Unknown	One primary only	1
Yes	One primary only	9
Yes	One primary only	43
Yes	One primary only	44
Yes	One primary only	42
Yes	One primary only	47
Yes	One primary only	42
Yes	One primary only	42
Yes	One primary only	38
Yes	One primary only	11
Yes	One primary only	40
Yes	One primary only	39
Yes	One primary only	24
Yes	One primary only	15
Yes	One primary only	27
Yes	One primary only	9
No/Unknown	2nd of 2 or more primaries	0
Yes	One primary only	29
Yes	One primary only	30
Yes	One primary only	27
Yes	One primary only	24
Yes	One primary only	24
Yes	One primary only	11
No/Unknown	One primary only	5
Yes	One primary only	20
Yes	1st of 2 or more primaries	23
Yes	One primary only	23
Yes	One primary only	13
Yes	One primary only	20
Yes	One primary only	15
Yes	One primary only	13
Yes	One primary only	19
Yes	One primary only	18
Yes	1st of 2 or more primaries	6
Yes	One primary only	5
Yes	One primary only	2
Yes	One primary only	4
Yes	One primary only	8
Yes	One primary only	10

Yes	One primary only	0
Yes	One primary only	0
No/Unknown	One primary only	Unknown
Yes	One primary only	13
Yes	1st of 2 or more primaries	13
Yes	One primary only	55
Yes	One primary only	21
Yes	One primary only	35
Yes	One primary only	233
Yes	One primary only	28
Yes	One primary only	224
Yes	One primary only	26
Yes	One primary only	12
Yes	One primary only	231
Yes	One primary only	34
Yes	One primary only	18
Yes	One primary only	213
Yes	One primary only	235
Yes	One primary only	226
Yes	2nd of 2 or more primaries	150
Yes	One primary only	28
Yes	One primary only	21
Yes	One primary only	26
Yes	One primary only	218
Yes	2nd of 2 or more primaries	20
Yes	One primary only	224
Yes	One primary only	4
No/Unknown	One primary only	232
No/Unknown	2nd of 2 or more primaries	112
Yes	One primary only	237
Yes	One primary only	235
Yes	One primary only	235
Yes	One primary only	147
Yes	One primary only	77
Yes	One primary only	25
Yes	One primary only	19
Yes	One primary only	208
Yes	One primary only	17
Yes	3rd of 3 or more primaries	73
Yes	One primary only	215
Yes	One primary only	18
Yes	1st of 2 or more primaries	94
Yes	2nd of 2 or more primaries	12
Yes	One primary only	61
Yes	One primary only	159
Yes	One primary only	9
Yes	One primary only	221
Yes	One primary only	210
Yes	2nd of 2 or more primaries	84
Yes	One primary only	78
Yes	One primary only	25
No/Unknown	One primary only	62
Yes	One primary only	35
Yes	One primary only	79
Yes	One primary only	237
Yes	One primary only	11
Yes	One primary only	229
Yes	One primary only	67

Yes	One primary only	33
Yes	One primary only	23
Yes	One primary only	220
Yes	One primary only	213
Yes	One primary only	68
Yes	One primary only	99
Yes	One primary only	48
Yes	One primary only	206
Yes	One primary only	30
Yes	One primary only	215
Yes	1st of 2 or more primaries	148
No/Unknown	One primary only	206
Yes	One primary only	25
Yes	One primary only	202
Yes	One primary only	215
Yes	One primary only	208
Yes	One primary only	28
No/Unknown	One primary only	10
Yes	One primary only	206
Yes	One primary only	197
Yes	One primary only	182
Yes	1st of 2 or more primaries	199
Yes	One primary only	8
Yes	One primary only	195
Yes	2nd of 2 or more primaries	50
Yes	One primary only	26
Yes	One primary only	27
Yes	One primary only	211
Yes	One primary only	20
Yes	One primary only	16
Yes	One primary only	198
Yes	One primary only	3
Yes	One primary only	16
Yes	One primary only	197
Yes	One primary only	208
Yes	1st of 2 or more primaries	11
Yes	One primary only	10
Yes	One primary only	37
Yes	One primary only	19
Yes	One primary only	14
Yes	2nd of 2 or more primaries	91
No/Unknown	One primary only	0
Yes	One primary only	192
Yes	One primary only	194
Yes	One primary only	11
Yes	One primary only	106
Yes	One primary only	183
Yes	One primary only	187
Yes	One primary only	186
Yes	One primary only	185
Yes	1st of 2 or more primaries	191
No/Unknown	2nd of 2 or more primaries	5
Yes	One primary only	14
Yes	One primary only	3
Yes	One primary only	182
Yes	One primary only	7
Yes	One primary only	53
Yes	One primary only	183

No/Unknown	One primary only	188
No/Unknown	One primary only	182
Yes	One primary only	184
Yes	One primary only	9
Yes	One primary only	181
Yes	One primary only	184
Yes	One primary only	97
Yes	One primary only	174
Yes	One primary only	28
Yes	One primary only	37
Yes	1st of 2 or more primaries	15
Yes	One primary only	119
Yes	One primary only	30
Yes	One primary only	22
Yes	One primary only	41
Yes	One primary only	170
Yes	One primary only	174
No/Unknown	One primary only	7
Yes	One primary only	2
Yes	One primary only	8
Yes	1st of 2 or more primaries	178
Yes	One primary only	27
Yes	One primary only	29
Yes	One primary only	172
Yes	One primary only	61
Yes	One primary only	171
Yes	One primary only	15
Yes	One primary only	171
Yes	One primary only	169
No/Unknown	One primary only	19
Yes	One primary only	168
No/Unknown	One primary only	198
Yes	One primary only	16
Yes	One primary only	10
Yes	One primary only	163
Yes	One primary only	22
Yes	One primary only	165
Yes	One primary only	6
Yes	One primary only	160
Yes	One primary only	20
Yes	One primary only	162
Yes	One primary only	28
Yes	One primary only	165
Yes	One primary only	86
Yes	One primary only	11
Yes	One primary only	155
Yes	One primary only	33
Yes	One primary only	58
Yes	One primary only	5
Yes	One primary only	152
Yes	One primary only	12
Yes	One primary only	12
No/Unknown	One primary only	9
Yes	One primary only	1
Yes	One primary only	29
Yes	One primary only	151
Yes	One primary only	44
Yes	One primary only	8

Yes	One primary only	154
Yes	One primary only	149
Yes	One primary only	150
Yes	One primary only	29
Yes	One primary only	12
Yes	One primary only	151
Yes	One primary only	13
Yes	One primary only	149
Yes	One primary only	147
Yes	One primary only	69
Yes	One primary only	146
Yes	One primary only	85
No/Unknown	One primary only	96
Yes	One primary only	148
Yes	One primary only	7
No/Unknown	2nd of 2 or more primaries	1
Yes	One primary only	148
Yes	One primary only	35
Yes	One primary only	11
No/Unknown	One primary only	214
Yes	One primary only	145
Yes	One primary only	2
Yes	One primary only	39
Yes	2nd of 2 or more primaries	111
No/Unknown	One primary only	128
Yes	1st of 2 or more primaries	99
Yes	One primary only	134
Yes	One primary only	26
Yes	One primary only	135
Yes	One primary only	133
Yes	One primary only	10
Yes	One primary only	137
Yes	One primary only	138
Yes	One primary only	34
Yes	One primary only	132
Yes	One primary only	134
Yes	One primary only	130
Yes	One primary only	131
Yes	One primary only	123
Yes	One primary only	128
Yes	One primary only	36
Yes	One primary only	18
Yes	One primary only	45
Yes	One primary only	9
Yes	One primary only	49
Yes	One primary only	126
Yes	One primary only	10
Yes	One primary only	33
Yes	One primary only	123
Yes	One primary only	10
Yes	One primary only	123
Yes	2nd of 2 or more primaries	5
Yes	One primary only	9
Yes	One primary only	120
Yes	One primary only	9
Yes	One primary only	27
Yes	One primary only	118
Yes	1st of 2 or more primaries	167

Yes	One primary only	117
Yes	One primary only	119
Yes	1st of 2 or more primaries	20
Yes	1st of 2 or more primaries	54
Yes	One primary only	113
Yes	One primary only	119
Yes	1st of 2 or more primaries	50
Yes	One primary only	37
Yes	One primary only	116
Yes	One primary only	108
Yes	One primary only	106
Yes	One primary only	21
Yes	One primary only	94
Yes	One primary only	104
Yes	One primary only	11
Yes	One primary only	104
Yes	One primary only	108
Yes	One primary only	98
Yes	One primary only	107
Yes	One primary only	68
Yes	One primary only	8
No/Unknown	One primary only	5
Yes	One primary only	12
Yes	One primary only	28
Yes	One primary only	97
No/Unknown	One primary only	0
Yes	One primary only	29
Yes	One primary only	34
Yes	One primary only	28
Yes	One primary only	22
Yes	One primary only	69
Yes	One primary only	15
Yes	One primary only	100
Yes	One primary only	52
Yes	One primary only	95
Yes	One primary only	94
Yes	One primary only	96
Yes	One primary only	30
Yes	One primary only	94
Yes	One primary only	68
Yes	One primary only	94
Yes	One primary only	95
Yes	One primary only	12
Yes	One primary only	94
Yes	One primary only	5
Yes	One primary only	19
Yes	1st of 2 or more primaries	58
Yes	One primary only	48
Yes	One primary only	95
Yes	One primary only	2
Yes	One primary only	48
Yes	One primary only	38
Yes	One primary only	90
Yes	One primary only	37
Yes	One primary only	12
No/Unknown	One primary only	40
Yes	One primary only	84
Yes	1st of 2 or more primaries	82

Yes	One primary only	82
Yes	1st of 2 or more primaries	56
Yes	One primary only	82
Yes	One primary only	45
Yes	One primary only	82
Yes	One primary only	10
Yes	One primary only	39
Yes	One primary only	5
Yes	1st of 2 or more primaries	78
Yes	One primary only	77
Yes	One primary only	76
Yes	One primary only	11
Yes	One primary only	61
Yes	One primary only	76
Yes	One primary only	57
Yes	One primary only	65
No/Unknown	One primary only	1
Yes	One primary only	73
Yes	One primary only	41
Yes	One primary only	70
Yes	One primary only	67
Yes	One primary only	70
Yes	One primary only	69
Yes	One primary only	69
Yes	1st of 2 or more primaries	7
Yes	One primary only	3
Yes	One primary only	30
Yes	1st of 2 or more primaries	50
Yes	1st of 2 or more primaries	37
Yes	One primary only	32
Yes	2nd of 2 or more primaries	14
Yes	One primary only	30
Yes	One primary only	63
Yes	One primary only	56
Yes	One primary only	12
Yes	One primary only	72
Yes	1st of 2 or more primaries	58
Yes	One primary only	35
Yes	One primary only	23
Yes	1st of 2 or more primaries	31
Yes	One primary only	16
Yes	1st of 2 or more primaries	12
Yes	One primary only	60
Yes	One primary only	25
Yes	One primary only	4
Yes	One primary only	59
Yes	One primary only	31
Yes	One primary only	23
Yes	One primary only	58
Yes	One primary only	56
Yes	One primary only	29
Yes	1st of 2 or more primaries	71
Yes	2nd of 2 or more primaries	56
Yes	3rd of 3 or more primaries	56
Yes	One primary only	55
Yes	One primary only	27
No/Unknown	One primary only	4
Yes	One primary only	56

Yes	One primary only	43
Yes	One primary only	54
Yes	One primary only	48
Yes	One primary only	55
Yes	One primary only	23
Yes	One primary only	56
No/Unknown	One primary only	52
Yes	One primary only	51
No/Unknown	2nd of 2 or more primaries	2
Yes	One primary only	50
Yes	One primary only	50
Yes	One primary only	38
Yes	One primary only	53
Yes	One primary only	48
Yes	One primary only	51
Yes	One primary only	48
Yes	One primary only	49
No/Unknown	One primary only	16
Yes	One primary only	9
Yes	2nd of 2 or more primaries	10
Yes	One primary only	13
Yes	One primary only	41
Yes	One primary only	34
Yes	One primary only	45
Yes	One primary only	41
Yes	One primary only	18
Yes	One primary only	44
Yes	One primary only	43
Yes	1st of 2 or more primaries	197
Yes	One primary only	37
Yes	One primary only	18
Yes	One primary only	18
Yes	One primary only	44
Yes	One primary only	24
Yes	One primary only	27
Yes	One primary only	20
Yes	One primary only	37
Yes	One primary only	39
Yes	One primary only	37
Yes	One primary only	32
Yes	One primary only	36
Yes	One primary only	37
Yes	One primary only	25
Yes	One primary only	24
Yes	One primary only	34
Yes	One primary only	8
Yes	One primary only	13
Yes	One primary only	34
Yes	One primary only	29
Yes	One primary only	28
Yes	One primary only	31
Yes	One primary only	15
Yes	One primary only	29
Yes	One primary only	28
Yes	One primary only	30
Yes	One primary only	32
Yes	One primary only	21
Yes	1st of 2 or more primaries	25

Yes	One primary only	29
Yes	One primary only	6
Yes	One primary only	17
No/Unknown	One primary only	30
Yes	One primary only	8
Yes	One primary only	24
No/Unknown	One primary only	29
Yes	One primary only	47
Yes	One primary only	53
Yes	One primary only	19
Yes	One primary only	13
Yes	One primary only	19
Yes	One primary only	21
Yes	One primary only	9
Yes	One primary only	13
Yes	One primary only	22
Yes	One primary only	21
Yes	One primary only	19
Yes	One primary only	19
Yes	One primary only	14
Yes	One primary only	12
Yes	One primary only	17
Yes	One primary only	17
Yes	One primary only	5
Yes	One primary only	11
No/Unknown	One primary only	17
Yes	One primary only	13
Yes	One primary only	9
Yes	One primary only	13
Yes	One primary only	9
Yes	One primary only	10
Yes	One primary only	10
Yes	One primary only	16
Yes	One primary only	6
Yes	One primary only	5
Yes	One primary only	5
Yes	One primary only	11
Yes	One primary only	18
Yes	One primary only	1
Yes	One primary only	7
Yes	One primary only	7
Yes	One primary only	7
Yes	One primary only	5
Yes	One primary only	12
No/Unknown	One primary only	8
Yes	One primary only	3
Yes	One primary only	2
Yes	One primary only	6
Yes	1st of 2 or more primaries	6
Yes	One primary only	5
Yes	One primary only	7
Yes	One primary only	9
Yes	One primary only	1
Yes	One primary only	1
Yes	One primary only	5
Yes	One primary only	6
Yes	One primary only	228
Yes	One primary only	225

Yes	One primary only	79
Yes	One primary only	223
Yes	One primary only	6
Yes	One primary only	215
Yes	One primary only	20
Yes	One primary only	92
Yes	One primary only	205
Yes	One primary only	213
Yes	One primary only	16
Yes	One primary only	201
Yes	One primary only	7
No/Unknown	One primary only	35
Yes	One primary only	56
Yes	2nd of 2 or more primaries	24
Yes	One primary only	21
Yes	One primary only	74
Yes	One primary only	40
No/Unknown	One primary only	199
Yes	One primary only	12
Yes	One primary only	77
Yes	One primary only	183
Yes	One primary only	195
Yes	One primary only	52
Yes	One primary only	168
Yes	1st of 2 or more primaries	156
Yes	One primary only	13
Yes	1st of 2 or more primaries	167
Yes	One primary only	165
Yes	One primary only	143
Yes	One primary only	42
Yes	One primary only	164
Yes	One primary only	14
Yes	One primary only	19
Yes	One primary only	11
Yes	One primary only	143
Yes	One primary only	138
Yes	One primary only	22
Yes	One primary only	24
No/Unknown	One primary only	31
Yes	One primary only	56
Yes	One primary only	136
Yes	One primary only	152
Yes	One primary only	140
Yes	One primary only	153
Yes	One primary only	27
Yes	One primary only	108
Yes	One primary only	122
Yes	One primary only	24
Yes	One primary only	109
Yes	One primary only	101
Yes	One primary only	101
Yes	1st of 2 or more primaries	30
Yes	One primary only	97
Yes	One primary only	19
Yes	One primary only	55
Yes	One primary only	92
Yes	One primary only	44
Yes	One primary only	89

Yes	One primary only	91
Yes	One primary only	89
Yes	One primary only	87
Yes	One primary only	87
Yes	One primary only	87
Yes	One primary only	12
Yes	One primary only	15
Yes	One primary only	84
Yes	One primary only	81
Yes	One primary only	57
Yes	One primary only	51
Yes	One primary only	48
Yes	One primary only	14
Yes	One primary only	85
No/Unknown	3rd of 3 or more primaries	0
Yes	One primary only	40
Yes	One primary only	61
Yes	One primary only	58
Yes	One primary only	72
Yes	One primary only	65
Yes	One primary only	61
Yes	One primary only	14
Yes	One primary only	24
Yes	One primary only	48
Yes	One primary only	8
Yes	One primary only	42
Yes	One primary only	42
Yes	One primary only	30
Yes	One primary only	39
Yes	1st of 2 or more primaries	39
Yes	One primary only	37
Yes	One primary only	36
Yes	One primary only	37
Yes	One primary only	33
Yes	One primary only	6
No/Unknown	One primary only	6
Yes	One primary only	31
No/Unknown	One primary only	26
Yes	One primary only	34
Yes	One primary only	29
Yes	One primary only	7
Yes	One primary only	24
Yes	One primary only	4
Yes	One primary only	11
Yes	One primary only	8
Yes	One primary only	10
Yes	One primary only	5
No/Unknown	2nd of 2 or more primaries	2
Yes	One primary only	16
Yes	One primary only	20
Yes	One primary only	10
Yes	One primary only	223
Yes	One primary only	10
Yes	One primary only	29
Yes	One primary only	17
Yes	One primary only	14
Yes	1st of 2 or more primaries	77
Yes	One primary only	22

Yes	One primary only	143
Yes	One primary only	149
No/Unknown	One primary only	4
Yes	One primary only	27
Yes	One primary only	57
Yes	One primary only	165
Yes	1st of 2 or more primaries	210
Yes	2nd of 2 or more primaries	126
Yes	2nd of 2 or more primaries	34
No/Unknown	One primary only	15
Yes	One primary only	17
Yes	One primary only	159
Yes	One primary only	12
Yes	One primary only	8
Yes	One primary only	201
Yes	One primary only	170
Yes	One primary only	227
Yes	One primary only	6
Yes	One primary only	138
Yes	One primary only	122
Yes	One primary only	28
Yes	One primary only	129
Yes	One primary only	131
Yes	One primary only	121
Yes	2nd of 2 or more primaries	80
Yes	One primary only	124
Yes	One primary only	7
Yes	One primary only	16
Yes	One primary only	64
Yes	One primary only	107
Yes	One primary only	72
Yes	2nd of 2 or more primaries	42
Yes	One primary only	105
Yes	One primary only	15
Yes	One primary only	22
Yes	One primary only	101
Yes	One primary only	93
Yes	One primary only	21
Yes	1st of 2 or more primaries	83
Yes	One primary only	28
No/Unknown	One primary only	12
No/Unknown	One primary only	95
Yes	One primary only	9
Yes	One primary only	65
Yes	One primary only	46
Yes	One primary only	62
Yes	One primary only	59
Yes	One primary only	73
Yes	1st of 2 or more primaries	65
No/Unknown	One primary only	56
Yes	One primary only	67
Yes	One primary only	11
No/Unknown	One primary only	23
No/Unknown	One primary only	161
Yes	One primary only	58
No/Unknown	One primary only	31
Yes	One primary only	11
Yes	One primary only	44

Yes	One primary only	41
Yes	One primary only	31
Yes	One primary only	39
Yes	One primary only	11
Yes	One primary only	37
Yes	One primary only	24
No/Unknown	One primary only	1
Yes	One primary only	13
Yes	One primary only	25
Yes	One primary only	18
Yes	One primary only	15
Yes	One primary only	15
No/Unknown	One primary only	9
Yes	One primary only	13
Yes	One primary only	15
Yes	One primary only	18
Yes	One primary only	14
Yes	One primary only	7
Yes	One primary only	4
Yes	One primary only	1
Yes	One primary only	0
Yes	2nd of 2 or more primaries	33
Yes	2nd of 2 or more primaries	135
Yes	3rd of 3 or more primaries	100
Yes	1st of 2 or more primaries	166
No/Unknown	2nd of 2 or more primaries	21
Yes	One primary only	16
Yes	One primary only	12
No/Unknown	One primary only	237
No/Unknown	One primary only	25
Yes	One primary only	233
No/Unknown	One primary only	0
Yes	One primary only	40
Yes	One primary only	10
Yes	One primary only	226
Yes	One primary only	9
Yes	One primary only	17
Yes	One primary only	221
Yes	One primary only	221
Yes	One primary only	27
Yes	1st of 2 or more primaries	35
Yes	One primary only	225
Yes	One primary only	13
Yes	One primary only	215
Yes	One primary only	212
Yes	One primary only	30
Yes	One primary only	208
Yes	One primary only	49
Yes	One primary only	87
Yes	One primary only	204
Yes	One primary only	21
Yes	One primary only	210
Yes	One primary only	211
Yes	One primary only	3
Yes	One primary only	214
Yes	One primary only	16
Yes	One primary only	21
Yes	1st of 2 or more primaries	198

Yes	One primary only	74
No/Unknown	One primary only	203
Yes	One primary only	205
Yes	One primary only	205
Yes	2nd of 2 or more primaries	18
Yes	One primary only	79
Yes	One primary only	200
Yes	One primary only	122
No/Unknown	2nd of 2 or more primaries	2
Yes	One primary only	13
Yes	One primary only	2
Yes	One primary only	192
Yes	One primary only	52
Yes	One primary only	16
Yes	One primary only	76
Yes	1st of 2 or more primaries	190
Yes	One primary only	195
Yes	One primary only	92
Yes	One primary only	188
Yes	One primary only	213
Yes	One primary only	11
Yes	One primary only	67
Yes	One primary only	49
Yes	One primary only	95
Yes	One primary only	190
Yes	One primary only	22
Yes	One primary only	45
Yes	2nd of 2 or more primaries	34
Yes	One primary only	174
Yes	One primary only	55
Yes	One primary only	173
No/Unknown	One primary only	8
No/Unknown	One primary only	170
Yes	One primary only	56
Yes	One primary only	11
Yes	One primary only	188
Yes	One primary only	51
Yes	One primary only	99
Yes	One primary only	170
Yes	One primary only	12
Yes	4th of 4 or more primaries	77
Yes	One primary only	163
Yes	One primary only	170
Yes	One primary only	26
Yes	One primary only	163
Yes	2nd of 2 or more primaries	102
Yes	One primary only	27
Yes	One primary only	157
Yes	One primary only	144
Yes	One primary only	12
Yes	One primary only	163
Yes	One primary only	44
No/Unknown	One primary only	59
Yes	One primary only	75
Yes	One primary only	130
Yes	One primary only	155
Yes	One primary only	10
Yes	One primary only	156

Yes	One primary only	165
Yes	One primary only	174
Yes	One primary only	149
Yes	One primary only	148
Yes	One primary only	144
Yes	One primary only	53
Yes	One primary only	109
Yes	One primary only	44
Yes	One primary only	140
Yes	One primary only	142
Yes	One primary only	134
Yes	One primary only	14
Yes	One primary only	8
Yes	One primary only	52
Yes	One primary only	155
Yes	One primary only	135
No/Unknown	One primary only	132
Yes	One primary only	133
Yes	One primary only	149
Yes	One primary only	138
Yes	One primary only	135
Yes	One primary only	103
Yes	4th of 4 or more primaries	40
Yes	One primary only	128
Yes	One primary only	86
Yes	One primary only	43
No/Unknown	One primary only	47
Yes	One primary only	123
Yes	One primary only	125
No/Unknown	One primary only	5
Yes	One primary only	32
Yes	One primary only	120
Yes	One primary only	114
Yes	One primary only	120
Yes	One primary only	47
Yes	One primary only	9
Yes	One primary only	2
Yes	One primary only	120
Yes	One primary only	115
Yes	One primary only	16
Yes	One primary only	119
Yes	One primary only	28
Yes	One primary only	2
Yes	One primary only	110
No/Unknown	One primary only	30
Yes	One primary only	8
Yes	One primary only	104
Yes	One primary only	23
Yes	One primary only	22
Yes	One primary only	106
Yes	One primary only	19
Yes	One primary only	110
Yes	One primary only	108
Yes	One primary only	59
Yes	One primary only	104
Yes	One primary only	14
Yes	One primary only	15
Yes	One primary only	104

Yes	One primary only	22
Yes	One primary only	91
Yes	One primary only	107
Yes	One primary only	107
Yes	1st of 2 or more primaries	24
Yes	2nd of 2 or more primaries	73
Yes	One primary only	9
Yes	2nd of 2 or more primaries	67
Yes	One primary only	89
Yes	One primary only	84
Yes	One primary only	71
Yes	One primary only	16
Yes	1st of 2 or more primaries	84
Yes	One primary only	10
Yes	One primary only	85
Yes	One primary only	30
Yes	One primary only	22
Yes	One primary only	21
Yes	One primary only	79
Yes	2nd of 2 or more primaries	20
No/Unknown	2nd of 2 or more primaries	0
Yes	One primary only	76
Yes	One primary only	73
Yes	2nd of 2 or more primaries	71
Yes	One primary only	75
Yes	1st of 2 or more primaries	31
Yes	One primary only	2
Yes	One primary only	64
Yes	1st of 2 or more primaries	68
Yes	2nd of 2 or more primaries	10
Yes	One primary only	65
Yes	One primary only	61
No/Unknown	One primary only	12
Yes	One primary only	68
Yes	One primary only	61
Yes	One primary only	30
Yes	One primary only	62
Yes	One primary only	65
Yes	One primary only	59
Yes	One primary only	61
Yes	One primary only	17
Yes	One primary only	60
Yes	One primary only	53
Yes	One primary only	54
Yes	One primary only	50
Yes	One primary only	56
Yes	One primary only	4
Yes	One primary only	46
Yes	One primary only	51
Yes	One primary only	16
Yes	3rd of 3 or more primaries	5
Yes	One primary only	44
Yes	One primary only	43
Yes	One primary only	16
Yes	One primary only	40
Yes	One primary only	31
Yes	One primary only	41
Yes	One primary only	38

Yes	One primary only	22
Yes	One primary only	36
Yes	One primary only	41
Yes	One primary only	31
Yes	One primary only	12
Yes	One primary only	27
Yes	One primary only	11
Yes	One primary only	34
Yes	One primary only	9
Yes	One primary only	35
Yes	One primary only	40
Yes	One primary only	8
Yes	One primary only	34
Yes	One primary only	10
Yes	One primary only	32
Yes	One primary only	12
Yes	One primary only	20
No/Unknown	One primary only	29
Yes	One primary only	20
Yes	One primary only	15
Yes	One primary only	24
Yes	One primary only	25
Yes	One primary only	16
Yes	One primary only	13
Yes	One primary only	14
Yes	One primary only	22
Yes	1st of 2 or more primaries	22
Yes	One primary only	20
Yes	One primary only	13
Yes	One primary only	12
Yes	One primary only	14
No/Unknown	One primary only	3
No/Unknown	One primary only	173
Yes	One primary only	15
Yes	One primary only	16
Yes	One primary only	3
Yes	One primary only	10
Yes	One primary only	3
Yes	One primary only	6
Yes	One primary only	4
Yes	3rd of 3 or more primaries	5
Yes	One primary only	8
Yes	One primary only	5
No/Unknown	One primary only	1
Yes	One primary only	6
Yes	One primary only	8
Yes	One primary only	5
Yes	One primary only	5
Yes	One primary only	29
Yes	One primary only	215
Yes	One primary only	238
Yes	One primary only	235
Yes	One primary only	235
Yes	One primary only	12
Yes	One primary only	226
Yes	One primary only	226
Yes	1st of 2 or more primaries	155
Yes	One primary only	219

No/Unknown	2nd of 2 or more primaries	6
No/Unknown	One primary only	85
Yes	1st of 2 or more primaries	75
Yes	2nd of 2 or more primaries	105
No/Unknown	3rd of 3 or more primaries	95
Yes	One primary only	212
No/Unknown	One primary only	230
Yes	One primary only	68
Yes	One primary only	10
Yes	One primary only	222
Yes	One primary only	73
Yes	One primary only	95
Yes	One primary only	102
Yes	One primary only	23
Yes	One primary only	199
Yes	2nd of 2 or more primaries	14
Yes	One primary only	193
Yes	One primary only	184
Yes	One primary only	11
Yes	One primary only	196
Yes	One primary only	177
Yes	One primary only	62
Yes	One primary only	21
Yes	One primary only	16
Yes	One primary only	174
No/Unknown	One primary only	179
Yes	One primary only	45
Yes	One primary only	169
Yes	One primary only	60
Yes	One primary only	187
Yes	One primary only	41
Yes	1st of 2 or more primaries	77
Yes	2nd of 2 or more primaries	20
Yes	One primary only	65
No/Unknown	One primary only	16
Yes	One primary only	0
Yes	One primary only	157
Yes	One primary only	34
Yes	One primary only	179
Yes	One primary only	146
Yes	One primary only	140
Yes	One primary only	159
Yes	One primary only	152
Yes	One primary only	148
Yes	One primary only	45
Yes	One primary only	7
Yes	One primary only	18
Yes	2nd of 2 or more primaries	18
Yes	One primary only	133
No/Unknown	One primary only	33
Yes	One primary only	62
Yes	One primary only	140
Yes	One primary only	137
No/Unknown	One primary only	169
Yes	One primary only	85
Yes	One primary only	114
Yes	One primary only	85
Yes	One primary only	39

Yes	One primary only	22
Yes	One primary only	123
Yes	One primary only	123
Yes	One primary only	59
Yes	2nd of 2 or more primaries	14
Yes	One primary only	106
Yes	One primary only	93
Yes	One primary only	46
Yes	One primary only	39
Yes	One primary only	112
Yes	One primary only	32
Yes	One primary only	98
Yes	One primary only	98
Yes	One primary only	97
Yes	One primary only	39
Yes	One primary only	102
Yes	One primary only	27
Yes	One primary only	91
Yes	One primary only	29
Yes	One primary only	92
Yes	One primary only	91
Yes	One primary only	93
Yes	One primary only	94
Yes	One primary only	92
Yes	One primary only	7
Yes	One primary only	85
Yes	One primary only	93
Yes	One primary only	85
Yes	One primary only	81
Yes	One primary only	79
Yes	One primary only	75
Yes	One primary only	42
Yes	One primary only	80
Yes	One primary only	72
Yes	2nd of 2 or more primaries	36
Yes	One primary only	72
Yes	2nd of 2 or more primaries	52
Yes	One primary only	25
Yes	One primary only	9
Yes	One primary only	10
Yes	1st of 2 or more primaries	66
Yes	One primary only	10
Yes	One primary only	101
Yes	1st of 2 or more primaries	69
Yes	One primary only	7
Yes	One primary only	68
Yes	One primary only	10
Yes	One primary only	61
Yes	1st of 2 or more primaries	60
Yes	One primary only	57
Yes	One primary only	54
Yes	1st of 2 or more primaries	64
Yes	One primary only	36
Yes	One primary only	30
Yes	One primary only	15
Yes	One primary only	22
Yes	One primary only	3
Yes	2nd of 2 or more primaries	42

Yes	One primary only	36
Yes	One primary only	18
Yes	One primary only	31
Yes	1st of 2 or more primaries	28
Yes	One primary only	29
Yes	One primary only	22
No/Unknown	One primary only	21
No/Unknown	One primary only	15
Yes	One primary only	15
Yes	One primary only	7
Yes	1st of 2 or more primaries	6
Yes	One primary only	9
Yes	One primary only	4
Yes	One primary only	9
Yes	One primary only	3
No/Unknown	One primary only	1
Yes	One primary only	10

Vital status recode	Primary Site - labeled
Alive	C41.9-Bone, NOS
Alive	C40.0-Long bones: upper limb, scapula, and associated joints
Alive	C41.3-Rib, sternum, clavicle and associated joints
Dead	C41.4-Pelvic bones, sacrum, coccyx and associated joints
Alive	C41.2-Vertebral column
Alive	C41.4-Pelvic bones, sacrum, coccyx and associated joints
Dead	C41.4-Pelvic bones, sacrum, coccyx and associated joints
Alive	C41.3-Rib, sternum, clavicle and associated joints
Alive	C40.2-Long bones of lower limb and associated joints
Alive	C40.3-Short bones of lower limb and associated joints
Dead	C41.9-Bone, NOS
Dead	C41.4-Pelvic bones, sacrum, coccyx and associated joints
Alive	C41.0-Bones of skull and face and associated joints
Dead	C41.4-Pelvic bones, sacrum, coccyx and associated joints
Dead	C41.2-Vertebral column
Alive	C40.0-Long bones: upper limb, scapula, and associated joints
Dead	C41.4-Pelvic bones, sacrum, coccyx and associated joints
Dead	C40.0-Long bones: upper limb, scapula, and associated joints
Alive	C41.4-Pelvic bones, sacrum, coccyx and associated joints
Alive	C41.3-Rib, sternum, clavicle and associated joints
Dead	C40.2-Long bones of lower limb and associated joints
Alive	C40.2-Long bones of lower limb and associated joints
Dead	C41.2-Vertebral column
Dead	C41.4-Pelvic bones, sacrum, coccyx and associated joints
Dead	C41.4-Pelvic bones, sacrum, coccyx and associated joints
Alive	C41.2-Vertebral column
Dead	C40.2-Long bones of lower limb and associated joints
Alive	C40.0-Long bones: upper limb, scapula, and associated joints
Alive	C40.2-Long bones of lower limb and associated joints
Dead	C40.0-Long bones: upper limb, scapula, and associated joints
Alive	C40.2-Long bones of lower limb and associated joints
Dead	C40.2-Long bones of lower limb and associated joints
Dead	C40.0-Long bones: upper limb, scapula, and associated joints
Dead	C41.9-Bone, NOS
Alive	C41.3-Rib, sternum, clavicle and associated joints
Alive	C40.2-Long bones of lower limb and associated joints
Alive	C41.4-Pelvic bones, sacrum, coccyx and associated joints
Alive	C41.4-Pelvic bones, sacrum, coccyx and associated joints
Alive	C41.3-Rib, sternum, clavicle and associated joints
Alive	C41.4-Pelvic bones, sacrum, coccyx and associated joints
Alive	C41.0-Bones of skull and face and associated joints
Alive	C40.2-Long bones of lower limb and associated joints
Alive	C40.0-Long bones: upper limb, scapula, and associated joints
Alive	C41.3-Rib, sternum, clavicle and associated joints
Dead	C40.2-Long bones of lower limb and associated joints
Dead	C41.4-Pelvic bones, sacrum, coccyx and associated joints
Dead	C41.3-Rib, sternum, clavicle and associated joints
Alive	C41.4-Pelvic bones, sacrum, coccyx and associated joints
Dead	C41.4-Pelvic bones, sacrum, coccyx and associated joints
Dead	C41.4-Pelvic bones, sacrum, coccyx and associated joints
Dead	C40.2-Long bones of lower limb and associated joints
Alive	C41.2-Vertebral column
Alive	C41.2-Vertebral column
Alive	C41.9-Bone, NOS
Alive	C40.2-Long bones of lower limb and associated joints
Alive	C40.0-Long bones: upper limb, scapula, and associated joints
Alive	C40.0-Long bones: upper limb, scapula, and associated joints

Dead	C40.2-Long bones of lower limb and associated joints
Alive	C41.2-Vertebral column
Dead	C40.2-Long bones of lower limb and associated joints
Dead	C40.3-Short bones of lower limb and associated joints
Dead	C40.2-Long bones of lower limb and associated joints
Alive	C41.4-Pelvic bones, sacrum, coccyx and associated joints
Dead	C41.2-Vertebral column
Alive	C41.1-Mandible
Alive	C41.2-Vertebral column
Alive	C41.3-Rib, sternum, clavicle and associated joints
Alive	C41.2-Vertebral column
Alive	C41.4-Pelvic bones, sacrum, coccyx and associated joints
Dead	C41.2-Vertebral column
Alive	C41.2-Vertebral column
Alive	C41.3-Rib, sternum, clavicle and associated joints
Alive	C41.3-Rib, sternum, clavicle and associated joints
Dead	C41.4-Pelvic bones, sacrum, coccyx and associated joints
Alive	C41.2-Vertebral column
Alive	C41.4-Pelvic bones, sacrum, coccyx and associated joints
Alive	C41.4-Pelvic bones, sacrum, coccyx and associated joints
Alive	C41.4-Pelvic bones, sacrum, coccyx and associated joints
Alive	C41.4-Pelvic bones, sacrum, coccyx and associated joints
Alive	C41.4-Pelvic bones, sacrum, coccyx and associated joints
Alive	C41.4-Pelvic bones, sacrum, coccyx and associated joints
Alive	C41.9-Bone, NOS
Alive	C40.0-Long bones: upper limb, scapula, and associated joints
Alive	C40.2-Long bones of lower limb and associated joints
Dead	C40.3-Short bones of lower limb and associated joints
Dead	C41.3-Rib, sternum, clavicle and associated joints
Alive	C41.9-Bone, NOS
Alive	C41.4-Pelvic bones, sacrum, coccyx and associated joints
Alive	C41.3-Rib, sternum, clavicle and associated joints
Alive	C40.8-Overlap of bones, joints, and art. cartilage of limbs
Dead	C41.3-Rib, sternum, clavicle and associated joints
Dead	C41.9-Bone, NOS
Alive	C40.2-Long bones of lower limb and associated joints
Dead	C41.4-Pelvic bones, sacrum, coccyx and associated joints
Dead	C40.3-Short bones of lower limb and associated joints
Alive	C41.2-Vertebral column
Dead	C41.4-Pelvic bones, sacrum, coccyx and associated joints
Alive	C41.4-Pelvic bones, sacrum, coccyx and associated joints
Alive	C41.4-Pelvic bones, sacrum, coccyx and associated joints
Alive	C41.2-Vertebral column
Alive	C41.4-Pelvic bones, sacrum, coccyx and associated joints
Dead	C41.9-Bone, NOS
Dead	C40.2-Long bones of lower limb and associated joints
Alive	C40.0-Long bones: upper limb, scapula, and associated joints
Dead	C41.9-Bone, NOS
Alive	C41.2-Vertebral column
Alive	C41.8-Overlap bones, joints, and art. cartilage
Alive	C40.3-Short bones of lower limb and associated joints
Alive	C40.2-Long bones of lower limb and associated joints
Dead	C41.4-Pelvic bones, sacrum, coccyx and associated joints
Alive	C41.3-Rib, sternum, clavicle and associated joints
Alive	C40.2-Long bones of lower limb and associated joints
Dead	C40.2-Long bones of lower limb and associated joints
Dead	C40.2-Long bones of lower limb and associated joints
Dead	C41.4-Pelvic bones, sacrum, coccyx and associated joints

Alive	C40.0-Long bones: upper limb, scapula, and associated joints
Dead	C41.8-Overlap bones, joints, and art. cartilage
Dead	C41.4-Pelvic bones, sacrum, coccyx and associated joints
Alive	C40.2-Long bones of lower limb and associated joints
Alive	C41.4-Pelvic bones, sacrum, coccyx and associated joints
Dead	C41.2-Vertebral column
Dead	C41.4-Pelvic bones, sacrum, coccyx and associated joints
Alive	C40.0-Long bones: upper limb, scapula, and associated joints
Alive	C41.4-Pelvic bones, sacrum, coccyx and associated joints
Dead	C41.2-Vertebral column
Alive	C41.4-Pelvic bones, sacrum, coccyx and associated joints
Alive	C41.4-Pelvic bones, sacrum, coccyx and associated joints
Alive	C41.2-Vertebral column
Dead	C40.2-Long bones of lower limb and associated joints
Dead	C40.0-Long bones: upper limb, scapula, and associated joints
Dead	C40.2-Long bones of lower limb and associated joints
Alive	C40.3-Short bones of lower limb and associated joints
Dead	C41.2-Vertebral column
Dead	C41.2-Vertebral column
Alive	C41.3-Rib, sternum, clavicle and associated joints
Alive	C41.4-Pelvic bones, sacrum, coccyx and associated joints
Alive	C40.2-Long bones of lower limb and associated joints
Alive	C41.4-Pelvic bones, sacrum, coccyx and associated joints
Dead	C41.4-Pelvic bones, sacrum, coccyx and associated joints
Alive	C40.2-Long bones of lower limb and associated joints
Alive	C41.3-Rib, sternum, clavicle and associated joints
Alive	C41.0-Bones of skull and face and associated joints
Dead	C41.9-Bone, NOS
Alive	C40.2-Long bones of lower limb and associated joints
Alive	C40.2-Long bones of lower limb and associated joints
Alive	C40.0-Long bones: upper limb, scapula, and associated joints
Alive	C40.0-Long bones: upper limb, scapula, and associated joints
Alive	C40.2-Long bones of lower limb and associated joints
Alive	C41.4-Pelvic bones, sacrum, coccyx and associated joints
Alive	C41.3-Rib, sternum, clavicle and associated joints
Alive	C40.2-Long bones of lower limb and associated joints
Alive	C40.2-Long bones of lower limb and associated joints
Dead	C41.2-Vertebral column
Dead	C41.4-Pelvic bones, sacrum, coccyx and associated joints
Alive	C40.2-Long bones of lower limb and associated joints
Alive	C40.2-Long bones of lower limb and associated joints
Alive	C40.0-Long bones: upper limb, scapula, and associated joints
Alive	C41.4-Pelvic bones, sacrum, coccyx and associated joints
Alive	C40.0-Long bones: upper limb, scapula, and associated joints
Alive	C41.9-Bone, NOS
Alive	C40.2-Long bones of lower limb and associated joints
Alive	C40.0-Long bones: upper limb, scapula, and associated joints
Alive	C41.0-Bones of skull and face and associated joints
Alive	C41.3-Rib, sternum, clavicle and associated joints
Alive	C41.3-Rib, sternum, clavicle and associated joints
Alive	C40.0-Long bones: upper limb, scapula, and associated joints
Alive	C41.4-Pelvic bones, sacrum, coccyx and associated joints
Alive	C41.0-Bones of skull and face and associated joints
Alive	C41.2-Vertebral column
Dead	C41.4-Pelvic bones, sacrum, coccyx and associated joints
Dead	C40.2-Long bones of lower limb and associated joints
Dead	C40.2-Long bones of lower limb and associated joints
Alive	C41.0-Bones of skull and face and associated joints

Dead	C41.4-Pelvic bones, sacrum, coccyx and associated joints
Dead	C41.4-Pelvic bones, sacrum, coccyx and associated joints
Alive	C41.3-Rib, sternum, clavicle and associated joints
Dead	C40.2-Long bones of lower limb and associated joints
Alive	C40.2-Long bones of lower limb and associated joints
Dead	C40.2-Long bones of lower limb and associated joints
Dead	C40.3-Short bones of lower limb and associated joints
Alive	C41.4-Pelvic bones, sacrum, coccyx and associated joints
Alive	C40.3-Short bones of lower limb and associated joints
Dead	C40.0-Long bones: upper limb, scapula, and associated joints
Dead	C41.4-Pelvic bones, sacrum, coccyx and associated joints
Dead	C41.0-Bones of skull and face and associated joints
Alive	C40.0-Long bones: upper limb, scapula, and associated joints
Alive	C40.2-Long bones of lower limb and associated joints
Dead	C41.8-Overlap bones, joints, and art. cartilage
Alive	C41.2-Vertebral column
Alive	C40.0-Long bones: upper limb, scapula, and associated joints
Dead	C41.3-Rib, sternum, clavicle and associated joints
Alive	C41.1-Mandible
Alive	C40.2-Long bones of lower limb and associated joints
Dead	C41.4-Pelvic bones, sacrum, coccyx and associated joints
Dead	C41.2-Vertebral column
Dead	C40.3-Short bones of lower limb and associated joints
Alive	C40.2-Long bones of lower limb and associated joints
Alive	C40.2-Long bones of lower limb and associated joints
Dead	C40.2-Long bones of lower limb and associated joints
Dead	C40.3-Short bones of lower limb and associated joints
Alive	C41.2-Vertebral column
Alive	C40.2-Long bones of lower limb and associated joints
Alive	C40.1-Short bones of upper limb and associated joints
Alive	C41.3-Rib, sternum, clavicle and associated joints
Dead	C41.4-Pelvic bones, sacrum, coccyx and associated joints
Alive	C40.2-Long bones of lower limb and associated joints
Alive	C41.3-Rib, sternum, clavicle and associated joints
Alive	C41.3-Rib, sternum, clavicle and associated joints
Dead	C41.4-Pelvic bones, sacrum, coccyx and associated joints
Dead	C41.3-Rib, sternum, clavicle and associated joints
Alive	C41.0-Bones of skull and face and associated joints
Alive	C40.0-Long bones: upper limb, scapula, and associated joints
Alive	C41.4-Pelvic bones, sacrum, coccyx and associated joints
Dead	C41.2-Vertebral column
Dead	C40.0-Long bones: upper limb, scapula, and associated joints
Alive	C40.0-Long bones: upper limb, scapula, and associated joints
Alive	C40.3-Short bones of lower limb and associated joints
Alive	C40.0-Long bones: upper limb, scapula, and associated joints
Alive	C41.4-Pelvic bones, sacrum, coccyx and associated joints
Dead	C41.4-Pelvic bones, sacrum, coccyx and associated joints
Dead	C41.4-Pelvic bones, sacrum, coccyx and associated joints
Alive	C40.2-Long bones of lower limb and associated joints
Alive	C41.3-Rib, sternum, clavicle and associated joints
Alive	C41.3-Rib, sternum, clavicle and associated joints
Dead	C41.9-Bone, NOS
Alive	C40.0-Long bones: upper limb, scapula, and associated joints
Alive	C41.2-Vertebral column
Dead	C41.4-Pelvic bones, sacrum, coccyx and associated joints
Alive	C41.2-Vertebral column
Alive	C40.1-Short bones of upper limb and associated joints
Alive	C40.2-Long bones of lower limb and associated joints

Alive	C40.2-Long bones of lower limb and associated joints
Alive	C40.2-Long bones of lower limb and associated joints
Alive	C41.2-Vertebral column
Alive	C41.3-Rib, sternum, clavicle and associated joints
Alive	C40.2-Long bones of lower limb and associated joints
Dead	C41.3-Rib, sternum, clavicle and associated joints
Dead	C41.9-Bone, NOS
Alive	C41.4-Pelvic bones, sacrum, coccyx and associated joints
Alive	C41.4-Pelvic bones, sacrum, coccyx and associated joints
Dead	C41.4-Pelvic bones, sacrum, coccyx and associated joints
Alive	C40.2-Long bones of lower limb and associated joints
Alive	C40.0-Long bones: upper limb, scapula, and associated joints
Alive	C40.2-Long bones of lower limb and associated joints
Alive	C41.4-Pelvic bones, sacrum, coccyx and associated joints
Dead	C40.2-Long bones of lower limb and associated joints
Alive	C41.2-Vertebral column
Alive	C41.4-Pelvic bones, sacrum, coccyx and associated joints
Alive	C41.4-Pelvic bones, sacrum, coccyx and associated joints
Alive	C40.2-Long bones of lower limb and associated joints
Dead	C40.9-Bone of limb, NOS
Dead	C40.2-Long bones of lower limb and associated joints
Alive	C41.2-Vertebral column
Alive	C40.2-Long bones of lower limb and associated joints
Dead	C40.2-Long bones of lower limb and associated joints
Alive	C40.2-Long bones of lower limb and associated joints
Dead	C40.2-Long bones of lower limb and associated joints
Dead	C41.4-Pelvic bones, sacrum, coccyx and associated joints
Alive	C40.0-Long bones: upper limb, scapula, and associated joints
Dead	C41.2-Vertebral column
Alive	C41.0-Bones of skull and face and associated joints
Alive	C40.2-Long bones of lower limb and associated joints
Alive	C40.2-Long bones of lower limb and associated joints
Alive	C41.3-Rib, sternum, clavicle and associated joints
Alive	C40.2-Long bones of lower limb and associated joints
Alive	C41.3-Rib, sternum, clavicle and associated joints
Alive	C40.0-Long bones: upper limb, scapula, and associated joints
Alive	C40.2-Long bones of lower limb and associated joints
Alive	C40.9-Bone of limb, NOS
Alive	C40.2-Long bones of lower limb and associated joints
Alive	C41.1-Mandible
Alive	C41.9-Bone, NOS
Alive	C41.0-Bones of skull and face and associated joints
Alive	C40.2-Long bones of lower limb and associated joints
Alive	C40.2-Long bones of lower limb and associated joints
Alive	C41.2-Vertebral column
Alive	C41.2-Vertebral column
Alive	C40.2-Long bones of lower limb and associated joints
Alive	C41.4-Pelvic bones, sacrum, coccyx and associated joints
Alive	C41.2-Vertebral column
Alive	C41.3-Rib, sternum, clavicle and associated joints
Dead	C41.2-Vertebral column
Alive	C41.4-Pelvic bones, sacrum, coccyx and associated joints
Alive	C41.4-Pelvic bones, sacrum, coccyx and associated joints
Alive	C41.2-Vertebral column
Dead	C41.4-Pelvic bones, sacrum, coccyx and associated joints
Dead	C41.3-Rib, sternum, clavicle and associated joints
Alive	C41.4-Pelvic bones, sacrum, coccyx and associated joints
Alive	C40.2-Long bones of lower limb and associated joints

Dead	C41.4-Pelvic bones, sacrum, coccyx and associated joints
Dead	C41.4-Pelvic bones, sacrum, coccyx and associated joints
Alive	C41.9-Bone, NOS
Alive	C41.0-Bones of skull and face and associated joints
Dead	C41.2-Vertebral column
Dead	C41.4-Pelvic bones, sacrum, coccyx and associated joints
Alive	C41.9-Bone, NOS
Dead	C41.4-Pelvic bones, sacrum, coccyx and associated joints
Alive	C41.2-Vertebral column
Dead	C40.2-Long bones of lower limb and associated joints
Alive	C40.9-Bone of limb, NOS
Alive	C41.2-Vertebral column
Alive	C40.2-Long bones of lower limb and associated joints
Dead	C41.4-Pelvic bones, sacrum, coccyx and associated joints
Dead	C41.2-Vertebral column
Dead	C40.2-Long bones of lower limb and associated joints
Alive	C41.4-Pelvic bones, sacrum, coccyx and associated joints
Alive	C41.4-Pelvic bones, sacrum, coccyx and associated joints
Dead	C41.4-Pelvic bones, sacrum, coccyx and associated joints
Dead	C41.2-Vertebral column
Dead	C41.1-Mandible
Dead	C41.3-Rib, sternum, clavicle and associated joints
Alive	C40.0-Long bones: upper limb, scapula, and associated joints
Alive	C41.4-Pelvic bones, sacrum, coccyx and associated joints
Alive	C40.2-Long bones of lower limb and associated joints
Dead	C41.8-Overlap bones, joints, and art. cartilage
Dead	C41.2-Vertebral column
Dead	C41.4-Pelvic bones, sacrum, coccyx and associated joints
Alive	C41.0-Bones of skull and face and associated joints
Dead	C41.9-Bone, NOS
Alive	C40.2-Long bones of lower limb and associated joints
Dead	C40.9-Bone of limb, NOS
Alive	C40.2-Long bones of lower limb and associated joints
Dead	C40.3-Short bones of lower limb and associated joints
Alive	C41.4-Pelvic bones, sacrum, coccyx and associated joints
Dead	C41.9-Bone, NOS
Alive	C41.9-Bone, NOS
Dead	C41.4-Pelvic bones, sacrum, coccyx and associated joints
Alive	C40.2-Long bones of lower limb and associated joints
Alive	C40.2-Long bones of lower limb and associated joints
Alive	C41.4-Pelvic bones, sacrum, coccyx and associated joints
Alive	C40.2-Long bones of lower limb and associated joints
Dead	C40.2-Long bones of lower limb and associated joints
Alive	C40.2-Long bones of lower limb and associated joints
Alive	C40.1-Short bones of upper limb and associated joints
Dead	C41.0-Bones of skull and face and associated joints
Alive	C41.4-Pelvic bones, sacrum, coccyx and associated joints
Dead	C41.4-Pelvic bones, sacrum, coccyx and associated joints
Dead	C41.3-Rib, sternum, clavicle and associated joints
Dead	C40.3-Short bones of lower limb and associated joints
Alive	C40.0-Long bones: upper limb, scapula, and associated joints
Dead	C41.2-Vertebral column
Alive	C40.2-Long bones of lower limb and associated joints
Alive	C40.2-Long bones of lower limb and associated joints
Alive	C40.2-Long bones of lower limb and associated joints
Dead	C41.3-Rib, sternum, clavicle and associated joints
Dead	C41.9-Bone, NOS
Dead	C41.4-Pelvic bones, sacrum, coccyx and associated joints

Alive	C41.4-Pelvic bones, sacrum, coccyx and associated joints
Dead	C41.4-Pelvic bones, sacrum, coccyx and associated joints
Dead	C40.2-Long bones of lower limb and associated joints
Alive	C41.4-Pelvic bones, sacrum, coccyx and associated joints
Alive	C40.2-Long bones of lower limb and associated joints
Alive	C41.1-Mandible
Alive	C40.2-Long bones of lower limb and associated joints
Alive	C40.2-Long bones of lower limb and associated joints
Dead	C40.3-Short bones of lower limb and associated joints
Dead	C41.4-Pelvic bones, sacrum, coccyx and associated joints
Alive	C41.0-Bones of skull and face and associated joints
Dead	C40.2-Long bones of lower limb and associated joints
Dead	C40.0-Long bones: upper limb, scapula, and associated joints
Alive	C41.4-Pelvic bones, sacrum, coccyx and associated joints
Alive	C41.3-Rib, sternum, clavicle and associated joints
Alive	C41.3-Rib, sternum, clavicle and associated joints
Dead	C40.2-Long bones of lower limb and associated joints
Alive	C40.0-Long bones: upper limb, scapula, and associated joints
Dead	C41.4-Pelvic bones, sacrum, coccyx and associated joints
Alive	C41.1-Mandible
Dead	C41.3-Rib, sternum, clavicle and associated joints
Alive	C41.3-Rib, sternum, clavicle and associated joints
Alive	C41.4-Pelvic bones, sacrum, coccyx and associated joints
Alive	C41.3-Rib, sternum, clavicle and associated joints
Alive	C40.2-Long bones of lower limb and associated joints
Dead	C40.3-Short bones of lower limb and associated joints
Alive	C41.4-Pelvic bones, sacrum, coccyx and associated joints
Dead	C41.4-Pelvic bones, sacrum, coccyx and associated joints
Alive	C40.2-Long bones of lower limb and associated joints
Alive	C40.2-Long bones of lower limb and associated joints
Alive	C41.4-Pelvic bones, sacrum, coccyx and associated joints
Alive	C40.3-Short bones of lower limb and associated joints
Alive	C40.2-Long bones of lower limb and associated joints
Alive	C40.2-Long bones of lower limb and associated joints
Alive	C41.4-Pelvic bones, sacrum, coccyx and associated joints
Dead	C40.2-Long bones of lower limb and associated joints
Alive	C41.4-Pelvic bones, sacrum, coccyx and associated joints
Dead	C40.0-Long bones: upper limb, scapula, and associated joints
Alive	C40.2-Long bones of lower limb and associated joints
Alive	C40.0-Long bones: upper limb, scapula, and associated joints
Alive	C41.2-Vertebral column
Dead	C40.2-Long bones of lower limb and associated joints
Dead	C40.2-Long bones of lower limb and associated joints
Alive	C41.4-Pelvic bones, sacrum, coccyx and associated joints
Dead	C41.4-Pelvic bones, sacrum, coccyx and associated joints
Dead	C41.0-Bones of skull and face and associated joints
Dead	C41.4-Pelvic bones, sacrum, coccyx and associated joints
Dead	C41.4-Pelvic bones, sacrum, coccyx and associated joints
Alive	C40.2-Long bones of lower limb and associated joints
Alive	C40.2-Long bones of lower limb and associated joints
Alive	C41.2-Vertebral column
Alive	C40.0-Long bones: upper limb, scapula, and associated joints
Alive	C40.2-Long bones of lower limb and associated joints
Alive	C41.4-Pelvic bones, sacrum, coccyx and associated joints
Alive	C41.4-Pelvic bones, sacrum, coccyx and associated joints
Alive	C40.2-Long bones of lower limb and associated joints
Alive	C40.2-Long bones of lower limb and associated joints
Dead	C40.1-Short bones of upper limb and associated joints

Alive	C41.2-Vertebral column
Alive	C41.3-Rib, sternum, clavicle and associated joints
Dead	C41.9-Bone, NOS
Dead	C41.4-Pelvic bones, sacrum, coccyx and associated joints
Dead	C41.4-Pelvic bones, sacrum, coccyx and associated joints
Alive	C41.2-Vertebral column
Alive	C40.2-Long bones of lower limb and associated joints
Alive	C40.2-Long bones of lower limb and associated joints
Alive	C41.2-Vertebral column
Alive	C41.3-Rib, sternum, clavicle and associated joints
Alive	C41.4-Pelvic bones, sacrum, coccyx and associated joints
Alive	C41.4-Pelvic bones, sacrum, coccyx and associated joints
Alive	C41.4-Pelvic bones, sacrum, coccyx and associated joints
Alive	C40.0-Long bones: upper limb, scapula, and associated joints
Dead	C41.9-Bone, NOS
Alive	C41.4-Pelvic bones, sacrum, coccyx and associated joints
Dead	C40.0-Long bones: upper limb, scapula, and associated joints
Alive	C41.2-Vertebral column
Alive	C41.4-Pelvic bones, sacrum, coccyx and associated joints
Alive	C41.3-Rib, sternum, clavicle and associated joints
Alive	C40.2-Long bones of lower limb and associated joints
Alive	C40.2-Long bones of lower limb and associated joints
Alive	C40.0-Long bones: upper limb, scapula, and associated joints
Alive	C40.3-Short bones of lower limb and associated joints
Alive	C40.2-Long bones of lower limb and associated joints
Alive	C40.0-Long bones: upper limb, scapula, and associated joints
Dead	C40.1-Short bones of upper limb and associated joints
Dead	C41.4-Pelvic bones, sacrum, coccyx and associated joints
Dead	C40.0-Long bones: upper limb, scapula, and associated joints
Dead	C41.4-Pelvic bones, sacrum, coccyx and associated joints
Alive	C40.2-Long bones of lower limb and associated joints
Alive	C41.3-Rib, sternum, clavicle and associated joints
Dead	C40.2-Long bones of lower limb and associated joints
Alive	C41.4-Pelvic bones, sacrum, coccyx and associated joints
Dead	C41.3-Rib, sternum, clavicle and associated joints
Dead	C41.4-Pelvic bones, sacrum, coccyx and associated joints
Alive	C40.2-Long bones of lower limb and associated joints
Dead	C41.4-Pelvic bones, sacrum, coccyx and associated joints
Dead	C41.3-Rib, sternum, clavicle and associated joints
Alive	C41.2-Vertebral column
Dead	C40.0-Long bones: upper limb, scapula, and associated joints
Dead	C40.2-Long bones of lower limb and associated joints
Alive	C41.4-Pelvic bones, sacrum, coccyx and associated joints
Dead	C41.2-Vertebral column
Dead	C41.4-Pelvic bones, sacrum, coccyx and associated joints
Alive	C41.2-Vertebral column
Dead	C40.2-Long bones of lower limb and associated joints
Dead	C40.2-Long bones of lower limb and associated joints
Dead	C40.2-Long bones of lower limb and associated joints
Dead	C41.4-Pelvic bones, sacrum, coccyx and associated joints
Dead	C41.3-Rib, sternum, clavicle and associated joints
Alive	C41.2-Vertebral column
Dead	C41.4-Pelvic bones, sacrum, coccyx and associated joints
Alive	C40.2-Long bones of lower limb and associated joints
Dead	C40.2-Long bones of lower limb and associated joints
Alive	C40.2-Long bones of lower limb and associated joints
Alive	C41.3-Rib, sternum, clavicle and associated joints
Alive	C40.3-Short bones of lower limb and associated joints

Alive	C40.0-Long bones: upper limb, scapula, and associated joints
Alive	C40.0-Long bones: upper limb, scapula, and associated joints
Alive	C41.4-Pelvic bones, sacrum, coccyx and associated joints
Alive	C41.2-Vertebral column
Dead	C40.2-Long bones of lower limb and associated joints
Dead	C41.4-Pelvic bones, sacrum, coccyx and associated joints
Alive	C40.2-Long bones of lower limb and associated joints
Alive	C41.2-Vertebral column
Alive	C41.4-Pelvic bones, sacrum, coccyx and associated joints
Alive	C40.2-Long bones of lower limb and associated joints
Alive	C41.2-Vertebral column
Alive	C41.2-Vertebral column
Dead	C41.2-Vertebral column
Alive	C41.9-Bone, NOS
Alive	C41.4-Pelvic bones, sacrum, coccyx and associated joints
Alive	C40.2-Long bones of lower limb and associated joints
Dead	C41.4-Pelvic bones, sacrum, coccyx and associated joints
Dead	C41.9-Bone, NOS
Alive	C40.2-Long bones of lower limb and associated joints
Alive	C40.2-Long bones of lower limb and associated joints
Alive	C40.1-Short bones of upper limb and associated joints
Alive	C41.3-Rib, sternum, clavicle and associated joints
Dead	C41.9-Bone, NOS
Alive	C41.2-Vertebral column
Alive	C40.3-Short bones of lower limb and associated joints
Dead	C41.3-Rib, sternum, clavicle and associated joints
Dead	C40.2-Long bones of lower limb and associated joints
Dead	C40.0-Long bones: upper limb, scapula, and associated joints
Dead	C41.4-Pelvic bones, sacrum, coccyx and associated joints
Alive	C41.0-Bones of skull and face and associated joints
Dead	C41.4-Pelvic bones, sacrum, coccyx and associated joints
Dead	C41.4-Pelvic bones, sacrum, coccyx and associated joints
Dead	C40.0-Long bones: upper limb, scapula, and associated joints
Alive	C41.3-Rib, sternum, clavicle and associated joints
Dead	C40.2-Long bones of lower limb and associated joints
Alive	C40.8-Overlap of bones, joints, and art. cartilage of limbs
Alive	C41.3-Rib, sternum, clavicle and associated joints
Dead	C40.0-Long bones: upper limb, scapula, and associated joints
Alive	C40.2-Long bones of lower limb and associated joints
Alive	C41.3-Rib, sternum, clavicle and associated joints
Alive	C41.4-Pelvic bones, sacrum, coccyx and associated joints
Alive	C41.0-Bones of skull and face and associated joints
Alive	C40.2-Long bones of lower limb and associated joints
Alive	C40.2-Long bones of lower limb and associated joints
Alive	C40.2-Long bones of lower limb and associated joints
Alive	C41.4-Pelvic bones, sacrum, coccyx and associated joints
Alive	C40.2-Long bones of lower limb and associated joints
Dead	C41.2-Vertebral column
Alive	C40.0-Long bones: upper limb, scapula, and associated joints
Alive	C40.0-Long bones: upper limb, scapula, and associated joints
Alive	C41.3-Rib, sternum, clavicle and associated joints
Alive	C41.0-Bones of skull and face and associated joints
Alive	C40.2-Long bones of lower limb and associated joints
Alive	C40.2-Long bones of lower limb and associated joints
Alive	C41.3-Rib, sternum, clavicle and associated joints
Alive	C40.2-Long bones of lower limb and associated joints
Alive	C41.0-Bones of skull and face and associated joints
Alive	C41.0-Bones of skull and face and associated joints

Alive	C40.2-Long bones of lower limb and associated joints
Dead	C41.4-Pelvic bones, sacrum, coccyx and associated joints
Alive	C40.0-Long bones: upper limb, scapula, and associated joints
Alive	C40.0-Long bones: upper limb, scapula, and associated joints
Alive	C40.2-Long bones of lower limb and associated joints
Alive	C40.0-Long bones: upper limb, scapula, and associated joints
Alive	C41.2-Vertebral column
Dead	C40.2-Long bones of lower limb and associated joints
Alive	C40.2-Long bones of lower limb and associated joints
Alive	C40.2-Long bones of lower limb and associated joints
Alive	C41.2-Vertebral column
Alive	C41.3-Rib, sternum, clavicle and associated joints
Alive	C40.2-Long bones of lower limb and associated joints
Dead	C41.3-Rib, sternum, clavicle and associated joints
Alive	C41.3-Rib, sternum, clavicle and associated joints
Dead	C41.3-Rib, sternum, clavicle and associated joints
Dead	C41.4-Pelvic bones, sacrum, coccyx and associated joints
Dead	C41.4-Pelvic bones, sacrum, coccyx and associated joints
Alive	C40.2-Long bones of lower limb and associated joints
Dead	C40.2-Long bones of lower limb and associated joints
Dead	C40.2-Long bones of lower limb and associated joints
Alive	C41.4-Pelvic bones, sacrum, coccyx and associated joints
Dead	C41.1-Mandible
Alive	C41.2-Vertebral column
Alive	C40.2-Long bones of lower limb and associated joints
Dead	C41.2-Vertebral column
Alive	C41.4-Pelvic bones, sacrum, coccyx and associated joints
Alive	C40.2-Long bones of lower limb and associated joints
Dead	C40.2-Long bones of lower limb and associated joints
Alive	C41.4-Pelvic bones, sacrum, coccyx and associated joints
Dead	C41.4-Pelvic bones, sacrum, coccyx and associated joints
Alive	C41.4-Pelvic bones, sacrum, coccyx and associated joints
Dead	C40.2-Long bones of lower limb and associated joints
Alive	C40.2-Long bones of lower limb and associated joints
Dead	C41.0-Bones of skull and face and associated joints
Dead	C41.2-Vertebral column
Dead	C41.0-Bones of skull and face and associated joints
Alive	C41.4-Pelvic bones, sacrum, coccyx and associated joints
Dead	C40.2-Long bones of lower limb and associated joints
Dead	C41.3-Rib, sternum, clavicle and associated joints
Alive	C40.2-Long bones of lower limb and associated joints
Dead	C40.2-Long bones of lower limb and associated joints
Alive	C41.4-Pelvic bones, sacrum, coccyx and associated joints
Alive	C40.2-Long bones of lower limb and associated joints
Alive	C40.2-Long bones of lower limb and associated joints
Dead	C41.3-Rib, sternum, clavicle and associated joints
Dead	C41.4-Pelvic bones, sacrum, coccyx and associated joints
Alive	C40.2-Long bones of lower limb and associated joints
Alive	C41.3-Rib, sternum, clavicle and associated joints
Dead	C41.3-Rib, sternum, clavicle and associated joints
Alive	C41.3-Rib, sternum, clavicle and associated joints
Alive	C41.3-Rib, sternum, clavicle and associated joints
Alive	C40.2-Long bones of lower limb and associated joints
Alive	C41.4-Pelvic bones, sacrum, coccyx and associated joints
Dead	C41.4-Pelvic bones, sacrum, coccyx and associated joints
Dead	C41.2-Vertebral column
Alive	C40.2-Long bones of lower limb and associated joints
Alive	C41.4-Pelvic bones, sacrum, coccyx and associated joints

Alive	C41.2-Vertebral column
Dead	C41.9-Bone, NOS
Alive	C40.3-Short bones of lower limb and associated joints
Alive	C41.4-Pelvic bones, sacrum, coccyx and associated joints
Alive	C41.9-Bone, NOS
Alive	C40.0-Long bones: upper limb, scapula, and associated joints
Alive	C40.2-Long bones of lower limb and associated joints
Alive	C40.2-Long bones of lower limb and associated joints
Alive	C40.0-Long bones: upper limb, scapula, and associated joints
Alive	C41.2-Vertebral column
Dead	C40.0-Long bones: upper limb, scapula, and associated joints
Alive	C41.4-Pelvic bones, sacrum, coccyx and associated joints
Alive	C40.2-Long bones of lower limb and associated joints
Alive	C41.3-Rib, sternum, clavicle and associated joints
Alive	C41.4-Pelvic bones, sacrum, coccyx and associated joints
Alive	C41.3-Rib, sternum, clavicle and associated joints
Alive	C40.3-Short bones of lower limb and associated joints
Alive	C41.0-Bones of skull and face and associated joints
Alive	C41.4-Pelvic bones, sacrum, coccyx and associated joints
Dead	C40.2-Long bones of lower limb and associated joints
Dead	C41.2-Vertebral column
Dead	C41.2-Vertebral column
Alive	C40.2-Long bones of lower limb and associated joints
Alive	C40.2-Long bones of lower limb and associated joints
Dead	C41.2-Vertebral column
Alive	C40.2-Long bones of lower limb and associated joints
Dead	C41.3-Rib, sternum, clavicle and associated joints
Dead	C40.1-Short bones of upper limb and associated joints
Alive	C41.4-Pelvic bones, sacrum, coccyx and associated joints
Dead	C41.4-Pelvic bones, sacrum, coccyx and associated joints
Dead	C41.9-Bone, NOS
Dead	C41.4-Pelvic bones, sacrum, coccyx and associated joints
Alive	C41.4-Pelvic bones, sacrum, coccyx and associated joints
Alive	C40.2-Long bones of lower limb and associated joints
Dead	C41.4-Pelvic bones, sacrum, coccyx and associated joints
Dead	C41.9-Bone, NOS
Dead	C41.4-Pelvic bones, sacrum, coccyx and associated joints
Alive	C41.4-Pelvic bones, sacrum, coccyx and associated joints
Dead	C41.4-Pelvic bones, sacrum, coccyx and associated joints
Dead	C40.2-Long bones of lower limb and associated joints
Alive	C40.2-Long bones of lower limb and associated joints
Dead	C41.2-Vertebral column
Dead	C41.4-Pelvic bones, sacrum, coccyx and associated joints
Dead	C40.3-Short bones of lower limb and associated joints
Alive	C40.2-Long bones of lower limb and associated joints
Alive	C40.0-Long bones: upper limb, scapula, and associated joints
Alive	C40.3-Short bones of lower limb and associated joints
Alive	C41.3-Rib, sternum, clavicle and associated joints
Alive	C40.2-Long bones of lower limb and associated joints
Dead	C41.4-Pelvic bones, sacrum, coccyx and associated joints
Alive	C41.3-Rib, sternum, clavicle and associated joints
Alive	C40.0-Long bones: upper limb, scapula, and associated joints
Alive	C41.3-Rib, sternum, clavicle and associated joints
Alive	C41.3-Rib, sternum, clavicle and associated joints
Dead	C41.4-Pelvic bones, sacrum, coccyx and associated joints
Alive	C41.2-Vertebral column
Dead	C41.3-Rib, sternum, clavicle and associated joints
Alive	C40.0-Long bones: upper limb, scapula, and associated joints

Dead	C41.3-Rib, sternum, clavicle and associated joints
Dead	C40.2-Long bones of lower limb and associated joints
Dead	C40.0-Long bones: upper limb, scapula, and associated joints
Dead	C40.0-Long bones: upper limb, scapula, and associated joints
Alive	C41.4-Pelvic bones, sacrum, coccyx and associated joints
Alive	C40.2-Long bones of lower limb and associated joints
Dead	C41.2-Vertebral column
Dead	C41.4-Pelvic bones, sacrum, coccyx and associated joints
Alive	C41.1-Mandible
Alive	C40.2-Long bones of lower limb and associated joints
Dead	C41.4-Pelvic bones, sacrum, coccyx and associated joints
Alive	C40.0-Long bones: upper limb, scapula, and associated joints
Alive	C40.2-Long bones of lower limb and associated joints
Alive	C40.0-Long bones: upper limb, scapula, and associated joints
Alive	C41.0-Bones of skull and face and associated joints
Dead	C40.0-Long bones: upper limb, scapula, and associated joints
Alive	C41.4-Pelvic bones, sacrum, coccyx and associated joints
Alive	C41.4-Pelvic bones, sacrum, coccyx and associated joints
Dead	C41.0-Bones of skull and face and associated joints
Alive	C41.2-Vertebral column
Dead	C40.0-Long bones: upper limb, scapula, and associated joints
Dead	C40.0-Long bones: upper limb, scapula, and associated joints
Dead	C41.4-Pelvic bones, sacrum, coccyx and associated joints
Alive	C41.4-Pelvic bones, sacrum, coccyx and associated joints
Alive	C40.2-Long bones of lower limb and associated joints
Alive	C41.4-Pelvic bones, sacrum, coccyx and associated joints
Alive	C40.2-Long bones of lower limb and associated joints
Alive	C41.9-Bone, NOS
Alive	C41.3-Rib, sternum, clavicle and associated joints
Alive	C40.2-Long bones of lower limb and associated joints
Alive	C41.0-Bones of skull and face and associated joints
Dead	C41.3-Rib, sternum, clavicle and associated joints
Alive	C41.2-Vertebral column
Dead	C40.0-Long bones: upper limb, scapula, and associated joints
Dead	C40.0-Long bones: upper limb, scapula, and associated joints
Alive	C40.0-Long bones: upper limb, scapula, and associated joints
Dead	C41.3-Rib, sternum, clavicle and associated joints
Dead	C40.0-Long bones: upper limb, scapula, and associated joints
Dead	C41.3-Rib, sternum, clavicle and associated joints
Alive	C40.2-Long bones of lower limb and associated joints
Alive	C40.0-Long bones: upper limb, scapula, and associated joints
Dead	C40.0-Long bones: upper limb, scapula, and associated joints
Dead	C41.9-Bone, NOS
Dead	C41.2-Vertebral column
Dead	C40.2-Long bones of lower limb and associated joints
Alive	C40.2-Long bones of lower limb and associated joints
Alive	C41.9-Bone, NOS
Alive	C40.2-Long bones of lower limb and associated joints
Dead	C40.0-Long bones: upper limb, scapula, and associated joints
Alive	C40.2-Long bones of lower limb and associated joints
Dead	C41.2-Vertebral column
Alive	C41.4-Pelvic bones, sacrum, coccyx and associated joints
Dead	C40.2-Long bones of lower limb and associated joints
Alive	C40.2-Long bones of lower limb and associated joints
Alive	C41.2-Vertebral column
Alive	C41.4-Pelvic bones, sacrum, coccyx and associated joints
Dead	C41.4-Pelvic bones, sacrum, coccyx and associated joints
Dead	C41.3-Rib, sternum, clavicle and associated joints

Dead	C41.2-Vertebral column
Dead	C40.2-Long bones of lower limb and associated joints
Dead	C41.9-Bone, NOS
Dead	C41.4-Pelvic bones, sacrum, coccyx and associated joints
Dead	C41.0-Bones of skull and face and associated joints
Alive	C41.4-Pelvic bones, sacrum, coccyx and associated joints
Alive	C40.0-Long bones: upper limb, scapula, and associated joints
Dead	C41.4-Pelvic bones, sacrum, coccyx and associated joints
Dead	C40.0-Long bones: upper limb, scapula, and associated joints
Dead	C41.4-Pelvic bones, sacrum, coccyx and associated joints
Dead	C40.0-Long bones: upper limb, scapula, and associated joints
Dead	C41.0-Bones of skull and face and associated joints
Alive	C40.0-Long bones: upper limb, scapula, and associated joints
Alive	C41.0-Bones of skull and face and associated joints
Alive	C41.4-Pelvic bones, sacrum, coccyx and associated joints
Alive	C40.2-Long bones of lower limb and associated joints
Alive	C40.0-Long bones: upper limb, scapula, and associated joints
Alive	C41.4-Pelvic bones, sacrum, coccyx and associated joints
Alive	C41.0-Bones of skull and face and associated joints
Alive	C41.0-Bones of skull and face and associated joints
Alive	C40.0-Long bones: upper limb, scapula, and associated joints
Dead	C41.2-Vertebral column
Alive	C40.2-Long bones of lower limb and associated joints
Alive	C41.0-Bones of skull and face and associated joints
Dead	C41.4-Pelvic bones, sacrum, coccyx and associated joints
Alive	C40.2-Long bones of lower limb and associated joints
Alive	C40.2-Long bones of lower limb and associated joints
Alive	C41.3-Rib, sternum, clavicle and associated joints
Dead	C40.0-Long bones: upper limb, scapula, and associated joints
Dead	C41.4-Pelvic bones, sacrum, coccyx and associated joints
Dead	C41.4-Pelvic bones, sacrum, coccyx and associated joints
Alive	C40.0-Long bones: upper limb, scapula, and associated joints
Dead	C40.2-Long bones of lower limb and associated joints
Alive	C41.0-Bones of skull and face and associated joints
Dead	C40.2-Long bones of lower limb and associated joints
Alive	C40.2-Long bones of lower limb and associated joints
Alive	C41.3-Rib, sternum, clavicle and associated joints
Alive	C41.4-Pelvic bones, sacrum, coccyx and associated joints
Dead	C41.2-Vertebral column
Alive	C40.0-Long bones: upper limb, scapula, and associated joints
Alive	C40.2-Long bones of lower limb and associated joints
Dead	C41.4-Pelvic bones, sacrum, coccyx and associated joints
Alive	C41.4-Pelvic bones, sacrum, coccyx and associated joints
Alive	C41.2-Vertebral column
Alive	C41.9-Bone, NOS
Alive	C40.2-Long bones of lower limb and associated joints
Dead	C41.2-Vertebral column
Alive	C40.3-Short bones of lower limb and associated joints
Dead	C41.4-Pelvic bones, sacrum, coccyx and associated joints
Dead	C40.2-Long bones of lower limb and associated joints
Alive	C40.0-Long bones: upper limb, scapula, and associated joints
Alive	C40.2-Long bones of lower limb and associated joints
Alive	C41.4-Pelvic bones, sacrum, coccyx and associated joints
Dead	C40.3-Short bones of lower limb and associated joints
Dead	C41.4-Pelvic bones, sacrum, coccyx and associated joints
Alive	C41.2-Vertebral column
Dead	C41.3-Rib, sternum, clavicle and associated joints
Dead	C40.2-Long bones of lower limb and associated joints

Dead	C40.2-Long bones of lower limb and associated joints
Dead	C41.4-Pelvic bones, sacrum, coccyx and associated joints
Dead	C40.2-Long bones of lower limb and associated joints
Alive	C40.2-Long bones of lower limb and associated joints
Alive	C40.2-Long bones of lower limb and associated joints
Alive	C41.0-Bones of skull and face and associated joints
Dead	C41.2-Vertebral column
Alive	C41.0-Bones of skull and face and associated joints
Dead	C41.9-Bone, NOS
Alive	C41.3-Rib, sternum, clavicle and associated joints
Dead	C41.2-Vertebral column
Alive	C40.2-Long bones of lower limb and associated joints
Alive	C41.2-Vertebral column
Alive	C41.4-Pelvic bones, sacrum, coccyx and associated joints
Dead	C41.3-Rib, sternum, clavicle and associated joints
Alive	C40.2-Long bones of lower limb and associated joints
Alive	C40.2-Long bones of lower limb and associated joints
Alive	C40.0-Long bones: upper limb, scapula, and associated joints
Dead	C41.4-Pelvic bones, sacrum, coccyx and associated joints
Dead	C40.0-Long bones: upper limb, scapula, and associated joints
Dead	C41.4-Pelvic bones, sacrum, coccyx and associated joints
Alive	C40.0-Long bones: upper limb, scapula, and associated joints
Alive	C40.1-Short bones of upper limb and associated joints
Alive	C41.0-Bones of skull and face and associated joints
Alive	C41.3-Rib, sternum, clavicle and associated joints
Dead	C41.2-Vertebral column
Alive	C40.2-Long bones of lower limb and associated joints
Alive	C40.1-Short bones of upper limb and associated joints
Dead	C41.1-Mandible
Alive	C40.2-Long bones of lower limb and associated joints
Dead	C41.3-Rib, sternum, clavicle and associated joints
Dead	C40.2-Long bones of lower limb and associated joints
Alive	C40.2-Long bones of lower limb and associated joints
Dead	C41.2-Vertebral column
Alive	C41.0-Bones of skull and face and associated joints
Dead	C41.9-Bone, NOS
Dead	C40.2-Long bones of lower limb and associated joints
Alive	C41.4-Pelvic bones, sacrum, coccyx and associated joints
Dead	C41.4-Pelvic bones, sacrum, coccyx and associated joints
Dead	C41.2-Vertebral column
Alive	C40.2-Long bones of lower limb and associated joints
Dead	C41.9-Bone, NOS
Alive	C41.2-Vertebral column
Alive	C40.0-Long bones: upper limb, scapula, and associated joints
Alive	C41.1-Mandible
Dead	C41.4-Pelvic bones, sacrum, coccyx and associated joints
Alive	C41.3-Rib, sternum, clavicle and associated joints
Alive	C41.0-Bones of skull and face and associated joints
Dead	C41.4-Pelvic bones, sacrum, coccyx and associated joints
Dead	C40.3-Short bones of lower limb and associated joints
Dead	C40.2-Long bones of lower limb and associated joints
Alive	C41.3-Rib, sternum, clavicle and associated joints
Dead	C41.3-Rib, sternum, clavicle and associated joints
Alive	C40.0-Long bones: upper limb, scapula, and associated joints
Alive	C41.4-Pelvic bones, sacrum, coccyx and associated joints
Alive	C40.9-Bone of limb, NOS
Dead	C41.2-Vertebral column
Alive	C40.0-Long bones: upper limb, scapula, and associated joints

Dead	C40.2-Long bones of lower limb and associated joints
Dead	C41.9-Bone, NOS
Alive	C40.2-Long bones of lower limb and associated joints
Alive	C41.4-Pelvic bones, sacrum, coccyx and associated joints
Alive	C41.4-Pelvic bones, sacrum, coccyx and associated joints
Dead	C41.9-Bone, NOS
Dead	C41.2-Vertebral column
Alive	C40.3-Short bones of lower limb and associated joints
Alive	C40.2-Long bones of lower limb and associated joints
Dead	C40.2-Long bones of lower limb and associated joints
Alive	C41.2-Vertebral column
Alive	C41.2-Vertebral column
Alive	C41.3-Rib, sternum, clavicle and associated joints
Alive	C41.3-Rib, sternum, clavicle and associated joints
Alive	C40.0-Long bones: upper limb, scapula, and associated joints
Dead	C41.4-Pelvic bones, sacrum, coccyx and associated joints
Dead	C41.4-Pelvic bones, sacrum, coccyx and associated joints
Alive	C40.0-Long bones: upper limb, scapula, and associated joints
Dead	C41.9-Bone, NOS
Dead	C41.9-Bone, NOS
Dead	C41.9-Bone, NOS
Alive	C40.0-Long bones: upper limb, scapula, and associated joints
Alive	C40.0-Long bones: upper limb, scapula, and associated joints
Alive	C40.2-Long bones of lower limb and associated joints
Alive	C41.4-Pelvic bones, sacrum, coccyx and associated joints
Alive	C40.2-Long bones of lower limb and associated joints
Alive	C41.4-Pelvic bones, sacrum, coccyx and associated joints
Alive	C40.2-Long bones of lower limb and associated joints
Dead	C41.4-Pelvic bones, sacrum, coccyx and associated joints
Alive	C41.4-Pelvic bones, sacrum, coccyx and associated joints
Alive	C41.0-Bones of skull and face and associated joints
Alive	C40.2-Long bones of lower limb and associated joints
Dead	C40.0-Long bones: upper limb, scapula, and associated joints
Alive	C41.4-Pelvic bones, sacrum, coccyx and associated joints
Alive	C41.0-Bones of skull and face and associated joints
Dead	C41.9-Bone, NOS
Alive	C41.4-Pelvic bones, sacrum, coccyx and associated joints
Alive	C40.0-Long bones: upper limb, scapula, and associated joints
Alive	C41.2-Vertebral column
Alive	C40.0-Long bones: upper limb, scapula, and associated joints
Alive	C40.2-Long bones of lower limb and associated joints
Dead	C41.4-Pelvic bones, sacrum, coccyx and associated joints
Dead	C41.2-Vertebral column
Alive	C41.2-Vertebral column
Alive	C40.2-Long bones of lower limb and associated joints
Alive	C41.4-Pelvic bones, sacrum, coccyx and associated joints
Alive	C40.2-Long bones of lower limb and associated joints
Alive	C41.3-Rib, sternum, clavicle and associated joints
Alive	C41.0-Bones of skull and face and associated joints
Alive	C40.2-Long bones of lower limb and associated joints
Alive	C41.4-Pelvic bones, sacrum, coccyx and associated joints
Alive	C41.2-Vertebral column
Alive	C40.2-Long bones of lower limb and associated joints
Alive	C41.0-Bones of skull and face and associated joints
Alive	C40.2-Long bones of lower limb and associated joints
Alive	C40.2-Long bones of lower limb and associated joints
Alive	C41.2-Vertebral column
Alive	C41.3-Rib, sternum, clavicle and associated joints

Alive	C40.0-Long bones: upper limb, scapula, and associated joints
Alive	C41.9-Bone, NOS
Dead	C41.9-Bone, NOS
Dead	C40.2-Long bones of lower limb and associated joints
Dead	C41.1-Mandible
Alive	C40.3-Short bones of lower limb and associated joints
Alive	C40.2-Long bones of lower limb and associated joints
Dead	C41.4-Pelvic bones, sacrum, coccyx and associated joints
Alive	C41.4-Pelvic bones, sacrum, coccyx and associated joints
Dead	C40.2-Long bones of lower limb and associated joints
Alive	C40.0-Long bones: upper limb, scapula, and associated joints
Dead	C40.2-Long bones of lower limb and associated joints
Dead	C40.0-Long bones: upper limb, scapula, and associated joints
Alive	C40.2-Long bones of lower limb and associated joints
Dead	C41.3-Rib, sternum, clavicle and associated joints
Dead	C41.4-Pelvic bones, sacrum, coccyx and associated joints
Alive	C41.4-Pelvic bones, sacrum, coccyx and associated joints
Alive	C41.2-Vertebral column
Alive	C41.2-Vertebral column
Alive	C40.2-Long bones of lower limb and associated joints
Dead	C40.0-Long bones: upper limb, scapula, and associated joints
Dead	C41.9-Bone, NOS
Dead	C40.2-Long bones of lower limb and associated joints
Alive	C40.2-Long bones of lower limb and associated joints
Dead	C41.3-Rib, sternum, clavicle and associated joints
Alive	C41.3-Rib, sternum, clavicle and associated joints
Dead	C40.2-Long bones of lower limb and associated joints
Alive	C41.2-Vertebral column
Alive	C41.2-Vertebral column
Alive	C41.3-Rib, sternum, clavicle and associated joints
Alive	C41.4-Pelvic bones, sacrum, coccyx and associated joints
Alive	C40.2-Long bones of lower limb and associated joints
Dead	C41.2-Vertebral column
Dead	C40.3-Short bones of lower limb and associated joints
Dead	C40.2-Long bones of lower limb and associated joints
Dead	C40.3-Short bones of lower limb and associated joints
Alive	C41.3-Rib, sternum, clavicle and associated joints
Dead	C41.4-Pelvic bones, sacrum, coccyx and associated joints
Alive	C41.4-Pelvic bones, sacrum, coccyx and associated joints
Alive	C40.0-Long bones: upper limb, scapula, and associated joints
Dead	C40.0-Long bones: upper limb, scapula, and associated joints
Dead	C41.4-Pelvic bones, sacrum, coccyx and associated joints
Dead	C41.0-Bones of skull and face and associated joints
Dead	C41.4-Pelvic bones, sacrum, coccyx and associated joints
Dead	C41.2-Vertebral column
Dead	C41.4-Pelvic bones, sacrum, coccyx and associated joints
Alive	C41.9-Bone, NOS
Dead	C40.0-Long bones: upper limb, scapula, and associated joints
Alive	C41.0-Bones of skull and face and associated joints
Dead	C40.2-Long bones of lower limb and associated joints
Dead	C41.4-Pelvic bones, sacrum, coccyx and associated joints
Dead	C40.3-Short bones of lower limb and associated joints
Dead	C41.2-Vertebral column
Dead	C41.4-Pelvic bones, sacrum, coccyx and associated joints
Alive	C40.2-Long bones of lower limb and associated joints
Dead	C41.4-Pelvic bones, sacrum, coccyx and associated joints
Alive	C41.4-Pelvic bones, sacrum, coccyx and associated joints
Dead	C41.9-Bone, NOS

Alive	C40.2-Long bones of lower limb and associated joints
Dead	C41.4-Pelvic bones, sacrum, coccyx and associated joints
Alive	C41.4-Pelvic bones, sacrum, coccyx and associated joints
Alive	C41.3-Rib, sternum, clavicle and associated joints
Dead	C40.0-Long bones: upper limb, scapula, and associated joints
Dead	C41.0-Bones of skull and face and associated joints
Dead	C40.2-Long bones of lower limb and associated joints
Alive	C41.2-Vertebral column
Dead	C41.4-Pelvic bones, sacrum, coccyx and associated joints
Alive	C41.0-Bones of skull and face and associated joints
Dead	C40.0-Long bones: upper limb, scapula, and associated joints
Alive	C41.4-Pelvic bones, sacrum, coccyx and associated joints
Dead	C41.3-Rib, sternum, clavicle and associated joints
Alive	C40.0-Long bones: upper limb, scapula, and associated joints
Alive	C41.3-Rib, sternum, clavicle and associated joints
Alive	C40.0-Long bones: upper limb, scapula, and associated joints
Dead	C41.4-Pelvic bones, sacrum, coccyx and associated joints
Dead	C40.2-Long bones of lower limb and associated joints
Alive	C41.4-Pelvic bones, sacrum, coccyx and associated joints
Alive	C41.3-Rib, sternum, clavicle and associated joints
Alive	C41.4-Pelvic bones, sacrum, coccyx and associated joints
Alive	C40.0-Long bones: upper limb, scapula, and associated joints
Dead	C41.4-Pelvic bones, sacrum, coccyx and associated joints
Alive	C41.4-Pelvic bones, sacrum, coccyx and associated joints
Alive	C41.4-Pelvic bones, sacrum, coccyx and associated joints
Dead	C40.2-Long bones of lower limb and associated joints
Dead	C41.9-Bone, NOS
Alive	C41.3-Rib, sternum, clavicle and associated joints
Dead	C40.0-Long bones: upper limb, scapula, and associated joints
Dead	C40.2-Long bones of lower limb and associated joints
Alive	C40.0-Long bones: upper limb, scapula, and associated joints
Dead	C41.4-Pelvic bones, sacrum, coccyx and associated joints
Dead	C41.4-Pelvic bones, sacrum, coccyx and associated joints
Alive	C41.2-Vertebral column
Alive	C40.2-Long bones of lower limb and associated joints
Dead	C41.9-Bone, NOS
Dead	C40.3-Short bones of lower limb and associated joints
Dead	C41.4-Pelvic bones, sacrum, coccyx and associated joints
Dead	C41.3-Rib, sternum, clavicle and associated joints
Dead	C41.4-Pelvic bones, sacrum, coccyx and associated joints
Alive	C40.0-Long bones: upper limb, scapula, and associated joints
Dead	C40.2-Long bones of lower limb and associated joints
Alive	C40.2-Long bones of lower limb and associated joints
Alive	C41.3-Rib, sternum, clavicle and associated joints
Dead	C41.4-Pelvic bones, sacrum, coccyx and associated joints
Alive	C41.4-Pelvic bones, sacrum, coccyx and associated joints
Alive	C41.4-Pelvic bones, sacrum, coccyx and associated joints
Alive	C40.2-Long bones of lower limb and associated joints
Alive	C40.0-Long bones: upper limb, scapula, and associated joints
Alive	C40.0-Long bones: upper limb, scapula, and associated joints
Alive	C40.0-Long bones: upper limb, scapula, and associated joints
Alive	C40.0-Long bones: upper limb, scapula, and associated joints
Dead	C40.0-Long bones: upper limb, scapula, and associated joints
Dead	C41.0-Bones of skull and face and associated joints
Alive	C40.2-Long bones of lower limb and associated joints
Dead	C41.3-Rib, sternum, clavicle and associated joints
Dead	C41.4-Pelvic bones, sacrum, coccyx and associated joints
Alive	C41.0-Bones of skull and face and associated joints

Alive	C40.0-Long bones: upper limb, scapula, and associated joints
Alive	C41.3-Rib, sternum, clavicle and associated joints
Alive	C40.3-Short bones of lower limb and associated joints
Dead	C40.0-Long bones: upper limb, scapula, and associated joints
Alive	C40.2-Long bones of lower limb and associated joints
Alive	C41.4-Pelvic bones, sacrum, coccyx and associated joints
Dead	C41.4-Pelvic bones, sacrum, coccyx and associated joints
Alive	C40.2-Long bones of lower limb and associated joints
Dead	C40.3-Short bones of lower limb and associated joints
Dead	C40.3-Short bones of lower limb and associated joints
Dead	C40.2-Long bones of lower limb and associated joints
Dead	C41.4-Pelvic bones, sacrum, coccyx and associated joints
Dead	C41.3-Rib, sternum, clavicle and associated joints
Dead	C41.3-Rib, sternum, clavicle and associated joints
Dead	C41.2-Vertebral column
Alive	C41.4-Pelvic bones, sacrum, coccyx and associated joints
Alive	C40.2-Long bones of lower limb and associated joints
Dead	C41.2-Vertebral column
Alive	C40.0-Long bones: upper limb, scapula, and associated joints
Dead	C41.9-Bone, NOS
Alive	C40.3-Short bones of lower limb and associated joints
Dead	C40.2-Long bones of lower limb and associated joints
Dead	C41.3-Rib, sternum, clavicle and associated joints
Alive	C40.0-Long bones: upper limb, scapula, and associated joints
Dead	C41.3-Rib, sternum, clavicle and associated joints
Alive	C40.2-Long bones of lower limb and associated joints
Dead	C41.4-Pelvic bones, sacrum, coccyx and associated joints
Alive	C41.4-Pelvic bones, sacrum, coccyx and associated joints
Alive	C40.2-Long bones of lower limb and associated joints
Dead	C41.9-Bone, NOS
Alive	C40.2-Long bones of lower limb and associated joints
Alive	C40.0-Long bones: upper limb, scapula, and associated joints
Dead	C40.2-Long bones of lower limb and associated joints
Alive	C41.4-Pelvic bones, sacrum, coccyx and associated joints
Alive	C40.2-Long bones of lower limb and associated joints
Dead	C41.4-Pelvic bones, sacrum, coccyx and associated joints
Alive	C40.0-Long bones: upper limb, scapula, and associated joints
Dead	C41.4-Pelvic bones, sacrum, coccyx and associated joints
Alive	C40.2-Long bones of lower limb and associated joints
Dead	C41.4-Pelvic bones, sacrum, coccyx and associated joints
Alive	C40.0-Long bones: upper limb, scapula, and associated joints
Dead	C41.2-Vertebral column
Alive	C41.0-Bones of skull and face and associated joints
Dead	C40.3-Short bones of lower limb and associated joints
Dead	C41.4-Pelvic bones, sacrum, coccyx and associated joints
Alive	C40.0-Long bones: upper limb, scapula, and associated joints
Dead	C41.3-Rib, sternum, clavicle and associated joints
Dead	C40.2-Long bones of lower limb and associated joints
Dead	C40.2-Long bones of lower limb and associated joints
Alive	C41.3-Rib, sternum, clavicle and associated joints
Dead	C41.3-Rib, sternum, clavicle and associated joints
Dead	C40.0-Long bones: upper limb, scapula, and associated joints
Dead	C40.2-Long bones of lower limb and associated joints
Alive	C40.2-Long bones of lower limb and associated joints
Dead	C41.3-Rib, sternum, clavicle and associated joints
Alive	C40.2-Long bones of lower limb and associated joints
Dead	C41.4-Pelvic bones, sacrum, coccyx and associated joints
Dead	C41.4-Pelvic bones, sacrum, coccyx and associated joints

Alive	C41.4-Pelvic bones, sacrum, coccyx and associated joints
Alive	C41.0-Bones of skull and face and associated joints
Alive	C41.0-Bones of skull and face and associated joints
Dead	C40.2-Long bones of lower limb and associated joints
Alive	C40.2-Long bones of lower limb and associated joints
Alive	C41.2-Vertebral column
Dead	C41.0-Bones of skull and face and associated joints
Alive	C40.2-Long bones of lower limb and associated joints
Alive	C40.2-Long bones of lower limb and associated joints
Dead	C40.2-Long bones of lower limb and associated joints
Alive	C41.4-Pelvic bones, sacrum, coccyx and associated joints
Dead	C40.2-Long bones of lower limb and associated joints
Alive	C40.0-Long bones: upper limb, scapula, and associated joints
Alive	C41.3-Rib, sternum, clavicle and associated joints
Dead	C41.1-Mandible
Dead	C41.2-Vertebral column
Alive	C41.4-Pelvic bones, sacrum, coccyx and associated joints
Dead	C41.4-Pelvic bones, sacrum, coccyx and associated joints
Dead	C40.2-Long bones of lower limb and associated joints
Alive	C41.3-Rib, sternum, clavicle and associated joints
Alive	C41.4-Pelvic bones, sacrum, coccyx and associated joints
Dead	C40.2-Long bones of lower limb and associated joints
Dead	C41.2-Vertebral column
Alive	C41.3-Rib, sternum, clavicle and associated joints
Alive	C41.3-Rib, sternum, clavicle and associated joints
Dead	C41.4-Pelvic bones, sacrum, coccyx and associated joints
Alive	C41.4-Pelvic bones, sacrum, coccyx and associated joints
Dead	C41.2-Vertebral column
Alive	C40.0-Long bones: upper limb, scapula, and associated joints
Alive	C40.0-Long bones: upper limb, scapula, and associated joints
Dead	C41.4-Pelvic bones, sacrum, coccyx and associated joints
Alive	C40.2-Long bones of lower limb and associated joints
Alive	C41.4-Pelvic bones, sacrum, coccyx and associated joints
Dead	C41.4-Pelvic bones, sacrum, coccyx and associated joints
Alive	C41.3-Rib, sternum, clavicle and associated joints
Alive	C40.2-Long bones of lower limb and associated joints
Alive	C40.2-Long bones of lower limb and associated joints
Alive	C40.2-Long bones of lower limb and associated joints
Alive	C41.3-Rib, sternum, clavicle and associated joints
Alive	C41.4-Pelvic bones, sacrum, coccyx and associated joints
Dead	C40.2-Long bones of lower limb and associated joints
Dead	C41.3-Rib, sternum, clavicle and associated joints
Dead	C41.4-Pelvic bones, sacrum, coccyx and associated joints
Dead	C41.4-Pelvic bones, sacrum, coccyx and associated joints
Dead	C41.4-Pelvic bones, sacrum, coccyx and associated joints
Alive	C40.2-Long bones of lower limb and associated joints
Alive	C41.9-Bone, NOS
Dead	C40.2-Long bones of lower limb and associated joints
Alive	C41.4-Pelvic bones, sacrum, coccyx and associated joints
Dead	C41.4-Pelvic bones, sacrum, coccyx and associated joints
Alive	C40.0-Long bones: upper limb, scapula, and associated joints
Dead	C41.2-Vertebral column
Dead	C41.4-Pelvic bones, sacrum, coccyx and associated joints
Alive	C40.2-Long bones of lower limb and associated joints
Dead	C41.2-Vertebral column
Dead	C41.4-Pelvic bones, sacrum, coccyx and associated joints
Alive	C41.3-Rib, sternum, clavicle and associated joints
Alive	C40.2-Long bones of lower limb and associated joints

Alive	C41.4-Pelvic bones, sacrum, coccyx and associated joints
Alive	C40.2-Long bones of lower limb and associated joints
Dead	C40.3-Short bones of lower limb and associated joints
Dead	C40.2-Long bones of lower limb and associated joints
Alive	C40.2-Long bones of lower limb and associated joints
Alive	C41.0-Bones of skull and face and associated joints
Dead	C41.9-Bone, NOS
Dead	C40.2-Long bones of lower limb and associated joints
Alive	C40.2-Long bones of lower limb and associated joints
Alive	C41.4-Pelvic bones, sacrum, coccyx and associated joints
Alive	C41.3-Rib, sternum, clavicle and associated joints
Dead	C41.2-Vertebral column
Dead	C41.4-Pelvic bones, sacrum, coccyx and associated joints
Alive	C41.2-Vertebral column
Dead	C40.2-Long bones of lower limb and associated joints
Alive	C41.4-Pelvic bones, sacrum, coccyx and associated joints
Alive	C40.2-Long bones of lower limb and associated joints
Alive	C41.2-Vertebral column
Alive	C40.2-Long bones of lower limb and associated joints
Dead	C40.0-Long bones: upper limb, scapula, and associated joints
Dead	C41.4-Pelvic bones, sacrum, coccyx and associated joints
Dead	C40.2-Long bones of lower limb and associated joints
Dead	C40.0-Long bones: upper limb, scapula, and associated joints
Dead	C41.3-Rib, sternum, clavicle and associated joints
Alive	C41.3-Rib, sternum, clavicle and associated joints
Alive	C40.2-Long bones of lower limb and associated joints
Dead	C41.4-Pelvic bones, sacrum, coccyx and associated joints
Dead	C40.2-Long bones of lower limb and associated joints
Dead	C41.2-Vertebral column
Dead	C41.4-Pelvic bones, sacrum, coccyx and associated joints
Dead	C40.2-Long bones of lower limb and associated joints
Dead	C41.4-Pelvic bones, sacrum, coccyx and associated joints
Alive	C41.4-Pelvic bones, sacrum, coccyx and associated joints
Alive	C40.2-Long bones of lower limb and associated joints
Alive	C40.2-Long bones of lower limb and associated joints
Alive	C41.2-Vertebral column
Alive	C41.3-Rib, sternum, clavicle and associated joints
Dead	C41.2-Vertebral column
Alive	C41.4-Pelvic bones, sacrum, coccyx and associated joints
Alive	C41.4-Pelvic bones, sacrum, coccyx and associated joints
Alive	C40.0-Long bones: upper limb, scapula, and associated joints
Alive	C40.0-Long bones: upper limb, scapula, and associated joints
Dead	C41.4-Pelvic bones, sacrum, coccyx and associated joints
Alive	C40.0-Long bones: upper limb, scapula, and associated joints
Dead	C41.0-Bones of skull and face and associated joints
Dead	C41.4-Pelvic bones, sacrum, coccyx and associated joints
Dead	C40.0-Long bones: upper limb, scapula, and associated joints
Dead	C41.3-Rib, sternum, clavicle and associated joints
Alive	C41.3-Rib, sternum, clavicle and associated joints
Dead	C41.4-Pelvic bones, sacrum, coccyx and associated joints
Alive	C41.4-Pelvic bones, sacrum, coccyx and associated joints
Dead	C41.2-Vertebral column
Alive	C40.0-Long bones: upper limb, scapula, and associated joints
Dead	C41.4-Pelvic bones, sacrum, coccyx and associated joints
Dead	C40.2-Long bones of lower limb and associated joints
Alive	C41.9-Bone, NOS
Alive	C41.4-Pelvic bones, sacrum, coccyx and associated joints
Alive	C41.2-Vertebral column

Alive	C41.0-Bones of skull and face and associated joints
Dead	C41.4-Pelvic bones, sacrum, coccyx and associated joints
Alive	C41.4-Pelvic bones, sacrum, coccyx and associated joints
Alive	C41.1-Mandible
Alive	C40.2-Long bones of lower limb and associated joints
Alive	C41.2-Vertebral column
Alive	C41.4-Pelvic bones, sacrum, coccyx and associated joints
Dead	C41.2-Vertebral column
Alive	C41.0-Bones of skull and face and associated joints
Alive	C40.2-Long bones of lower limb and associated joints
Alive	C41.4-Pelvic bones, sacrum, coccyx and associated joints
Dead	C41.4-Pelvic bones, sacrum, coccyx and associated joints
Dead	C40.2-Long bones of lower limb and associated joints
Alive	C40.2-Long bones of lower limb and associated joints
Alive	C41.2-Vertebral column
Dead	C41.2-Vertebral column
Dead	C41.2-Vertebral column
Alive	C41.3-Rib, sternum, clavicle and associated joints
Alive	C41.2-Vertebral column
Alive	C40.2-Long bones of lower limb and associated joints
Alive	C41.2-Vertebral column
Alive	C40.2-Long bones of lower limb and associated joints
Alive	C41.4-Pelvic bones, sacrum, coccyx and associated joints
Alive	C40.2-Long bones of lower limb and associated joints
Dead	C41.4-Pelvic bones, sacrum, coccyx and associated joints
Dead	C41.0-Bones of skull and face and associated joints
Dead	C41.9-Bone, NOS
Alive	C41.2-Vertebral column
Dead	C41.2-Vertebral column
Dead	C41.4-Pelvic bones, sacrum, coccyx and associated joints
Alive	C40.2-Long bones of lower limb and associated joints
Dead	C40.2-Long bones of lower limb and associated joints
Alive	C41.4-Pelvic bones, sacrum, coccyx and associated joints
Alive	C40.2-Long bones of lower limb and associated joints
Dead	C41.2-Vertebral column
Alive	C40.2-Long bones of lower limb and associated joints
Dead	C40.3-Short bones of lower limb and associated joints
Dead	C41.4-Pelvic bones, sacrum, coccyx and associated joints
Dead	C40.0-Long bones: upper limb, scapula, and associated joints
Dead	C41.4-Pelvic bones, sacrum, coccyx and associated joints
Dead	C40.2-Long bones of lower limb and associated joints
Dead	C40.2-Long bones of lower limb and associated joints
Alive	C40.0-Long bones: upper limb, scapula, and associated joints
Dead	C40.0-Long bones: upper limb, scapula, and associated joints
Dead	C40.2-Long bones of lower limb and associated joints
Alive	C40.0-Long bones: upper limb, scapula, and associated joints
Dead	C41.9-Bone, NOS
Dead	C41.2-Vertebral column
Alive	C40.0-Long bones: upper limb, scapula, and associated joints
Alive	C40.0-Long bones: upper limb, scapula, and associated joints
Dead	C41.3-Rib, sternum, clavicle and associated joints
Alive	C40.3-Short bones of lower limb and associated joints
Alive	C40.0-Long bones: upper limb, scapula, and associated joints
Alive	C41.0-Bones of skull and face and associated joints
Dead	C41.4-Pelvic bones, sacrum, coccyx and associated joints
Alive	C41.4-Pelvic bones, sacrum, coccyx and associated joints
Dead	C41.4-Pelvic bones, sacrum, coccyx and associated joints
Alive	C41.2-Vertebral column

Alive	C41.3-Rib, sternum, clavicle and associated joints
Alive	C40.2-Long bones of lower limb and associated joints
Dead	C40.2-Long bones of lower limb and associated joints
Alive	C41.4-Pelvic bones, sacrum, coccyx and associated joints
Alive	C40.2-Long bones of lower limb and associated joints
Alive	C41.3-Rib, sternum, clavicle and associated joints
Alive	C40.2-Long bones of lower limb and associated joints
Alive	C41.4-Pelvic bones, sacrum, coccyx and associated joints
Dead	C40.2-Long bones of lower limb and associated joints
Alive	C41.4-Pelvic bones, sacrum, coccyx and associated joints
Alive	C40.0-Long bones: upper limb, scapula, and associated joints
Dead	C41.3-Rib, sternum, clavicle and associated joints
Alive	C40.2-Long bones of lower limb and associated joints
Alive	C41.4-Pelvic bones, sacrum, coccyx and associated joints
Alive	C41.2-Vertebral column
Alive	C40.2-Long bones of lower limb and associated joints
Alive	C40.2-Long bones of lower limb and associated joints
Dead	C40.2-Long bones of lower limb and associated joints
Dead	C41.4-Pelvic bones, sacrum, coccyx and associated joints
Alive	C40.2-Long bones of lower limb and associated joints
Dead	C41.4-Pelvic bones, sacrum, coccyx and associated joints
Dead	C41.3-Rib, sternum, clavicle and associated joints
Alive	C40.0-Long bones: upper limb, scapula, and associated joints
Alive	C41.3-Rib, sternum, clavicle and associated joints
Alive	C40.2-Long bones of lower limb and associated joints
Alive	C40.9-Bone of limb, NOS
Alive	C40.2-Long bones of lower limb and associated joints
Alive	C40.2-Long bones of lower limb and associated joints
Alive	C40.2-Long bones of lower limb and associated joints
Alive	C41.2-Vertebral column
Alive	C40.2-Long bones of lower limb and associated joints
Dead	C41.3-Rib, sternum, clavicle and associated joints
Alive	C41.4-Pelvic bones, sacrum, coccyx and associated joints
Dead	C41.3-Rib, sternum, clavicle and associated joints
Alive	C41.4-Pelvic bones, sacrum, coccyx and associated joints
Dead	C41.4-Pelvic bones, sacrum, coccyx and associated joints
Alive	C41.4-Pelvic bones, sacrum, coccyx and associated joints
Alive	C41.3-Rib, sternum, clavicle and associated joints
Alive	C40.2-Long bones of lower limb and associated joints
Alive	C41.4-Pelvic bones, sacrum, coccyx and associated joints
Alive	C40.2-Long bones of lower limb and associated joints
Alive	C41.4-Pelvic bones, sacrum, coccyx and associated joints
Dead	C41.3-Rib, sternum, clavicle and associated joints
Dead	C41.4-Pelvic bones, sacrum, coccyx and associated joints
Alive	C40.2-Long bones of lower limb and associated joints
Dead	C41.2-Vertebral column
Dead	C41.4-Pelvic bones, sacrum, coccyx and associated joints
Alive	C41.4-Pelvic bones, sacrum, coccyx and associated joints
Dead	C41.3-Rib, sternum, clavicle and associated joints
Alive	C40.3-Short bones of lower limb and associated joints
Dead	C40.3-Short bones of lower limb and associated joints
Dead	C41.2-Vertebral column
Alive	C41.4-Pelvic bones, sacrum, coccyx and associated joints
Alive	C40.1-Short bones of upper limb and associated joints
Alive	C41.4-Pelvic bones, sacrum, coccyx and associated joints
Alive	C41.4-Pelvic bones, sacrum, coccyx and associated joints
Dead	C41.3-Rib, sternum, clavicle and associated joints
Alive	C41.3-Rib, sternum, clavicle and associated joints

Alive	C41.0-Bones of skull and face and associated joints
Alive	C41.2-Vertebral column
Dead	C41.2-Vertebral column
Dead	C40.2-Long bones of lower limb and associated joints
Alive	C41.4-Pelvic bones, sacrum, coccyx and associated joints
Alive	C41.0-Bones of skull and face and associated joints
Alive	C40.2-Long bones of lower limb and associated joints
Alive	C40.2-Long bones of lower limb and associated joints
Dead	C40.9-Bone of limb, NOS
Alive	C40.2-Long bones of lower limb and associated joints
Alive	C41.3-Rib, sternum, clavicle and associated joints
Alive	C41.3-Rib, sternum, clavicle and associated joints
Alive	C41.0-Bones of skull and face and associated joints
Dead	C40.2-Long bones of lower limb and associated joints
Alive	C40.3-Short bones of lower limb and associated joints
Alive	C41.4-Pelvic bones, sacrum, coccyx and associated joints
Alive	C40.2-Long bones of lower limb and associated joints
Alive	C40.2-Long bones of lower limb and associated joints
Alive	C41.1-Mandible
Alive	C41.3-Rib, sternum, clavicle and associated joints
Alive	C40.2-Long bones of lower limb and associated joints
Dead	C41.3-Rib, sternum, clavicle and associated joints
Alive	C41.4-Pelvic bones, sacrum, coccyx and associated joints
Alive	C41.2-Vertebral column
Alive	C40.2-Long bones of lower limb and associated joints
Alive	C41.3-Rib, sternum, clavicle and associated joints
Alive	C41.9-Bone, NOS
Alive	C41.2-Vertebral column
Alive	C40.2-Long bones of lower limb and associated joints
Alive	C41.3-Rib, sternum, clavicle and associated joints
Alive	C41.3-Rib, sternum, clavicle and associated joints
Alive	C41.3-Rib, sternum, clavicle and associated joints
Alive	C40.2-Long bones of lower limb and associated joints
Alive	C41.4-Pelvic bones, sacrum, coccyx and associated joints
Alive	C41.9-Bone, NOS
Alive	C40.2-Long bones of lower limb and associated joints
Alive	C41.3-Rib, sternum, clavicle and associated joints
Alive	C40.3-Short bones of lower limb and associated joints
Alive	C40.3-Short bones of lower limb and associated joints
Alive	C41.2-Vertebral column
Alive	C41.9-Bone, NOS
Alive	C41.3-Rib, sternum, clavicle and associated joints
Alive	C41.4-Pelvic bones, sacrum, coccyx and associated joints
Alive	C40.2-Long bones of lower limb and associated joints
Dead	C41.1-Mandible
Alive	C40.3-Short bones of lower limb and associated joints
Alive	C41.3-Rib, sternum, clavicle and associated joints
Alive	C41.9-Bone, NOS
Alive	C40.3-Short bones of lower limb and associated joints
Alive	C41.9-Bone, NOS
Alive	C40.0-Long bones: upper limb, scapula, and associated joints
Alive	C41.4-Pelvic bones, sacrum, coccyx and associated joints
Alive	C41.3-Rib, sternum, clavicle and associated joints
Alive	C41.2-Vertebral column
Alive	C41.2-Vertebral column
Alive	C40.0-Long bones: upper limb, scapula, and associated joints
Alive	C41.4-Pelvic bones, sacrum, coccyx and associated joints
Alive	C40.2-Long bones of lower limb and associated joints

Dead	C41.1-Mandible
Alive	C41.3-Rib, sternum, clavicle and associated joints
Dead	C40.2-Long bones of lower limb and associated joints
Alive	C40.2-Long bones of lower limb and associated joints
Dead	C40.0-Long bones: upper limb, scapula, and associated joints
Dead	C41.3-Rib, sternum, clavicle and associated joints
Alive	C41.2-Vertebral column
Alive	C41.4-Pelvic bones, sacrum, coccyx and associated joints
Dead	C40.0-Long bones: upper limb, scapula, and associated joints
Alive	C41.4-Pelvic bones, sacrum, coccyx and associated joints
Dead	C41.9-Bone, NOS
Dead	C41.4-Pelvic bones, sacrum, coccyx and associated joints
Dead	C41.2-Vertebral column
Dead	C40.0-Long bones: upper limb, scapula, and associated joints
Dead	C41.4-Pelvic bones, sacrum, coccyx and associated joints
Dead	C40.2-Long bones of lower limb and associated joints
Dead	C40.3-Short bones of lower limb and associated joints
Alive	C40.9-Bone of limb, NOS
Dead	C41.4-Pelvic bones, sacrum, coccyx and associated joints
Dead	C40.2-Long bones of lower limb and associated joints
Alive	C40.2-Long bones of lower limb and associated joints
Alive	C40.0-Long bones: upper limb, scapula, and associated joints
Dead	C41.4-Pelvic bones, sacrum, coccyx and associated joints
Alive	C41.4-Pelvic bones, sacrum, coccyx and associated joints
Dead	C40.3-Short bones of lower limb and associated joints
Dead	C41.9-Bone, NOS
Alive	C41.0-Bones of skull and face and associated joints
Alive	C41.0-Bones of skull and face and associated joints
Alive	C41.4-Pelvic bones, sacrum, coccyx and associated joints
Dead	C40.2-Long bones of lower limb and associated joints
Alive	C40.2-Long bones of lower limb and associated joints
Dead	C41.4-Pelvic bones, sacrum, coccyx and associated joints
Dead	C40.3-Short bones of lower limb and associated joints
Dead	C41.4-Pelvic bones, sacrum, coccyx and associated joints
Alive	C40.0-Long bones: upper limb, scapula, and associated joints
Alive	C40.0-Long bones: upper limb, scapula, and associated joints
Dead	C41.2-Vertebral column
Dead	C40.2-Long bones of lower limb and associated joints
Dead	C41.9-Bone, NOS
Dead	C40.0-Long bones: upper limb, scapula, and associated joints
Alive	C40.0-Long bones: upper limb, scapula, and associated joints
Alive	C40.2-Long bones of lower limb and associated joints
Alive	C40.2-Long bones of lower limb and associated joints
Alive	C41.0-Bones of skull and face and associated joints
Dead	C41.4-Pelvic bones, sacrum, coccyx and associated joints
Alive	C41.0-Bones of skull and face and associated joints
Alive	C41.2-Vertebral column
Dead	C41.2-Vertebral column
Alive	C41.3-Rib, sternum, clavicle and associated joints
Alive	C41.3-Rib, sternum, clavicle and associated joints
Alive	C41.3-Rib, sternum, clavicle and associated joints
Dead	C41.4-Pelvic bones, sacrum, coccyx and associated joints
Alive	C40.0-Long bones: upper limb, scapula, and associated joints
Dead	C41.2-Vertebral column
Dead	C41.2-Vertebral column
Alive	C40.2-Long bones of lower limb and associated joints
Dead	C40.2-Long bones of lower limb and associated joints
Alive	C40.2-Long bones of lower limb and associated joints

Alive	C40.0-Long bones: upper limb, scapula, and associated joints
Alive	C40.2-Long bones of lower limb and associated joints
Alive	C41.3-Rib, sternum, clavicle and associated joints
Alive	C41.3-Rib, sternum, clavicle and associated joints
Alive	C40.2-Long bones of lower limb and associated joints
Dead	C40.0-Long bones: upper limb, scapula, and associated joints
Dead	C41.4-Pelvic bones, sacrum, coccyx and associated joints
Alive	C41.1-Mandible
Alive	C40.2-Long bones of lower limb and associated joints
Alive	C40.3-Short bones of lower limb and associated joints
Dead	C40.0-Long bones: upper limb, scapula, and associated joints
Dead	C41.2-Vertebral column
Dead	C40.0-Long bones: upper limb, scapula, and associated joints
Dead	C41.4-Pelvic bones, sacrum, coccyx and associated joints
Dead	C41.9-Bone, NOS
Dead	C41.4-Pelvic bones, sacrum, coccyx and associated joints
Alive	C40.0-Long bones: upper limb, scapula, and associated joints
Dead	C41.4-Pelvic bones, sacrum, coccyx and associated joints
Alive	C41.4-Pelvic bones, sacrum, coccyx and associated joints
Alive	C41.3-Rib, sternum, clavicle and associated joints
Alive	C40.0-Long bones: upper limb, scapula, and associated joints
Dead	C41.4-Pelvic bones, sacrum, coccyx and associated joints
Dead	C40.2-Long bones of lower limb and associated joints
Alive	C41.4-Pelvic bones, sacrum, coccyx and associated joints
Dead	C40.2-Long bones of lower limb and associated joints
Alive	C41.3-Rib, sternum, clavicle and associated joints
Alive	C40.2-Long bones of lower limb and associated joints
Dead	C41.3-Rib, sternum, clavicle and associated joints
Alive	C41.4-Pelvic bones, sacrum, coccyx and associated joints
Alive	C41.4-Pelvic bones, sacrum, coccyx and associated joints
Alive	C40.2-Long bones of lower limb and associated joints
Dead	C41.2-Vertebral column
Alive	C41.2-Vertebral column
Dead	C40.0-Long bones: upper limb, scapula, and associated joints
Dead	C41.9-Bone, NOS
Dead	C40.0-Long bones: upper limb, scapula, and associated joints
Alive	C40.0-Long bones: upper limb, scapula, and associated joints
Alive	C41.2-Vertebral column
Alive	C41.2-Vertebral column
Dead	C41.4-Pelvic bones, sacrum, coccyx and associated joints
Alive	C40.2-Long bones of lower limb and associated joints
Alive	C41.0-Bones of skull and face and associated joints
Alive	C40.2-Long bones of lower limb and associated joints
Alive	C41.2-Vertebral column
Alive	C40.2-Long bones of lower limb and associated joints
Alive	C40.2-Long bones of lower limb and associated joints
Alive	C40.2-Long bones of lower limb and associated joints
Dead	C41.9-Bone, NOS
Dead	C40.2-Long bones of lower limb and associated joints
Dead	C41.4-Pelvic bones, sacrum, coccyx and associated joints
Dead	C40.2-Long bones of lower limb and associated joints
Alive	C41.4-Pelvic bones, sacrum, coccyx and associated joints
Dead	C41.4-Pelvic bones, sacrum, coccyx and associated joints
Dead	C41.4-Pelvic bones, sacrum, coccyx and associated joints
Dead	C40.2-Long bones of lower limb and associated joints
Dead	C40.2-Long bones of lower limb and associated joints
Dead	C41.1-Mandible
Dead	C41.4-Pelvic bones, sacrum, coccyx and associated joints

Alive	C40.0-Long bones: upper limb, scapula, and associated joints
Alive	C40.0-Long bones: upper limb, scapula, and associated joints
Dead	C40.2-Long bones of lower limb and associated joints
Dead	C40.2-Long bones of lower limb and associated joints
Dead	C41.4-Pelvic bones, sacrum, coccyx and associated joints
Alive	C41.4-Pelvic bones, sacrum, coccyx and associated joints
Alive	C41.2-Vertebral column
Alive	C41.2-Vertebral column
Dead	C41.4-Pelvic bones, sacrum, coccyx and associated joints
Dead	C41.3-Rib, sternum, clavicle and associated joints
Dead	C41.4-Pelvic bones, sacrum, coccyx and associated joints
Alive	C40.9-Bone of limb, NOS
Dead	C41.4-Pelvic bones, sacrum, coccyx and associated joints
Dead	C41.4-Pelvic bones, sacrum, coccyx and associated joints
Alive	C41.4-Pelvic bones, sacrum, coccyx and associated joints
Alive	C40.2-Long bones of lower limb and associated joints
Alive	C40.2-Long bones of lower limb and associated joints
Dead	C40.0-Long bones: upper limb, scapula, and associated joints
Alive	C40.0-Long bones: upper limb, scapula, and associated joints
Alive	C40.2-Long bones of lower limb and associated joints
Dead	C40.2-Long bones of lower limb and associated joints
Alive	C40.0-Long bones: upper limb, scapula, and associated joints
Alive	C41.3-Rib, sternum, clavicle and associated joints
Alive	C41.0-Bones of skull and face and associated joints
Alive	C41.4-Pelvic bones, sacrum, coccyx and associated joints
Alive	C41.4-Pelvic bones, sacrum, coccyx and associated joints
Dead	C41.3-Rib, sternum, clavicle and associated joints
Dead	C40.2-Long bones of lower limb and associated joints
Dead	C41.0-Bones of skull and face and associated joints
Alive	C41.3-Rib, sternum, clavicle and associated joints
Alive	C41.3-Rib, sternum, clavicle and associated joints
Dead	C40.2-Long bones of lower limb and associated joints
Alive	C41.8-Overlap bones, joints, and art. cartilage
Dead	C41.2-Vertebral column
Dead	C40.1-Short bones of upper limb and associated joints
Alive	C41.4-Pelvic bones, sacrum, coccyx and associated joints
Alive	C40.2-Long bones of lower limb and associated joints
Dead	C40.0-Long bones: upper limb, scapula, and associated joints
Alive	C40.3-Short bones of lower limb and associated joints
Dead	C41.4-Pelvic bones, sacrum, coccyx and associated joints
Dead	C41.3-Rib, sternum, clavicle and associated joints
Alive	C40.0-Long bones: upper limb, scapula, and associated joints
Dead	C41.4-Pelvic bones, sacrum, coccyx and associated joints
Alive	C41.4-Pelvic bones, sacrum, coccyx and associated joints
Dead	C41.4-Pelvic bones, sacrum, coccyx and associated joints
Alive	C40.2-Long bones of lower limb and associated joints
Alive	C41.3-Rib, sternum, clavicle and associated joints
Alive	C40.9-Bone of limb, NOS
Alive	C41.0-Bones of skull and face and associated joints
Alive	C40.0-Long bones: upper limb, scapula, and associated joints
Alive	C41.2-Vertebral column
Dead	C41.4-Pelvic bones, sacrum, coccyx and associated joints
Dead	C41.9-Bone, NOS
Dead	C41.2-Vertebral column
Alive	C41.2-Vertebral column
Dead	C41.4-Pelvic bones, sacrum, coccyx and associated joints
Dead	C41.4-Pelvic bones, sacrum, coccyx and associated joints
Alive	C40.2-Long bones of lower limb and associated joints

Alive	C41.4-Pelvic bones, sacrum, coccyx and associated joints
Alive	C41.3-Rib, sternum, clavicle and associated joints
Alive	C41.4-Pelvic bones, sacrum, coccyx and associated joints
Dead	C41.4-Pelvic bones, sacrum, coccyx and associated joints
Alive	C40.2-Long bones of lower limb and associated joints
Dead	C41.2-Vertebral column
Dead	C40.0-Long bones: upper limb, scapula, and associated joints
Alive	C40.2-Long bones of lower limb and associated joints
Dead	C40.2-Long bones of lower limb and associated joints
Alive	C41.4-Pelvic bones, sacrum, coccyx and associated joints
Dead	C41.9-Bone, NOS
Alive	C41.4-Pelvic bones, sacrum, coccyx and associated joints
Alive	C41.3-Rib, sternum, clavicle and associated joints
Alive	C40.0-Long bones: upper limb, scapula, and associated joints
Alive	C41.2-Vertebral column
Alive	C40.2-Long bones of lower limb and associated joints
Alive	C41.3-Rib, sternum, clavicle and associated joints
Alive	C41.3-Rib, sternum, clavicle and associated joints
Dead	C41.4-Pelvic bones, sacrum, coccyx and associated joints
Alive	C41.0-Bones of skull and face and associated joints
Alive	C40.2-Long bones of lower limb and associated joints
Dead	C41.3-Rib, sternum, clavicle and associated joints
Alive	C40.2-Long bones of lower limb and associated joints
Alive	C41.9-Bone, NOS
Dead	C41.4-Pelvic bones, sacrum, coccyx and associated joints
Dead	C40.9-Bone of limb, NOS
Dead	C41.3-Rib, sternum, clavicle and associated joints
Dead	C41.2-Vertebral column
Alive	C40.2-Long bones of lower limb and associated joints
Dead	C41.9-Bone, NOS
Alive	C40.2-Long bones of lower limb and associated joints
Dead	C41.9-Bone, NOS
Dead	C40.2-Long bones of lower limb and associated joints
Dead	C40.2-Long bones of lower limb and associated joints
Alive	C41.4-Pelvic bones, sacrum, coccyx and associated joints
Dead	C40.2-Long bones of lower limb and associated joints
Dead	C41.2-Vertebral column
Alive	C41.3-Rib, sternum, clavicle and associated joints
Alive	C40.0-Long bones: upper limb, scapula, and associated joints
Dead	C40.0-Long bones: upper limb, scapula, and associated joints
Dead	C41.4-Pelvic bones, sacrum, coccyx and associated joints
Alive	C41.2-Vertebral column
Dead	C40.0-Long bones: upper limb, scapula, and associated joints
Alive	C40.2-Long bones of lower limb and associated joints
Alive	C40.0-Long bones: upper limb, scapula, and associated joints
Dead	C41.3-Rib, sternum, clavicle and associated joints
Alive	C41.2-Vertebral column
Dead	C41.4-Pelvic bones, sacrum, coccyx and associated joints
Dead	C41.3-Rib, sternum, clavicle and associated joints
Alive	C40.0-Long bones: upper limb, scapula, and associated joints
Dead	C40.2-Long bones of lower limb and associated joints
Alive	C40.3-Short bones of lower limb and associated joints
Alive	C41.9-Bone, NOS
Dead	C40.2-Long bones of lower limb and associated joints
Alive	C40.2-Long bones of lower limb and associated joints
Dead	C41.4-Pelvic bones, sacrum, coccyx and associated joints
Dead	C41.4-Pelvic bones, sacrum, coccyx and associated joints
Alive	C40.2-Long bones of lower limb and associated joints

Dead	C41.4-Pelvic bones, sacrum, coccyx and associated joints
Alive	C41.3-Rib, sternum, clavicle and associated joints
Alive	C41.4-Pelvic bones, sacrum, coccyx and associated joints
Alive	C41.3-Rib, sternum, clavicle and associated joints
Dead	C41.2-Vertebral column
Dead	C41.4-Pelvic bones, sacrum, coccyx and associated joints
Alive	C40.2-Long bones of lower limb and associated joints
Dead	C40.2-Long bones of lower limb and associated joints
Dead	C41.2-Vertebral column
Dead	C41.4-Pelvic bones, sacrum, coccyx and associated joints
Dead	C41.9-Bone, NOS
Alive	C40.2-Long bones of lower limb and associated joints
Dead	C40.2-Long bones of lower limb and associated joints
Alive	C41.3-Rib, sternum, clavicle and associated joints
Dead	C41.2-Vertebral column
Alive	C41.4-Pelvic bones, sacrum, coccyx and associated joints
Alive	C40.2-Long bones of lower limb and associated joints
Dead	C40.2-Long bones of lower limb and associated joints
Alive	C40.2-Long bones of lower limb and associated joints
Alive	C41.3-Rib, sternum, clavicle and associated joints
Dead	C41.4-Pelvic bones, sacrum, coccyx and associated joints
Dead	C40.2-Long bones of lower limb and associated joints
Dead	C40.2-Long bones of lower limb and associated joints
Dead	C41.3-Rib, sternum, clavicle and associated joints
Alive	C40.2-Long bones of lower limb and associated joints
Dead	C41.4-Pelvic bones, sacrum, coccyx and associated joints
Dead	C41.3-Rib, sternum, clavicle and associated joints
Dead	C40.0-Long bones: upper limb, scapula, and associated joints
Alive	C41.4-Pelvic bones, sacrum, coccyx and associated joints
Dead	C41.4-Pelvic bones, sacrum, coccyx and associated joints
Alive	C41.3-Rib, sternum, clavicle and associated joints
Dead	C41.2-Vertebral column
Alive	C41.9-Bone, NOS
Dead	C41.1-Mandible
Dead	C41.0-Bones of skull and face and associated joints
Alive	C41.3-Rib, sternum, clavicle and associated joints
Dead	C41.4-Pelvic bones, sacrum, coccyx and associated joints
Dead	C41.0-Bones of skull and face and associated joints
Alive	C41.4-Pelvic bones, sacrum, coccyx and associated joints
Dead	C40.2-Long bones of lower limb and associated joints
Alive	C41.9-Bone, NOS
Alive	C40.9-Bone of limb, NOS
Alive	C41.4-Pelvic bones, sacrum, coccyx and associated joints
Dead	C41.4-Pelvic bones, sacrum, coccyx and associated joints
Alive	C40.3-Short bones of lower limb and associated joints
Alive	C40.2-Long bones of lower limb and associated joints
Dead	C41.3-Rib, sternum, clavicle and associated joints
Alive	C41.4-Pelvic bones, sacrum, coccyx and associated joints
Alive	C40.0-Long bones: upper limb, scapula, and associated joints
Dead	C40.0-Long bones: upper limb, scapula, and associated joints
Alive	C40.2-Long bones of lower limb and associated joints
Dead	C40.2-Long bones of lower limb and associated joints
Dead	C41.2-Vertebral column
Dead	C41.3-Rib, sternum, clavicle and associated joints
Alive	C40.0-Long bones: upper limb, scapula, and associated joints
Alive	C41.2-Vertebral column
Dead	C41.4-Pelvic bones, sacrum, coccyx and associated joints
Alive	C40.3-Short bones of lower limb and associated joints

Alive	C41.3-Rib, sternum, clavicle and associated joints
Alive	C40.0-Long bones: upper limb, scapula, and associated joints
Alive	C40.0-Long bones: upper limb, scapula, and associated joints
Alive	C40.1-Short bones of upper limb and associated joints
Alive	C41.4-Pelvic bones, sacrum, coccyx and associated joints
Alive	C40.0-Long bones: upper limb, scapula, and associated joints
Dead	C41.2-Vertebral column
Dead	C40.2-Long bones of lower limb and associated joints
Alive	C41.2-Vertebral column
Alive	C40.2-Long bones of lower limb and associated joints
Alive	C41.4-Pelvic bones, sacrum, coccyx and associated joints
Dead	C41.4-Pelvic bones, sacrum, coccyx and associated joints
Dead	C40.2-Long bones of lower limb and associated joints
Alive	C41.9-Bone, NOS
Alive	C40.1-Short bones of upper limb and associated joints
Alive	C40.0-Long bones: upper limb, scapula, and associated joints
Alive	C40.2-Long bones of lower limb and associated joints
Alive	C41.3-Rib, sternum, clavicle and associated joints
Alive	C40.2-Long bones of lower limb and associated joints
Alive	C40.2-Long bones of lower limb and associated joints
Alive	C40.2-Long bones of lower limb and associated joints
Alive	C41.4-Pelvic bones, sacrum, coccyx and associated joints
Alive	C41.2-Vertebral column
Alive	C40.2-Long bones of lower limb and associated joints
Dead	C41.4-Pelvic bones, sacrum, coccyx and associated joints
Dead	C41.4-Pelvic bones, sacrum, coccyx and associated joints
Dead	C41.2-Vertebral column
Alive	C40.2-Long bones of lower limb and associated joints
Alive	C40.2-Long bones of lower limb and associated joints
Dead	C40.2-Long bones of lower limb and associated joints
Dead	C41.2-Vertebral column
Alive	C41.4-Pelvic bones, sacrum, coccyx and associated joints
Alive	C40.2-Long bones of lower limb and associated joints
Alive	C40.2-Long bones of lower limb and associated joints
Dead	C40.0-Long bones: upper limb, scapula, and associated joints
Dead	C41.3-Rib, sternum, clavicle and associated joints
Dead	C40.2-Long bones of lower limb and associated joints
Alive	C41.4-Pelvic bones, sacrum, coccyx and associated joints
Alive	C40.0-Long bones: upper limb, scapula, and associated joints
Dead	C41.3-Rib, sternum, clavicle and associated joints
Alive	C40.0-Long bones: upper limb, scapula, and associated joints
Dead	C41.4-Pelvic bones, sacrum, coccyx and associated joints
Dead	C40.2-Long bones of lower limb and associated joints
Alive	C40.2-Long bones of lower limb and associated joints
Dead	C41.3-Rib, sternum, clavicle and associated joints
Dead	C41.0-Bones of skull and face and associated joints
Alive	C40.0-Long bones: upper limb, scapula, and associated joints
Dead	C40.2-Long bones of lower limb and associated joints
Dead	C41.4-Pelvic bones, sacrum, coccyx and associated joints
Alive	C40.0-Long bones: upper limb, scapula, and associated joints
Dead	C40.2-Long bones of lower limb and associated joints
Alive	C41.4-Pelvic bones, sacrum, coccyx and associated joints
Alive	C41.3-Rib, sternum, clavicle and associated joints
Dead	C41.4-Pelvic bones, sacrum, coccyx and associated joints
Alive	C41.2-Vertebral column
Dead	C41.4-Pelvic bones, sacrum, coccyx and associated joints
Dead	C40.2-Long bones of lower limb and associated joints
Alive	C40.2-Long bones of lower limb and associated joints

Dead	C41.2-Vertebral column
Alive	C41.4-Pelvic bones, sacrum, coccyx and associated joints
Alive	C41.3-Rib, sternum, clavicle and associated joints
Alive	C40.2-Long bones of lower limb and associated joints
Dead	C41.3-Rib, sternum, clavicle and associated joints
Alive	C41.2-Vertebral column
Dead	C41.3-Rib, sternum, clavicle and associated joints
Dead	C41.3-Rib, sternum, clavicle and associated joints
Alive	C41.4-Pelvic bones, sacrum, coccyx and associated joints
Alive	C41.2-Vertebral column
Alive	C40.3-Short bones of lower limb and associated joints
Dead	C41.2-Vertebral column
Alive	C41.9-Bone, NOS
Dead	C40.0-Long bones: upper limb, scapula, and associated joints
Alive	C40.2-Long bones of lower limb and associated joints
Dead	C40.2-Long bones of lower limb and associated joints
Dead	C40.0-Long bones: upper limb, scapula, and associated joints
Alive	C40.0-Long bones: upper limb, scapula, and associated joints
Alive	C40.2-Long bones of lower limb and associated joints
Alive	C40.0-Long bones: upper limb, scapula, and associated joints
Dead	C41.0-Bones of skull and face and associated joints
Alive	C41.2-Vertebral column
Alive	C41.2-Vertebral column
Alive	C40.2-Long bones of lower limb and associated joints
Alive	C40.3-Short bones of lower limb and associated joints
Dead	C41.0-Bones of skull and face and associated joints
Dead	C41.8-Overlap bones, joints, and art. cartilage
Alive	C41.4-Pelvic bones, sacrum, coccyx and associated joints
Alive	C40.2-Long bones of lower limb and associated joints
Dead	C41.4-Pelvic bones, sacrum, coccyx and associated joints
Alive	C41.1-Mandible
Alive	C41.3-Rib, sternum, clavicle and associated joints
Dead	C40.2-Long bones of lower limb and associated joints
Alive	C41.4-Pelvic bones, sacrum, coccyx and associated joints
Alive	C41.4-Pelvic bones, sacrum, coccyx and associated joints
Alive	C41.2-Vertebral column
Alive	C41.0-Bones of skull and face and associated joints
Alive	C41.1-Mandible
Alive	C41.4-Pelvic bones, sacrum, coccyx and associated joints
Alive	C41.4-Pelvic bones, sacrum, coccyx and associated joints
Dead	C40.8-Overlap of bones, joints, and art. cartilage of limbs
Alive	C40.0-Long bones: upper limb, scapula, and associated joints
Alive	C40.2-Long bones of lower limb and associated joints
Alive	C41.4-Pelvic bones, sacrum, coccyx and associated joints
Alive	C41.0-Bones of skull and face and associated joints
Alive	C40.3-Short bones of lower limb and associated joints
Dead	C41.4-Pelvic bones, sacrum, coccyx and associated joints
Alive	C41.4-Pelvic bones, sacrum, coccyx and associated joints
Alive	C41.4-Pelvic bones, sacrum, coccyx and associated joints
Dead	C41.9-Bone, NOS
Dead	C41.9-Bone, NOS
Alive	C40.2-Long bones of lower limb and associated joints
Alive	C41.3-Rib, sternum, clavicle and associated joints
Dead	C41.0-Bones of skull and face and associated joints
Alive	C41.3-Rib, sternum, clavicle and associated joints
Alive	C41.3-Rib, sternum, clavicle and associated joints
Alive	C41.0-Bones of skull and face and associated joints
Alive	C40.2-Long bones of lower limb and associated joints

Alive	C40.0-Long bones: upper limb, scapula, and associated joints
Alive	C40.0-Long bones: upper limb, scapula, and associated joints
Alive	C40.3-Short bones of lower limb and associated joints
Alive	C41.4-Pelvic bones, sacrum, coccyx and associated joints
Dead	C40.0-Long bones: upper limb, scapula, and associated joints
Dead	C41.2-Vertebral column
Dead	C41.4-Pelvic bones, sacrum, coccyx and associated joints
Dead	C40.2-Long bones of lower limb and associated joints
Dead	C41.4-Pelvic bones, sacrum, coccyx and associated joints
Alive	C40.2-Long bones of lower limb and associated joints
Alive	C41.4-Pelvic bones, sacrum, coccyx and associated joints
Alive	C41.4-Pelvic bones, sacrum, coccyx and associated joints
Alive	C40.3-Short bones of lower limb and associated joints
Alive	C41.2-Vertebral column
Alive	C41.9-Bone, NOS
Dead	C41.4-Pelvic bones, sacrum, coccyx and associated joints
Alive	C41.2-Vertebral column
Alive	C40.0-Long bones: upper limb, scapula, and associated joints
Alive	C41.3-Rib, sternum, clavicle and associated joints
Alive	C41.4-Pelvic bones, sacrum, coccyx and associated joints
Alive	C41.4-Pelvic bones, sacrum, coccyx and associated joints
Alive	C40.0-Long bones: upper limb, scapula, and associated joints
Dead	C40.3-Short bones of lower limb and associated joints
Alive	C40.3-Short bones of lower limb and associated joints
Alive	C40.0-Long bones: upper limb, scapula, and associated joints
Alive	C41.3-Rib, sternum, clavicle and associated joints
Alive	C41.9-Bone, NOS
Alive	C41.4-Pelvic bones, sacrum, coccyx and associated joints
Alive	C41.0-Bones of skull and face and associated joints
Alive	C40.0-Long bones: upper limb, scapula, and associated joints
Alive	C40.2-Long bones of lower limb and associated joints
Alive	C40.2-Long bones of lower limb and associated joints
Alive	C40.0-Long bones: upper limb, scapula, and associated joints
Alive	C41.3-Rib, sternum, clavicle and associated joints
Alive	C41.3-Rib, sternum, clavicle and associated joints
Alive	C41.4-Pelvic bones, sacrum, coccyx and associated joints
Alive	C40.2-Long bones of lower limb and associated joints
Alive	C40.2-Long bones of lower limb and associated joints
Dead	C40.2-Long bones of lower limb and associated joints
Alive	C41.3-Rib, sternum, clavicle and associated joints
Alive	C41.4-Pelvic bones, sacrum, coccyx and associated joints
Alive	C40.0-Long bones: upper limb, scapula, and associated joints
Alive	C40.2-Long bones of lower limb and associated joints
Alive	C41.3-Rib, sternum, clavicle and associated joints
Alive	C41.4-Pelvic bones, sacrum, coccyx and associated joints
Alive	C40.2-Long bones of lower limb and associated joints
Alive	C41.4-Pelvic bones, sacrum, coccyx and associated joints
Alive	C40.0-Long bones: upper limb, scapula, and associated joints
Dead	C41.4-Pelvic bones, sacrum, coccyx and associated joints
Alive	C40.0-Long bones: upper limb, scapula, and associated joints
Alive	C40.2-Long bones of lower limb and associated joints
Alive	C40.0-Long bones: upper limb, scapula, and associated joints
Alive	C41.3-Rib, sternum, clavicle and associated joints
Dead	C41.4-Pelvic bones, sacrum, coccyx and associated joints
Alive	C41.2-Vertebral column
Alive	C40.2-Long bones of lower limb and associated joints
Dead	C40.2-Long bones of lower limb and associated joints
Alive	C41.4-Pelvic bones, sacrum, coccyx and associated joints

Dead	C40.2-Long bones of lower limb and associated joints
Dead	C40.0-Long bones: upper limb, scapula, and associated joints
Dead	C40.2-Long bones of lower limb and associated joints
Dead	C41.2-Vertebral column
Alive	C40.9-Bone of limb, NOS
Alive	C40.2-Long bones of lower limb and associated joints
Alive	C41.2-Vertebral column
Dead	C40.0-Long bones: upper limb, scapula, and associated joints
Dead	C41.2-Vertebral column
Alive	C40.3-Short bones of lower limb and associated joints
Dead	C41.4-Pelvic bones, sacrum, coccyx and associated joints
Dead	C41.2-Vertebral column
Alive	C40.0-Long bones: upper limb, scapula, and associated joints
Dead	C41.2-Vertebral column
Alive	C41.0-Bones of skull and face and associated joints
Dead	C41.9-Bone, NOS
Alive	C40.2-Long bones of lower limb and associated joints
Alive	C40.0-Long bones: upper limb, scapula, and associated joints
Dead	C41.9-Bone, NOS
Alive	C41.2-Vertebral column
Alive	C40.2-Long bones of lower limb and associated joints
Alive	C41.4-Pelvic bones, sacrum, coccyx and associated joints
Dead	C40.2-Long bones of lower limb and associated joints
Dead	C41.4-Pelvic bones, sacrum, coccyx and associated joints
Alive	C41.2-Vertebral column
Alive	C40.3-Short bones of lower limb and associated joints
Dead	C41.9-Bone, NOS
Alive	C41.4-Pelvic bones, sacrum, coccyx and associated joints
Dead	C41.3-Rib, sternum, clavicle and associated joints
Alive	C40.2-Long bones of lower limb and associated joints
Dead	C41.4-Pelvic bones, sacrum, coccyx and associated joints
Dead	C41.3-Rib, sternum, clavicle and associated joints
Dead	C40.2-Long bones of lower limb and associated joints
Dead	C41.2-Vertebral column
Dead	C41.4-Pelvic bones, sacrum, coccyx and associated joints
Dead	C41.4-Pelvic bones, sacrum, coccyx and associated joints
Alive	C40.3-Short bones of lower limb and associated joints
Dead	C40.2-Long bones of lower limb and associated joints
Alive	C40.3-Short bones of lower limb and associated joints
Alive	C40.2-Long bones of lower limb and associated joints
Dead	C40.0-Long bones: upper limb, scapula, and associated joints
Alive	C41.0-Bones of skull and face and associated joints
Alive	C41.4-Pelvic bones, sacrum, coccyx and associated joints
Dead	C40.0-Long bones: upper limb, scapula, and associated joints
Dead	C41.4-Pelvic bones, sacrum, coccyx and associated joints
Dead	C41.0-Bones of skull and face and associated joints
Dead	C41.0-Bones of skull and face and associated joints
Dead	C41.3-Rib, sternum, clavicle and associated joints
Alive	C40.2-Long bones of lower limb and associated joints
Dead	C41.2-Vertebral column
Dead	C41.3-Rib, sternum, clavicle and associated joints
Alive	C41.0-Bones of skull and face and associated joints
Alive	C41.0-Bones of skull and face and associated joints
Alive	C40.0-Long bones: upper limb, scapula, and associated joints
Dead	C40.2-Long bones of lower limb and associated joints
Alive	C40.2-Long bones of lower limb and associated joints
Dead	C40.2-Long bones of lower limb and associated joints
Dead	C40.3-Short bones of lower limb and associated joints

Dead	C41.4-Pelvic bones, sacrum, coccyx and associated joints
Alive	C41.3-Rib, sternum, clavicle and associated joints
Alive	C41.4-Pelvic bones, sacrum, coccyx and associated joints
Dead	C40.2-Long bones of lower limb and associated joints
Dead	C41.2-Vertebral column
Alive	C41.2-Vertebral column
Alive	C41.2-Vertebral column
Dead	C41.3-Rib, sternum, clavicle and associated joints
Dead	C41.2-Vertebral column
Alive	C40.2-Long bones of lower limb and associated joints
Dead	C40.2-Long bones of lower limb and associated joints
Alive	C40.2-Long bones of lower limb and associated joints
Alive	C40.2-Long bones of lower limb and associated joints
Alive	C40.3-Short bones of lower limb and associated joints
Dead	C40.2-Long bones of lower limb and associated joints
Alive	C41.1-Mandible
Dead	C40.0-Long bones: upper limb, scapula, and associated joints
Alive	C40.2-Long bones of lower limb and associated joints
Dead	C40.2-Long bones of lower limb and associated joints
Alive	C40.2-Long bones of lower limb and associated joints
Alive	C41.4-Pelvic bones, sacrum, coccyx and associated joints
Alive	C41.4-Pelvic bones, sacrum, coccyx and associated joints
Alive	C41.4-Pelvic bones, sacrum, coccyx and associated joints
Alive	C40.2-Long bones of lower limb and associated joints
Dead	C40.2-Long bones of lower limb and associated joints
Alive	C40.2-Long bones of lower limb and associated joints
Alive	C40.2-Long bones of lower limb and associated joints
Alive	C40.3-Short bones of lower limb and associated joints
Alive	C40.2-Long bones of lower limb and associated joints
Alive	C40.0-Long bones: upper limb, scapula, and associated joints
Alive	C41.2-Vertebral column
Dead	C41.4-Pelvic bones, sacrum, coccyx and associated joints
Alive	C41.3-Rib, sternum, clavicle and associated joints
Alive	C41.2-Vertebral column
Alive	C40.0-Long bones: upper limb, scapula, and associated joints
Alive	C40.2-Long bones of lower limb and associated joints
Alive	C41.4-Pelvic bones, sacrum, coccyx and associated joints
Dead	C40.2-Long bones of lower limb and associated joints
Dead	C41.4-Pelvic bones, sacrum, coccyx and associated joints
Dead	C40.0-Long bones: upper limb, scapula, and associated joints
Alive	C41.9-Bone, NOS
Dead	C41.4-Pelvic bones, sacrum, coccyx and associated joints
Alive	C41.4-Pelvic bones, sacrum, coccyx and associated joints
Alive	C41.0-Bones of skull and face and associated joints
Dead	C41.3-Rib, sternum, clavicle and associated joints
Alive	C41.4-Pelvic bones, sacrum, coccyx and associated joints
Dead	C40.2-Long bones of lower limb and associated joints
Alive	C41.4-Pelvic bones, sacrum, coccyx and associated joints
Alive	C41.1-Mandible
Alive	C40.2-Long bones of lower limb and associated joints
Alive	C41.2-Vertebral column
Alive	C41.2-Vertebral column
Dead	C40.2-Long bones of lower limb and associated joints
Dead	C40.2-Long bones of lower limb and associated joints
Dead	C41.3-Rib, sternum, clavicle and associated joints
Dead	C40.2-Long bones of lower limb and associated joints
Dead	C41.4-Pelvic bones, sacrum, coccyx and associated joints
Alive	C41.0-Bones of skull and face and associated joints

Alive	C41.2-Vertebral column
Dead	C41.4-Pelvic bones, sacrum, coccyx and associated joints
Alive	C41.4-Pelvic bones, sacrum, coccyx and associated joints
Alive	C40.2-Long bones of lower limb and associated joints
Alive	C41.3-Rib, sternum, clavicle and associated joints
Alive	C41.2-Vertebral column
Alive	C40.2-Long bones of lower limb and associated joints
Dead	C40.2-Long bones of lower limb and associated joints
Alive	C41.4-Pelvic bones, sacrum, coccyx and associated joints
Alive	C41.3-Rib, sternum, clavicle and associated joints
Alive	C40.2-Long bones of lower limb and associated joints
Alive	C40.0-Long bones: upper limb, scapula, and associated joints
Alive	C40.0-Long bones: upper limb, scapula, and associated joints
Alive	C40.2-Long bones of lower limb and associated joints
Alive	C40.0-Long bones: upper limb, scapula, and associated joints
Alive	C41.4-Pelvic bones, sacrum, coccyx and associated joints
Alive	C41.3-Rib, sternum, clavicle and associated joints
