# Supplementary material for: Overall survival comparison between pediatric and adult Ewing sarcoma of bone and adult nomogram construction: a large population-based analysis
Source: Front Pediatr. 2023 May 23;11:1103565. doi: 10.3389/fped.2023.1103565 (PMC10242502; doi:10.3389/fped.2023.1103565)
Supplement: Supplementary file 2 [file Datasheet2.pdf]

| Age | Race | Sex | T.stage | N.stage | M.stage | Surgery | Radiation |
|---|---|---|---|---|---|---|---|
| <18 | White | Female | T2 | N0 | M0 | No | Yes |
| <18 | White | Male | T3 | N0 | M1 | No | Yes |
| <18 | White | Female | T2 | N0 | M1 | No | Yes |
| >=28 | White | Female | T2 | N0 | M1 | Yes | None/Unknown |
| <18 | White | Female | T1 | N0 | M0 | Yes | None/Unknown |
| <18 | White | Male | T1 | N0 | M0 | No | Yes |
| <18 | Others | Female | T2 | N0 | M0 | No | Yes |
| >=28 | White | Male | T3 | N0 | M1 | No | None/Unknown |
| >=28 | White | Female | T1 | N0 | M0 | Yes | Yes |
| <18 | White | Female | T2 | N1 | M0 | Yes | None/Unknown |
| >=28 | Others | Female | T2 | N0 | M0 | No | Yes |
| <18 | White | Female | T1 | N0 | M0 | Yes | None/Unknown |
| >=28 | White | Female | T2 | N0 | M1 | No | None/Unknown |
| >=28 | White | Male | T1 | N0 | M0 | Yes | None/Unknown |
| <18 | White | Female | T1 | N1 | M0 | Yes | Yes |
| <18 | White | Female | T1 | N0 | M0 | No | Yes |
| <18 | White | Male | T2 | N0 | M1 | Yes | Yes |
| <18 | White | Female | T2 | N0 | M0 | No | Yes |
| <18 | Others | Female | T1 | N0 | M0 | No | Yes |
| >=28 | White | Female | T2 | N0 | M0 | Yes | Yes |
| >=28 | White | Male | T1 | N0 | M1 | Yes | Yes |
| <18 | Others | Female | T1 | N0 | M0 | No | Yes |
| >=28 | White | Male | T1 | N0 | M0 | Yes | None/Unknown |
| >=28 | White | Male | T2 | N0 | M0 | No | Yes |
| <18 | White | Male | T1 | N0 | M1 | Yes | Yes |
| <18 | White | Male | T2 | N0 | M0 | Yes | None/Unknown |
| >=28 | White | Female | T1 | N0 | M1 | Yes | Yes |
| >=28 | White | Female | T2 | N0 | M0 | Yes | None/Unknown |
| <18 | White | Female | T2 | N1 | M1 | No | Yes |
| >=28 | White | Female | T2 | N0 | M0 | No | Yes |
| <18 | Others | Male | T2 | N0 | M1 | No | Yes |
| >=28 | White | Female | T2 | N0 | M1 | No | Yes |
| >=28 | White | Male | T1 | N0 | M0 | Yes | None/Unknown |
| <18 | Others | Male | T1 | N0 | M0 | No | None/Unknown |
| <18 | White | Female | T3 | N0 | M1 | No | Yes |
| <18 | White | Female | T1 | N0 | M0 | No | Yes |
| <18 | Others | Female | T2 | N0 | M0 | No | Yes |
| >=28 | White | Female | T2 | N0 | M0 | Yes | None/Unknown |
| <18 | Others | Male | T2 | N0 | M1 | No | Yes |
| >=28 | White | Female | T3 | N0 | M1 | Yes | Yes |
| <18 | White | Female | T1 | N0 | M0 | No | Yes |
| <18 | White | Female | T2 | N0 | M1 | No | Yes |
| <18 | Black | Female | T2 | N0 | M0 | No | Yes |
| <18 | White | Male | T1 | N0 | M0 | Yes | Yes |
| >=28 | White | Male | T1 | N0 | M0 | Yes | Yes |
| >=28 | White | Female | T1 | N0 | M0 | No | Yes |
| >=28 | White | Female | T1 | N0 | M0 | Yes | Yes |
| <18 | White | Female | T1 | N0 | M0 | Yes | None/Unknown |
| <18 | White | Female | T1 | N0 | M0 | Yes | None/Unknown |
| >=28 | White | Male | T1 | N0 | M0 | Yes | None/Unknown |
| >=28 | White | Female | T2 | N0 | M1 | No | Yes |
| <18 | White | Male | T2 | N0 | M0 | Yes | Yes |
| >=28 | White | Male | T1 | N0 | M0 | Yes | None/Unknown |
| >=28 | White | Female | T1 | N0 | M0 | Yes | Yes |
| <18 | White | Female | T2 | N0 | M0 | No | None/Unknown |
| >=28 | White | Female | T1 | N0 | M0 | Yes | None/Unknown |
| <18 | White | Male | T1 | N0 | M0 | Yes | Yes |

| | | | | | | | |
|---|---|---|---|---|---|---|---|
| >=28 | White | Female | T1 | N0 | M1 | No | None/Unknown |
| >=28 | White | Female | T2 | N0 | M0 | Yes | Yes |
| <18 | White | Male | T3 | N0 | M1 | No | None/Unknown |
| <18 | White | Male | T1 | N0 | M0 | Yes | None/Unknown |
| >=28 | White | Male | T1 | N0 | M0 | Yes | Yes |
| <18 | White | Female | T3 | N0 | M1 | No | Yes |
| <18 | White | Female | T1 | N0 | M0 | Yes | Yes |
| >=28 | White | Female | T1 | N0 | M0 | Yes | None/Unknown |
| <18 | White | Male | T1 | N0 | M1 | No | Yes |
| <18 | White | Male | T2 | N0 | M0 | Yes | None/Unknown |
| >=28 | White | Female | T1 | N0 | M0 | Yes | None/Unknown |
| <18 | White | Female | T1 | N0 | M0 | Yes | None/Unknown |
| >=28 | Black | Female | T2 | N0 | M0 | Yes | Yes |
| <18 | White | Male | T1 | N0 | M0 | Yes | Yes |
| >=28 | White | Female | T2 | N0 | M0 | Yes | None/Unknown |
| <18 | White | Female | T2 | N0 | M1 | No | Yes |
| >=28 | White | Male | T2 | N0 | M1 | No | Yes |
| <18 | White | Male | T1 | N0 | M1 | Yes | None/Unknown |
| <18 | White | Male | T1 | N0 | M0 | Yes | None/Unknown |
| <18 | White | Female | T1 | N0 | M0 | Yes | None/Unknown |
| <18 | Others | Female | T1 | N0 | M0 | Yes | Yes |
| <18 | Others | Female | T1 | N1 | M0 | Yes | None/Unknown |
| <18 | Others | Male | T1 | N0 | M0 | Yes | None/Unknown |
| >=28 | Others | Female | T3 | N0 | M1 | No | Yes |
| >=28 | Others | Male | T1 | N1 | M1 | Yes | None/Unknown |
| <18 | White | Female | T2 | N1 | M0 | No | None/Unknown |
| <18 | Others | Male | T1 | N0 | M1 | No | Yes |
| <18 | Others | Female | T2 | N0 | M1 | Yes | None/Unknown |
| >=28 | White | Female | T3 | N0 | M1 | No | None/Unknown |
| >=28 | White | Male | T1 | N0 | M0 | No | Yes |
| <18 | White | Male | T1 | N0 | M0 | Yes | None/Unknown |
| <18 | White | Female | T1 | N0 | M0 | Yes | None/Unknown |
| >=28 | White | Female | T2 | N0 | M0 | No | None/Unknown |
| <18 | White | Female | T2 | N0 | M1 | No | None/Unknown |
| <18 | White | Female | T1 | N0 | M0 | Yes | None/Unknown |
| <18 | White | Male | T1 | N0 | M0 | Yes | None/Unknown |
| <18 | White | Male | T1 | N0 | M0 | Yes | None/Unknown |
| >=28 | White | Female | T1 | N0 | M0 | Yes | Yes |
| <18 | White | Male | T1 | N0 | M0 | Yes | None/Unknown |
| >=28 | White | Female | T2 | N0 | M1 | No | None/Unknown |
| >=28 | White | Female | T1 | N0 | M0 | Yes | None/Unknown |
| <18 | White | Female | T2 | N0 | M0 | Yes | None/Unknown |
| <18 | White | Female | T1 | N0 | M0 | Yes | None/Unknown |
| <18 | White | Female | T2 | N0 | M0 | No | Yes |
| <18 | White | Female | T2 | N0 | M0 | No | Yes |
| <18 | White | Female | T1 | N0 | M1 | Yes | Yes |
| <18 | White | Female | T2 | N0 | M0 | No | Yes |
| <18 | White | Female | T2 | N0 | M1 | Yes | Yes |
| >=28 | White | Male | T1 | N0 | M0 | No | Yes |
| >=28 | White | Male | T2 | N0 | M0 | No | Yes |
| <18 | White | Male | T2 | N0 | M0 | Yes | None/Unknown |
| >=28 | White | Female | T2 | N0 | M0 | Yes | None/Unknown |
| >=28 | White | Female | T1 | N0 | M0 | Yes | None/Unknown |
| >=28 | White | Female | T1 | N1 | M1 | Yes | None/Unknown |
| >=28 | White | Female | T1 | N0 | M1 | No | None/Unknown |
| <18 | White | Female | T3 | N0 | M1 | No | Yes |
| >=28 | White | Male | T2 | N0 | M0 | Yes | None/Unknown |
| <18 | White | Male | T1 | N0 | M0 | Yes | None/Unknown |

| | | | | | | | |
|---|---|---|---|---|---|---|---|
| <18 | White | Female | T2 | N0 | M0 | Yes | None/Unknown |
| <18 | White | Female | T2 | N0 | M0 | Yes | Yes |
| <18 | Others | Male | T1 | N0 | M0 | Yes | None/Unknown |
| >=28 | White | Male | T1 | N0 | M0 | Yes | None/Unknown |
| >=28 | White | Female | T3 | N0 | M1 | No | Yes |
| <18 | White | Female | T2 | N0 | M0 | Yes | Yes |
| <18 | White | Female | T1 | N0 | M0 | No | None/Unknown |
| >=28 | White | Female | T1 | N0 | M0 | No | Yes |
| <18 | White | Male | T2 | N0 | M0 | Yes | None/Unknown |
| <18 | White | Male | T2 | N0 | M0 | Yes | None/Unknown |
| <18 | White | Female | T2 | N0 | M0 | No | None/Unknown |
| <18 | White | Female | T2 | N0 | M1 | No | None/Unknown |
| <18 | Others | Female | T3 | N0 | M0 | Yes | None/Unknown |
| <18 | White | Male | T1 | N0 | M0 | Yes | Yes |
| >=28 | White | Male | T1 | N0 | M1 | Yes | Yes |
| <18 | Others | Female | T1 | N0 | M0 | Yes | Yes |
| >=28 | White | Female | T2 | N0 | M0 | Yes | None/Unknown |
| >=28 | White | Female | T2 | N0 | M0 | Yes | Yes |
| <18 | White | Female | T1 | N0 | M0 | No | Yes |
| <18 | Others | Male | T2 | N0 | M1 | Yes | Yes |
| <18 | White | Female | T1 | N0 | M0 | No | Yes |
| >=28 | White | Male | T1 | N0 | M0 | Yes | Yes |
| <18 | White | Male | T1 | N0 | M0 | No | None/Unknown |
| <18 | White | Female | T1 | N0 | M0 | No | None/Unknown |
| >=28 | White | Female | T2 | N0 | M1 | No | Yes |
| <18 | White | Female | T1 | N0 | M0 | Yes | None/Unknown |
| >=28 | White | Female | T2 | N0 | M1 | Yes | Yes |
| >=28 | White | Female | T2 | N0 | M0 | Yes | Yes |
| <18 | White | Male | T1 | N0 | M0 | Yes | None/Unknown |
| >=28 | White | Male | T1 | N0 | M1 | No | None/Unknown |
| <18 | White | Female | T2 | N0 | M0 | No | Yes |
| >=28 | White | Male | T1 | N0 | M0 | No | Yes |
| >=28 | White | Female | T2 | N0 | M0 | No | Yes |
| <18 | White | Male | T1 | N0 | M0 | Yes | None/Unknown |
| <18 | White | Female | T1 | N0 | M1 | Yes | Yes |
| >=28 | White | Female | T1 | N0 | M0 | Yes | None/Unknown |
| >=28 | White | Male | T2 | N0 | M0 | Yes | None/Unknown |
| <18 | White | Female | T2 | N0 | M0 | Yes | Yes |
| >=28 | White | Male | T1 | N0 | M0 | Yes | Yes |
| >=28 | White | Female | T2 | N0 | M0 | Yes | None/Unknown |
| <18 | White | Female | T1 | N0 | M0 | Yes | None/Unknown |
| <18 | White | Female | T2 | N0 | M0 | Yes | None/Unknown |
| >=28 | White | Female | T1 | N0 | M0 | Yes | None/Unknown |
| >=28 | White | Female | T2 | N0 | M0 | No | Yes |
| >=28 | White | Female | T1 | N0 | M1 | Yes | Yes |
| <18 | White | Male | T1 | N0 | M0 | Yes | Yes |
| >=28 | Others | Female | T2 | N0 | M0 | No | Yes |
| >=28 | White | Female | T2 | N0 | M0 | Yes | Yes |
| <18 | White | Male | T2 | N0 | M0 | Yes | None/Unknown |
| <18 | White | Female | T1 | N0 | M0 | Yes | Yes |
| <18 | White | Female | T1 | N0 | M0 | Yes | None/Unknown |
| >=28 | Others | Female | T2 | N0 | M1 | No | Yes |
| <18 | White | Male | T2 | N0 | M0 | No | Yes |
| <18 | White | Male | T2 | N0 | M0 | Yes | None/Unknown |
| <18 | White | Female | T2 | N0 | M0 | Yes | None/Unknown |
| >=28 | White | Female | T2 | N1 | M0 | Yes | Yes |
| <18 | White | Male | T1 | N0 | M0 | No | Yes |
| <18 | White | Female | T1 | N0 | M0 | Yes | None/Unknown |

| | | | | | | | |
|---|---|---|---|---|---|---|---|
| >=28 | White | Female | T2 | N0 | M1 | No | Yes |
| >=28 | White | Female | T2 | N0 | M0 | No | Yes |
| <18 | White | Female | T1 | N0 | M0 | Yes | None/Unknown |
| <18 | White | Female | T2 | N0 | M0 | Yes | None/Unknown |
| <18 | White | Female | T2 | N0 | M1 | Yes | Yes |
| >=28 | White | Male | T1 | N0 | M0 | Yes | None/Unknown |
| >=28 | White | Female | T1 | N0 | M1 | No | Yes |
| >=28 | White | Female | T2 | N0 | M1 | No | Yes |
| >=28 | White | Female | T2 | N0 | M0 | Yes | Yes |
| <18 | White | Female | T2 | N1 | M1 | No | Yes |
| >=28 | White | Female | T1 | N0 | M0 | Yes | None/Unknown |
| <18 | White | Female | T3 | N0 | M0 | Yes | Yes |
| <18 | White | Female | T2 | N0 | M0 | Yes | Yes |
| <18 | White | Male | T1 | N1 | M0 | No | Yes |
| >=28 | White | Female | T2 | N0 | M1 | No | Yes |
| <18 | White | Female | T2 | N0 | M0 | Yes | Yes |
| <18 | White | Male | T2 | N0 | M1 | No | Yes |
| <18 | White | Male | T2 | N0 | M0 | Yes | None/Unknown |
| <18 | White | Male | T1 | N0 | M0 | Yes | Yes |
| <18 | White | Male | T2 | N0 | M0 | Yes | None/Unknown |
| <18 | White | Female | T2 | N0 | M0 | Yes | None/Unknown |
| <18 | White | Male | T1 | N0 | M0 | Yes | Yes |
| >=28 | White | Male | T3 | N0 | M1 | Yes | Yes |
| <18 | White | Male | T1 | N0 | M0 | Yes | Yes |
| <18 | White | Female | T1 | N0 | M0 | Yes | None/Unknown |
| >=28 | White | Male | T2 | N0 | M1 | No | Yes |
| <18 | White | Female | T2 | N0 | M0 | Yes | None/Unknown |
| <18 | White | Female | T1 | N0 | M0 | Yes | None/Unknown |
| <18 | White | Female | T1 | N0 | M0 | Yes | Yes |
| >=28 | White | Female | T1 | N0 | M0 | Yes | None/Unknown |
| <18 | White | Female | T1 | N0 | M0 | No | Yes |
| <18 | White | Female | T1 | N0 | M0 | Yes | None/Unknown |
| <18 | White | Female | T2 | N1 | M1 | No | Yes |
| >=28 | White | Female | T1 | N0 | M1 | No | Yes |
| <18 | White | Female | T1 | N0 | M0 | No | Yes |
| <18 | White | Male | T2 | N1 | M1 | No | None/Unknown |
| >=28 | White | Female | T2 | N0 | M0 | No | Yes |
| <18 | White | Female | T2 | N0 | M0 | No | Yes |
| <18 | White | Female | T2 | N0 | M1 | Yes | Yes |
| >=28 | White | Female | T1 | N0 | M1 | No | Yes |
| >=28 | White | Female | T1 | N0 | M0 | Yes | None/Unknown |
| <18 | White | Male | T2 | N0 | M0 | Yes | Yes |
| >=28 | White | Female | T2 | N0 | M0 | Yes | Yes |
| <18 | White | Male | T1 | N0 | M0 | Yes | None/Unknown |
| >=28 | White | Male | T1 | N0 | M1 | Yes | None/Unknown |
| <18 | White | Male | T3 | N0 | M0 | Yes | None/Unknown |
| >=28 | White | Female | T1 | N0 | M0 | Yes | None/Unknown |
| <18 | White | Female | T1 | N0 | M0 | No | None/Unknown |
| <18 | White | Female | T2 | N0 | M0 | No | None/Unknown |
| <18 | White | Male | T1 | N0 | M1 | No | Yes |
| >=28 | Black | Female | T1 | N0 | M0 | Yes | Yes |
| >=28 | White | Male | T2 | N0 | M0 | Yes | None/Unknown |
| <18 | White | Female | T2 | N0 | M1 | Yes | Yes |
| <18 | White | Male | T2 | N0 | M1 | No | Yes |
| >=28 | White | Female | T2 | N0 | M0 | Yes | Yes |
| >=28 | White | Female | T2 | N0 | M0 | No | Yes |
| <18 | White | Male | T2 | N0 | M0 | Yes | None/Unknown |
| >=28 | White | Female | T2 | N0 | M0 | Yes | None/Unknown |

| | | | | | | | |
|---|---|---|---|---|---|---|---|
| >=28 | White | Female | T3 | N0 | M1 | No | None/Unknown |
| >=28 | Black | Female | T1 | N0 | M0 | No | Yes |
| >=28 | White | Female | T1 | N0 | M0 | Yes | None/Unknown |
| <18 | White | Male | T1 | N0 | M0 | Yes | None/Unknown |
| <18 | White | Male | T2 | N0 | M1 | Yes | Yes |
| <18 | White | Male | T2 | N0 | M0 | No | Yes |
| >=28 | Others | Female | T2 | N0 | M0 | No | Yes |
| <18 | Others | Male | T1 | N0 | M1 | Yes | Yes |
| <18 | White | Female | T2 | N0 | M1 | No | Yes |
| <18 | White | Female | T2 | N0 | M1 | Yes | None/Unknown |
| >=28 | Others | Female | T1 | N0 | M0 | Yes | None/Unknown |
| >=28 | Others | Female | T2 | N1 | M0 | Yes | None/Unknown |
| >=28 | Others | Female | T1 | N0 | M0 | Yes | Yes |
| <18 | White | Female | T2 | N0 | M0 | Yes | None/Unknown |
| >=28 | White | Female | T2 | N0 | M0 | No | Yes |
| >=28 | White | Female | T2 | N0 | M0 | No | None/Unknown |
| <18 | White | Male | T1 | N1 | M0 | No | Yes |
| >=28 | Black | Female | T2 | N0 | M0 | Yes | None/Unknown |
| >=28 | White | Female | T2 | N0 | M0 | Yes | None/Unknown |
| <18 | White | Male | T2 | N0 | M0 | No | Yes |
| <18 | White | Female | T2 | N0 | M0 | Yes | None/Unknown |
| >=28 | White | Female | T1 | N0 | M1 | Yes | Yes |
| <18 | White | Male | T2 | N0 | M0 | Yes | None/Unknown |
| <18 | White | Female | T1 | N0 | M0 | No | None/Unknown |
| >=28 | White | Male | T2 | N0 | M0 | Yes | Yes |
| <18 | Black | Female | T1 | N0 | M0 | Yes | None/Unknown |
| <18 | White | Female | T1 | N0 | M0 | Yes | Yes |
| >=28 | White | Female | T2 | N0 | M1 | Yes | Yes |
| <18 | White | Female | T2 | N1 | M1 | No | Yes |
| >=28 | White | Male | T1 | N0 | M1 | No | None/Unknown |
| >=28 | White | Male | T1 | N0 | M1 | No | Yes |
| <18 | White | Female | T1 | N0 | M0 | Yes | None/Unknown |
| <18 | Others | Female | T1 | N0 | M0 | No | Yes |
| <18 | White | Female | T2 | N0 | M1 | No | Yes |
| >=28 | White | Female | T2 | N1 | M1 | No | Yes |
| <18 | White | Female | T2 | N0 | M1 | Yes | Yes |
| <18 | White | Female | T1 | N0 | M0 | Yes | None/Unknown |
| >=28 | White | Male | T2 | N0 | M0 | Yes | Yes |
| >=28 | White | Female | T2 | N0 | M0 | Yes | Yes |
| >=28 | White | Female | T2 | N0 | M0 | Yes | Yes |
| <18 | White | Female | T1 | N0 | M1 | No | None/Unknown |
| <18 | White | Male | T2 | N0 | M0 | Yes | Yes |
| <18 | White | Male | T2 | N0 | M0 | Yes | Yes |
| <18 | White | Female | T2 | N0 | M1 | No | Yes |
| >=28 | White | Male | T1 | N0 | M0 | Yes | Yes |
| <18 | White | Female | T1 | N0 | M1 | Yes | None/Unknown |
| >=28 | White | Female | T1 | N0 | M0 | Yes | Yes |
| >=28 | White | Male | T1 | N0 | M0 | Yes | Yes |
| <18 | White | Male | T2 | N0 | M0 | Yes | Yes |
| <18 | White | Female | T2 | N1 | M0 | Yes | None/Unknown |
| >=28 | White | Female | T2 | N0 | M0 | No | Yes |
| >=28 | White | Male | T1 | N0 | M1 | Yes | Yes |
| <18 | White | Female | T1 | N1 | M0 | Yes | Yes |
| <18 | White | Female | T2 | N0 | M0 | Yes | None/Unknown |
| <18 | White | Female | T2 | N0 | M0 | Yes | None/Unknown |
| <18 | White | Female | T1 | N0 | M0 | Yes | None/Unknown |
| <18 | White | Female | T2 | N1 | M0 | Yes | Yes |
| <18 | Others | Female | T1 | N0 | M0 | No | None/Unknown |

| | | | | | | | |
|---|---|---|---|---|---|---|---|
| >=28 | White | Female | T2 | N0 | M0 | Yes | Yes |
| <18 | White | Female | T2 | N0 | M1 | No | Yes |
| >=28 | White | Female | T1 | N0 | M0 | Yes | Yes |
| >=28 | White | Female | T2 | N1 | M0 | Yes | None/Unknown |
| >=28 | White | Female | T2 | N0 | M0 | Yes | Yes |
| <18 | White | Male | T2 | N1 | M1 | Yes | None/Unknown |
| <18 | White | Female | T2 | N0 | M0 | Yes | None/Unknown |
| <18 | White | Male | T2 | N0 | M0 | Yes | None/Unknown |
| <18 | White | Male | T1 | N0 | M0 | Yes | None/Unknown |
| >=28 | White | Female | T1 | N0 | M0 | Yes | Yes |
| <18 | White | Male | T1 | N0 | M0 | No | None/Unknown |
| <18 | White | Female | T2 | N0 | M0 | No | Yes |
| >=28 | Others | Male | T1 | N0 | M0 | Yes | None/Unknown |
| <18 | White | Female | T3 | N0 | M1 | No | None/Unknown |
| <18 | White | Female | T1 | N0 | M0 | Yes | None/Unknown |
| <18 | White | Male | T2 | N0 | M1 | Yes | Yes |
| <18 | White | Female | T2 | N0 | M0 | No | Yes |
| >=28 | White | Female | T2 | N0 | M1 | No | Yes |
| <18 | White | Male | T1 | N0 | M0 | Yes | None/Unknown |
| >=28 | White | Male | T1 | N0 | M0 | Yes | None/Unknown |
| <18 | White | Female | T1 | N0 | M0 | Yes | Yes |
| >=28 | White | Male | T1 | N0 | M0 | Yes | None/Unknown |
| >=28 | Black | Male | T2 | N0 | M1 | No | None/Unknown |
| >=28 | White | Female | T2 | N0 | M0 | No | None/Unknown |
| <18 | White | Female | T1 | N0 | M0 | Yes | None/Unknown |
| >=28 | White | Male | T3 | N0 | M0 | No | None/Unknown |
| >=28 | White | Female | T1 | N0 | M0 | Yes | None/Unknown |
| >=28 | White | Female | T2 | N0 | M1 | No | None/Unknown |
| >=28 | White | Male | T2 | N0 | M1 | No | None/Unknown |
| <18 | White | Male | T1 | N0 | M0 | Yes | None/Unknown |
| <18 | White | Male | T1 | N1 | M0 | No | None/Unknown |
| >=28 | White | Male | T1 | N0 | M0 | No | Yes |
| >=28 | White | Female | T3 | N0 | M1 | No | None/Unknown |
| <18 | White | Male | T1 | N0 | M0 | Yes | None/Unknown |
| >=28 | White | Female | T2 | N0 | M1 | Yes | Yes |
| <18 | White | Male | T1 | N0 | M0 | Yes | None/Unknown |
| <18 | White | Male | T1 | N1 | M0 | Yes | Yes |
| <18 | Black | Female | T2 | N0 | M0 | Yes | None/Unknown |
| <18 | White | Female | T1 | N0 | M1 | Yes | Yes |
| >=28 | Others | Male | T2 | N0 | M0 | No | None/Unknown |
| >=28 | White | Male | T1 | N0 | M0 | No | None/Unknown |
| <18 | White | Female | T2 | N0 | M0 | Yes | None/Unknown |
| <18 | White | Female | T2 | N0 | M0 | Yes | None/Unknown |
| <18 | White | Male | T1 | N0 | M0 | Yes | None/Unknown |
| <18 | White | Male | T2 | N0 | M0 | Yes | None/Unknown |
| <18 | White | Female | T1 | N0 | M0 | Yes | None/Unknown |
| >=28 | White | Female | T2 | N1 | M1 | No | None/Unknown |
| <18 | White | Male | T2 | N0 | M0 | No | Yes |
| >=28 | White | Female | T1 | N0 | M0 | Yes | None/Unknown |
| <18 | White | Female | T2 | N1 | M1 | Yes | Yes |
| >=28 | White | Female | T2 | N0 | M0 | Yes | None/Unknown |
| <18 | White | Female | T2 | N0 | M0 | No | None/Unknown |
| <18 | White | Female | T2 | N0 | M1 | Yes | Yes |
| <18 | White | Male | T1 | N0 | M0 | Yes | Yes |
| <18 | White | Male | T2 | N0 | M0 | Yes | None/Unknown |
| <18 | White | Female | T2 | N0 | M1 | Yes | Yes |
| <18 | White | Female | T1 | N0 | M0 | Yes | None/Unknown |
| <18 | White | Female | T2 | N1 | M1 | No | None/Unknown |

| | | | | | | | |
|---|---|---|---|---|---|---|---|
| <18 | White | Female | T3 | N0 | M1 | No | None/Unknown |
| <18 | White | Female | T3 | N1 | M1 | Yes | None/Unknown |
| <18 | White | Female | T1 | N0 | M0 | No | Yes |
| >=28 | White | Female | T1 | N0 | M0 | Yes | None/Unknown |
| >=28 | White | Male | T1 | N0 | M0 | Yes | None/Unknown |
| <18 | White | Female | T1 | N0 | M0 | No | None/Unknown |
| >=28 | White | Male | T2 | N0 | M0 | Yes | Yes |
| >=28 | Others | Male | T3 | N1 | M1 | No | Yes |
| >=28 | White | Female | T2 | N0 | M0 | Yes | None/Unknown |
| <18 | White | Female | T1 | N0 | M0 | Yes | None/Unknown |
| >=28 | White | Female | T3 | N0 | M1 | No | None/Unknown |
| >=28 | White | Female | T3 | N0 | M1 | No | None/Unknown |
| >=28 | White | Female | T2 | N0 | M0 | Yes | Yes |
| >=28 | White | Male | T3 | N0 | M1 | No | None/Unknown |
| <18 | White | Female | T1 | N0 | M1 | No | Yes |
| <18 | White | Female | T1 | N1 | M0 | No | None/Unknown |
| >=28 | Others | Male | T1 | N0 | M0 | Yes | Yes |
| >=28 | White | Female | T2 | N0 | M0 | Yes | Yes |
| >=28 | White | Female | T2 | N1 | M1 | Yes | None/Unknown |
| <18 | White | Female | T2 | N0 | M1 | Yes | Yes |
| >=28 | White | Female | T2 | N0 | M0 | No | Yes |
| <18 | White | Female | T2 | N0 | M0 | Yes | None/Unknown |
| <18 | White | Male | T1 | N0 | M0 | No | None/Unknown |
| <18 | White | Female | T1 | N0 | M0 | Yes | None/Unknown |
| <18 | White | Female | T1 | N0 | M0 | Yes | Yes |
| <18 | White | Female | T2 | N0 | M0 | No | Yes |
| >=28 | White | Female | T3 | N0 | M1 | No | Yes |
| <18 | White | Female | T1 | N0 | M1 | Yes | None/Unknown |
| >=28 | White | Female | T2 | N0 | M0 | Yes | Yes |
| <18 | White | Female | T1 | N0 | M1 | Yes | Yes |
| >=28 | White | Female | T1 | N0 | M0 | Yes | None/Unknown |
| <18 | White | Female | T2 | N0 | M0 | Yes | None/Unknown |
| <18 | Black | Female | T2 | N0 | M0 | Yes | None/Unknown |
| <18 | White | Female | T2 | N0 | M0 | Yes | None/Unknown |
| >=28 | White | Female | T1 | N0 | M0 | No | Yes |
| <18 | White | Female | T1 | N0 | M1 | No | Yes |
| >=28 | White | Male | T1 | N0 | M0 | No | Yes |
| <18 | White | Female | T2 | N0 | M1 | Yes | None/Unknown |
| >=28 | White | Female | T2 | N1 | M0 | Yes | Yes |
| <18 | White | Female | T2 | N0 | M0 | Yes | None/Unknown |
| <18 | White | Male | T1 | N0 | M0 | No | Yes |
| <18 | White | Female | T2 | N0 | M0 | Yes | Yes |
| >=28 | Others | Female | T2 | N0 | M0 | No | Yes |
| <18 | White | Female | T1 | N0 | M0 | Yes | None/Unknown |
| <18 | White | Female | T2 | N0 | M0 | Yes | Yes |
| >=28 | White | Female | T1 | N0 | M0 | No | Yes |
| >=28 | White | Female | T2 | N0 | M1 | No | Yes |
| >=28 | White | Female | T2 | N0 | M0 | Yes | None/Unknown |
| >=28 | White | Female | T2 | N0 | M0 | Yes | Yes |
| <18 | White | Female | T2 | N0 | M0 | Yes | None/Unknown |
| <18 | White | Female | T1 | N0 | M1 | Yes | Yes |
| >=28 | White | Female | T1 | N0 | M1 | No | Yes |
| >=28 | White | Male | T2 | N0 | M0 | Yes | None/Unknown |
| >=28 | White | Male | T2 | N0 | M1 | No | Yes |
| >=28 | White | Female | T2 | N0 | M0 | No | Yes |
| >=28 | White | Female | T3 | N0 | M1 | No | Yes |
| <18 | White | Female | T1 | N0 | M0 | Yes | None/Unknown |
| >=28 | White | Male | T1 | N0 | M0 | No | Yes |

| | | | | | | | |
|---|---|---|---|---|---|---|---|
| >=28 | White | Female | T1 | N0 | M0 | Yes | None/Unknown |
| >=28 | White | Male | T1 | N0 | M0 | No | Yes |
| <18 | White | Female | T2 | N0 | M0 | Yes | None/Unknown |
| >=28 | White | Male | T1 | N0 | M0 | Yes | None/Unknown |
| >=28 | Others | Male | T1 | N0 | M0 | No | Yes |
| >=28 | White | Female | T1 | N0 | M1 | No | Yes |
| <18 | White | Female | T2 | N0 | M1 | Yes | None/Unknown |
| >=28 | White | Female | T2 | N0 | M0 | Yes | None/Unknown |
| >=28 | Others | Female | T2 | N0 | M0 | Yes | None/Unknown |
| >=28 | White | Female | T1 | N0 | M0 | Yes | Yes |
| >=28 | White | Female | T2 | N0 | M1 | No | Yes |
| >=28 | White | Female | T2 | N0 | M0 | No | Yes |
| <18 | White | Male | T2 | N0 | M1 | No | None/Unknown |
| <18 | White | Female | T2 | N0 | M1 | Yes | Yes |
| >=28 | White | Female | T2 | N0 | M1 | Yes | None/Unknown |
| <18 | White | Female | T1 | N0 | M0 | Yes | None/Unknown |
| >=28 | White | Female | T2 | N0 | M1 | No | Yes |
| <18 | White | Female | T2 | N1 | M0 | Yes | Yes |
| <18 | Others | Female | T1 | N0 | M1 | No | Yes |
| >=28 | White | Female | T2 | N0 | M1 | No | Yes |
| <18 | White | Female | T2 | N0 | M1 | No | None/Unknown |
| <18 | White | Female | T1 | N0 | M1 | Yes | Yes |
| <18 | White | Female | T2 | N0 | M1 | Yes | None/Unknown |
| >=28 | White | Male | T1 | N0 | M0 | No | Yes |
| <18 | White | Female | T2 | N1 | M0 | No | Yes |
| >=28 | White | Male | T1 | N1 | M1 | Yes | Yes |
| <18 | Others | Female | T2 | N0 | M1 | No | Yes |
| >=28 | White | Female | T1 | N0 | M0 | Yes | Yes |
| <18 | White | Female | T1 | N0 | M0 | Yes | None/Unknown |
| >=28 | Others | Female | T2 | N0 | M1 | No | Yes |
| >=28 | White | Female | T1 | N0 | M0 | No | Yes |
| <18 | White | Female | T2 | N0 | M0 | No | Yes |
| <18 | White | Male | T2 | N0 | M1 | No | Yes |
| <18 | White | Female | T2 | N1 | M1 | No | None/Unknown |
| >=28 | White | Male | T1 | N0 | M0 | Yes | Yes |
| >=28 | White | Female | T1 | N0 | M0 | Yes | Yes |
| >=28 | White | Male | T2 | N0 | M1 | No | None/Unknown |
| >=28 | Others | Female | T2 | N0 | M0 | No | Yes |
| >=28 | White | Male | T1 | N0 | M0 | Yes | None/Unknown |
| >=28 | White | Male | T2 | N0 | M0 | Yes | None/Unknown |
| <18 | White | Male | T1 | N0 | M0 | No | Yes |
| >=28 | White | Male | T1 | N0 | M0 | Yes | Yes |
| <18 | White | Female | T2 | N0 | M0 | Yes | None/Unknown |
| <18 | White | Female | T2 | N1 | M1 | No | Yes |
| <18 | White | Male | T3 | N0 | M1 | No | Yes |
| <18 | White | Female | T1 | N0 | M0 | No | Yes |
| >=28 | White | Female | T1 | N0 | M0 | Yes | Yes |
| <18 | White | Female | T1 | N0 | M0 | Yes | None/Unknown |
| <18 | White | Female | T2 | N0 | M0 | No | Yes |
| >=28 | White | Female | T1 | N0 | M0 | Yes | None/Unknown |
| >=28 | White | Female | T3 | N0 | M1 | Yes | None/Unknown |
| >=28 | White | Female | T1 | N0 | M1 | No | None/Unknown |
| <18 | White | Female | T2 | N0 | M1 | No | Yes |
| <18 | White | Female | T2 | N0 | M1 | No | None/Unknown |
| >=28 | White | Female | T1 | N0 | M0 | Yes | None/Unknown |
| >=28 | White | Female | T3 | N0 | M0 | No | None/Unknown |
| >=28 | Black | Female | T1 | N0 | M0 | Yes | None/Unknown |
| <18 | White | Male | T2 | N0 | M0 | No | Yes |

| | | | | | | | |
|---|---|---|---|---|---|---|---|
| >=28 | White | Male | T2 | N0 | M0 | No | Yes |
| >=28 | White | Male | T2 | N0 | M0 | Yes | None/Unknown |
| >=28 | White | Female | T2 | N0 | M0 | Yes | Yes |
| >=28 | White | Male | T2 | N1 | M1 | No | None/Unknown |
| <18 | White | Female | T1 | N0 | M0 | Yes | None/Unknown |
| <18 | White | Male | T1 | N1 | M1 | No | Yes |
| >=28 | White | Male | T1 | N0 | M0 | Yes | Yes |
| <18 | White | Female | T1 | N0 | M0 | No | Yes |
| <18 | White | Female | T2 | N0 | M0 | No | Yes |
| >=28 | White | Female | T2 | N0 | M0 | No | Yes |
| >=28 | White | Female | T1 | N0 | M0 | No | Yes |
| >=28 | White | Female | T1 | N0 | M0 | Yes | None/Unknown |
| >=28 | White | Female | T3 | N0 | M1 | No | Yes |
| >=28 | White | Female | T2 | N0 | M0 | Yes | None/Unknown |
| >=28 | White | Male | T3 | N0 | M0 | Yes | Yes |
| >=28 | White | Male | T2 | N0 | M1 | No | None/Unknown |
| >=28 | White | Female | T1 | N0 | M0 | Yes | None/Unknown |
| <18 | White | Female | T1 | N0 | M0 | Yes | None/Unknown |
| <18 | White | Female | T1 | N0 | M0 | Yes | None/Unknown |
| >=28 | Others | Female | T2 | N0 | M0 | Yes | None/Unknown |
| >=28 | White | Female | T3 | N0 | M0 | Yes | None/Unknown |
| >=28 | White | Female | T2 | N0 | M1 | No | None/Unknown |
| >=28 | White | Male | T1 | N0 | M0 | Yes | Yes |
| <18 | White | Female | T1 | N0 | M0 | Yes | Yes |
| <18 | White | Female | T1 | N0 | M0 | Yes | None/Unknown |
| >=28 | White | Female | T2 | N0 | M0 | Yes | None/Unknown |
| <18 | White | Female | T3 | N0 | M1 | Yes | Yes |
| >=28 | White | Female | T3 | N1 | M1 | No | None/Unknown |
| <18 | White | Female | T2 | N0 | M0 | Yes | None/Unknown |
| <18 | White | Male | T3 | N0 | M1 | No | Yes |
| >=28 | White | Female | T2 | N0 | M0 | Yes | None/Unknown |
| <18 | White | Female | T1 | N0 | M0 | Yes | None/Unknown |
| <18 | White | Female | T1 | N0 | M0 | Yes | None/Unknown |
| >=28 | White | Female | T1 | N0 | M0 | Yes | None/Unknown |
| >=28 | White | Male | T1 | N0 | M0 | Yes | None/Unknown |
| >=28 | White | Female | T2 | N0 | M0 | No | Yes |
| >=28 | White | Male | T2 | N0 | M0 | No | None/Unknown |
| >=28 | White | Male | T1 | N0 | M0 | No | None/Unknown |
| >=28 | White | Female | T2 | N0 | M0 | No | None/Unknown |
| >=28 | White | Female | T1 | N0 | M0 | Yes | None/Unknown |
| >=28 | White | Female | T1 | N0 | M0 | Yes | None/Unknown |
| <18 | White | Male | T3 | N0 | M1 | No | Yes |
| >=28 | Others | Male | T1 | N0 | M0 | No | Yes |
| >=28 | White | Female | T1 | N0 | M0 | Yes | None/Unknown |
| >=28 | White | Male | T1 | N0 | M0 | Yes | None/Unknown |
| >=28 | White | Male | T1 | N0 | M1 | No | Yes |
| <18 | White | Female | T1 | N0 | M0 | No | Yes |
| >=28 | White | Female | T1 | N0 | M0 | No | None/Unknown |
| <18 | White | Female | T1 | N0 | M0 | No | Yes |
| <18 | White | Female | T1 | N0 | M1 | No | Yes |
| >=28 | White | Male | T1 | N0 | M0 | No | None/Unknown |
| >=28 | White | Female | T2 | N0 | M0 | Yes | None/Unknown |
| >=28 | White | Female | T1 | N0 | M1 | Yes | None/Unknown |
| <18 | White | Male | T2 | N0 | M0 | No | Yes |
| <18 | White | Male | T2 | N0 | M1 | No | Yes |
| >=28 | White | Female | T1 | N1 | M0 | Yes | None/Unknown |
| >=28 | White | Female | T2 | N0 | M1 | No | None/Unknown |
| <18 | White | Female | T2 | N0 | M1 | No | Yes |

| | | | | | | | |
|---|---|---|---|---|---|---|---|
| <18 | White | Male | T3 | N0 | M0 | No | Yes |
| >=28 | White | Female | T2 | N0 | M0 | Yes | None/Unknown |
| <18 | White | Female | T1 | N0 | M0 | Yes | None/Unknown |
| >=28 | White | Female | T1 | N0 | M0 | Yes | Yes |
| <18 | Black | Male | T1 | N0 | M0 | No | Yes |
| >=28 | White | Female | T1 | N0 | M0 | Yes | None/Unknown |
| >=28 | Others | Male | T2 | N0 | M0 | Yes | Yes |
| >=28 | White | Male | T1 | N0 | M0 | No | None/Unknown |
| <18 | White | Male | T1 | N0 | M0 | No | Yes |
| >=28 | White | Female | T2 | N0 | M1 | No | Yes |
| >=28 | White | Male | T2 | N0 | M0 | Yes | Yes |
| >=28 | White | Male | T1 | N0 | M0 | Yes | None/Unknown |
| >=28 | White | Male | T2 | N0 | M0 | No | Yes |
| >=28 | White | Male | T1 | N0 | M0 | Yes | Yes |
| >=28 | White | Female | T2 | N0 | M0 | Yes | None/Unknown |
| <18 | White | Female | T1 | N0 | M0 | No | None/Unknown |
| >=28 | White | Male | T1 | N0 | M0 | Yes | Yes |
| >=28 | White | Female | T1 | N0 | M0 | No | Yes |
| >=28 | White | Male | T1 | N1 | M1 | Yes | Yes |
| >=28 | White | Female | T2 | N0 | M1 | Yes | None/Unknown |
| >=28 | White | Female | T1 | N0 | M0 | Yes | None/Unknown |
| >=28 | White | Female | T2 | N0 | M1 | Yes | None/Unknown |
| <18 | White | Female | T1 | N0 | M0 | No | None/Unknown |
| >=28 | White | Female | T2 | N0 | M1 | No | None/Unknown |
| >=28 | White | Male | T2 | N0 | M1 | No | None/Unknown |
| >=28 | White | Female | T1 | N0 | M0 | Yes | None/Unknown |
| <18 | White | Female | T1 | N0 | M0 | No | Yes |
| >=28 | Black | Female | T3 | N0 | M1 | No | None/Unknown |
| >=28 | White | Male | T2 | N0 | M0 | No | Yes |
| <18 | White | Female | T2 | N0 | M1 | No | Yes |
| >=28 | White | Male | T2 | N0 | M0 | Yes | None/Unknown |
| >=28 | White | Female | T3 | N1 | M1 | No | Yes |
| <18 | White | Female | T2 | N0 | M1 | No | None/Unknown |
| >=28 | White | Male | T2 | N0 | M0 | Yes | None/Unknown |
| <18 | White | Male | T2 | N0 | M0 | No | Yes |
| >=28 | White | Female | T1 | N0 | M1 | Yes | None/Unknown |
| >=28 | White | Female | T1 | N0 | M0 | Yes | Yes |
| >=28 | White | Female | T2 | N0 | M0 | Yes | None/Unknown |
| >=28 | White | Female | T1 | N0 | M0 | Yes | None/Unknown |
| >=28 | White | Male | T1 | N0 | M0 | Yes | None/Unknown |
| <18 | White | Female | T1 | N0 | M1 | No | Yes |
| >=28 | White | Female | T2 | N0 | M0 | No | Yes |
| <18 | White | Male | T1 | N0 | M0 | No | Yes |
| >=28 | White | Male | T1 | N0 | M0 | Yes | None/Unknown |
| >=28 | White | Female | T2 | N1 | M1 | No | Yes |
| <18 | White | Female | T1 | N0 | M0 | No | Yes |
| >=28 | White | Female | T1 | N0 | M0 | Yes | None/Unknown |
| >=28 | White | Female | T3 | N0 | M1 | No | Yes |
| >=28 | White | Female | T1 | N0 | M0 | No | None/Unknown |
| <18 | Black | Female | T2 | N0 | M0 | No | Yes |
| >=28 | White | Female | T1 | N0 | M0 | Yes | Yes |
| >=28 | White | Male | T1 | N0 | M0 | Yes | None/Unknown |
| >=28 | White | Female | T2 | N0 | M0 | No | None/Unknown |
| >=28 | White | Female | T2 | N0 | M0 | No | Yes |
| >=28 | White | Female | T2 | N1 | M1 | No | Yes |
| >=28 | White | Female | T1 | N0 | M0 | No | None/Unknown |
| >=28 | White | Female | T1 | N0 | M0 | Yes | None/Unknown |
| >=28 | White | Female | T2 | N0 | M0 | Yes | None/Unknown |

| | | | | | | | |
|---|---|---|---|---|---|---|---|
| <18 | White | Female | T1 | N0 | M1 | No | Yes |
| >=28 | White | Female | T1 | N0 | M0 | No | None/Unknown |
| <18 | White | Female | T2 | N0 | M0 | No | Yes |
| >=28 | White | Female | T1 | N0 | M0 | Yes | Yes |
| >=28 | White | Male | T1 | N0 | M0 | Yes | None/Unknown |
| >=28 | White | Male | T1 | N0 | M1 | Yes | Yes |
| <18 | Black | Female | T1 | N0 | M0 | Yes | None/Unknown |
| >=28 | White | Female | T2 | N0 | M0 | Yes | None/Unknown |
| >=28 | White | Male | T2 | N0 | M1 | No | Yes |
| >=28 | White | Male | T1 | N0 | M0 | Yes | None/Unknown |
| >=28 | White | Female | T2 | N0 | M0 | No | Yes |

| Chemotherapy | Primary.site | futime | fustat | distance | weights | subclass |
|---|---|---|---|---|---|---|
| Yes | Axial | 72 | Dead | 0.504247 | 1 | 12 |
| Yes | Extremity | 34 | Dead | 0.436654 | 1 | 286 |
| Yes | Axial | 166 | Alive | 0.504247 | 1 | 89 |
| Yes | Extremity | 7 | Dead | 0.392588 | 1 | 132 |
| Yes | Extremity | 180 | Alive | 0.392588 | 1 | 132 |
| Yes | Axial | 88 | Dead | 0.436654 | 1 | 63 |
| Yes | Axial | 34 | Dead | 0.407462 | 1 | 3 |
| Yes | Axial | 13 | Dead | 0.436654 | 1 | 286 |
| Yes | Axial | 166 | Alive | 0.392588 | 1 | 291 |
| Yes | Extremity | 50 | Dead | 0.392588 | 1 | 291 |
| Yes | Extremity | 163 | Alive | 0.407462 | 1 | 3 |
| Yes | Extremity | 160 | Alive | 0.392588 | 1 | 44 |
| Yes | Extremity | 17 | Dead | 0.504247 | 1 | 12 |
| Yes | Extremity | 77 | Alive | 0.33 | 1 | 17 |
| Yes | Extremity | 91 | Dead | 0.392588 | 1 | 76 |
| Yes | Extremity | 78 | Dead | 0.504247 | 1 | 97 |
| Yes | Axial | 61 | Alive | 0.33 | 1 | 17 |
| Yes | Extremity | 132 | Alive | 0.504247 | 1 | 166 |
| Yes | Axial | 130 | Alive | 0.407462 | 1 | 287 |
| Yes | Axial | 126 | Alive | 0.392588 | 1 | 44 |
| Yes | Axial | 123 | Alive | 0.33 | 1 | 47 |
| Yes | Axial | 121 | Alive | 0.407462 | 1 | 31 |
| Yes | Extremity | 110 | Alive | 0.33 | 1 | 56 |
| Yes | Extremity | 113 | Alive | 0.436654 | 1 | 63 |
| Yes | Axial | 123 | Alive | 0.33 | 1 | 47 |
| Yes | Extremity | 49 | Dead | 0.33 | 1 | 56 |
| Yes | Axial | 24 | Dead | 0.392588 | 1 | 76 |
| Yes | Axial | 85 | Dead | 0.392588 | 1 | 80 |
| Yes | Axial | 102 | Alive | 0.504247 | 1 | 191 |
| Yes | Axial | 47 | Dead | 0.504247 | 1 | 89 |
| Yes | Axial | 9 | Dead | 0.343844 | 1 | 110 |
| Yes | Extremity | 17 | Dead | 0.504247 | 1 | 97 |
| Yes | Axial | 98 | Alive | 0.33 | 1 | 105 |
| Yes | Axial | 97 | Alive | 0.343844 | 1 | 118 |
| Yes | Extremity | 74 | Alive | 0.504247 | 1 | 224 |
| Yes | Extremity | 81 | Alive | 0.504247 | 1 | 289 |
| Yes | Extremity | 78 | Alive | 0.407462 | 1 | 33 |
| Yes | Extremity | 37 | Dead | 0.392588 | 1 | 124 |
| Yes | Extremity | 65 | Dead | 0.343844 | 1 | 151 |
| Yes | Extremity | 45 | Dead | 0.392588 | 1 | 136 |
| Yes | Axial | 55 | Alive | 0.504247 | 1 | 292 |
| Yes | Axial | 13 | Dead | 0.504247 | 1 | 294 |
| Yes | Axial | 67 | Dead | 0.773802 | 1 | 62 |
| Yes | Axial | 176 | Alive | 0.33 | 1 | 105 |
| Yes | Axial | 172 | Alive | 0.33 | 1 | 161 |
| Yes | Axial | 161 | Alive | 0.504247 | 1 | 166 |
| Yes | Axial | 146 | Alive | 0.392588 | 1 | 173 |
| Yes | Extremity | 146 | Alive | 0.392588 | 1 | 80 |
| Yes | Extremity | 147 | Alive | 0.392588 | 1 | 124 |
| Yes | Axial | 137 | Alive | 0.33 | 1 | 187 |
| Yes | Extremity | 14 | Dead | 0.504247 | 1 | 191 |
| Yes | Extremity | 96 | Dead | 0.33 | 1 | 161 |
| Yes | Extremity | 13 | Alive | 0.33 | 1 | 203 |
| Yes | Axial | 17 | Dead | 0.392588 | 1 | 207 |
| Yes | Extremity | 126 | Alive | 0.504247 | 1 | 7 |
| Yes | Extremity | 117 | Alive | 0.392588 | 1 | 216 |
| Yes | Axial | 111 | Alive | 0.33 | 1 | 187 |

| | | | | | | |
|---|---|---|---|---|---|---|
| Yes | Axial | 13 | Dead | 0.504247 | 1 | 224 |
| Yes | Axial | 102 | Alive | 0.392588 | 1 | 228 |
| Yes | Extremity | 8 | Dead | 0.436654 | 1 | 283 |
| Yes | Extremity | 73 | Dead | 0.33 | 1 | 203 |
| Yes | Axial | 19 | Dead | 0.33 | 1 | 241 |
| Yes | Axial | 18 | Dead | 0.504247 | 1 | 10 |
| Yes | Axial | 83 | Alive | 0.392588 | 1 | 136 |
| Yes | Extremity | 76 | Alive | 0.392588 | 1 | 255 |
| Yes | Axial | 82 | Alive | 0.436654 | 1 | 290 |
| Yes | Extremity | 69 | Alive | 0.33 | 1 | 241 |
| Yes | Axial | 71 | Alive | 0.392588 | 1 | 265 |
| Yes | Axial | 64 | Alive | 0.392588 | 1 | 173 |
| Yes | Extremity | 60 | Alive | 0.684919 | 1 | 276 |
| Yes | Extremity | 62 | Alive | 0.33 | 1 | 8 |
| Yes | Extremity | 50 | Alive | 0.392588 | 1 | 280 |
| Yes | Extremity | 51 | Alive | 0.504247 | 1 | 11 |
| Yes | Axial | 49 | Alive | 0.436654 | 1 | 283 |
| Yes | Axial | 59 | Alive | 0.33 | 1 | 9 |
| Yes | Extremity | 51 | Alive | 0.33 | 1 | 13 |
| Yes | Extremity | 16 | Alive | 0.392588 | 1 | 207 |
| Yes | Axial | 186 | Alive | 0.304089 | 1 | 66 |
| Yes | Axial | 142 | Alive | 0.304089 | 1 | 67 |
| Yes | Extremity | 111 | Alive | 0.249806 | 1 | 288 |
| Yes | Extremity | 28 | Dead | 0.407462 | 1 | 287 |
| Yes | Extremity | 37 | Dead | 0.249806 | 1 | 288 |
| Yes | Axial | 86 | Alive | 0.504247 | 1 | 18 |
| Yes | Extremity | 74 | Alive | 0.343844 | 1 | 215 |
| Yes | Extremity | 10 | Dead | 0.304089 | 1 | 68 |
| Yes | Axial | 8 | Dead | 0.504247 | 1 | 289 |
| Yes | Axial | 189 | Alive | 0.436654 | 1 | 290 |
| Yes | Extremity | 178 | Alive | 0.33 | 1 | 16 |
| Yes | Extremity | 172 | Alive | 0.392588 | 1 | 216 |
| Yes | Axial | 174 | Alive | 0.504247 | 1 | 292 |
| Yes | Axial | 10 | Dead | 0.504247 | 1 | 23 |
| Yes | Extremity | 169 | Alive | 0.392588 | 1 | 228 |
| Yes | Axial | 173 | Alive | 0.33 | 1 | 25 |
| Yes | Extremity | 167 | Alive | 0.33 | 1 | 26 |
| Yes | Axial | 157 | Alive | 0.392588 | 1 | 293 |
| Yes | Extremity | 152 | Alive | 0.33 | 1 | 37 |
| Yes | Extremity | 143 | Alive | 0.504247 | 1 | 294 |
| None/Unknown | Extremity | 140 | Alive | 0.734652 | 1 | 295 |
| Yes | Extremity | 125 | Alive | 0.392588 | 1 | 255 |
| Yes | Axial | 109 | Dead | 0.392588 | 1 | 265 |
| Yes | Axial | 126 | Alive | 0.504247 | 1 | 29 |
| Yes | Axial | 15 | Dead | 0.504247 | 1 | 35 |
| Yes | Extremity | 115 | Alive | 0.392588 | 1 | 280 |
| Yes | Extremity | 112 | Alive | 0.504247 | 1 | 36 |
| Yes | Axial | 104 | Alive | 0.392588 | 1 | 293 |
| Yes | Axial | 119 | Alive | 0.436654 | 1 | 1 |
| Yes | Axial | 98 | Alive | 0.436654 | 1 | 2 |
| Yes | Extremity | 96 | Alive | 0.33 | 1 | 43 |
| Yes | Extremity | 6 | Dead | 0.392588 | 1 | 4 |
| Yes | Extremity | 35 | Dead | 0.392588 | 1 | 5 |
| Yes | Extremity | 73 | Alive | 0.392588 | 1 | 6 |
| Yes | Extremity | 16 | Dead | 0.504247 | 1 | 7 |
| Yes | Axial | 54 | Dead | 0.504247 | 1 | 38 |
| Yes | Extremity | 65 | Alive | 0.33 | 1 | 8 |
| Yes | Extremity | 81 | Alive | 0.33 | 1 | 53 |

| | | | | | | |
|---|---|---|---|---|---|---|
| Yes | Extremity | 60 | Alive | 0.392588 | 1 | 4 |
| Yes | Axial | 53 | Alive | 0.392588 | 1 | 5 |
| Yes | Axial | 183 | Alive | 0.249806 | 1 | 95 |
| Yes | Axial | 117 | Dead | 0.33 | 1 | 9 |
| Yes | Axial | 25 | Dead | 0.504247 | 1 | 10 |
| Yes | Axial | 79 | Dead | 0.392588 | 1 | 6 |
| Yes | Axial | 123 | Alive | 0.504247 | 1 | 39 |
| Yes | Axial | 56 | Dead | 0.504247 | 1 | 11 |
| Yes | Axial | 38 | Dead | 0.33 | 1 | 57 |
| Yes | Extremity | 75 | Alive | 0.33 | 1 | 74 |
| Yes | Extremity | 68 | Alive | 0.504247 | 1 | 42 |
| Yes | Axial | 31 | Dead | 0.504247 | 1 | 48 |
| Yes | Axial | 3 | Dead | 0.304089 | 1 | 154 |
| Yes | Axial | 13 | Dead | 0.33 | 1 | 81 |
| Yes | Axial | 98 | Dead | 0.33 | 1 | 13 |
| Yes | Axial | 177 | Alive | 0.304089 | 1 | 198 |
| Yes | Extremity | 39 | Dead | 0.392588 | 1 | 14 |
| Yes | Extremity | 170 | Alive | 0.392588 | 1 | 15 |
| Yes | Axial | 167 | Alive | 0.504247 | 1 | 49 |
| Yes | Axial | 28 | Dead | 0.249806 | 1 | 125 |
| Yes | Axial | 160 | Alive | 0.504247 | 1 | 50 |
| Yes | Axial | 152 | Alive | 0.33 | 1 | 16 |
| Yes | Axial | 151 | Alive | 0.436654 | 1 | 1 |
| Yes | Axial | 150 | Alive | 0.504247 | 1 | 59 |
| Yes | Axial | 14 | Dead | 0.504247 | 1 | 18 |
| Yes | Axial | 137 | Alive | 0.392588 | 1 | 14 |
| Yes | Axial | 57 | Dead | 0.392588 | 1 | 19 |
| Yes | Axial | 132 | Alive | 0.392588 | 1 | 20 |
| Yes | Extremity | 132 | Alive | 0.33 | 1 | 84 |
| Yes | Extremity | 31 | Dead | 0.436654 | 1 | 21 |
| Yes | Axial | 127 | Alive | 0.504247 | 1 | 61 |
| Yes | Axial | 37 | Dead | 0.436654 | 1 | 22 |
| Yes | Extremity | 16 | Alive | 0.504247 | 1 | 23 |
| Yes | Extremity | 123 | Alive | 0.33 | 1 | 86 |
| Yes | Axial | 123 | Alive | 0.392588 | 1 | 15 |
| None/Unknown | Extremity | 120 | Alive | 0.734652 | 1 | 24 |
| Yes | Extremity | 116 | Alive | 0.33 | 1 | 25 |
| Yes | Axial | 111 | Alive | 0.392588 | 1 | 19 |
| Yes | Extremity | 102 | Dead | 0.33 | 1 | 26 |
| Yes | Extremity | 104 | Alive | 0.392588 | 1 | 27 |
| Yes | Extremity | 102 | Alive | 0.392588 | 1 | 20 |
| Yes | Extremity | 44 | Dead | 0.392588 | 1 | 27 |
| Yes | Extremity | 39 | Dead | 0.392588 | 1 | 28 |
| Yes | Axial | 90 | Alive | 0.504247 | 1 | 29 |
| Yes | Axial | 25 | Dead | 0.392588 | 1 | 30 |
| Yes | Axial | 33 | Dead | 0.33 | 1 | 88 |
| Yes | Axial | 15 | Dead | 0.407462 | 1 | 31 |
| Yes | Axial | 20 | Dead | 0.392588 | 1 | 32 |
| Yes | Extremity | 81 | Alive | 0.33 | 1 | 98 |
| Yes | Axial | 80 | Alive | 0.392588 | 1 | 28 |
| Yes | Extremity | 70 | Alive | 0.392588 | 1 | 30 |
| Yes | Axial | 75 | Alive | 0.407462 | 1 | 33 |
| Yes | Axial | 70 | Alive | 0.436654 | 1 | 2 |
| Yes | Extremity | 66 | Alive | 0.33 | 1 | 99 |
| Yes | Extremity | 44 | Alive | 0.392588 | 1 | 32 |
| Yes | Extremity | 29 | Dead | 0.392588 | 1 | 34 |
| Yes | Axial | 61 | Alive | 0.436654 | 1 | 21 |
| Yes | Axial | 61 | Alive | 0.392588 | 1 | 34 |

| | | | | | | |
|---|---|---|---|---|---|---|
| Yes | Axial | 27 | Dead | 0.504247 | 1 | 35 |
| Yes | Axial | 25 | Dead | 0.504247 | 1 | 36 |
| Yes | Extremity | 56 | Alive | 0.392588 | 1 | 40 |
| Yes | Extremity | 54 | Alive | 0.392588 | 1 | 41 |
| Yes | Axial | 58 | Dead | 0.392588 | 1 | 46 |
| Yes | Extremity | 43 | Dead | 0.33 | 1 | 37 |
| Yes | Axial | 18 | Dead | 0.504247 | 1 | 38 |
| Yes | Extremity | 112 | Dead | 0.504247 | 1 | 39 |
| Yes | Extremity | 12 | Dead | 0.392588 | 1 | 40 |
| Yes | Axial | 33 | Dead | 0.504247 | 1 | 69 |
| Yes | Extremity | 128 | Dead | 0.392588 | 1 | 41 |
| Yes | Axial | 133 | Alive | 0.392588 | 1 | 51 |
| Yes | Extremity | 81 | Alive | 0.392588 | 1 | 52 |
| Yes | Axial | 120 | Alive | 0.436654 | 1 | 22 |
| Yes | Axial | 120 | Alive | 0.504247 | 1 | 42 |
| Yes | Extremity | 19 | Dead | 0.392588 | 1 | 54 |
| Yes | Axial | 11 | Dead | 0.436654 | 1 | 45 |
| Yes | Extremity | 116 | Alive | 0.33 | 1 | 116 |
| Yes | Axial | 114 | Alive | 0.33 | 1 | 117 |
| Yes | Axial | 118 | Alive | 0.33 | 1 | 143 |
| Yes | Extremity | 107 | Alive | 0.392588 | 1 | 58 |
| Yes | Axial | 105 | Alive | 0.33 | 1 | 150 |
| Yes | Axial | 35 | Dead | 0.33 | 1 | 43 |
| Yes | Axial | 142 | Alive | 0.33 | 1 | 163 |
| Yes | Extremity | 140 | Alive | 0.392588 | 1 | 60 |
| Yes | Axial | 15 | Dead | 0.436654 | 1 | 45 |
| Yes | Extremity | 92 | Alive | 0.392588 | 1 | 64 |
| Yes | Extremity | 94 | Alive | 0.392588 | 1 | 72 |
| Yes | Extremity | 64 | Alive | 0.392588 | 1 | 73 |
| Yes | Axial | 84 | Alive | 0.392588 | 1 | 46 |
| Yes | Axial | 85 | Alive | 0.504247 | 1 | 70 |
| Yes | Extremity | 94 | Alive | 0.392588 | 1 | 75 |
| Yes | Extremity | 54 | Dead | 0.504247 | 1 | 79 |
| Yes | Axial | 12 | Dead | 0.504247 | 1 | 48 |
| Yes | Axial | 79 | Alive | 0.504247 | 1 | 87 |
| Yes | Axial | 7 | Dead | 0.436654 | 1 | 77 |
| Yes | Axial | 15 | Dead | 0.504247 | 1 | 49 |
| Yes | Extremity | 53 | Dead | 0.504247 | 1 | 96 |
| Yes | Axial | 69 | Alive | 0.392588 | 1 | 82 |
| Yes | Extremity | 32 | Dead | 0.504247 | 1 | 50 |
| Yes | Extremity | 64 | Alive | 0.392588 | 1 | 51 |
| Yes | Axial | 71 | Alive | 0.33 | 1 | 168 |
| Yes | Extremity | 15 | Dead | 0.392588 | 1 | 52 |
| Yes | Extremity | 58 | Alive | 0.33 | 1 | 172 |
| Yes | Axial | 57 | Alive | 0.33 | 1 | 53 |
| Yes | Axial | 51 | Alive | 0.33 | 1 | 174 |
| Yes | Extremity | 54 | Alive | 0.392588 | 1 | 54 |
| Yes | Extremity | 51 | Alive | 0.504247 | 1 | 101 |
| Yes | Axial | 178 | Alive | 0.504247 | 1 | 104 |
| Yes | Extremity | 16 | Dead | 0.436654 | 1 | 78 |
| Yes | Axial | 93 | Dead | 0.684919 | 1 | 55 |
| Yes | Axial | 31 | Dead | 0.33 | 1 | 57 |
| Yes | Extremity | 20 | Dead | 0.392588 | 1 | 83 |
| Yes | Extremity | 106 | Alive | 0.436654 | 1 | 102 |
| Yes | Axial | 33 | Dead | 0.392588 | 1 | 58 |
| Yes | Axial | 36 | Dead | 0.504247 | 1 | 59 |
| Yes | Extremity | 86 | Alive | 0.33 | 1 | 175 |
| Yes | Axial | 102 | Alive | 0.392588 | 1 | 60 |

| | | | | | | |
|---|---|---|---|---|---|---|
| Yes | Axial | 4 | Dead | 0.504247 | 1 | 61 |
| Yes | Axial | 82 | Alive | 0.773802 | 1 | 62 |
| Yes | Axial | 74 | Alive | 0.392588 | 1 | 64 |
| Yes | Extremity | 72 | Alive | 0.33 | 1 | 184 |
| Yes | Axial | 48 | Dead | 0.33 | 1 | 188 |
| Yes | Extremity | 63 | Alive | 0.436654 | 1 | 106 |
| Yes | Axial | 54 | Alive | 0.407462 | 1 | 65 |
| Yes | Extremity | 58 | Alive | 0.249806 | 1 | 231 |
| Yes | Axial | 48 | Alive | 0.504247 | 1 | 108 |
| Yes | Extremity | 25 | Alive | 0.392588 | 1 | 85 |
| Yes | Extremity | 47 | Dead | 0.304089 | 1 | 66 |
| Yes | Extremity | 93 | Alive | 0.304089 | 1 | 67 |
| Yes | Extremity | 68 | Alive | 0.304089 | 1 | 68 |
| Yes | Extremity | 187 | Alive | 0.392588 | 1 | 90 |
| Yes | Axial | 15 | Dead | 0.504247 | 1 | 69 |
| Yes | Axial | 162 | Alive | 0.504247 | 1 | 70 |
| Yes | Axial | 43 | Dead | 0.436654 | 1 | 107 |
| Yes | Extremity | 16 | Dead | 0.684919 | 1 | 71 |
| Yes | Extremity | 37 | Alive | 0.392588 | 1 | 72 |
| Yes | Extremity | 154 | Alive | 0.436654 | 1 | 111 |
| Yes | Extremity | 148 | Alive | 0.392588 | 1 | 91 |
| Yes | Extremity | 147 | Alive | 0.392588 | 1 | 73 |
| Yes | Axial | 141 | Alive | 0.33 | 1 | 195 |
| Yes | Extremity | 140 | Alive | 0.504247 | 1 | 112 |
| Yes | Axial | 135 | Alive | 0.33 | 1 | 74 |
| Yes | Axial | 129 | Alive | 0.684919 | 1 | 276 |
| Yes | Axial | 26 | Dead | 0.392588 | 1 | 92 |
| Yes | Axial | 64 | Dead | 0.392588 | 1 | 75 |
| Yes | Extremity | 42 | Dead | 0.504247 | 1 | 120 |
| Yes | Extremity | 11 | Dead | 0.436654 | 1 | 77 |
| Yes | Axial | 100 | Alive | 0.436654 | 1 | 78 |
| Yes | Extremity | 96 | Alive | 0.392588 | 1 | 93 |
| Yes | Axial | 79 | Alive | 0.407462 | 1 | 65 |
| Yes | Extremity | 72 | Alive | 0.504247 | 1 | 121 |
| Yes | Axial | 31 | Dead | 0.504247 | 1 | 79 |
| Yes | Extremity | 62 | Alive | 0.392588 | 1 | 94 |
| Yes | Axial | 51 | Alive | 0.392588 | 1 | 103 |
| Yes | Extremity | 191 | Alive | 0.33 | 1 | 81 |
| Yes | Axial | 101 | Dead | 0.392588 | 1 | 82 |
| Yes | Extremity | 50 | Dead | 0.392588 | 1 | 83 |
| Yes | Axial | 10 | Dead | 0.504247 | 1 | 128 |
| Yes | Extremity | 181 | Alive | 0.33 | 1 | 201 |
| Yes | Extremity | 182 | Alive | 0.33 | 1 | 208 |
| Yes | Axial | 181 | Alive | 0.504247 | 1 | 129 |
| Yes | Extremity | 178 | Alive | 0.33 | 1 | 84 |
| Yes | Axial | 3 | Dead | 0.392588 | 1 | 109 |
| Yes | Extremity | 176 | Alive | 0.392588 | 1 | 85 |
| Yes | Extremity | 170 | Alive | 0.33 | 1 | 86 |
| Yes | Axial | 170 | Alive | 0.33 | 1 | 218 |
| Yes | Extremity | 13 | Dead | 0.392588 | 1 | 113 |
| Yes | Axial | 54 | Dead | 0.504247 | 1 | 87 |
| Yes | Axial | 15 | Dead | 0.33 | 1 | 88 |
| Yes | Axial | 161 | Alive | 0.392588 | 1 | 114 |
| Yes | Extremity | 77 | Dead | 0.392588 | 1 | 115 |
| Yes | Extremity | 161 | Alive | 0.392588 | 1 | 119 |
| Yes | Extremity | 153 | Alive | 0.392588 | 1 | 122 |
| Yes | Axial | 20 | Dead | 0.392588 | 1 | 126 |
| Yes | Axial | 86 | Alive | 0.407462 | 1 | 137 |

| | | | | | | |
|---|---|---|---|---|---|---|
| Yes | Extremity | 154 | Alive | 0.392588 | 1 | 90 |
| Yes | Axial | 47 | Dead | 0.504247 | 1 | 133 |
| Yes | Extremity | 150 | Alive | 0.392588 | 1 | 91 |
| Yes | Extremity | 12 | Dead | 0.392588 | 1 | 92 |
| Yes | Extremity | 11 | Dead | 0.392588 | 1 | 93 |
| Yes | Extremity | 43 | Dead | 0.33 | 1 | 234 |
| Yes | Extremity | 135 | Alive | 0.392588 | 1 | 127 |
| Yes | Extremity | 128 | Alive | 0.33 | 1 | 235 |
| Yes | Axial | 134 | Alive | 0.33 | 1 | 237 |
| Yes | Axial | 40 | Alive | 0.392588 | 1 | 94 |
| Yes | Axial | 127 | Alive | 0.436654 | 1 | 123 |
| Yes | Axial | 123 | Alive | 0.504247 | 1 | 138 |
| Yes | Axial | 30 | Dead | 0.249806 | 1 | 95 |
| Yes | Extremity | 119 | Alive | 0.504247 | 1 | 139 |
| Yes | Extremity | 121 | Alive | 0.392588 | 1 | 130 |
| Yes | Extremity | 119 | Alive | 0.33 | 1 | 239 |
| Yes | Axial | 24 | Dead | 0.504247 | 1 | 142 |
| Yes | Extremity | 70 | Dead | 0.504247 | 1 | 96 |
| Yes | Extremity | 116 | Alive | 0.33 | 1 | 242 |
| Yes | Extremity | 116 | Alive | 0.33 | 1 | 98 |
| Yes | Axial | 110 | Alive | 0.392588 | 1 | 131 |
| Yes | Extremity | 107 | Alive | 0.33 | 1 | 99 |
| Yes | Extremity | 102 | Alive | 0.722754 | 1 | 100 |
| Yes | Extremity | 27 | Dead | 0.504247 | 1 | 101 |
| Yes | Extremity | 102 | Alive | 0.392588 | 1 | 135 |
| Yes | Axial | 20 | Dead | 0.436654 | 1 | 102 |
| Yes | Axial | 58 | Alive | 0.392588 | 1 | 103 |
| Yes | Axial | 95 | Alive | 0.504247 | 1 | 104 |
| Yes | Axial | 12 | Dead | 0.436654 | 1 | 106 |
| Yes | Extremity | 6 | Alive | 0.33 | 1 | 251 |
| Yes | Axial | 78 | Alive | 0.436654 | 1 | 134 |
| Yes | Axial | 78 | Alive | 0.436654 | 1 | 107 |
| Yes | Axial | 19 | Dead | 0.504247 | 1 | 108 |
| Yes | Extremity | 10 | Dead | 0.33 | 1 | 253 |
| Yes | Axial | 42 | Dead | 0.392588 | 1 | 109 |
| Yes | Extremity | 71 | Alive | 0.33 | 1 | 259 |
| Yes | Extremity | 75 | Alive | 0.33 | 1 | 261 |
| Yes | Extremity | 67 | Alive | 0.684919 | 1 | 55 |
| Yes | Extremity | 64 | Alive | 0.392588 | 1 | 140 |
| Yes | Axial | 9 | Alive | 0.343844 | 1 | 110 |
| Yes | Axial | 21 | Dead | 0.436654 | 1 | 111 |
| Yes | Extremity | 55 | Alive | 0.392588 | 1 | 141 |
| Yes | Extremity | 43 | Dead | 0.392588 | 1 | 148 |
| Yes | Axial | 53 | Alive | 0.33 | 1 | 268 |
| Yes | Axial | 53 | Alive | 0.33 | 1 | 278 |
| Yes | Axial | 51 | Alive | 0.392588 | 1 | 153 |
| Yes | Axial | 18 | Dead | 0.504247 | 1 | 112 |
| Yes | Axial | 10 | Dead | 0.436654 | 1 | 144 |
| Yes | Axial | 13 | Dead | 0.392588 | 1 | 113 |
| Yes | Extremity | 55 | Alive | 0.392588 | 1 | 155 |
| Yes | Axial | 106 | Alive | 0.392588 | 1 | 114 |
| Yes | Axial | 183 | Alive | 0.504247 | 1 | 145 |
| Yes | Extremity | 191 | Alive | 0.392588 | 1 | 158 |
| Yes | Axial | 3 | Dead | 0.33 | 1 | 279 |
| Yes | Extremity | 182 | Alive | 0.33 | 1 | 284 |
| Yes | Extremity | 9 | Dead | 0.392588 | 1 | 164 |
| Yes | Extremity | 181 | Alive | 0.392588 | 1 | 169 |
| Yes | Axial | 97 | Dead | 0.504247 | 1 | 146 |

| | | | | | | |
|---|---|---|---|---|---|---|
| Yes | Extremity | 28 | Dead | 0.504247 | 1 | 152 |
| Yes | Extremity | 15 | Dead | 0.392588 | 1 | 176 |
| Yes | Axial | 119 | Dead | 0.504247 | 1 | 156 |
| Yes | Axial | 22 | Dead | 0.392588 | 1 | 115 |
| Yes | Extremity | 174 | Alive | 0.33 | 1 | 116 |
| Yes | Extremity | 2 | Alive | 0.504247 | 1 | 157 |
| Yes | Axial | 29 | Dead | 0.33 | 1 | 117 |
| Yes | Axial | 15 | Dead | 0.343844 | 1 | 118 |
| Yes | Extremity | 169 | Alive | 0.392588 | 1 | 119 |
| Yes | Extremity | 168 | Alive | 0.392588 | 1 | 177 |
| Yes | Extremity | 16 | Dead | 0.504247 | 1 | 120 |
| Yes | Axial | 10 | Alive | 0.504247 | 1 | 121 |
| Yes | Axial | 22 | Dead | 0.392588 | 1 | 122 |
| Yes | Axial | 6 | Dead | 0.436654 | 1 | 123 |
| Yes | Axial | 20 | Dead | 0.504247 | 1 | 159 |
| Yes | Extremity | 162 | Alive | 0.504247 | 1 | 160 |
| Yes | Extremity | 86 | Dead | 0.249806 | 1 | 125 |
| Yes | Extremity | 58 | Dead | 0.392588 | 1 | 126 |
| Yes | Extremity | 5 | Dead | 0.392588 | 1 | 127 |
| Yes | Axial | 12 | Dead | 0.392588 | 1 | 178 |
| Yes | Extremity | 12 | Dead | 0.504247 | 1 | 128 |
| None/Unknown | Extremity | 9 | Dead | 0.734652 | 1 | 295 |
| Yes | Extremity | 1 | Alive | 0.436654 | 1 | 147 |
| Yes | Axial | 29 | Dead | 0.392588 | 1 | 180 |
| Yes | Extremity | 151 | Alive | 0.392588 | 1 | 185 |
| Yes | Axial | 44 | Dead | 0.504247 | 1 | 167 |
| Yes | Axial | 8 | Dead | 0.504247 | 1 | 129 |
| Yes | Extremity | 29 | Dead | 0.392588 | 1 | 192 |
| Yes | Extremity | 12 | Alive | 0.392588 | 1 | 130 |
| Yes | Axial | 13 | Dead | 0.392588 | 1 | 194 |
| Yes | Extremity | 149 | Alive | 0.392588 | 1 | 131 |
| Yes | Extremity | 147 | Alive | 0.392588 | 1 | 197 |
| Yes | Extremity | 69 | Dead | 0.684919 | 1 | 71 |
| Yes | Axial | 146 | Alive | 0.392588 | 1 | 199 |
| Yes | Axial | 7 | Dead | 0.504247 | 1 | 133 |
| Yes | Axial | 35 | Dead | 0.504247 | 1 | 179 |
| Yes | Axial | 99 | Dead | 0.436654 | 1 | 134 |
| Yes | Axial | 26 | Dead | 0.392588 | 1 | 202 |
| Yes | Extremity | 135 | Alive | 0.392588 | 1 | 135 |
| Yes | Axial | 138 | Alive | 0.392588 | 1 | 205 |
| Yes | Axial | 34 | Dead | 0.436654 | 1 | 149 |
| Yes | Axial | 132 | Alive | 0.392588 | 1 | 206 |
| Yes | Extremity | 134 | Alive | 0.407462 | 1 | 137 |
| Yes | Extremity | 130 | Alive | 0.392588 | 1 | 213 |
| Yes | Extremity | 131 | Alive | 0.392588 | 1 | 214 |
| Yes | Axial | 128 | Alive | 0.504247 | 1 | 138 |
| Yes | Extremity | 36 | Dead | 0.504247 | 1 | 139 |
| Yes | Axial | 18 | Dead | 0.392588 | 1 | 140 |
| Yes | Axial | 9 | Dead | 0.392588 | 1 | 141 |
| Yes | Extremity | 126 | Alive | 0.392588 | 1 | 217 |
| Yes | Axial | 123 | Alive | 0.392588 | 1 | 222 |
| Yes | Axial | 10 | Dead | 0.504247 | 1 | 142 |
| Yes | Extremity | 123 | Alive | 0.33 | 1 | 143 |
| Yes | Axial | 9 | Dead | 0.436654 | 1 | 144 |
| Yes | Axial | 27 | Dead | 0.504247 | 1 | 145 |
| Yes | Axial | 117 | Alive | 0.504247 | 1 | 146 |
| Yes | Extremity | 119 | Alive | 0.392588 | 1 | 223 |
| Yes | Extremity | 20 | Dead | 0.436654 | 1 | 147 |

| | | | | | | |
|---|---|---|---|---|---|---|
| Yes | Extremity | 113 | Alive | 0.392588 | 1 | 148 |
| Yes | Axial | 119 | Alive | 0.436654 | 1 | 149 |
| Yes | Axial | 108 | Alive | 0.392588 | 1 | 225 |
| Yes | Axial | 106 | Alive | 0.33 | 1 | 150 |
| Yes | Axial | 94 | Dead | 0.343844 | 1 | 151 |
| Yes | Extremity | 11 | Dead | 0.504247 | 1 | 152 |
| Yes | Axial | 104 | Alive | 0.392588 | 1 | 227 |
| Yes | Extremity | 108 | Alive | 0.392588 | 1 | 153 |
| Yes | Extremity | 107 | Alive | 0.304089 | 1 | 154 |
| Yes | Extremity | 68 | Dead | 0.392588 | 1 | 155 |
| Yes | Axial | 8 | Dead | 0.504247 | 1 | 156 |
| Yes | Extremity | 12 | Dead | 0.504247 | 1 | 157 |
| Yes | Axial | 97 | Alive | 0.436654 | 1 | 162 |
| Yes | Axial | 29 | Dead | 0.392588 | 1 | 229 |
| Yes | Extremity | 34 | Dead | 0.392588 | 1 | 158 |
| Yes | Axial | 28 | Dead | 0.392588 | 1 | 230 |
| Yes | Axial | 22 | Dead | 0.504247 | 1 | 159 |
| Yes | Extremity | 69 | Dead | 0.392588 | 1 | 238 |
| Yes | Axial | 15 | Dead | 0.407462 | 1 | 165 |
| Yes | Axial | 100 | Alive | 0.504247 | 1 | 160 |
| Yes | Extremity | 95 | Alive | 0.504247 | 1 | 181 |
| Yes | Axial | 96 | Alive | 0.392588 | 1 | 243 |
| Yes | Axial | 94 | Alive | 0.392588 | 1 | 244 |
| Yes | Extremity | 95 | Alive | 0.436654 | 1 | 162 |
| Yes | Extremity | 94 | Alive | 0.504247 | 1 | 189 |
| Yes | Axial | 5 | Dead | 0.33 | 1 | 163 |
| Yes | Extremity | 58 | Dead | 0.407462 | 1 | 171 |
| Yes | Axial | 48 | Dead | 0.392588 | 1 | 164 |
| Yes | Axial | 95 | Alive | 0.392588 | 1 | 245 |
| Yes | Axial | 2 | Dead | 0.407462 | 1 | 165 |
| Yes | Axial | 48 | Alive | 0.504247 | 1 | 167 |
| Yes | Extremity | 90 | Alive | 0.504247 | 1 | 190 |
| Yes | Axial | 37 | Dead | 0.436654 | 1 | 170 |
| Yes | Axial | 84 | Alive | 0.504247 | 1 | 193 |
| Yes | Axial | 82 | Alive | 0.33 | 1 | 168 |
| Yes | Axial | 82 | Alive | 0.392588 | 1 | 169 |
| Yes | Axial | 56 | Dead | 0.436654 | 1 | 170 |
| Yes | Axial | 82 | Alive | 0.407462 | 1 | 171 |
| Yes | Axial | 45 | Alive | 0.33 | 1 | 172 |
| Yes | Extremity | 82 | Alive | 0.33 | 1 | 174 |
| Yes | Axial | 39 | Alive | 0.436654 | 1 | 183 |
| Yes | Axial | 78 | Alive | 0.33 | 1 | 175 |
| Yes | Extremity | 77 | Alive | 0.392588 | 1 | 248 |
| Yes | Axial | 76 | Alive | 0.504247 | 1 | 200 |
| Yes | Extremity | 76 | Alive | 0.436654 | 1 | 186 |
| Yes | Axial | 65 | Dead | 0.504247 | 1 | 204 |
| Yes | Axial | 41 | Alive | 0.392588 | 1 | 176 |
| Yes | Axial | 67 | Alive | 0.392588 | 1 | 254 |
| Yes | Axial | 69 | Alive | 0.504247 | 1 | 209 |
| Yes | Axial | 3 | Dead | 0.392588 | 1 | 177 |
| Yes | Axial | 30 | Dead | 0.392588 | 1 | 178 |
| Yes | Axial | 50 | Alive | 0.504247 | 1 | 179 |
| Yes | Extremity | 30 | Dead | 0.504247 | 1 | 212 |
| Yes | Axial | 63 | Alive | 0.504247 | 1 | 220 |
| Yes | Axial | 12 | Dead | 0.392588 | 1 | 180 |
| Yes | Extremity | 72 | Alive | 0.504247 | 1 | 181 |
| Yes | Extremity | 23 | Dead | 0.684919 | 1 | 182 |
| Yes | Axial | 31 | Dead | 0.436654 | 1 | 196 |

| | | | | | | |
|---|---|---|---|---|---|---|
| Yes | Extremity | 12 | Dead | 0.436654 | 1 | 183 |
| Yes | Extremity | 60 | Alive | 0.33 | 1 | 184 |
| Yes | Extremity | 25 | Dead | 0.392588 | 1 | 185 |
| Yes | Extremity | 4 | Dead | 0.436654 | 1 | 186 |
| Yes | Extremity | 59 | Alive | 0.392588 | 1 | 256 |
| Yes | Extremity | 56 | Alive | 0.436654 | 1 | 210 |
| Yes | Axial | 29 | Dead | 0.33 | 1 | 188 |
| Yes | Extremity | 71 | Alive | 0.504247 | 1 | 226 |
| Yes | Axial | 55 | Dead | 0.504247 | 1 | 233 |
| Yes | Axial | 27 | Alive | 0.504247 | 1 | 189 |
| Yes | Axial | 56 | Alive | 0.504247 | 1 | 190 |
| Yes | Extremity | 48 | Dead | 0.392588 | 1 | 192 |
| Yes | Axial | 55 | Alive | 0.504247 | 1 | 193 |
| Yes | Axial | 56 | Alive | 0.392588 | 1 | 194 |
| Yes | Axial | 50 | Alive | 0.33 | 1 | 195 |
| Yes | Axial | 38 | Dead | 0.436654 | 1 | 196 |
| Yes | Extremity | 53 | Alive | 0.392588 | 1 | 197 |
| Yes | Axial | 48 | Alive | 0.392588 | 1 | 257 |
| Yes | Extremity | 48 | Alive | 0.392588 | 1 | 258 |
| Yes | Extremity | 49 | Alive | 0.304089 | 1 | 198 |
| Yes | Extremity | 74 | Dead | 0.392588 | 1 | 199 |
| Yes | Axial | 12 | Dead | 0.504247 | 1 | 200 |
| Yes | Axial | 167 | Alive | 0.33 | 1 | 201 |
| Yes | Axial | 165 | Alive | 0.392588 | 1 | 263 |
| Yes | Axial | 143 | Alive | 0.392588 | 1 | 267 |
| Yes | Extremity | 42 | Dead | 0.392588 | 1 | 202 |
| Yes | Extremity | 19 | Dead | 0.392588 | 1 | 273 |
| Yes | Axial | 11 | Dead | 0.504247 | 1 | 204 |
| Yes | Extremity | 143 | Alive | 0.392588 | 1 | 274 |
| Yes | Extremity | 24 | Dead | 0.436654 | 1 | 211 |
| Yes | Extremity | 136 | Alive | 0.392588 | 1 | 205 |
| Yes | Extremity | 152 | Alive | 0.392588 | 1 | 277 |
| Yes | Axial | 108 | Alive | 0.392588 | 1 | 281 |
| Yes | Axial | 122 | Alive | 0.392588 | 1 | 206 |
| Yes | Axial | 101 | Alive | 0.33 | 1 | 208 |
| Yes | Extremity | 97 | Alive | 0.504247 | 1 | 209 |
| Yes | Extremity | 44 | Dead | 0.436654 | 1 | 210 |
| Yes | Extremity | 87 | Alive | 0.436654 | 1 | 211 |
| Yes | Extremity | 12 | Dead | 0.504247 | 1 | 212 |
| Yes | Axial | 84 | Alive | 0.392588 | 1 | 213 |
| Yes | Extremity | 57 | Alive | 0.392588 | 1 | 214 |
| Yes | Extremity | 14 | Dead | 0.436654 | 1 | 219 |
| Yes | Axial | 58 | Dead | 0.343844 | 1 | 215 |
| Yes | Extremity | 61 | Alive | 0.392588 | 1 | 217 |
| Yes | Axial | 14 | Dead | 0.33 | 1 | 218 |
| Yes | Extremity | 24 | Dead | 0.436654 | 1 | 219 |
| Yes | Axial | 30 | Dead | 0.504247 | 1 | 240 |
| Yes | Extremity | 149 | Alive | 0.504247 | 1 | 220 |
| Yes | Axial | 165 | Alive | 0.504247 | 1 | 246 |
| Yes | Axial | 12 | Dead | 0.504247 | 1 | 252 |
| Yes | Extremity | 138 | Alive | 0.436654 | 1 | 221 |
| Yes | Extremity | 122 | Alive | 0.392588 | 1 | 222 |
| Yes | Extremity | 28 | Dead | 0.392588 | 1 | 223 |
| Yes | Axial | 131 | Alive | 0.436654 | 1 | 221 |
| Yes | Axial | 101 | Alive | 0.436654 | 1 | 232 |
| Yes | Extremity | 21 | Dead | 0.392588 | 1 | 225 |
| Yes | Axial | 46 | Dead | 0.504247 | 1 | 226 |
| Yes | Extremity | 62 | Alive | 0.504247 | 1 | 260 |

| | | | | | | |
|---|---|---|---|---|---|---|
| Yes | Axial | 59 | Alive | 0.436654 | 1 | 236 |
| Yes | Extremity | 73 | Alive | 0.392588 | 1 | 227 |
| None/Unknown | Extremity | 56 | Alive | 0.734652 | 1 | 24 |
| Yes | Axial | 67 | Alive | 0.392588 | 1 | 229 |
| Yes | Axial | 11 | Dead | 0.722754 | 1 | 100 |
| Yes | Extremity | 190 | Alive | 0.392588 | 1 | 230 |
| Yes | Axial | 45 | Dead | 0.249806 | 1 | 231 |
| Yes | Axial | 56 | Dead | 0.436654 | 1 | 232 |
| Yes | Axial | 11 | Dead | 0.436654 | 1 | 247 |
| Yes | Axial | 170 | Alive | 0.504247 | 1 | 233 |
| Yes | Axial | 170 | Alive | 0.33 | 1 | 234 |
| Yes | Axial | 27 | Dead | 0.33 | 1 | 235 |
| Yes | Axial | 157 | Alive | 0.436654 | 1 | 236 |
| Yes | Extremity | 156 | Alive | 0.33 | 1 | 237 |
| Yes | Axial | 144 | Alive | 0.392588 | 1 | 238 |
| Yes | Extremity | 53 | Alive | 0.504247 | 1 | 262 |
| Yes | Axial | 109 | Dead | 0.33 | 1 | 239 |
| Yes | Axial | 14 | Dead | 0.504247 | 1 | 240 |
| Yes | Extremity | 8 | Dead | 0.33 | 1 | 242 |
| Yes | Axial | 52 | Alive | 0.392588 | 1 | 243 |
| Yes | Extremity | 155 | Alive | 0.392588 | 1 | 244 |
| Yes | Extremity | 135 | Alive | 0.392588 | 1 | 245 |
| Yes | Axial | 103 | Alive | 0.504247 | 1 | 264 |
| Yes | Axial | 86 | Dead | 0.504247 | 1 | 246 |
| Yes | Axial | 43 | Dead | 0.436654 | 1 | 247 |
| Yes | Extremity | 125 | Alive | 0.392588 | 1 | 248 |
| Yes | Axial | 32 | Dead | 0.504247 | 1 | 266 |
| Yes | Extremity | 2 | Dead | 0.773802 | 1 | 249 |
| Yes | Axial | 16 | Dead | 0.436654 | 1 | 250 |
| Yes | Axial | 28 | Dead | 0.504247 | 1 | 269 |
| Yes | Extremity | 104 | Alive | 0.33 | 1 | 251 |
| Yes | Extremity | 23 | Dead | 0.504247 | 1 | 252 |
| Yes | Axial | 22 | Dead | 0.504247 | 1 | 270 |
| Yes | Extremity | 106 | Alive | 0.33 | 1 | 253 |
| Yes | Axial | 59 | Dead | 0.436654 | 1 | 250 |
| Yes | Axial | 104 | Alive | 0.392588 | 1 | 254 |
| Yes | Axial | 14 | Dead | 0.392588 | 1 | 256 |
| Yes | Extremity | 15 | Dead | 0.392588 | 1 | 257 |
| Yes | Axial | 84 | Alive | 0.392588 | 1 | 258 |
| Yes | Extremity | 85 | Alive | 0.33 | 1 | 259 |
| Yes | Extremity | 30 | Dead | 0.504247 | 1 | 271 |
| Yes | Extremity | 75 | Alive | 0.504247 | 1 | 260 |
| Yes | Axial | 31 | Dead | 0.436654 | 1 | 282 |
| Yes | Extremity | 68 | Alive | 0.33 | 1 | 261 |
| Yes | Axial | 68 | Alive | 0.504247 | 1 | 262 |
| Yes | Axial | 65 | Alive | 0.504247 | 1 | 272 |
| Yes | Axial | 59 | Alive | 0.392588 | 1 | 263 |
| Yes | Extremity | 17 | Dead | 0.504247 | 1 | 264 |
| Yes | Extremity | 60 | Alive | 0.504247 | 1 | 266 |
| Yes | Axial | 54 | Alive | 0.773802 | 1 | 249 |
| Yes | Axial | 50 | Alive | 0.392588 | 1 | 267 |
| Yes | Extremity | 56 | Alive | 0.33 | 1 | 268 |
| Yes | Axial | 11 | Dead | 0.504247 | 1 | 269 |
| Yes | Axial | 62 | Alive | 0.504247 | 1 | 270 |
| Yes | Extremity | 21 | Dead | 0.504247 | 1 | 271 |
| Yes | Axial | 169 | Alive | 0.504247 | 1 | 272 |
| Yes | Axial | 77 | Dead | 0.392588 | 1 | 273 |
| Yes | Axial | 65 | Dead | 0.392588 | 1 | 274 |

| | | | | | | |
|---|---|---|---|---|---|---|
| Yes | Extremity | 157 | Alive | 0.504247 | 1 | 275 |
| Yes | Extremity | 148 | Dead | 0.504247 | 1 | 275 |
| Yes | Axial | 22 | Dead | 0.504247 | 1 | 285 |
| Yes | Axial | 93 | Alive | 0.392588 | 1 | 277 |
| Yes | Axial | 46 | Dead | 0.33 | 1 | 278 |
| Yes | Extremity | 27 | Dead | 0.33 | 1 | 279 |
| Yes | Extremity | 91 | Alive | 0.684919 | 1 | 182 |
| Yes | Extremity | 25 | Dead | 0.392588 | 1 | 281 |
| Yes | Axial | 61 | Alive | 0.436654 | 1 | 282 |
| Yes | Extremity | 30 | Dead | 0.33 | 1 | 284 |
| Yes | Extremity | 22 | Dead | 0.504247 | 1 | 285 |
